# Genome-wide association study of antidepressant response: involvement of the inorganic cation transmembrane transporter activity pathway

**DOI:** 10.1186/s12888-016-0813-x

**Published:** 2016-04-18

**Authors:** Enrico Cocchi, Chiara Fabbri, Changsu Han, Soo-Jung Lee, Ashwin A. Patkar, Prakash S. Masand, Chi-Un Pae, Alessandro Serretti

**Affiliations:** Department of Biomedical and NeuroMotor Sciences, University of Bologna, Bologna, Italy; Department of Psychiatry, Korea University, College of Medicine, Seoul, Republic of Korea; Department of Psychiatry, The Catholic University of Korea College of Medicine, Seoul, Republic of Korea; Department of Psychiatry and Behavioural Sciences, Duke University Medical Center, Durham, NC USA; Global Medical Education, New York, NY USA

**Keywords:** Pharmacogenomics, GWAS, Major depression, Antidepressant, Gene, Pathway, Calcium channel, Cation transmembrane transporter

## Abstract

**Background:**

Genome-wide association studies (GWAS) represent the current frontier in pharmacogenomics. Thousands of subjects of Caucasian ancestry have been included in previous GWAS investigating antidepressant response. GWAS focused on this phenotype are lacking in Asian populations.

**Methods:**

A sample of 109 major depressive disorder (MDD) patients of Korean origin in antidepressant treatment was collected. Phenotypes were response and remission according to the Hamilton Rating Scale for Depression (HRSD). Genome-wide genotyping was performed using the Illumina Human Omni2.5-8 platform. The same phenotypes were used in the STAR*D level 1 (*n* = 1677) for independent replication. In order to corroborate findings and increase the comparability between the two datasets, three levels of analysis (SNPs, genes and pathways) were carried out. Bonferroni correction, permutations, and replication across samples were used to reduce the risk of false positives.

**Results:**

Among the genes replicated across the two samples (permutated *p* < 0.05 in both of them), *CTNNA3* appeared promising. The inorganic cation transmembrane transporter activity pathway (GO:0022890) was associated with antidepressant response in both samples (*p* = 2.9e-5 and *p* = 0.001 in the Korean and STAR*D samples, respectively) and this pathway included *CACNA1A*, *CACNA1C*, and *CACNB2* genes.

**Conclusions:**

The present study supported the involvement of genes coding for subunits of L-type voltage-gated calcium channel in antidepressant efficacy across different ethnicities but replication of findings is required before any definitive statement.

**Electronic supplementary material:**

The online version of this article (doi:10.1186/s12888-016-0813-x) contains supplementary material, which is available to authorized users.

## Background

### Previous findings

Antidepressant response was demonstrated to have a relevant genetic component, exemplified by the contribution of common single nucleotide polymorphisms (SNPs) that was estimated to explain 0.42 of variance in this phenotype [1]. Three main GWAS (genome-wide association studies) investigated antidepressant response in samples of mainly Caucasian ancestry [2–4] and several re-analyses [5] or meta-analysis [6, 7] were performed on these data.

Less GWAS data are available in regard to antidepressant efficacy in samples of Asian ancestry compared to Caucasian populations and previous studies performed only SNP-level analysis.

One previous GWAS on Japanese patients suggested the possible involvement of *CUX1* (Cut-Like Homeobox 1) gene that codes for a member of the homeodomain family of DNA binding proteins [8]. It may regulate gene expression, morphogenesis, and differentiation and it may also play a role in the cell cycle progression.

To the best of our knowledge, only another GWAS investigated antidepressant response in a Korean population and it reported two SNPs in the *AUTS2* (Autism Susceptibility Candidate 2) gene (rs7785360 and rs12698828) as genome-wide significant [9]. *AUTS2* codes for a nuclear protein that is expressed in the central nervous system in humans, especially in some hippocampal areas and it has been implicated in neurodevelopmental disorders, schizoaffective and bipolar affective disorders [9].

Another recent GWAS included a relevant number of Asian subjects especially from Taiwan and secondly from Japan and it did not report genome-wide significant results. Among the top genes, *NRG1* (neuregulin-1) is particularly interesting since this gene is involved in many aspects of brain development and it was associated with schizophrenia risk [10].

### Methodological considerations

Recent studies suggested that genes (and not only SNPs) should be investigated in GWAS since they represent the functional units of genome. Molecular pathways are more complex functional units that represent the following step in the development of multi-marker analysis in GWAS. Several methods have been applied to identify molecular pathways that may be involved in antidepressant efficacy, and they can be classified according to the criteria used to define gene sets, to select p values to be considered in the analysis and the statistics used to assess the significance of each gene set [11]. Gene sets can be defined a priori including pathways reported by available databases (e.g. KEGG, BioCarta) or according to the over-representation of some gene sets in the data. In detail, genes including trait-associated SNPs can be grouped in functionally-enriched sets according to physical and genetic interaction data, predicted protein interaction, pathway and molecular interaction data as implemented by GeneMANIA [12]. After the definition of gene sets, several methods are available to test their significance in GWAS. Gene-set statistics can take into account the most significant p value in each gene (e.g. [13]) or SNPs with p under a defined threshold (e.g. [14, 15]) and a permutation-based procedure is usually applied to test the significance of each gene set against the null hypothesis. Another possible method takes into consideration all the p values in each gene set and it assigns a score to each gene set using the Z-statistic [16]. Starting from the assumption that only some gene sets may be relevant to a particular trait, we performed a pathway analysis based on two steps. Firstly, we performed an over-representation analysis using GeneMANIA [12] in order to identify functionally-enriched gene sets in our data and secondly enriched gene sets were investigated comparing the distribution of independent SNPs with p values under defined thresholds within each target gene set to a random gene set. The same two-step method was previously applied [17].

### Aims of the present study

Given that antidepressant response was less investigated by GWAS in Asian samples compared to Caucasian ones, the present GWAS aimed to investigate antidepressant efficacy in a relatively small but homogenous sample of depressed patients of Korean origin. The analysis was based on three levels, i.e. SNPs, genes and pathways in order to not overlook the correlation structures among multiple SNPs within genes and multiple genes within pathways. This is the first GWAS that performed also gene- and pathway-level analyses on an Asian population. Finally, this study directly compared findings obtained in the Korean GWAS with those from the STAR*D (Sequenced Treatment Alternatives to Relieve Depression) GWAS (mainly including subjects of white non-Hispanic origin) in order to identify shared genetic factors modulating antidepressant response across different ethnic groups.

## Methods

### Samples

#### Korean sample

Patients were consecutively collected among patients admitted to the psychiatric unit of the Department of Psychiatry, Catholic University of Korea College of Medicine, Seoul, Korea. Inclusion criteria were: 1) age between 18–80, 2) current episode of major depression in patients with major depressive disorder (MDD) diagnosis, 3) current antidepressant pharmacological treatment. Exclusion criteria were: 1) current or recent substance abuse (at least during the past 6 months), 2) severe or unstable medical condition that may impair evaluations, 3) neurological disorders, 4) non-Asian ethnicity, 5) other treatments than antidepressants with the exception of anti-anxiety drugs, 6) poor understanding or fluency of Korean language, 7) mental retardation.

Clinical and socio-demographic data were collected by interviews or revision of the clinical charts. Diagnosis of MDD and current depressive episode were performed according to the Diagnostic and Statistical Manual of mental disorders IV, text revised (DSM-IV-TR) criteria by the Semi-structured clinical interview for DSM-IV (SCID-I) [18]. Symptom severity was evaluated by the Hamilton Rating Scale for Depression (HRSD17) [19]. All patients were evaluated for symptom severity at admission and discharge (observation period of 4–6 weeks). Evaluations were performed by trained interviewers blind to genetic data. Anxiolytics (alprazolam, lorazepam, clonazepam or buspirone) were the only concomitant psychotropic medications allowed. The clinical-demographic characteristics of the included patients are summarized in Additional file 1: Table S1.

### Ethics, consent and permissions

All the patients were informed in detail about the aims and the procedures of the study and they signed a written informed consent prior inclusion into the study. The protocol and the written informed consent were approved by the local ethical committee (Catholic Medical Center, Clinical Research Coordinator Center; approval number HC10TISI0031).

### STAR*D sample

The sample under investigation was retrieved from the public available Sequenced Treatment Alternatives to Relieve Depression (STAR*D) study, level 1. Detailed descriptions of the study design and study population are detailed elsewhere [20]. In brief, non psychotic MDD (DSM-IV criteria) patients with age between 18 and 75 years were enrolled from primary care or psychiatric outpatient clinics and a current 17-item Hamilton Depression Rating score of ≥14 by independent raters was obtained. All patients received a systematic assessment battery at entry and were treated by a psychiatrist (trained to deliver care and measure outcomes in patients with MDD) using a series of model practice procedures consistent with expert recommendations. Severity of depression was assessed using the 16-item Quick Inventory of Depressive Symptomatology-Clinician Rated (QIDS-C) [21] at baseline, weeks 2, 4, 6, 9, and 12. All patients received citalopram in level 1. Concomitant treatments for current general medical conditions (as part of ongoing clinical care), for associated symptoms of depression (e.g., sleep, anxiety, and agitation), and for citalopram side effects (e.g., sexual dysfunction) were permitted on the basis of clinical judgment. Stimulants, anticonvulsants, antipsychotics, alprazolam, non-protocol antidepressants (except trazodone ≤200 mg at bedtime for insomnia), and depression-targeted psychotherapies were proscribed [22]. The clinical-demographic characteristics of the patients are summarized in Additional file 1: Table S1.

In order to avoid possible biases due to the consideration of mild depression forms, we only included patients with an entry QIDS-SR score greater or equal to 10 (significantly depressed) that corresponds to a Hamilton Depression Rating score (HDRS) greater or equal to 14.

### Definition of phenotypes

According to standard criteria, in the Korean sample response was defined as a ≥50 % symptoms reduction (HRSD17 score) from baseline to discharge and remission was defined as the HRSD17 score ≤7 at discharge. In the STAR*D sample, response was defined as a ≥50 % symptoms reduction (QIDS-SR score) from baseline to week 12 while remission was defined as the QIDS-SR score ≤5 at week 12. The last observation carried forward method (LOCF) was applied when baseline and at least one post-baseline observation were available, according to previous studies that analyzed STAR*D data [22]. The choice to not use week 6 response and remission in the STAR*D (that would correspond to the time end-point in the Korean sample) was due to the observation that the most part of STAR*D patients reached response and remission at or after week 8 [22]. Some clinical differences between the two samples (e.g. inpatient vs. outpatient status) were probably responsible for the observed similar response and remission rates at week 6 in the Korean sample and week 12 in the STAR*D (see Additional file 1: Table S1).

### Genotyping

#### Korean sample

Genotyping was performed using Illumina Human Omni2.5-8 platform. The HumanOmni2.5-8 BeadChip delivers comprehensive coverage of both common and rare SNP content from the 1000 Genomes Project (1kGP; MAF > 2.5 %), designed to be maximally informative for diverse world populations. It is able to identify 2,379,855 polymorphisms. The scan protocol followed the standard Illumina procedures using Illumina BeadXpress Reader. The microarray data was analyzed with GenomeStudio V2011.1 and GT module 1.9.4 using default analysis settings. Each SNP is analyzed independently to cluster and identify genotypes. Genotype calls are generated by comparing experimental data with those in the supplied cluster file(*.egt). Calls are generally highly accurate and unambiguous for high quality samples.

### STAR*D sample

STAR*D patients were genotyped using the Affymetrix Human Mapping 500 k Array Set. The Mapping 500 K Array Set is comprised of two arrays, each capable of genotyping on average 250,000 SNPs. One array uses the Nsp I restriction enzyme (~262,000 SNPs), while the second uses Sty I (~238,000 SNPs). Together, the family of GeneChip Mapping products offers solutions for genotyping ~500,000 SNPs. Given that the number of SNPs genotyped in this sample is lower compared to that available in the Korean sample (500,000 vs. 2,379,855) and different platforms were used, only a partial overlap between genotyped polymorphism could be expected.

### Statistical analysis

#### Quality control

Quality control of genome-wide data was performed according to the following criteria in both samples. SNPs were pruned out according to linkage disequilibrium (r2 > 0.8), minor allele frequency (MAF < 0.01), individual genotyping rate (>0.05 missing genotypes) and Hardy-Weinberg equilibrium (*p* < 0.001). Results of quality control are shown by QQ plots in Additional file 2: Figure S1. Identity-by-descent (IBD) analysis was used to identify related subjects (IBD > 0.1875 [23]) in the STAR*D. 21 pairs of related subjects were identified (accordingly to what previous reported [24]) and one subject per pair was excluded from the analysis.

#### Individual polymorphisms association analysis

The analysis of individual SNPs association with the dichotomous phenotypes (remission and response) was performed by logistic regression models, including as covariates potential stratification factors as discussed in paragraph 2.3.3. PLINK software was used for these analysis [25].

SNPs with *p* < 0.05 in the Korean sample were tested in the STAR*D and those showing *p* < 0.05 in both samples were used to select genes that were included in the functional enrichment analysis and gene set enrichment analysis (GSEA). The direction of the association was not a considered a criteria to select SNPs to include in the following levels of analysis since probable relevant pharmacogenetic associations were found to show opposite direction in different populations (e.g. 5-HTTLPR in the SLC6A4 gene [26]). We underline that SNP-level analysis was exploratory only and it mainly aimed to identify signals to include in the following steps of the study (gene- and pathway-level analysis).

#### Selection of covariates

We considered sex and age as covariates in both samples. The entry level of HRSD17 (Korean sample) and QIDS-SR (STAR*D) was also used as covariate for remission only since the variable highly affected this phenotype in both samples (*p* = 8.03e-7 and *p* = 1.75e-10 in the Korean sample and STAR*D, respectively) while it did not affect response. In the STAR*D also ancestry was included among covariates. Briefly, a complete agglomerative clustering was applied, based on a multidimensional scaling of a matrix of pairwise identity-by-state (IBS) values between samples, and clusters were defined on the base of the pairwise population concordance test (PPC < 0.0001, −-cluster and –-ppc options in Plink according to [25]). In the Korean sample all subjects were of Korean origin thus the inclusion of the covariate ancestry was not required.

#### Functional enrichment analysis

The SNPs with *p* < 0.05 in both the Korean and STAR*D samples for each phenotype were annotated (PLINK function --annotate) using a 20 Kbp window upstream and downstream of each gene. In order to take into account all the possible genes related with the phenotypes under analysis we enriched the obtained gene lists using Cytoscape [27] and the GeneMania [28] plugin. GeneMania uses a large database of functional interaction networks from multiple organisms and each related gene is traceable to the source network used to make the prediction [28]. This plugin also identifies the biological processes in which these genes play a role, on the basis of the Gene Ontology international database [29]. Gene Ontology biological processes are defined as pathways in the present paper since they can be used as functional units to perform a pathway analysis despite the description of the dynamics or dependencies that would be required to fully describe a pathway was not performed yet in the context of the Gene Ontology project.

At the end of the process 83 and 77 genes including SNPs with *p* < 0.05 in both samples or interacting with them were identified for remission and response, respectively. These genes play a role in 10 and 7 biological pathways (Gene Ontology [29]), respectively. Graphic representation of genes and pathways are shown in Additional file 3: Figure S2. Additional file 4: Table S2 and Additional file 5: Table S3 report the characteristics of genes and pathways under analysis for response and remission phenotypes, respectively. These genes and pathways were then used for the gene set enrichment analysis (GSEA).

#### Gene set enrichment analysis (GSEA)

As previously described [30], results referred to individual SNPs can be analyzed as organized in pathways by comparing the distribution of SNPs with p value under a defined threshold between the index pathway and a random pathway (Fisher exact test). The same method can be applied to genes, i.e. the distribution of SNPs with p under a defined threshold within a candidate gene is compared with that of a random gene.

In the present study, genes included in each of the pathways selected in 2.3.4 were firstly imputed using IMPUTE2 (http://mathgen.stats.ox.ac.uk/impute/impute_v2.html) and 1000 Genomes data (NCBI Build 36 (dbSNP b126) for the STAR*D and GRCh37 Build for the Korean sample) as reference panel. Imputed SNPs were pruned according to linkage disequilibrium (r2 ≥ 0.8) and poor imputation quality (info < 0.8). Secondly, variations showing *p* < 0.05 in each pathway of interest were tested for a significant different distribution (Fisher exact test) compared to a random pathway. Each random pathway was matched with the index pathway in terms of number of SNPs within it and intragenic position of the SNPs but with random distribution within the whole genome.

The same procedure was performed for each gene included in the candidate pathways, i.e. variations showing *p* < 0.05 in each gene of interest were tested for a significant different distribution (Fisher exact test) compared to a random gene. Nominal p values under 0.05 were permutated randomly re-assigning the SNPs in the two groups 100,000 times for each pathway/gene. The present method based on a Fisher exact test avoids the restriction of the focus on the “top” p values of the pathway/gene, and thus it is able to detect more subtle signals compared to methods that are based on the smallest individual p values in the pathway [31]. In fact, methods based on Fisher’s exact test resulted to be among those with the highest power in such type of analysis [32]. We finally underline that GSEA investigated a different hypothesis compared to functional enrichment, since it did not search for interactions among genes but it tested if the distribution of SNPs with p value below a threshold in the gene set compared to what observed by chance (i.e. in a random set) was different. Further, the genes included in the functional enrichment analysis were selected only according to the criteria of including at least one SNP with *p* < 0.05 in both samples while our GSEA took into account the p values of all the SNPs within each gene.

### Multiple testing correction

Multiple testing corrections were performed according to the Bonferroni correction and permutation. The p value for a significant individual SNP result was set at 0.05/585,693 = 8e-8 in the Korean sample for remission and response. The liberal p threshold of 0.05 was used to select individual SNPs to be tested in the replication sample (STAR*D).

The significance of results of gene- and pathway-based analyses was tested by 100,000 permutations after a first selection based on the Bonferroni correction (*p* = 0.05/83 = 0.0006 for gene-based analysis; *p* = 0.05/10 = 0.005 and *p* = 0.05/7 = 0.007 for pathway analysis, remission and response phenotypes, respectively).

### Power of the study

When considering an intermediate MAF of 0.20 and the response phenotype, the Korean sample provided a power of 0.72 for a SNP with OR = 2.84, while the STAR*D sample provided a power of 0.61 when considering a SNP with OR = 1.26 (data referred to SNPs rs2112460 and rs2299267, respectively, were used for power estimation).

For the purpose of SNP-level power analysis a nominal p value of 0.05 was considered because this analysis was aimed to be an exploratory analysis to provide data for subsequent analyses (gene- and pathway-base analyses). Genetic power calculator was used to estimate the power of the study [33].

For gene- and pathway-based analyses the power was estimated using G*Power 3.1 [34]. When considering the significant gene/pathway with the smallest number of total SNPs (the *MTRF1L* gene in the STAR*D sample that included only 26 SNPs) we had a power of about 0.80 setting alpha to 0.0006 (Bonferroni corrected p) if the proportion of SNPs with p under the significance threshold was 29 % more than expected by chance and a power of 0.95 in the observed case (the proportion of SNPs with p under the significance threshold was 33 % more than expected by chance). When considering the gene/pathway with the highest number of total SNPs (the GO:0022890 pathway in the Korean sample that included 7531 SNPs) we had a power of about 0.80 setting alpha to 0.007 (Bonferroni corrected p) if the proportion of SNPs with p under the significance threshold was 1.9 % more than expected by chance and of a power 0.76 in the observed case (the proportion of SNPs with p under the significance threshold was 1.3 % more than expected by chance).

## Results

### Individual SNP association analysis

The analysis of the remission phenotype provided 25,976 SNPs with *p* < 0.05 in the Korean sample, but no one survived after the Bonferroni correction. Of these 25,976 SNPs, 1455 (Additional file 6: Table S4) were available in the STAR*D dataset and 76 (Additional file 7: Table S5) showed *p* < 0.05 also in this sample; the top six SNPs for remission are reported in Table 1A.

**Table 1:** Description of the 6 best SNP for remission (A) and response (B) in both the analyzed samples

			Korean sample				STAR*D			
SNP	Chromosome base pair	Gene	MAF	Odds ratio	Statistics	*p*	MAF	Odds ratio	Statistics	*p*
A										
rs9315310	13	NBEA (165 Kbp)	0.28	3.67	3.1.32	<0.00001	0.21	1.21	2.112	0.034700
	35351431									
rs672170	6	RGS17	0.45	3.48	3.130	0.001748	0.36	1.18	2.155	0.031140
	153345185									
rs2532560	12	PARP11 (179 Kbp)	0.14	0.16	−3.079	0.002075	0.20	1.20	2.002	0.045270
	4161871									
rs2831440	21	N6ATM1 (800 Kbp)	0.39	3.53	3.045	0.002327	0.32	0.79	−3.126	0.001774
	29428098									
rs766127	6	RGS17 |MTRF1L |FBXO5	0.42	2.89	2.954	0.003134	0.30	1.21	2.374	0.017580
	153313832									
rs4737771	8	CRH (100 Kbp)	0.20	0.24	−2.943	0.003246	0.12	1.26	2.005	0.044920
	67192379									
B										
rs49411	3	FHIT	0.50	0.36	−3.292	0.0009939	0.49	0.85	−2.277	0.022760
	59757575									
rs10997242	10	CTNNA3	0.29	0.32	−3.025	0.0024890	0.16	1.30	2.788	0.005303
	68336295									
rs16873129	7	RAPGEF5	0.17	0.32	−2.912	0.0035970	0.07	1.32	2.103	0.035470
	22145084									
rs2299267	7	PON2	0.39	0.46	−2.858	0.0042650	0.17	1.26	2.486	0.012930
	95061921									
rs2112460	19	CACNA1A	0.21	2.84	2.759	0.0057920	0.48	1.19	2.497	0.012530
	13590412									
rs521093	1	EPS8L3 (62 Kbp)	0.10	5.13	2.728	0.0063760	0.20	1.21	2.096	0.036040
	110368230									

When a SNP is localized in an intergenic region, the nearest gene is reported with distance in parenthesis

*MAF* minor allele frequency

The analysis of the response phenotype provided 24,606 SNPs with *p* < 0.05 in the Korean sample, but no one survived after the Bonferroni correction. Of these 24,606 SNPs, 1392 (Additional file 8: Table S6) were available in the STAR*D dataset and 77 (Additional file 9: Table S7) showed *p* < 0.05 also in this sample; the top six SNPs for response are reported in Table 1B.

Manhattan plots referred to remission and response phenotypes in the Korean sample are shown in Additional file 10: Figure S3 and top SNPs (*p* < 10e-05) in this sample are reported in Additional file 11: Table S8.

### Gene association analysis

The association analysis between the 84 genes harbored by the SNPs showing *p* < 0.05 in both the two samples and remission showed several replicated genes that survived after permutations: ATP/GTP Binding Protein-Like 1 (*AGBL1*), Cytochrome B5 Type A (Microsomal) (*CYB5A*), Mitochondrial Translational Release Factor 1-Like (*MTRF1L*) and Regulator Of G-Protein Signaling 22 (*RGS22*) (Table 2A). For the response phenotype the following genes (tot. genes = 77) survived after permutations in both samples: Catenin (Cadherin-Associated Protein), Alpha 3 (*CTNNA3*) and Heparan Sulfate 6-O-Sulfotransferase 3 (*HS6ST3*) (Table 2B).

**Table 2:** Genes that resulted associated with remission (A) and response (B) in both the analyzed samples

		Korean sample				STAR*D			
Gene	Gene full name	SNPs with *p* < 0.05		SNPs (tot N)	Permuted *p*	SNPs with *p* < 0.05		SNPs (tot N)	Permuted *p*
		N true	N false	/		N true	N false	/	
A									
*AGBL1*	ATP/GTP Binding Protein-Like 1	268 (7 %)	3454 (92 %)	3722	<0.00001	65 (9 %)	601 (90 %)	656	8.9e-5
*CYB5A*	Cytochrome B5 Type A (Microsomal)	216 (58 %)	152 (41 %)	368	<0.00001	25 (37 %)	41 (62 %)	66	3.9e-5
*MTRF1L*	Mitochondrial Translational Release Factor 1-Like	85 (47 %)	95 (52 %)	180	<0.00001	10 (38 %)	16 (61 %)	26	0.0003
*RGS22*	Regulator Of G-Protein Signaling 22	129 (29 %	311 (70 %)	440	<0.00001	25 (46 %)	29 (53 %)	104	0.0001
B									
*CTNNA3*	Catenin (Cadherin-Associated Protein), Alpha 3	446 (6 %)	6628 (93 %)	7074	<0.00001	118 (11 %)	952 (88 %)	1070	<0.00001
*HS6ST3*	Heparan Sulfate 6-O-Sulfotransferase 3	305 (15 %)	1626 (84 %)	1931	<0.00001	50 (14 %)	298 (85 %)	348	8.9e-5

True/False are referred to the number (and percentage) of SNPs with *p* < 0.05 in the considered subset. The expected number (due to chance) of N true corresponds to 5 % of the total

### Pathway association analysis

The association analysis between the molecular pathways that are harbored by the replicated genes and response showed only one pathway that survived after permutations in both samples: GO:0022890 (Gene Ontology ID), relative to inorganic cation transmembrane transporter activity. The investigated genes pertaining to this pathway are Calcium Channel, Voltage-Dependent, P/Q Type, Alpha 1A Subunit (*CACNA1A*), Calcium Channel, Voltage-Dependent, P/Q Type, Alpha 1C Subunit (*CACNA1C*), Calcium Channel, Voltage-Dependent, Beta 1 Subunit (*CACNB1*), Calcium Channel, Voltage-Dependent, Beta 2 Subunit (*CACNB2*), Cytochrome B5 Type A (Microsomal) (*CYB5A*), Solute Carrier Family 4 (Sodium Bicarbonate Cotransporter), Member 4 (*SLC4A4*), Solute Carrier Family 4 (Sodium Bicarbonate Cotransporter), Member 5 (*SLC4A5*), Solute Carrier Family 4 (Sodium Bicarbonate Cotransporter), Member 7 (*SLC4A7*), Solute Carrier Family 6 (Neurotransmitter Transporter, Serotonin), Member 4 (*SLC6A4*), Solute Carrier Family 6 (Neurotransmitter Transporter, Serotonin), Member 2 (*SLC6A2*). Table 3 reports the characteristics and statistics of this pathway. No pathway survived in both samples after multiple-testing correction when the remission phenotype was analyzed.

**Table 3:** Results pertaining to the only molecular pathway that was replicated in both samples (N tot. molecular pathways = 7) for the response phenotype

		Korean sample				STAR*D			
GO ID	Pathway name	SNPs with *p* < 0.05		SNPs (tot N)	Permuted *p*	SNPs with *p* < 0.05		SNPs (tot N)	Permuted *p*
		True	False	/		True	False	/	
GO:0022890	Inorganic cation transmembrane transporter activity	478 (6 %)	7053 (93 %)	7531	2.9e-5	123 (8 %)	1317 (91 %)	1440	0.001

True/False are referred to the number (and percentage) of SNPs showing association with *p* < 0.05 in the considered subset. The expected number (due to chance) of N true corresponds to 5 % of the total

## Discussion

The present study investigated the genetic markers of antidepressant response/remission in two independent genome-wide datasets through SNP-, gene- and pathway-based analyses. The original sample included Korean MDD subjects and the STAR*D study served as independent replication on a different ethnicity. Despite the limited sample size of the Korean sample, we underline that relatively limited data exist on Asian populations [8, 10] and only one previous study was performed on a Korean sample [9]. The use of three levels of analysis, i.e. SNPs, genes and molecular pathways, was expected to reduce the risk of false positive findings despite our results should be considered as exploratory given the sample size issue. The number of SNPs that showed *p* < 0.05 in both GWAS was approximately the same expected by chance, but SNP-level analysis was not intended to discover associated SNPs but to provide a relaxed criteria to select findings for subsequent levels of analysis. Gene- and pathway-based analyses were corrected for multiple-testing (Bonferroni correction and permutations), thus providing an acceptable support for the results of these analysis that can be hints for future studies.

SNP-level analysis did not retrieve any genome-wide significant result, but some potential interesting findings emerged when looking at the SNPs showing *p* < 0.05 in both the samples. The top finding for remission was rs9315310 which nearest gene is *NBEA* (neurobeachin). *NBEA* codes a multidomain scaffolding protein primarily expressed in the brain where it is involved in trafficking of vesicles containing neurotransmitter receptors, specifically GABA and glutamate receptors [35, 36]. No previous study suggested the involvement of this gene in antidepressant efficacy or MDD, but the glutamatergic and gabaergic systems are known to be involved in antidepressant mechanisms of action [37, 38]. Another interesting finding for remission was rs4737771 that is located 100 Kbp far from the *CRH* (corticotropin releasing hormone) gene. Indeed, the inhibition of *CRH* gene expression by antidepressant drugs was demonstrated [39] and polymorphisms within *CRHR1* and *CRHR2* (*CRH* receptors 1 and 2) were associated with antidepressant response [26]. The other SNPs with *p* < 0.05 in both samples (for remission) are shown in Additional file 7: Table S5 and we underline the presence of the following genes: 1) *SLC6A3* (solute carrier family 6 member gene 3), that codes for the dopamine transporter and other polymorphisms within it were previously associated with antidepressant response by candidate gene studies [40, 41]); 2) *CACNA1A* (calcium channel, voltage-dependent, P/Q type, alpha 1A subunit) and *CACNB2* (calcium channel, voltage-dependent, beta 2 subunit), that were demonstrated as the top signals in a GWAS investigating risk loci with shared effects among major psychiatric disorders [42]; and 3) *NRG3* (neuroregulin 3) gene that is a ligand of *ERBB4* which signaling is involved in neurotransmission, synaptic plasticity, and ketamine antidepressant action [43]. Interestingly, a signal in *NRG1* was recently identified as promising marker of antidepressant response by a meta-analysis [10].

The most interesting SNPs showing *p* < 0.05 for response in both samples were in the *CTNNA3* (catenin (cadherin-associated protein), alpha 3) and *CACNA1A* genes (Table 1).

The *CTNNA3* (catenin alpha-3) gene was associated with the risk of schizophrenia [44] and intracellular signaling through beta-catenin translocation and AKT/PKB pathways have implicated in antidepressant-induced hippocampal cell proliferation [45]. Most importantly, a suggestive signal in the *CTNNA3* gene was recently associated with antidepressant response in a GWAS including mainly patients of Asian ancestry [10]. Among SNPs with *p* < 0.05 for response (Additional file 9: Table S7), a SNP near to *SLC6A3* was again present (see results referred to the remission phenotype). Further, the intronic rs582854 polymorphism in the serotonin receptor 2A (*HTR2A*) gene was only at 17 Kbp from a previous signal found in this gene [46] and in linkage disequilibrium (*r*^2^ = 0.6 and D’ = 0.97) with rs6313 and rs6311 in the CEU population (1000 Genomes phase 3 data). *HTR2A* is a replicated candidate gene for involvement in antidepressant response, rs6313-rs6311 were associated with this phenotype by candidate gene studies [26] and rs6313 was confirmed by a recent meta-analysis [47].

Compared to a previous GWAS of antidepressant response in a Japanese sample reporting the *CUX1* rs365836 and rs201522 as top findings [8], we found that weak signals came from this gene (remission: rs10240601 *p* = 0.028; rs73412036 *p* = 0.039; rs12668172 *p* = 0.026; rs11971570 *p* = 0.025; response: rs2694159 *p* = 0.040). The *CUX1* rs365836 and rs201522 are ~ 99 far from rs2694159 and ~ 88 Kbp from rs11971570, despite no evidence of linkage disequilibrium among these SNPs was found in Asian populations according to 1000 Genomes data. No *CUX1* SNP with *p* < 0.05 was found in the STAR*D.

Only one previous GWAS investigated antidepressant response in a Korean population [9] and two SNPs in the *AUTS2* gene (rs7785360 and rs12698828) were genome-wide significant. In the present study several SNPs in the *AUTS2* gene showed p values < 0.05 in the Korean sample (remission: rs17141924 *p* = 0.01; rs4718974 *p* = 0.03; response: rs77393802 *p* = 0.03; rs10950209 *p* = 0.008; rs10234816 *p* = 0.04), and one SNP showed *p* < 0.05 in both samples (rs7459368: *p* = 0.0007 and *p* = 0.03 in the STAR*D and the Korean sample, respectively), but these SNPs are about 700 Kbp from the previous findings and no evidence of linkage disequilibrium exists among them.

Gene-based analysis and pathway analysis provided some additional findings compared to SNP-level analysis. *AGBL1, CYB5A, MTRF1L,* and *RGS22* emerged from SNP-based analysis and they were associated with remission also in gene-based analysis, as well as for *CTNNA3* and *HS6ST3* genes and response. A role of *CTNNA3* in antidepressant response is supported by literature as we discussed, while no previous study supporting the involvement of the other findings of gene-based analysis was published. Anyway, several of these genes are related to processes involved in cell survival, proliferation and migration, that are encompassed in the neural plasticity theory of depression [48]. *AGBL1* has a role in controlling the length of the polyglutamate side chains on tubulin and this process is critical for neuronal survival and the lack of such control results in neurodegeneration in mice [38]. *HS6ST3* generates structures required for interactions between heparan sulfate and a variety of proteins. These interactions are implicated in cell proliferation, differentiation, adhesion and migration [49]. *RGS22* (regulator of G-protein signaling 22) has been implicated in the processes of cell migration in cancer [48]. *CYB5A* (cytochrome B5 type A (microsomal)) was related to autophagy induction, concomitant with reduced proliferation and migration/invasion in cancer cells [50].

Despite their limited evidence due the small sample size, some genes were significant (*p* < 0.0006) in the Korean sample but they were not replicated in the STAR*D. They included *NR3C2* (nuclear receptor subfamily 3 group C member 2), *SLC6A4* (solute carrier family 6 member 4), *HTR2A*, *SLC25A4* (solute carrier family 25 member 4), *SLC6A3*, and *CACNA1A* for both response and remission. *NR3C2* codes for mineralocorticoid receptor 1 (MR1) and antidepressants were shown to modulate MR hormone-binding [51] and expression [52] in the context of the corticosteroid receptor hypothesis of depression. *SLC6A4* is a known candidate gene for involvement in antidepressant response [26] even if the most studied polymorphism is an insertion/deletion thus it was not available in this sample. Quantitative proteomic analyses on mice hippocampal tissue implicated *SLC25A4* product in serotonergic antidepressant action [53]. *HTR2A, SLC6A3*, and *CACNA1A* are discussed elsewhere in this paragraph. *HTR2A* (response, *p* = 0.03), *SLC6A3* and *CACNA1A* (remission, *p* = 0.05 and *p* = 0.03, respectively) gene-based analyses showed non-significant trends in the STAR*D.

The only pathway that survived multiple-test correction in both samples was GO:0022890 related to inorganic cation transmembrane transporter activity (Table 3). This pathway included several genes coding for calcium channels (*CACNA1A*, *CACNA1C*, *CACNB1, CACNB2*). As we discussed above, polymorphisms in *CACNA1A* and *CACNB2* were demonstrated to be trans-diagnostic markers of major psychiatric disorders [42]. *CACNB1* expression was modified in response to nortriptyline in hippocampal mice tissues [53]. Preclinical studies supported a role of *CACNA1C* in the pathogenesis of mood disorders [54] and the gene was associated with MDD, bipolar disorder, schizophrenia and autism spectrum disorders [55]. These genes code for subunits of the L-type voltage-gated calcium channel (LTCC) that is mainly involved in coupling of cell membrane depolarization to transient increase of the membrane permeability for calcium, leading to potential changes in intracellular signaling, gene transcription, and synaptic plasticity. These functions of LTCC involved brain regions that are pivotal in MDD pathogenesis such as the hippocampus and amygdale. LTCC antagonists were suggested as potential antidepressant molecules alone or in combination with SSRIs [55] and imipramine was also demonstrated to modulate Ca(2+) intracellular rise in rat hippocampus [56].

In the Korean sample only, the GO:0015844 pathway (monoamine transport) survived after multiple-testing correction for both response and remission (*p* = 7.00e-05 and *p* = 0.0006, respectively). This result is in line with the gene-based analysis in the Korean sample, but it was not replicated and thus poorly relevant given the small sample size.

Some limitations of the present study should be considered. First, the limited size of the Korean sample, thus the present findings should be interpreted cautiously. Quite large samples of white race were previously collected for GWAS on antidepressant efficacy, while less data are available on samples of Asian ancestry [8–10], thus this study can contribute to expand our knowledge in the field and provide data for future meta-analysis (no meta-analysis including only subjects of Asian ancestry was performed yet). The risk of false positive findings was faced through the use of multi-level analysis (SNP-, gene, and pathway-based), permutation, and replication to corroborate findings. The imputation of data only at level of gene- and pathway analyses have limited the comparability of the two datasets at SNP-level, but this choice was based on resource/benefit ratio considerations. Indeed, it would have been more resource consuming and a Korean reference panel provided by the 1000 Genomes or HapMap projects is not available. The Korean population was demonstrated to have a distinctive genetic architecture [57] and decreased imputation quality due to the lack of a Korean reference panel [58] can be balanced in the context of multimarker tests (gene- and pathway-based tests) while it could affect the results at SNP level. The use of the STAR*D as replication sample can be seen as a further limitation because subjects were mainly of non-Hispanic white origin. Some polymorphisms were found overrepresented in certain ancestry groups [59] but similar genes have been often implicated in the same phenotype across populations (e.g. rheumatoid arthritis [60] and also antidepressant response [26]). Given that the present study was not limited to the analysis of individual polymorphisms but included a gene-based analysis and pathway analysis, this approach is expected to increase the comparability between the two datasets. Anyway, we examined the MAFs of the SNPs reported in Table 1 across the main ethnic groups of the STAR*D (white non-Hispanic, white Hispanic and African-American). For 5/12 SNPs and 9/12 SNPs the MAF difference was less than 10 % and less than 15 %, respectively. Other stratification factors between the two samples should be considered, such as treatment and other clinical variables. Regarding treatment, it was not standardized, but the most part (76 %) of patients included in the Korean sample were treated with the SSRI paroxetine and all patients in STAR*D level 1 were treated with the SSRI citalopram. Inter-class pharmacogenetic differences between antidepressant drugs are possible [61] while differences among drugs of the same class are less known. Anyway, non-standardized treatment provides the advantage of being more similar to real clinical settings. Treatment duration was also not standardized, but it was within a narrow range of 4–6 weeks. Finally, the Fisher’s exact test method that we applied to perform the GSEA was based on the distribution of SNPs with two a priori defined thresholds (0.05 and 0.01), thus we cannot exclude that the use of different p thresholds would have provided higher statistical power. On the other hand, this method avoided the restriction of the focus on the “top” findings of the pathway/gene and it provided an internal term of comparison in the real data (the random pathway) to test the null hypothesis.

## Conclusions

The present study replicated the association of several genes and the inorganic cation transmembrane transporter activity pathway (GO:0022890) with antidepressant efficacy in a Korean GWAS and the STAR*D. Among promising genes, we reported *CACNA1A*, *CACNB2*, *CACNA1C*, *CACNB2*, *CTNNA3* and other genes involved in cell adhesion, migration, survival and proliferation (*AGBL1*, *HS6ST3*, *RGS22*, *CYB5A*). Given the small size of the Korean sample, these findings should be considered cautiously and independent replication is required.

### Availability of data and materials

Raw dataset from the Korean sample cannot be shared since the informed consents from the participants at the point of study recruitment included study results publication but not sharing them in public. STAR*D data are available from NIMH genetics (https://www.nimhgenetics.org/) via submission of a research project.

### Ethics

All the patients were informed in detail about the aims and the procedures of the study and they signed a written informed consent prior to participation in the study. The protocol was approved by the local ethical committee (Bucheon St. Mary’s Hospital, Clinical Research Coordinator Center; approval number HC10TISI0031).

### Consent to publish

Not applicable.

## Additional files

Additional file 1: Table S1. Clinical-demographic characteristics of the Korean sample and the STAR*D level 1 sample. Mean ± SD or variable distribution were reported as appropriate. (DOC 33 kb) Additional file 2: Figure S1. QQ plots referred to remission (A) and response (B) in the Korean sample. (DOC 179 kb) Additional file 3: Figure S2. Graphic representation of enriched pathways for remission (A) and response (B). (DOC 510 kb) Additional file 4: Table S2. Characteristics of the pathways obtained by functional enrichment analysis when considering the response phenotype. (DOC 31 kb) Additional file 5: Table S3. Characteristics of the pathways obtained by functional enrichment analysis when considering the remission phenotype. (DOC 33 kb) Additional file 6: Table S4. SNPs that showed *p*<0.05 (remission phenotype) in the Korean sample and are available in the STAR*D. (DOC 1622 kb) Additional file 7: Table S5. Description and statistics referred to the SNPs that showed *p*<0.05 (remission phenotype) in both the analyzed samples. (DOC 155 kb) Additional file 8: Table S6. SNPs that showed *p*<0.05 (response phenotype) in the Korean sample and are available in the STAR*D. (DOC 1542 kb) Additional file 9: Table S7. Description and statistics referred to the SNPs that showed *p*<0.05 (response phenotype) in both the analyzed samples. (DOC 157 kb) Additional file 10: Figure S3. Manhattan plots for remission (A) and response (B) in the Korean sample. (DOC 2 kb) Additional file 11: Table S8. Top SNPs in the Korean sample (*p*<10e-05) for the response (A) and remission (B) phenotypes. When a SNP is localized in an intergenic region, the nearest gene is reported with distance in parenthesis. MAF=minor allele frequency. Chr=chromosome. (DOCX 17 kb)

## Abbreviations

GSEA: gene set enrichment analysis
GWAS: genome-wide association studies
HRSD: Hamilton Rating Scale for Depression
IBD: identity-by-descent
MAF: minor allele frequency
MDD: major depressive disorder
QIDS-C: Quick Inventory of Depressive Symptomatology-Clinician Rated
SNP: single nucleotide polymorphism
STAR*D: Sequenced Treatment Alternatives to Relieve Depression
