# Supplementary material for: Genome-wide association study of antidepressant response: involvement of the inorganic cation transmembrane transporter activity pathway
Source: BMC Psychiatry. 2016 Apr 18;16:106. doi: 10.1186/s12888-016-0813-x (PMC4836090; doi:10.1186/s12888-016-0813-x)
Supplement: Additional file 1: Table S1. — Clinical-demographic characteristics of the Korean sample and the STAR*D level 1 sample. Mean ± SD or variable distribution were reported as appropriate. (DOC 33 kb) [file 12888_2016_813_MOESM1_ESM.doc]

**Table S1**: clinical-demographic characteristics of the Korean sample and the STAR*D level 1 sample. Mean ± SD or variable distribution were reported as appropriate.

| **Variable** | **Korean sample (n=109)** | **STAR*D Level 1 (n=1677)** |
| --- | --- | --- |
| Gender (F/M) | 70/39 | 1046/631 |
| Age | 43.99±15.03 | 42.67 ±13.11 |
| Ethnicity (%) | Korean (100%) | White non hispanic (68%), White hispanic (13%), African-American (17%), Asian (2%) |
| Age at onset | 39.37±13.71 | 25.33 ±14.66 |
| Baseline severity (HDRS17) | 21.89±7.83 | 23.36±5.03 |
| Response (%) | 52 (47.71%) | 840 (50.09%) |
| Remission (%) | 42 (38.53%) | 575 (34.29%) |
| Antidepressant treatment | Paroxetine (n=83) or venlafaxine (n=26) | Citalopram |
| Mean final antidepressant dose (mg) | Paroxetine: 20.69±10.18  Venlafaxine: 152.88±23.53 | 20.37±4.70 |
