# Supplementary figures and images for: Genome-wide association study of antidepressant response: involvement of the inorganic cation transmembrane transporter activity pathway
Source: BMC Psychiatry. 2016 Apr 18;16:106. doi: 10.1186/s12888-016-0813-x (PMC4836090; doi:10.1186/s12888-016-0813-x)

**Figure S1**: QQ plots referred to remission (**A**) and response (**B**) in the Korean sample.

| **A** | **B** |
| --- | --- |
| 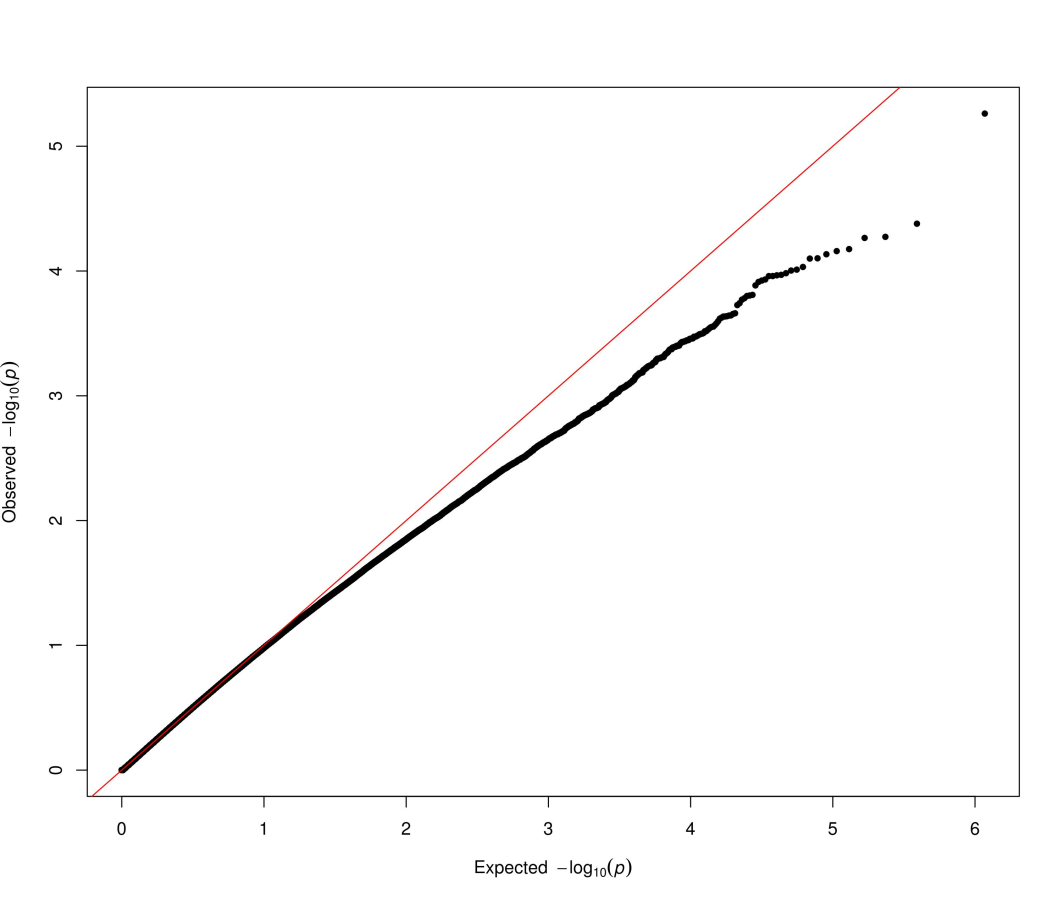 | 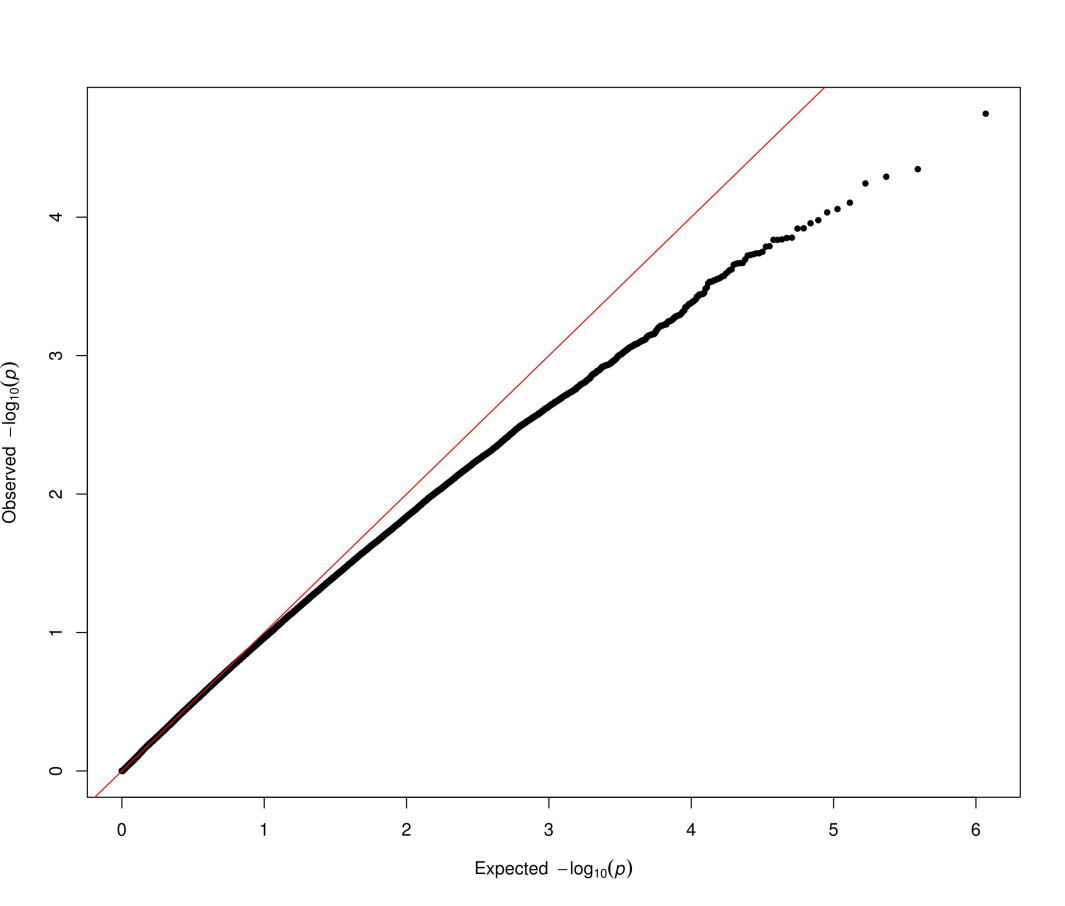 |

Supplement: Additional file 2: Figure S1. — QQ plots referred to remission (A) and response (B) in the Korean sample. (DOC 179 kb) [file 12888_2016_813_MOESM2_ESM.doc]

**Figure S2**: Graphic representation of enriched pathways for remission (**A**) and response (**B**).

**A**


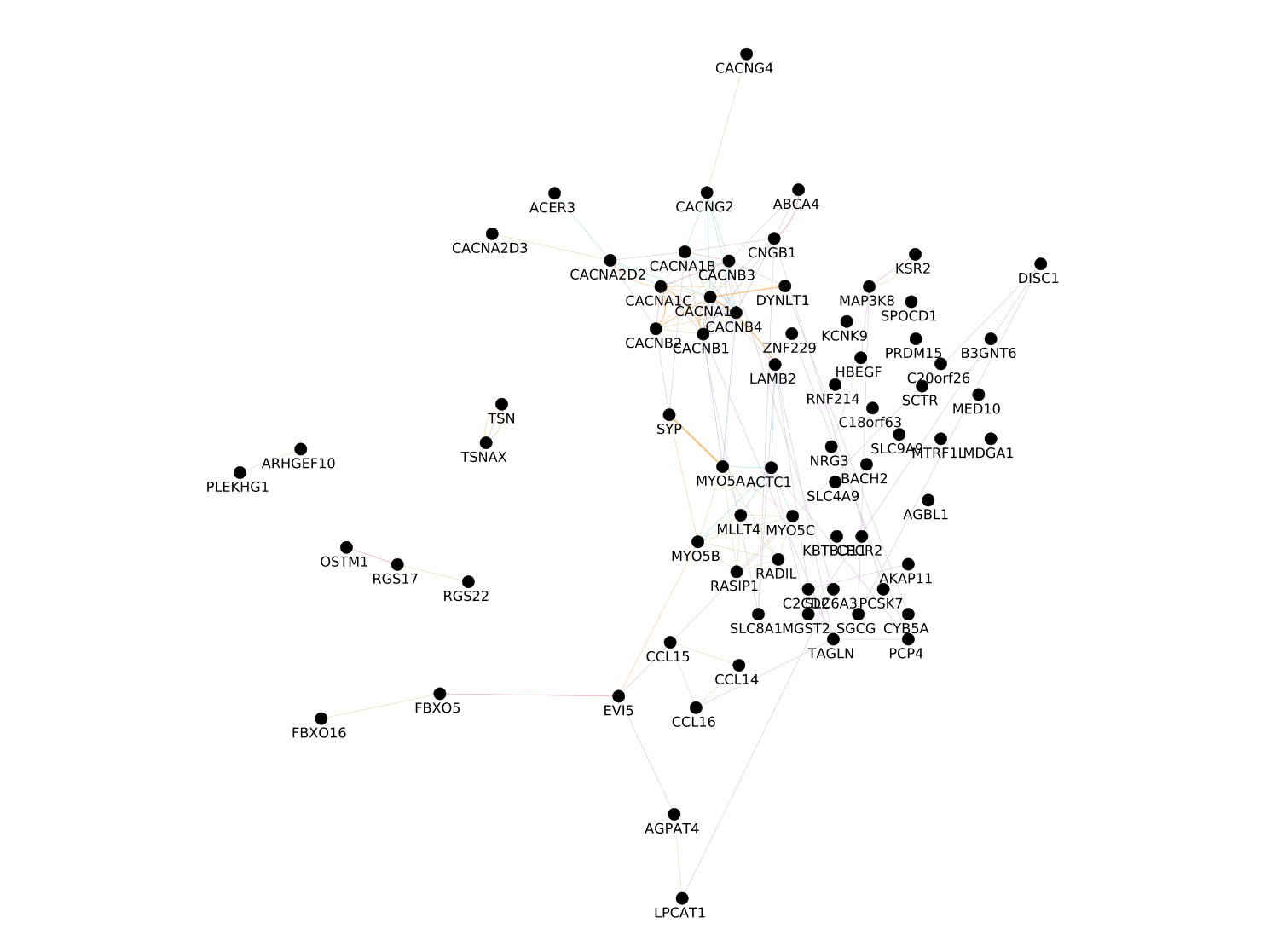


**B**


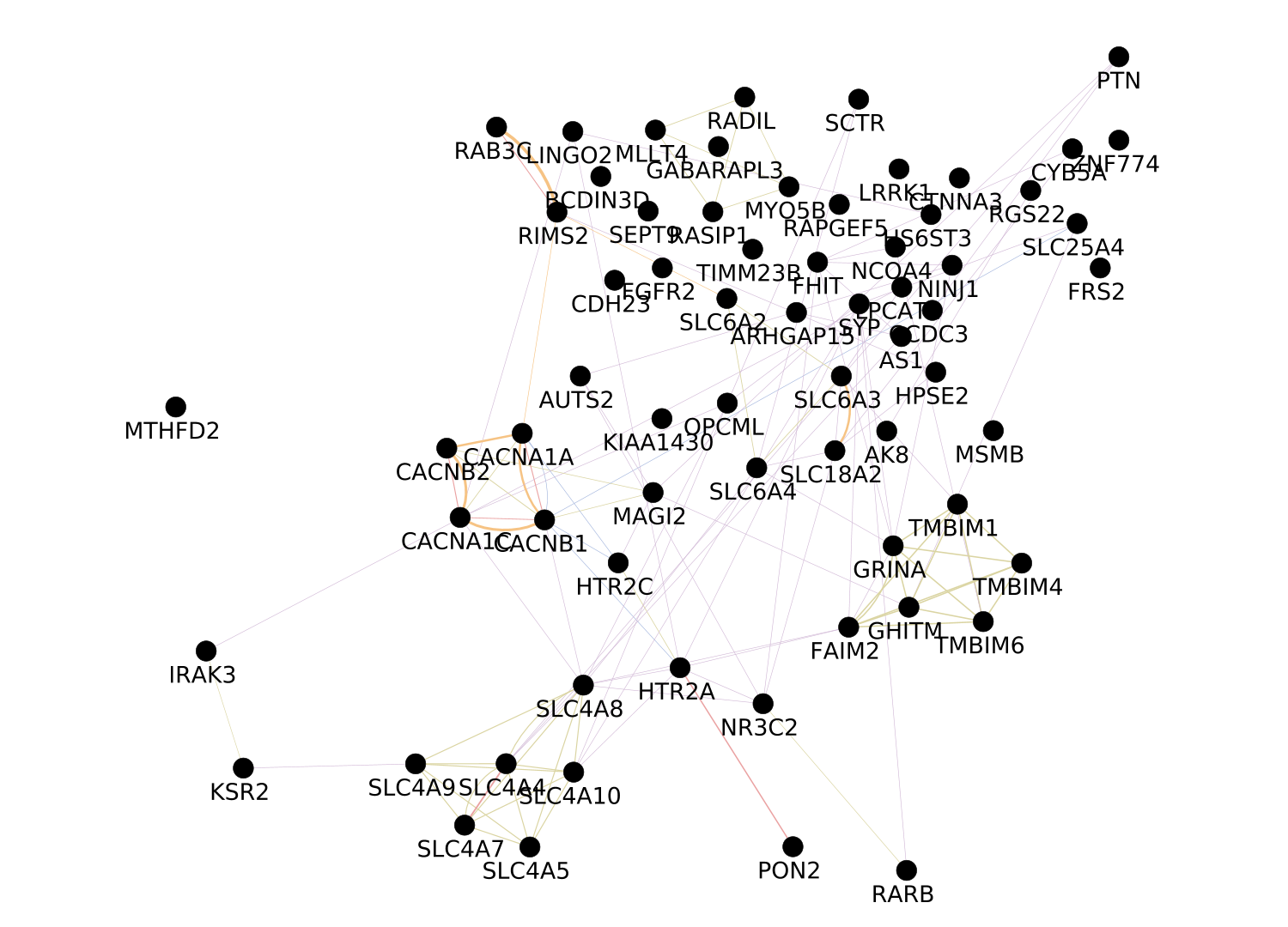

Supplement: Additional file 3: Figure S2. — Graphic representation of enriched pathways for remission (A) and response (B). (DOC 510 kb) [file 12888_2016_813_MOESM3_ESM.doc]

**Supplementary Figure 3**: Manhattan plots for remission (A) and response (B) in the Korean sample.

| **A** | **B** |
| --- | --- |
| 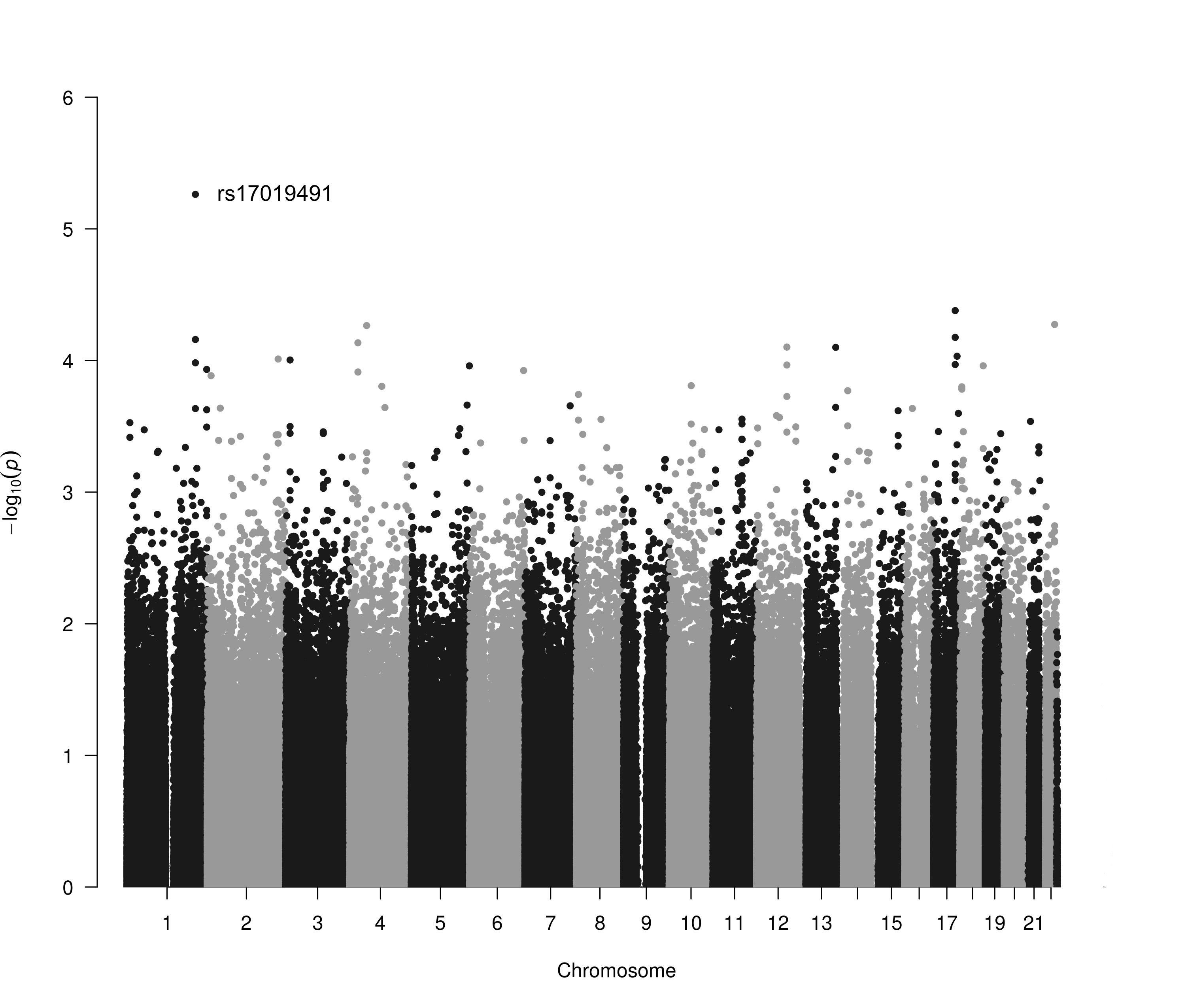 | 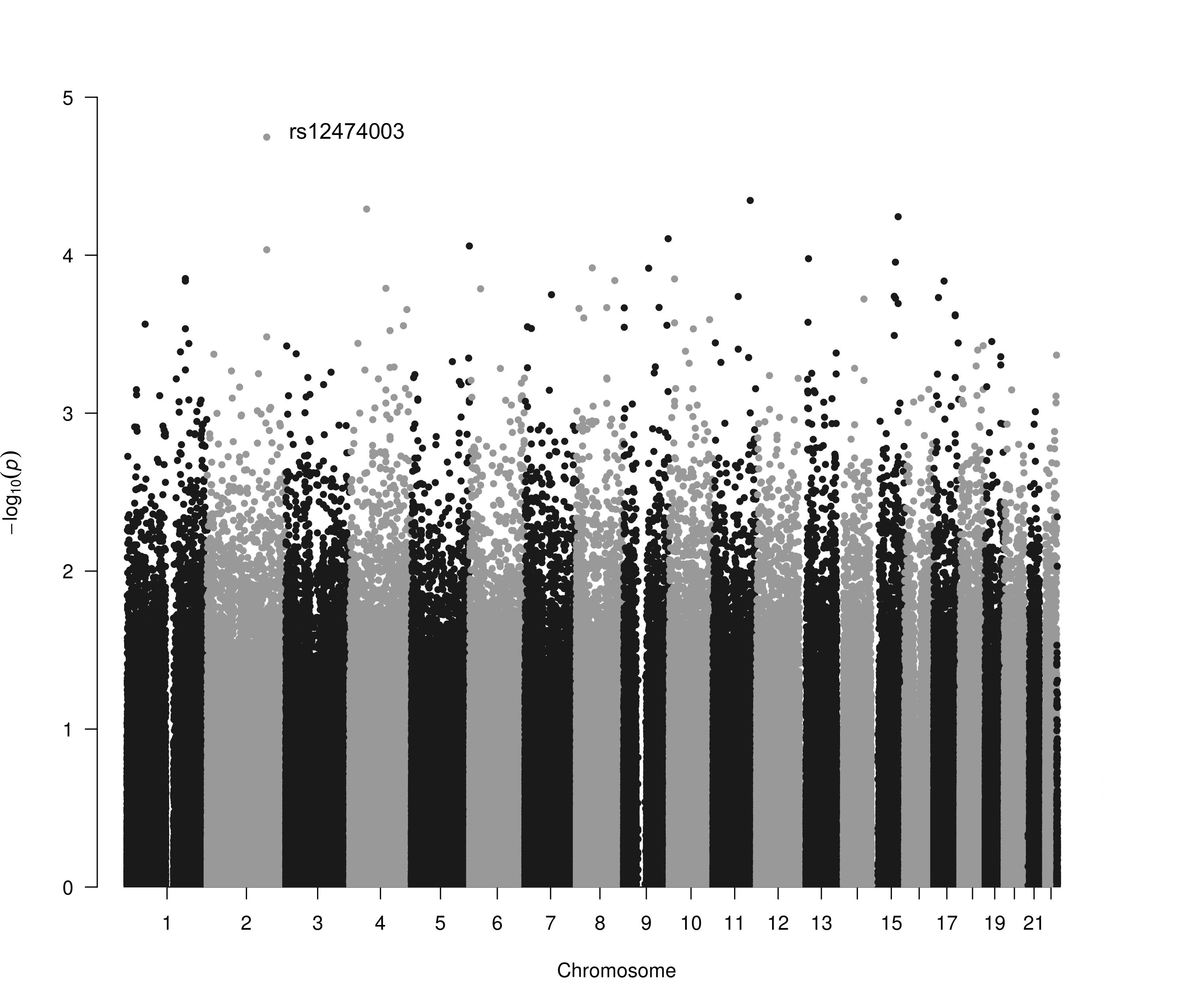 |

Supplement: Additional file 10: Figure S3. — Manhattan plots for remission (A) and response (B) in the Korean sample. (DOC 2 kb) [file 12888_2016_813_MOESM10_ESM.doc]
