# Supplementary material for: Genome-wide association study of antidepressant response: involvement of the inorganic cation transmembrane transporter activity pathway
Source: BMC Psychiatry. 2016 Apr 18;16:106. doi: 10.1186/s12888-016-0813-x (PMC4836090; doi:10.1186/s12888-016-0813-x)
Supplement: Additional file 4: Table S2. — Characteristics of the pathways obtained by functional enrichment analysis when considering the response phenotype. (DOC 31 kb) [file 12888_2016_813_MOESM4_ESM.doc]

**Table S2**: characteristics of the pathways obtained by functional enrichment analysis when considering the response phenotype.

| **gene ontology ID** | **function** | **q-value** | **Genes in the pathway** |
| --- | --- | --- | --- |
| GO:0005245 | voltage-gated calcium channel activity | 0.01 | CACNA1A, CACNA1C, CACNB1, CACNB2 |
| GO:0015081 | sodium ion transmembrane transporter activity | 0.01 | SLC4A4, SLC4A5, SLC4A7, SLC6A2, SLC6A4 |
| GO:0015701 | bicarbonate transport | 5.3e-5 | SLC4A10, SLC4A4, SLC4A5, SLC4A7, SLC4A8, SLC4A9 |
| GO:0015844 | monoamine transport | 0.01 | SLC18A2, SLC6A2, SLC6A3, SLC6A4 |
| GO:0022804 | active transmembrane transporter activity | 0.003 | SLC18A2, SLC4A4, SLC4A5, SLC4A7, SLC6A2, SLC6A3, SLC6A4 |
| GO:0022890 | inorganic cation transmembrane transporter activity | 0.002 | CACNA1A, CACNA1C, CACNB1, CACNB2, CYB5A, SLC4A4, SLC4A5, SLC4A7, SLC6A2, SLC6A4 |
| GO:0046873 | metal ion transmembrane transporter activity | 0.003 | CACNA1A, CACNA1C, CACNB1, CACNB2, SLC4A4, SLC4A5, SLC4A7, SLC6A2, SLC6A4 |
