# Supplementary material for: Genome-wide association study of antidepressant response: involvement of the inorganic cation transmembrane transporter activity pathway
Source: BMC Psychiatry. 2016 Apr 18;16:106. doi: 10.1186/s12888-016-0813-x (PMC4836090; doi:10.1186/s12888-016-0813-x)
Supplement: Additional file 5: Table S3. — Characteristics of the pathways obtained by functional enrichment analysis when considering the remission phenotype. (DOC 33 kb) [file 12888_2016_813_MOESM5_ESM.doc]

**Table S3**: characteristics of the pathways obtained by functional enrichment analysis when considering the remission phenotype.

| **gene ontology ID** | **function** | **q-value** | **Genes in the pathway** |
| --- | --- | --- | --- |
| GO:0005216 | ion channel activity | 0.004 | CACNA1A, CACNA1C, CACNB1, CACNB2, CACNB3, CACNB4, CNGB1 |
| GO:0005244 | voltage-gated ion channel activity | 7.5e-4 | CACNA1A, CACNA1C, CACNB1, CACNB2, CACNB3, CACNB4 |
| GO:0005891 | voltage-gated calcium channel complex | 7.5e-4 | CACNA1C, CACNB2, CACNB3, CACNB4 |
| GO:0006874 | cellular calcium ion homeostasis | 0.07 | CACNA1A, CACNA1C, CCL14, CCL15, SLC8A1 |
| GO:0015085 | calcium ion transmembrane transporter activity | 8e-6 | CACNA1A, CACNA1C, CACNB1, CACNB2, CACNB3, CACNB4, SLC8A1 |
| GO:0022890 | inorganic cation transmembrane transporter activity | 7.5e-4 | CACNA1A, CACNA1C, CACNB1, CACNB2, CACNB3, CACNB4, CYB5A, SLC8A1 |
| GO:0034703 | cation channel complex | 0.003 | CACNA1C, CACNB2, CACNB3, CACNB4, CNGB1 |
| GO:0042391 | regulation of membrane potential | 3.3e-4 | ARHGEF10, CACNA1A, CACNA1B, CACNB3, CACNB4, CACNG2, CACNG4 |
| GO:0043269 | regulation of ion transport | 0.09 | CACNA1C, CACNB2, CACNG2, CACNG4, SLC8A1 |
| GO:0051899 | membrane depolarization | 2.1e-5 | CACNA1A, CACNA1B, CACNB3, CACNB4, CACNG2, CACNG4 |
