# Supplementary material for: Genome-wide association study of antidepressant response: involvement of the inorganic cation transmembrane transporter activity pathway
Source: BMC Psychiatry. 2016 Apr 18;16:106. doi: 10.1186/s12888-016-0813-x (PMC4836090; doi:10.1186/s12888-016-0813-x)
Supplement: Additional file 6: Table S4. — SNPs that showed p<0.05 (remission phenotype) in the Korean sample and are available in the STAR*D. (DOC 1622 kb) [file 12888_2016_813_MOESM6_ESM.doc]

**Table S4**: SNPs that showed p<0.05 (remission phenotype) in the Korean sample and are available in the STAR*D.

| **chromosome** | **SNP** | **position** | **test** | **non missing** | **odds ratio** | **stat** | **p** |
| --- | --- | --- | --- | --- | --- | --- | --- |
| 1 | rs2842909 | 2849584 | ADD | 1617 | 1.003 | 0.03252 | 0.9741 |
| 1 | rs1798246 | 3070715 | ADD | 1638 | 0.9816 | -0.2569 | 0.7973 |
| 1 | rs4129100 | 3978308 | ADD | 1636 | 1.16 | 2.155 | 0.03114 |
| 1 | rs707458 | 7754799 | ADD | 1637 | 1.107 | 1.407 | 0.1596 |
| 1 | rs10927440 | 14663429 | ADD | 1631 | 0.9452 | -0.6696 | 0.5031 |
| 1 | rs2293914 | 17422901 | ADD | 1633 | 1.101 | 0.9022 | 0.3669 |
| 1 | rs560514 | 18005188 | ADD | 1619 | 0.9452 | -0.8191 | 0.4127 |
| 1 | rs16861827 | 18550757 | ADD | 1637 | 1.056 | 0.4863 | 0.6268 |
| 1 | rs7411058 | 19006847 | ADD | 1596 | 1.034 | 0.3713 | 0.7104 |
| 1 | rs16862370 | 19114491 | ADD | 1636 | 1.137 | 1.379 | 0.1678 |
| 1 | rs3820317 | 20013168 | ADD | 1638 | 0.9441 | -0.397 | 0.6913 |
| 1 | rs12023742 | 20164593 | ADD | 1636 | 0.9515 | -0.6552 | 0.5123 |
| 1 | rs601786 | 23559385 | ADD | 1638 | 1.103 | 0.8266 | 0.4085 |
| 1 | rs1767523 | 27928929 | ADD | 1620 | 0.9865 | -0.1921 | 0.8477 |
| 1 | rs11589992 | 32072717 | ADD | 1638 | 0.8595 | -2.126 | 0.03347 |
| 1 | rs10914912 | 34566417 | ADD | 1617 | 0.966 | -0.475 | 0.6348 |
| 1 | rs3820271 | 37122320 | ADD | 1618 | 1.132 | 1.29 | 0.197 |
| 1 | rs10908337 | 37538507 | ADD | 1626 | 1.012 | 0.1677 | 0.8668 |
| 1 | rs1317557 | 41611929 | ADD | 1637 | 1.146 | 0.7637 | 0.4451 |
| 1 | rs841860 | 43139478 | ADD | 1636 | 1.108 | 1.104 | 0.2694 |
| 1 | rs10789439 | 43895778 | ADD | 1638 | 0.9114 | -1.304 | 0.1923 |
| 1 | rs868351 | 45782405 | ADD | 1623 | 0.9635 | -0.4258 | 0.6703 |
| 1 | rs6588492 | 54100613 | ADD | 1638 | 0.976 | -0.3271 | 0.7436 |
| 1 | rs904218 | 54733577 | ADD | 1625 | 0.9114 | -0.7809 | 0.4349 |
| 1 | rs10888879 | 55016178 | ADD | 1632 | 1.201 | 1.123 | 0.2613 |
| 1 | rs638944 | 55114503 | ADD | 1628 | 1.065 | 0.8727 | 0.3828 |
| 1 | rs168549 | 55771023 | ADD | 1637 | 1.175 | 1.994 | 0.04614 |
| 1 | rs10888934 | 55806480 | ADD | 1638 | 0.8856 | -1.567 | 0.1171 |
| 1 | rs857107 | 56995187 | ADD | 1634 | 1.115 | 1.336 | 0.1817 |
| 1 | rs11207409 | 59379228 | ADD | 1630 | 0.9118 | -1.262 | 0.2068 |
| 1 | rs1440575 | 59796529 | ADD | 1636 | 1.013 | 0.1902 | 0.8491 |
| 1 | rs12565296 | 61138425 | ADD | 1638 | 1.03 | 0.2659 | 0.7903 |
| 1 | rs17390595 | 63348003 | ADD | 1636 | 0.9209 | -0.9281 | 0.3534 |
| 1 | rs7541092 | 71135060 | ADD | 1638 | 1.048 | 0.3039 | 0.7612 |
| 1 | rs10789398 | 74791009 | ADD | 1635 | 1.125 | 1.609 | 0.1076 |
| 1 | rs505867 | 76915412 | ADD | 1620 | 0.9344 | -0.8689 | 0.3849 |
| 1 | rs11161574 | 85499632 | ADD | 1569 | 1.028 | 0.2962 | 0.7671 |
| 1 | rs1524004 | 85783645 | ADD | 1629 | 0.8631 | -1.857 | 0.06326 |
| 1 | rs969396 | 86545634 | ADD | 1607 | 1.161 | 1.896 | 0.05798 |
| 1 | rs2791473 | 86761297 | ADD | 1638 | 0.941 | -0.8723 | 0.383 |
| 1 | rs4147849 | 94253358 | ADD | 1638 | 0.8263 | -1.224 | 0.2209 |
| 1 | rs4147839 | 94281814 | ADD | 1630 | 0.8453 | -2.215 | 0.02676 |
| 1 | rs10874867 | 94856424 | ADD | 1634 | 1.112 | 1.338 | 0.1808 |
| 1 | rs2766005 | 95201002 | ADD | 1617 | 0.9646 | -0.5041 | 0.6142 |
| 1 | rs6694095 | 102057631 | ADD | 1633 | 1.008 | 0.08756 | 0.9302 |
| 1 | rs7532749 | 105021286 | ADD | 1614 | 1.109 | 1.236 | 0.2166 |
| 1 | rs10494090 | 108494859 | ADD | 1638 | 0.8951 | -1.515 | 0.1297 |
| 1 | rs10494092 | 108935362 | ADD | 1574 | 1.038 | 0.448 | 0.6542 |
| 1 | rs641227 | 110841578 | ADD | 1638 | 0.9257 | -1.037 | 0.2996 |
| 1 | rs3818729 | 112793385 | ADD | 1594 | 0.9243 | -0.9235 | 0.3558 |
| 1 | rs4378202 | 113132657 | ADD | 1621 | 0.8925 | -1.546 | 0.122 |
| 1 | rs662353 | 114687164 | ADD | 1638 | 0.8958 | -1.392 | 0.1639 |
| 1 | rs688325 | 145543907 | ADD | 1618 | 0.9002 | -1.136 | 0.2561 |
| 1 | rs2054855 | 152637562 | ADD | 1637 | 1.037 | 0.3746 | 0.708 |
| 1 | rs10908787 | 158814871 | ADD | 1592 | 0.9637 | -0.3629 | 0.7167 |
| 1 | rs4657219 | 160906398 | ADD | 1634 | 1.054 | 0.6143 | 0.539 |
| 1 | rs16843630 | 160936667 | ADD | 1635 | 0.9519 | -0.4719 | 0.637 |
| 1 | rs2684879 | 161069239 | ADD | 1637 | 1.043 | 0.5307 | 0.5956 |
| 1 | rs2419114 | 166804269 | ADD | 1634 | 0.9722 | -0.3874 | 0.6985 |
| 1 | rs2223286 | 167932256 | ADD | 1637 | 0.9901 | -0.1339 | 0.8935 |
| 1 | rs4987313 | 167938984 | ADD | 1603 | 0.7853 | -0.8491 | 0.3958 |
| 1 | rs10800485 | 168113770 | ADD | 1638 | 1.02 | 0.2546 | 0.799 |
| 1 | rs12042109 | 168352077 | ADD | 1638 | 0.9466 | -0.743 | 0.4575 |
| 1 | rs983118 | 168473526 | ADD | 1636 | 0.9674 | -0.4653 | 0.6417 |
| 1 | rs12122745 | 168477099 | ADD | 1607 | 0.9675 | -0.4298 | 0.6673 |
| 1 | rs10489318 | 173593770 | ADD | 1633 | 0.9736 | -0.3421 | 0.7323 |
| 1 | rs7544659 | 173913664 | ADD | 1638 | 1.025 | 0.3355 | 0.7372 |
| 1 | rs7552857 | 174695987 | ADD | 1638 | 0.8736 | -1.226 | 0.2201 |
| 1 | rs12083856 | 177304778 | ADD | 1638 | 0.8643 | -1.088 | 0.2764 |
| 1 | rs1570807 | 177327731 | ADD | 1634 | 0.9608 | -0.5141 | 0.6072 |
| 1 | rs10753199 | 177826255 | ADD | 1635 | 0.937 | -0.9371 | 0.3487 |
| 1 | rs10157919 | 179438391 | ADD | 1563 | 1.137 | 1.132 | 0.2575 |
| 1 | rs175337 | 179877910 | ADD | 1616 | 0.9718 | -0.3154 | 0.7525 |
| 1 | rs546191 | 180019653 | ADD | 1636 | 1.066 | 0.8185 | 0.4131 |
| 1 | rs9286844 | 180094186 | ADD | 1637 | 1.062 | 0.8068 | 0.4198 |
| 1 | rs630341 | 180094612 | ADD | 1603 | 1.137 | 1.773 | 0.07627 |
| 1 | rs12566065 | 188003456 | ADD | 1602 | 1.048 | 0.6455 | 0.5186 |
| 1 | rs12138550 | 196132980 | ADD | 1638 | 0.8864 | -1.49 | 0.1361 |
| 1 | rs10494795 | 197753444 | ADD | 1638 | 1.035 | 0.3066 | 0.7591 |
| 1 | rs1060061 | 198410065 | ADD | 1638 | 0.9204 | -1.162 | 0.2453 |
| 1 | rs6427830 | 198731426 | ADD | 1637 | 0.8829 | -1.602 | 0.1092 |
| 1 | rs2250538 | 202348515 | ADD | 1638 | 0.9727 | -0.3696 | 0.7117 |
| 1 | rs6693954 | 202399261 | ADD | 1598 | 1.005 | 0.06312 | 0.9497 |
| 1 | rs2842754 | 205323926 | ADD | 1638 | 1.041 | 0.4984 | 0.6182 |
| 1 | rs12747035 | 212785969 | ADD | 1638 | 1.026 | 0.3582 | 0.7202 |
| 1 | rs12750128 | 213139727 | ADD | 1630 | 1.066 | 0.7442 | 0.4568 |
| 1 | rs2797222 | 213982001 | ADD | 1635 | 0.9602 | -0.5525 | 0.5806 |
| 1 | rs1416526 | 215303518 | ADD | 1637 | 0.9235 | -0.7959 | 0.4261 |
| 1 | rs7555255 | 215417006 | ADD | 1635 | 1.072 | 0.7179 | 0.4728 |
| 1 | rs17047306 | 216310320 | ADD | 1637 | 1.12 | 0.8416 | 0.4 |
| 1 | rs2046842 | 216320435 | ADD | 1633 | 1.057 | 0.5714 | 0.5678 |
| 1 | rs2808026 | 218399499 | ADD | 1638 | 1.031 | 0.4229 | 0.6723 |
| 1 | rs17009664 | 219464373 | ADD | 1636 | 0.9754 | -0.1752 | 0.861 |
| 1 | rs6661550 | 225159485 | ADD | 1637 | 0.9529 | -0.6016 | 0.5475 |
| 1 | rs710824 | 227143381 | ADD | 1636 | 0.934 | -0.9306 | 0.3521 |
| 1 | rs687723 | 227957188 | ADD | 1617 | 1.008 | 0.1128 | 0.9102 |
| 1 | rs11122458 | 228375478 | ADD | 1587 | 1.007 | 0.07841 | 0.9375 |
| 1 | rs10864669 | 229320626 | ADD | 1589 | 1.115 | 1.485 | 0.1375 |
| 1 | rs7365975 | 230422758 | ADD | 1635 | 0.9278 | -0.9704 | 0.3319 |
| 1 | rs1805087 | 235115123 | ADD | 1638 | 0.924 | -0.87 | 0.3843 |
| 1 | rs560229 | 236792385 | ADD | 1620 | 1.092 | 0.813 | 0.4162 |
| 1 | rs12096679 | 238678237 | ADD | 1637 | 1.02 | 0.2658 | 0.7904 |
| 1 | rs12069867 | 239115345 | ADD | 1636 | 1.003 | 0.03481 | 0.9722 |
| 1 | rs10465632 | 239386990 | ADD | 1629 | 0.9789 | -0.3075 | 0.7585 |
| 1 | rs10926423 | 239405181 | ADD | 1635 | 0.891 | -1.092 | 0.2749 |
| 1 | rs4278360 | 241119680 | ADD | 1591 | 0.819 | -2.122 | 0.03381 |
| 1 | rs4658558 | 241572464 | ADD | 1634 | 1.054 | 0.74 | 0.4593 |
| 1 | rs4658649 | 242921444 | ADD | 1638 | 1.012 | 0.1476 | 0.8827 |
| 1 | rs9793102 | 245389883 | ADD | 1631 | 1.058 | 0.766 | 0.4436 |
| 1 | rs10924936 | 245406391 | ADD | 1633 | 1.093 | 1.303 | 0.1925 |
| 2 | rs2564010 | 5906090 | ADD | 1581 | 1.118 | 1.52 | 0.1285 |
| 2 | rs2351104 | 6283601 | ADD | 1635 | 0.9754 | -0.3373 | 0.7359 |
| 2 | rs10929507 | 7956645 | ADD | 1624 | 1.07 | 0.983 | 0.3256 |
| 2 | rs4669613 | 10791963 | ADD | 1635 | 0.9783 | -0.2677 | 0.7889 |
| 2 | rs2716628 | 11933214 | ADD | 1609 | 1.014 | 0.183 | 0.8548 |
| 2 | rs2675888 | 14599225 | ADD | 1624 | 1.114 | 1.447 | 0.1479 |
| 2 | rs11886084 | 18069894 | ADD | 1630 | 0.9663 | -0.4801 | 0.6311 |
| 2 | rs1204005 | 19850921 | ADD | 1636 | 1.044 | 0.4243 | 0.6714 |
| 2 | rs6744750 | 20930541 | ADD | 1629 | 1.109 | 1.353 | 0.1762 |
| 2 | rs875077 | 23438070 | ADD | 1629 | 0.9829 | -0.2299 | 0.8182 |
| 2 | rs4665665 | 24234219 | ADD | 1624 | 1.272 | 2.228 | 0.02585 |
| 2 | rs12998454 | 29808156 | ADD | 1633 | 0.9076 | -0.8372 | 0.4025 |
| 2 | rs6543592 | 31093750 | ADD | 1629 | 1.178 | 1.401 | 0.1611 |
| 2 | rs4670110 | 35269716 | ADD | 1632 | 1.074 | 0.924 | 0.3555 |
| 2 | rs4670551 | 36467811 | ADD | 1626 | 1.096 | 1.253 | 0.2101 |
| 2 | rs11901530 | 39964883 | ADD | 1624 | 1.051 | 0.7082 | 0.4788 |
| 2 | rs4952404 | 40414134 | ADD | 1638 | 0.8245 | -2.221 | 0.02635 |
| 2 | rs4952521 | 41943543 | ADD | 1637 | 1.019 | 0.2678 | 0.7889 |
| 2 | rs7569588 | 45273586 | ADD | 1638 | 0.9624 | -0.5231 | 0.6009 |
| 2 | rs1868271 | 46170852 | ADD | 1634 | 1.057 | 0.7564 | 0.4494 |
| 2 | rs4971869 | 49701792 | ADD | 1558 | 0.9521 | -0.6144 | 0.5389 |
| 2 | rs6731061 | 50096906 | ADD | 1622 | 0.9654 | -0.4876 | 0.6258 |
| 2 | rs350753 | 52727292 | ADD | 1629 | 0.9672 | -0.4685 | 0.6394 |
| 2 | rs7559322 | 52877554 | ADD | 1631 | 0.8853 | -1.705 | 0.08825 |
| 2 | rs848286 | 58248047 | ADD | 1574 | 0.9016 | -1.391 | 0.1643 |
| 2 | rs10205398 | 62533657 | ADD | 1565 | 0.9139 | -0.9197 | 0.3578 |
| 2 | rs17029079 | 64589576 | ADD | 1576 | 0.7365 | -1.177 | 0.2393 |
| 2 | rs6733507 | 64789976 | ADD | 1638 | 1.063 | 0.7382 | 0.4604 |
| 2 | rs6760401 | 64801882 | ADD | 1599 | 1.161 | 1.166 | 0.2436 |
| 2 | rs2241241 | 65822288 | ADD | 1637 | 0.9781 | -0.1462 | 0.8838 |
| 2 | rs7572685 | 67723729 | ADD | 1638 | 1.061 | 0.5055 | 0.6132 |
| 2 | rs1075454 | 68195947 | ADD | 1637 | 0.8169 | -1.389 | 0.1649 |
| 2 | rs744636 | 68196534 | ADD | 1636 | 0.9471 | -0.7293 | 0.4658 |
| 2 | rs1529409 | 71684197 | ADD | 1638 | 1.015 | 0.1226 | 0.9024 |
| 2 | rs10199560 | 74296559 | ADD | 1635 | 1.128 | 0.8059 | 0.4203 |
| 2 | rs3771766 | 74949317 | ADD | 1638 | 1.151 | 0.8197 | 0.4124 |
| 2 | rs12989728 | 74992350 | ADD | 1631 | 0.8976 | -1.516 | 0.1296 |
| 2 | rs6711618 | 81339645 | ADD | 1635 | 0.8822 | -1.648 | 0.09935 |
| 2 | rs7589293 | 81437068 | ADD | 1595 | 1.007 | 0.08964 | 0.9286 |
| 2 | rs2438307 | 85978810 | ADD | 1637 | 0.932 | -0.6393 | 0.5226 |
| 2 | rs1659248 | 88416088 | ADD | 1638 | 1.049 | 0.3487 | 0.7273 |
| 2 | rs4850905 | 99518797 | ADD | 1638 | 1.13 | 1.376 | 0.1688 |
| 2 | rs12467316 | 101971081 | ADD | 1637 | 0.8778 | -1.664 | 0.09611 |
| 2 | rs17020852 | 104717323 | ADD | 1638 | 0.8954 | -1.426 | 0.1539 |
| 2 | rs13422730 | 105151304 | ADD | 1638 | 0.9963 | -0.04944 | 0.9606 |
| 2 | rs6543317 | 105526930 | ADD | 1621 | 0.9495 | -0.6538 | 0.5132 |
| 2 | rs6723920 | 105529956 | ADD | 1625 | 1.115 | 1.464 | 0.1432 |
| 2 | rs1037381 | 105669675 | ADD | 1605 | 1.06 | 0.7777 | 0.4368 |
| 2 | rs6746088 | 107353467 | ADD | 1635 | 1.175 | 2.294 | 0.02179 |
| 2 | rs13384147 | 109805518 | ADD | 1637 | 1.02 | 0.189 | 0.8501 |
| 2 | rs6757604 | 119961173 | ADD | 1592 | 1.154 | 1.985 | 0.04714 |
| 2 | rs2579604 | 119981320 | ADD | 1570 | 0.9943 | -0.07467 | 0.9405 |
| 2 | rs2920692 | 119982320 | ADD | 1629 | 0.7391 | -1.692 | 0.0907 |
| 2 | rs17364261 | 122850905 | ADD | 1635 | 0.6576 | -1.503 | 0.1328 |
| 2 | rs1453678 | 122857073 | ADD | 1638 | 0.9264 | -1.07 | 0.2845 |
| 2 | rs360234 | 126758025 | ADD | 1638 | 1.055 | 0.6514 | 0.5148 |
| 2 | rs7606197 | 126957270 | ADD | 1636 | 1.126 | 1.476 | 0.14 |
| 2 | rs2246289 | 129417087 | ADD | 1638 | 0.9842 | -0.2189 | 0.8267 |
| 2 | rs12466662 | 131490656 | ADD | 1638 | 0.9868 | -0.1479 | 0.8824 |
| 2 | rs12691834 | 133668510 | ADD | 1595 | 0.9953 | -0.05911 | 0.9529 |
| 2 | rs1519305 | 134530256 | ADD | 1633 | 0.9065 | -1.354 | 0.1756 |
| 2 | rs2322659 | 136272129 | ADD | 1629 | 1.04 | 0.5426 | 0.5874 |
| 2 | rs7561228 | 143982974 | ADD | 1589 | 1.098 | 1.106 | 0.2687 |
| 2 | rs697651 | 155031524 | ADD | 1622 | 1.001 | 0.01153 | 0.9908 |
| 2 | rs747282 | 158257988 | ADD | 1610 | 0.959 | -0.5388 | 0.59 |
| 2 | rs264626 | 159656347 | ADD | 1560 | 0.9918 | -0.06903 | 0.945 |
| 2 | rs6718526 | 160922421 | ADD | 1636 | 1.098 | 0.9797 | 0.3273 |
| 2 | rs2288331 | 170300955 | ADD | 1633 | 0.9327 | -0.7301 | 0.4653 |
| 2 | rs16858054 | 170814034 | ADD | 1635 | 1.02 | 0.1348 | 0.8928 |
| 2 | rs10174809 | 173094780 | ADD | 1638 | 0.981 | -0.2276 | 0.82 |
| 2 | rs12622834 | 173246793 | ADD | 1630 | 0.9189 | -1.023 | 0.3064 |
| 2 | rs7575189 | 173723414 | ADD | 1637 | 1.026 | 0.3697 | 0.7116 |
| 2 | rs16861605 | 173961556 | ADD | 1638 | 0.8878 | -1.268 | 0.2048 |
| 2 | rs515309 | 174180931 | ADD | 1638 | 1.152 | 1.369 | 0.1709 |
| 2 | rs1434091 | 178940806 | ADD | 1637 | 0.9165 | -0.9119 | 0.3618 |
| 2 | rs10497546 | 180228875 | ADD | 1638 | 1.056 | 0.4856 | 0.6273 |
| 2 | rs7559692 | 197805197 | ADD | 1635 | 0.9476 | -0.617 | 0.5372 |
| 2 | rs166849 | 200487111 | ADD | 1622 | 0.9915 | -0.1173 | 0.9066 |
| 2 | rs868810 | 201224184 | ADD | 1620 | 0.9966 | -0.02877 | 0.977 |
| 2 | rs13402805 | 202256209 | ADD | 1635 | 0.9392 | -0.6804 | 0.4963 |
| 2 | rs4321346 | 202560461 | ADD | 1637 | 1.027 | 0.3312 | 0.7405 |
| 2 | rs4335928 | 204510225 | ADD | 1634 | 1.06 | 0.4958 | 0.62 |
| 2 | rs7606468 | 205853078 | ADD | 1637 | 0.9306 | -0.9268 | 0.354 |
| 2 | rs3770536 | 216547065 | ADD | 1631 | 1.063 | 0.8502 | 0.3952 |
| 2 | rs16855663 | 216780472 | ADD | 1637 | 0.9835 | -0.1046 | 0.9167 |
| 2 | rs768921 | 218372224 | ADD | 1594 | 0.9252 | -0.5288 | 0.597 |
| 2 | rs2385199 | 219458990 | ADD | 1636 | 1.012 | 0.1449 | 0.8848 |
| 2 | rs16860351 | 220398375 | ADD | 1631 | 1.04 | 0.4386 | 0.6609 |
| 2 | rs6739216 | 223259391 | ADD | 1638 | 0.9673 | -0.3338 | 0.7386 |
| 2 | rs1821409 | 225404645 | ADD | 1635 | 1.029 | 0.3742 | 0.7082 |
| 2 | rs1501002 | 227102120 | ADD | 1637 | 1.025 | 0.3472 | 0.7284 |
| 2 | rs6726046 | 233951960 | ADD | 1627 | 1.117 | 1.529 | 0.1262 |
| 2 | rs4277473 | 235675961 | ADD | 1638 | 0.9927 | -0.0971 | 0.9226 |
| 2 | rs13392175 | 237101143 | ADD | 1638 | 0.9274 | -0.6085 | 0.5429 |
| 2 | rs6431547 | 238157857 | ADD | 1634 | 0.9562 | -0.6163 | 0.5377 |
| 2 | rs4676431 | 241105074 | ADD | 1628 | 0.8892 | -1.165 | 0.244 |
| 3 | rs428179 | 1585284 | ADD | 1632 | 0.9155 | -1.119 | 0.263 |
| 3 | rs1317443 | 1685731 | ADD | 1637 | 1.009 | 0.1023 | 0.9185 |
| 3 | rs6774407 | 3648983 | ADD | 1636 | 1.125 | 1.111 | 0.2666 |
| 3 | rs342003 | 5282598 | ADD | 1638 | 1.089 | 0.9719 | 0.3311 |
| 3 | rs9311642 | 5748145 | ADD | 1638 | 1.015 | 0.2123 | 0.8319 |
| 3 | rs156168 | 6398320 | ADD | 1629 | 0.8893 | -1.356 | 0.1752 |
| 3 | rs13070476 | 7234313 | ADD | 1637 | 0.9554 | -0.5016 | 0.6159 |
| 3 | rs11918634 | 7658434 | ADD | 1637 | 0.9496 | -0.6453 | 0.5187 |
| 3 | rs2619507 | 10310786 | ADD | 1638 | 1.002 | 0.01957 | 0.9844 |
| 3 | rs28280 | 10501438 | ADD | 1633 | 0.9318 | -0.9501 | 0.3421 |
| 3 | rs1865396 | 10641716 | ADD | 1638 | 0.9653 | -0.3274 | 0.7434 |
| 3 | rs1809529 | 10852611 | ADD | 1631 | 1.019 | 0.2712 | 0.7862 |
| 3 | rs2447613 | 11645573 | ADD | 1635 | 0.8792 | -1.475 | 0.1401 |
| 3 | rs3729931 | 12601516 | ADD | 1638 | 1.103 | 1.371 | 0.1704 |
| 3 | rs7634112 | 13508582 | ADD | 1606 | 1.156 | 1.515 | 0.1299 |
| 3 | rs9881693 | 14286277 | ADD | 1609 | 1.077 | 0.8036 | 0.4216 |
| 3 | rs4334629 | 17081031 | ADD | 1637 | 0.9864 | -0.1898 | 0.8495 |
| 3 | rs9881375 | 17110188 | ADD | 1633 | 0.9483 | -0.7097 | 0.4779 |
| 3 | rs1523351 | 20552477 | ADD | 1626 | 1.087 | 1.11 | 0.2672 |
| 3 | rs9825481 | 21072117 | ADD | 1636 | 0.9027 | -1.439 | 0.15 |
| 3 | rs12106696 | 21731557 | ADD | 1568 | 0.9266 | -1.042 | 0.2972 |
| 3 | rs7628655 | 21789862 | ADD | 1632 | 1.121 | 1.515 | 0.1297 |
| 3 | rs184174 | 22885441 | ADD | 1637 | 0.9528 | -0.492 | 0.6227 |
| 3 | rs7641032 | 23118465 | ADD | 1605 | 1.018 | 0.2403 | 0.8101 |
| 3 | rs6791919 | 23779748 | ADD | 1638 | 1.097 | 0.8898 | 0.3736 |
| 3 | rs4478032 | 24747000 | ADD | 1628 | 1.142 | 1.464 | 0.1432 |
| 3 | rs9881863 | 24804092 | ADD | 1635 | 1.153 | 1.314 | 0.1889 |
| 3 | rs1542542 | 27864039 | ADD | 1628 | 1.035 | 0.3439 | 0.7309 |
| 3 | rs4305453 | 30534500 | ADD | 1608 | 1.009 | 0.1211 | 0.9036 |
| 3 | rs1875688 | 30772129 | ADD | 1638 | 1.031 | 0.2555 | 0.7983 |
| 3 | rs11921928 | 31709993 | ADD | 1638 | 0.9741 | -0.3228 | 0.7468 |
| 3 | rs4678523 | 33012725 | ADD | 1630 | 1.072 | 0.8784 | 0.3797 |
| 3 | rs11920118 | 34575762 | ADD | 1632 | 1.053 | 0.6671 | 0.5047 |
| 3 | rs17033748 | 35762893 | ADD | 1629 | 1.1 | 0.3932 | 0.6942 |
| 3 | rs6599240 | 38713721 | ADD | 1560 | 0.9009 | -1.398 | 0.162 |
| 3 | rs6793877 | 43662277 | ADD | 1636 | 0.9867 | -0.1781 | 0.8586 |
| 3 | rs2159272 | 45804999 | ADD | 1636 | 1.008 | 0.1178 | 0.9062 |
| 3 | rs13064991 | 45809815 | ADD | 1580 | 1.12 | 1.194 | 0.2327 |
| 3 | rs358803 | 55293749 | ADD | 1638 | 1.022 | 0.3022 | 0.7625 |
| 3 | rs1026952 | 58620635 | ADD | 1632 | 1.087 | 1.139 | 0.2549 |
| 3 | rs4129352 | 60968955 | ADD | 1638 | 1.081 | 0.361 | 0.7181 |
| 3 | rs1376917 | 62473418 | ADD | 1638 | 1.13 | 1.126 | 0.2601 |
| 3 | rs1221474 | 63719773 | ADD | 1633 | 0.9893 | -0.1099 | 0.9125 |
| 3 | rs17045666 | 67097455 | ADD | 1565 | 0.6333 | -1.712 | 0.08696 |
| 3 | rs2101566 | 67513045 | ADD | 1611 | 0.886 | -1.608 | 0.1079 |
| 3 | rs7623610 | 70087971 | ADD | 1636 | 1.013 | 0.1853 | 0.853 |
| 3 | rs13080756 | 72663534 | ADD | 1636 | 1.029 | 0.3222 | 0.7473 |
| 3 | rs11128334 | 73521155 | ADD | 1636 | 0.9769 | -0.3124 | 0.7548 |
| 3 | rs3816831 | 73532888 | ADD | 1638 | 1.071 | 0.6654 | 0.5058 |
| 3 | rs4622849 | 73888657 | ADD | 1638 | 0.8413 | -1.369 | 0.1711 |
| 3 | rs11712680 | 75091709 | ADD | 1638 | 1.012 | 0.1194 | 0.905 |
| 3 | rs17015201 | 77468660 | ADD | 1630 | 0.8899 | -1.251 | 0.2108 |
| 3 | rs9818075 | 77469545 | ADD | 1614 | 1.1 | 1.327 | 0.1846 |
| 3 | rs4680919 | 79108259 | ADD | 1637 | 1.036 | 0.3781 | 0.7053 |
| 3 | rs17018482 | 81212634 | ADD | 1634 | 1.188 | 1.855 | 0.06353 |
| 3 | rs9820995 | 86540225 | ADD | 1629 | 1.007 | 0.08498 | 0.9323 |
| 3 | rs17026569 | 89130713 | ADD | 1637 | 0.8605 | -1.539 | 0.1239 |
| 3 | rs4894936 | 106825705 | ADD | 1637 | 1.022 | 0.3104 | 0.7563 |
| 3 | rs12637512 | 112511415 | ADD | 1638 | 0.9451 | -0.7805 | 0.4351 |
| 3 | rs10511316 | 113829769 | ADD | 1573 | 0.8828 | -1.542 | 0.1232 |
| 3 | rs1401072 | 117764397 | ADD | 1635 | 1.084 | 1.017 | 0.3094 |
| 3 | rs13088089 | 118262712 | ADD | 1626 | 0.9901 | -0.1275 | 0.8985 |
| 3 | rs2241994 | 120592696 | ADD | 1634 | 1.061 | 0.5148 | 0.6067 |
| 3 | rs1470399 | 124181451 | ADD | 1616 | 0.9948 | -0.05891 | 0.953 |
| 3 | rs33926252 | 125752222 | ADD | 1637 | 0.8392 | -1.432 | 0.152 |
| 3 | rs9968134 | 126247104 | ADD | 1636 | 0.9153 | -1.225 | 0.2205 |
| 3 | rs4679251 | 127704774 | ADD | 1634 | 0.9169 | -1.195 | 0.2319 |
| 3 | rs7610027 | 132933577 | ADD | 1637 | 1.105 | 0.9419 | 0.3462 |
| 3 | rs9840108 | 135039035 | ADD | 1638 | 0.9432 | -0.8154 | 0.4148 |
| 3 | rs6440165 | 144580138 | ADD | 1638 | 0.9092 | -1.101 | 0.2707 |
| 3 | rs6782465 | 144584003 | ADD | 1637 | 0.7657 | -2.359 | 0.01835 |
| 3 | rs905142 | 144637716 | ADD | 1633 | 0.909 | -1.257 | 0.2087 |
| 3 | rs6781717 | 167699740 | ADD | 1632 | 1.076 | 0.9272 | 0.3538 |
| 3 | rs756644 | 170567521 | ADD | 1626 | 1.096 | 1.266 | 0.2055 |
| 3 | rs4894643 | 172463751 | ADD | 1608 | 1.087 | 0.9504 | 0.3419 |
| 3 | rs4854948 | 180900670 | ADD | 1624 | 0.8912 | -0.8906 | 0.3731 |
| 3 | rs13096649 | 185694678 | ADD | 1621 | 0.8392 | -2.449 | 0.01431 |
| 3 | rs4309706 | 185957520 | ADD | 1638 | 0.9069 | -1.31 | 0.1903 |
| 3 | rs12629404 | 186378157 | ADD | 1629 | 0.918 | -1.003 | 0.316 |
| 3 | rs4686880 | 188648468 | ADD | 1638 | 0.9833 | -0.1567 | 0.8755 |
| 3 | rs7636839 | 190839635 | ADD | 1638 | 1.007 | 0.1019 | 0.9188 |
| 3 | rs7641791 | 191147311 | ADD | 1638 | 1.005 | 0.06404 | 0.9489 |
| 3 | rs3773963 | 191790142 | ADD | 1633 | 1.101 | 1.026 | 0.3048 |
| 3 | rs9814777 | 194740192 | ADD | 1624 | 0.9278 | -0.7785 | 0.4363 |
| 3 | rs4493370 | 195727220 | ADD | 1638 | 0.9943 | -0.04876 | 0.9611 |
| 3 | rs7652843 | 196036174 | ADD | 1638 | 1.136 | 1.768 | 0.07706 |
| 3 | rs7629533 | 198262835 | ADD | 1635 | 1.008 | 0.09538 | 0.924 |
| 4 | rs4689626 | 7240916 | ADD | 1630 | 0.8882 | -1.17 | 0.2422 |
| 4 | rs12374320 | 9729707 | ADD | 1595 | 1.066 | 0.9172 | 0.359 |
| 4 | rs524129 | 12716280 | ADD | 1607 | 1.046 | 0.5651 | 0.572 |
| 4 | rs1019271 | 13397414 | ADD | 1623 | 0.985 | -0.2131 | 0.8312 |
| 4 | rs7697386 | 13427979 | ADD | 1633 | 0.9576 | -0.6196 | 0.5355 |
| 4 | rs1501140 | 16100382 | ADD | 1638 | 1.063 | 0.7988 | 0.4244 |
| 4 | rs13435197 | 19919374 | ADD | 1628 | 0.9641 | -0.4929 | 0.6221 |
| 4 | rs4697493 | 24508684 | ADD | 1636 | 0.9606 | -0.4118 | 0.6805 |
| 4 | rs10033468 | 25751935 | ADD | 1637 | 1.018 | 0.2499 | 0.8027 |
| 4 | rs6856464 | 26432096 | ADD | 1613 | 1.406 | 2.277 | 0.02276 |
| 4 | rs1488290 | 26806884 | ADD | 1628 | 1.081 | 1.093 | 0.2742 |
| 4 | rs6811398 | 29051674 | ADD | 1609 | 0.947 | -0.7605 | 0.447 |
| 4 | rs10050320 | 31585804 | ADD | 1589 | 1.001 | 0.01934 | 0.9846 |
| 4 | rs2270880 | 36021772 | ADD | 1638 | 1.051 | 0.4649 | 0.642 |
| 4 | rs17493545 | 37061405 | ADD | 1638 | 0.735 | -3.144 | 0.001666 |
| 4 | rs1648082 | 43380218 | ADD | 1636 | 1.056 | 0.5804 | 0.5617 |
| 4 | rs4694999 | 43881190 | ADD | 1613 | 0.8668 | -1.253 | 0.21 |
| 4 | rs7656613 | 54836600 | ADD | 1638 | 0.9885 | -0.1358 | 0.892 |
| 4 | rs1107674 | 57533447 | ADD | 1636 | 1.076 | 0.9802 | 0.327 |
| 4 | rs12499997 | 60390306 | ADD | 1637 | 0.9974 | -0.03222 | 0.9743 |
| 4 | rs2090189 | 64020195 | ADD | 1638 | 0.9725 | -0.3705 | 0.711 |
| 4 | rs6815080 | 66045082 | ADD | 1613 | 1.044 | 0.5639 | 0.5728 |
| 4 | rs6852744 | 67842380 | ADD | 1568 | 0.9865 | -0.1881 | 0.8508 |
| 4 | rs959636 | 68446389 | ADD | 1632 | 0.9723 | -0.3584 | 0.72 |
| 4 | rs168482 | 68491562 | ADD | 1601 | 0.9415 | -0.7222 | 0.4702 |
| 4 | rs6446945 | 72010366 | ADD | 1638 | 1.11 | 0.6123 | 0.5403 |
| 4 | rs919047 | 73989242 | ADD | 1630 | 0.9955 | -0.05369 | 0.9572 |
| 4 | rs2047985 | 77053233 | ADD | 1636 | 1.003 | 0.02477 | 0.9802 |
| 4 | rs7675843 | 82254091 | ADD | 1636 | 1.075 | 0.8098 | 0.4181 |
| 4 | rs17009317 | 85988880 | ADD | 1633 | 1.025 | 0.3147 | 0.753 |
| 4 | rs6857600 | 89285099 | ADD | 1638 | 0.941 | -0.7277 | 0.4668 |
| 4 | rs12507470 | 109431427 | ADD | 1634 | 0.9048 | -1.177 | 0.2391 |
| 4 | rs924758 | 112639008 | ADD | 1637 | 1.117 | 1.534 | 0.1251 |
| 4 | rs13110184 | 118300076 | ADD | 1638 | 0.9648 | -0.4794 | 0.6317 |
| 4 | rs17366987 | 121124690 | ADD | 1638 | 0.7707 | -1.873 | 0.06105 |
| 4 | rs17343008 | 121772276 | ADD | 1637 | 0.9505 | -0.6555 | 0.5121 |
| 4 | rs843575 | 121864462 | ADD | 1616 | 1.105 | 1.16 | 0.2459 |
| 4 | rs1027374 | 124502588 | ADD | 1603 | 1.019 | 0.2716 | 0.7859 |
| 4 | rs9993784 | 125869071 | ADD | 1630 | 0.9646 | -0.4604 | 0.6452 |
| 4 | rs17786141 | 126000979 | ADD | 1620 | 0.9343 | -0.9039 | 0.3661 |
| 4 | rs7670660 | 129777843 | ADD | 1637 | 1.248 | 2.495 | 0.01261 |
| 4 | rs1505413 | 133218512 | ADD | 1635 | 1.165 | 1.222 | 0.2216 |
| 4 | rs706349 | 140819757 | ADD | 1632 | 1.166 | 2.055 | 0.03983 |
| 4 | rs1521496 | 141288597 | ADD | 1634 | 1 | 0.004616 | 0.9963 |
| 4 | rs4544728 | 146987844 | ADD | 1629 | 0.9264 | -1.04 | 0.2985 |
| 4 | rs9996620 | 147692764 | ADD | 1626 | 0.9504 | -0.4841 | 0.6283 |
| 4 | rs1032328 | 149682552 | ADD | 1636 | 1.068 | 0.9221 | 0.3565 |
| 4 | rs13120414 | 154137272 | ADD | 1637 | 0.9117 | -1.288 | 0.1977 |
| 4 | rs4533716 | 163462694 | ADD | 1638 | 0.8413 | -2.4 | 0.01639 |
| 4 | rs17504069 | 166812299 | ADD | 1621 | 0.8935 | -1.523 | 0.1277 |
| 4 | rs7675062 | 167165316 | ADD | 1638 | 1.031 | 0.4136 | 0.6792 |
| 4 | rs7698485 | 167996750 | ADD | 1637 | 0.9747 | -0.3598 | 0.719 |
| 4 | rs1020231 | 174662719 | ADD | 1637 | 0.9762 | -0.3105 | 0.7562 |
| 4 | rs9999393 | 175090270 | ADD | 1633 | 1.154 | 0.9971 | 0.3187 |
| 4 | rs379519 | 179856518 | ADD | 1638 | 0.9793 | -0.293 | 0.7695 |
| 4 | rs346119 | 179920397 | ADD | 1634 | 0.9754 | -0.3519 | 0.7249 |
| 4 | rs1994816 | 181205425 | ADD | 1635 | 1.223 | 2.368 | 0.01789 |
| 4 | rs7659727 | 182034590 | ADD | 1638 | 1.004 | 0.05686 | 0.9547 |
| 4 | rs11727972 | 182129747 | ADD | 1625 | 1.055 | 0.7367 | 0.4613 |
| 4 | rs6813301 | 183096346 | ADD | 1638 | 1.009 | 0.1019 | 0.9189 |
| 4 | rs17072756 | 183232158 | ADD | 1631 | 1.165 | 1.822 | 0.06841 |
| 4 | rs335067 | 183285690 | ADD | 1637 | 1.049 | 0.6823 | 0.4951 |
| 4 | rs9985535 | 183755240 | ADD | 1602 | 1.142 | 1.867 | 0.06186 |
| 4 | rs12506141 | 186296887 | ADD | 1571 | 0.9064 | -1.349 | 0.1773 |
| 4 | rs4862571 | 186950998 | ADD | 1625 | 1.048 | 0.6294 | 0.5291 |
| 4 | rs976000 | 188464945 | ADD | 1636 | 0.9427 | -0.7445 | 0.4566 |
| 4 | rs13107630 | 189602503 | ADD | 1566 | 1.112 | 1.463 | 0.1436 |
| 4 | rs1395612 | 189670284 | ADD | 1634 | 1.031 | 0.4128 | 0.6797 |
| 5 | rs4246740 | 1239086 | ADD | 1613 | 0.9108 | -1.28 | 0.2006 |
| 5 | rs7737692 | 1514167 | ADD | 1635 | 1.324 | 3.686 | 0.0002276 |
| 5 | rs7735656 | 2203273 | ADD | 1638 | 1.018 | 0.2276 | 0.82 |
| 5 | rs6864829 | 2836869 | ADD | 1636 | 1.054 | 0.6487 | 0.5165 |
| 5 | rs16870453 | 2848776 | ADD | 1631 | 1.073 | 0.7725 | 0.4398 |
| 5 | rs2860294 | 2955999 | ADD | 1633 | 1.032 | 0.4365 | 0.6624 |
| 5 | rs160770 | 3160861 | ADD | 1635 | 0.8623 | -1.865 | 0.06215 |
| 5 | rs4476727 | 3340958 | ADD | 1637 | 0.9747 | -0.3162 | 0.7518 |
| 5 | rs959937 | 4277788 | ADD | 1637 | 1.078 | 0.9676 | 0.3332 |
| 5 | rs156251 | 5116242 | ADD | 1636 | 1.026 | 0.2689 | 0.788 |
| 5 | rs271421 | 6407132 | ADD | 1636 | 1.299 | 2.634 | 0.008432 |
| 5 | rs26737 | 7516625 | ADD | 1634 | 1.063 | 0.868 | 0.3854 |
| 5 | rs12189232 | 9201278 | ADD | 1638 | 1.025 | 0.3469 | 0.7287 |
| 5 | rs410741 | 9454606 | ADD | 1611 | 1.008 | 0.0956 | 0.9238 |
| 5 | rs118583 | 9768924 | ADD | 1637 | 1.052 | 0.6161 | 0.5378 |
| 5 | rs1911942 | 10211297 | ADD | 1638 | 1.103 | 1.391 | 0.1642 |
| 5 | rs10491237 | 10965382 | ADD | 1635 | 0.9728 | -0.3115 | 0.7554 |
| 5 | rs16901339 | 11222601 | ADD | 1637 | 0.9907 | -0.1057 | 0.9158 |
| 5 | rs1429036 | 15624736 | ADD | 1637 | 0.9928 | -0.09542 | 0.924 |
| 5 | rs32199 | 16090234 | ADD | 1616 | 0.9929 | -0.09876 | 0.9213 |
| 5 | rs953431 | 17302420 | ADD | 1637 | 1.097 | 1.026 | 0.3047 |
| 5 | rs919336 | 31806568 | ADD | 1637 | 1.089 | 1.199 | 0.2306 |
| 5 | rs283107 | 32137157 | ADD | 1638 | 0.9207 | -0.6672 | 0.5047 |
| 5 | rs599932 | 32509763 | ADD | 1616 | 1.04 | 0.5313 | 0.5952 |
| 5 | rs7712316 | 33830924 | ADD | 1619 | 1.037 | 0.3552 | 0.7224 |
| 5 | rs36651 | 36759152 | ADD | 1638 | 0.8223 | -1.843 | 0.06534 |
| 5 | rs1428256 | 38309217 | ADD | 1633 | 0.8514 | -1.731 | 0.08347 |
| 5 | rs27964 | 50188224 | ADD | 1638 | 1.058 | 0.8024 | 0.4223 |
| 5 | rs17220752 | 50408414 | ADD | 1637 | 0.8489 | -1.853 | 0.06389 |
| 5 | rs1450949 | 51954893 | ADD | 1634 | 0.9377 | -0.8268 | 0.4083 |
| 5 | rs16885510 | 55800618 | ADD | 1638 | 1.098 | 0.7453 | 0.4561 |
| 5 | rs10939931 | 61512658 | ADD | 1636 | 1.004 | 0.04442 | 0.9646 |
| 5 | rs6449611 | 62114079 | ADD | 1637 | 1.053 | 0.6303 | 0.5285 |
| 5 | rs7725568 | 66151738 | ADD | 1637 | 1.157 | 1.891 | 0.05863 |
| 5 | rs17237118 | 67441983 | ADD | 1638 | 1.077 | 0.8894 | 0.3738 |
| 5 | rs1104891 | 72506411 | ADD | 1636 | 1.007 | 0.09958 | 0.9207 |
| 5 | rs10462509 | 74285663 | ADD | 1606 | 0.8326 | -0.986 | 0.3241 |
| 5 | rs7707801 | 75866793 | ADD | 1637 | 1.036 | 0.4907 | 0.6237 |
| 5 | rs254411 | 77115613 | ADD | 1627 | 0.9066 | -1.354 | 0.1757 |
| 5 | rs7725100 | 77280552 | ADD | 1632 | 1.017 | 0.2192 | 0.8265 |
| 5 | rs3749683 | 79130901 | ADD | 1622 | 0.9531 | -0.3909 | 0.6959 |
| 5 | rs4704624 | 79516376 | ADD | 1621 | 1.153 | 1.953 | 0.0508 |
| 5 | rs4916821 | 90126155 | ADD | 1638 | 0.8732 | -1.386 | 0.1657 |
| 5 | rs2137009 | 90529870 | ADD | 1636 | 0.8148 | -1.573 | 0.1158 |
| 5 | rs3762986 | 95796618 | ADD | 1638 | 0.9481 | -0.7315 | 0.4645 |
| 5 | rs7727752 | 97455142 | ADD | 1637 | 1.092 | 0.9317 | 0.3515 |
| 5 | rs1482900 | 105172733 | ADD | 1638 | 0.8074 | -2.064 | 0.03902 |
| 5 | rs872622 | 111127055 | ADD | 1633 | 1.007 | 0.0994 | 0.9208 |
| 5 | rs2061448 | 116404589 | ADD | 1637 | 0.94 | -0.7777 | 0.4367 |
| 5 | rs304379 | 121796570 | ADD | 1638 | 0.9591 | -0.5782 | 0.5631 |
| 5 | rs2147866 | 127263913 | ADD | 1638 | 0.964 | -0.5149 | 0.6066 |
| 5 | rs10519976 | 127556779 | ADD | 1629 | 0.9395 | -0.7002 | 0.4838 |
| 5 | rs30645 | 129043351 | ADD | 1634 | 0.9994 | -0.007918 | 0.9937 |
| 5 | rs2237077 | 139700584 | ADD | 1606 | 1.254 | 2.821 | 0.004786 |
| 5 | rs4912610 | 141034424 | ADD | 1597 | 1.109 | 1.047 | 0.2949 |
| 5 | rs153516 | 142959376 | ADD | 1637 | 0.9789 | -0.284 | 0.7764 |
| 5 | rs1549896 | 144242936 | ADD | 1637 | 0.8637 | -1.018 | 0.3085 |
| 5 | rs452399 | 146198436 | ADD | 1635 | 1.062 | 0.7793 | 0.4358 |
| 5 | rs10515577 | 146203007 | ADD | 1620 | 1.11 | 1.008 | 0.3134 |
| 5 | rs11750184 | 148826711 | ADD | 1637 | 0.9155 | -1.065 | 0.287 |
| 5 | rs4705377 | 149143607 | ADD | 1638 | 1.037 | 0.4892 | 0.6247 |
| 5 | rs9688182 | 149669594 | ADD | 1635 | 1.052 | 0.7064 | 0.4799 |
| 5 | rs17111695 | 150412639 | ADD | 1635 | 0.9184 | -0.8742 | 0.382 |
| 5 | rs966088 | 152389223 | ADD | 1631 | 1.031 | 0.3936 | 0.6939 |
| 5 | rs1209063 | 157275238 | ADD | 1635 | 1.175 | 2.251 | 0.02442 |
| 5 | rs4334868 | 158916865 | ADD | 1637 | 1.038 | 0.4561 | 0.6483 |
| 5 | rs883517 | 159837307 | ADD | 1637 | 1.023 | 0.2558 | 0.7981 |
| 5 | rs3926173 | 163663585 | ADD | 1637 | 1.137 | 1.421 | 0.1553 |
| 5 | rs17324544 | 164225627 | ADD | 1636 | 0.9692 | -0.4092 | 0.6824 |
| 5 | rs1862370 | 164844511 | ADD | 1637 | 0.9743 | -0.148 | 0.8824 |
| 5 | rs7714651 | 167162881 | ADD | 1636 | 0.9163 | -0.9079 | 0.3639 |
| 5 | rs1477284 | 167217586 | ADD | 1630 | 0.8816 | -1.789 | 0.07355 |
| 5 | rs4867879 | 169064953 | ADD | 1627 | 0.8889 | -1.675 | 0.0939 |
| 5 | rs17669654 | 169136824 | ADD | 1626 | 1.139 | 1.343 | 0.1793 |
| 5 | rs261625 | 169200769 | ADD | 1616 | 0.873 | -1.907 | 0.05651 |
| 5 | rs17738959 | 169462481 | ADD | 1612 | 1.09 | 1.126 | 0.2602 |
| 5 | rs2339316 | 170843820 | ADD | 1574 | 1 | 0.004864 | 0.9961 |
| 5 | rs7711912 | 170853308 | ADD | 1638 | 1.021 | 0.2901 | 0.7717 |
| 5 | rs895312 | 172697004 | ADD | 1630 | 0.956 | -0.3576 | 0.7206 |
| 5 | rs17733311 | 172712710 | ADD | 1638 | 1.016 | 0.1753 | 0.8608 |
| 5 | rs6556114 | 173504283 | ADD | 1629 | 0.9428 | -0.793 | 0.4278 |
| 5 | rs2436319 | 173620108 | ADD | 1638 | 0.945 | -0.7663 | 0.4435 |
| 5 | rs6601221 | 177507027 | ADD | 1638 | 0.8771 | -1.77 | 0.0767 |
| 5 | rs9717043 | 178136191 | ADD | 1627 | 0.9372 | -0.5761 | 0.5646 |
| 6 | rs9405444 | 1114047 | ADD | 1638 | 0.9579 | -0.5136 | 0.6075 |
| 6 | rs9328052 | 1388759 | ADD | 1633 | 1.085 | 0.761 | 0.4467 |
| 6 | rs1474721 | 1400569 | ADD | 1621 | 1.013 | 0.1618 | 0.8715 |
| 6 | rs12197419 | 1654036 | ADD | 1635 | 0.9847 | -0.2117 | 0.8323 |
| 6 | rs1157673 | 1769440 | ADD | 1636 | 1.079 | 0.9588 | 0.3377 |
| 6 | rs160666 | 2719051 | ADD | 1638 | 0.931 | -0.8983 | 0.369 |
| 6 | rs9504439 | 5539198 | ADD | 1638 | 0.9804 | -0.2835 | 0.7768 |
| 6 | rs4960121 | 5594265 | ADD | 1637 | 0.9102 | -1.169 | 0.2424 |
| 6 | rs11969912 | 6120974 | ADD | 1609 | 1.115 | 1.415 | 0.1572 |
| 6 | rs2327112 | 8944645 | ADD | 1625 | 0.9781 | -0.3033 | 0.7616 |
| 6 | rs2070699 | 12400758 | ADD | 1638 | 0.9447 | -0.797 | 0.4254 |
| 6 | rs1317584 | 12450775 | ADD | 1637 | 0.9393 | -0.7481 | 0.4544 |
| 6 | rs2050288 | 12462373 | ADD | 1610 | 0.9874 | -0.1568 | 0.8754 |
| 6 | rs10949221 | 14154469 | ADD | 1637 | 1.029 | 0.3061 | 0.7595 |
| 6 | rs2092204 | 14201535 | ADD | 1637 | 0.9643 | -0.5032 | 0.6148 |
| 6 | rs441539 | 15872617 | ADD | 1637 | 1.038 | 0.4975 | 0.6188 |
| 6 | rs2056937 | 16252593 | ADD | 1636 | 1.102 | 1.317 | 0.1878 |
| 6 | rs6902696 | 16915071 | ADD | 1635 | 1.296 | 2.914 | 0.003574 |
| 6 | rs823433 | 17602442 | ADD | 1638 | 0.9276 | -0.7504 | 0.453 |
| 6 | rs7744164 | 18278503 | ADD | 1631 | 1.072 | 0.9653 | 0.3344 |
| 6 | rs973244 | 19934609 | ADD | 1607 | 0.8821 | -1.663 | 0.09622 |
| 6 | rs2457336 | 20215406 | ADD | 1638 | 1.067 | 0.8689 | 0.3849 |
| 6 | rs2076890 | 25528723 | ADD | 1576 | 1.178 | 1.636 | 0.1018 |
| 6 | rs6935041 | 33961697 | ADD | 1629 | 0.942 | -0.8459 | 0.3976 |
| 6 | rs6918981 | 34346492 | ADD | 1636 | 1.116 | 1.378 | 0.1681 |
| 6 | rs9394287 | 35324718 | ADD | 1636 | 1.113 | 0.9217 | 0.3567 |
| 6 | rs1776447 | 37710438 | ADD | 1638 | 1.225 | 2.064 | 0.03903 |
| 6 | rs9296264 | 38908879 | ADD | 1637 | 0.922 | -1.052 | 0.2927 |
| 6 | rs932443 | 39150312 | ADD | 1636 | 0.9738 | -0.3378 | 0.7355 |
| 6 | rs9462479 | 39213881 | ADD | 1598 | 0.8513 | -0.9235 | 0.3558 |
| 6 | rs6901022 | 39414041 | ADD | 1638 | 0.9075 | -1.278 | 0.2014 |
| 6 | rs1018631 | 40379505 | ADD | 1632 | 0.9952 | -0.06821 | 0.9456 |
| 6 | rs9357360 | 41490550 | ADD | 1638 | 0.9413 | -0.7243 | 0.4689 |
| 6 | rs2477838 | 41589138 | ADD | 1635 | 1.078 | 0.985 | 0.3246 |
| 6 | rs6941212 | 43023898 | ADD | 1618 | 1.025 | 0.3385 | 0.735 |
| 6 | rs700004 | 44140309 | ADD | 1606 | 0.9977 | -0.02453 | 0.9804 |
| 6 | rs4714738 | 44164563 | ADD | 1638 | 1.016 | 0.1485 | 0.882 |
| 6 | rs11758366 | 47361590 | ADD | 1629 | 0.9898 | -0.1131 | 0.91 |
| 6 | rs742552 | 52234664 | ADD | 1626 | 1.047 | 0.606 | 0.5445 |
| 6 | rs6915699 | 53124265 | ADD | 1624 | 1.161 | 1.53 | 0.1261 |
| 6 | rs3805994 | 70823225 | ADD | 1627 | 0.9998 | -0.002999 | 0.9976 |
| 6 | rs782002 | 71424287 | ADD | 1632 | 0.9757 | -0.346 | 0.7293 |
| 6 | rs11753467 | 74103723 | ADD | 1638 | 1.029 | 0.2288 | 0.819 |
| 6 | rs9352668 | 79629397 | ADD | 1611 | 0.962 | -0.5271 | 0.5982 |
| 6 | rs735641 | 85008568 | ADD | 1637 | 0.8765 | -1.47 | 0.1416 |
| 6 | rs188750 | 86099356 | ADD | 1635 | 0.8945 | -1.314 | 0.1889 |
| 6 | rs10944288 | 87708461 | ADD | 1638 | 0.995 | -0.06733 | 0.9463 |
| 6 | rs10944335 | 88714066 | ADD | 1637 | 0.9347 | -0.9119 | 0.3618 |
| 6 | rs990060 | 89131055 | ADD | 1635 | 1.026 | 0.3499 | 0.7264 |
| 6 | rs9444725 | 90770775 | ADD | 1612 | 0.8182 | -2.435 | 0.01488 |
| 6 | rs791059 | 91249278 | ADD | 1636 | 0.9084 | -1.333 | 0.1826 |
| 6 | rs806272 | 91260936 | ADD | 1627 | 1.063 | 0.8553 | 0.3924 |
| 6 | rs6918777 | 93426376 | ADD | 1638 | 1.093 | 1.257 | 0.2087 |
| 6 | rs9373250 | 100362192 | ADD | 1608 | 0.9883 | -0.1519 | 0.8793 |
| 6 | rs17760780 | 102095420 | ADD | 1567 | 0.9179 | -0.9419 | 0.3463 |
| 6 | rs4142560 | 105087171 | ADD | 1606 | 0.9512 | -0.6732 | 0.5008 |
| 6 | rs6940468 | 105192818 | ADD | 1628 | 1.077 | 0.695 | 0.4871 |
| 6 | rs6922009 | 110925752 | ADD | 1638 | 0.936 | -0.779 | 0.436 |
| 6 | rs9400661 | 114147623 | ADD | 1638 | 1.13 | 1.647 | 0.09959 |
| 6 | rs1031800 | 114256717 | ADD | 1605 | 0.9329 | -0.8378 | 0.4021 |
| 6 | rs4962262 | 123214382 | ADD | 1613 | 1.094 | 1.037 | 0.2996 |
| 6 | rs492800 | 125444410 | ADD | 1638 | 1.01 | 0.1431 | 0.8862 |
| 6 | rs1341200 | 125688823 | ADD | 1638 | 0.9281 | -0.8899 | 0.3735 |
| 6 | rs17065090 | 136264929 | ADD | 1638 | 1.156 | 0.9038 | 0.3661 |
| 6 | rs5029939 | 138237416 | ADD | 1635 | 1.019 | 0.1554 | 0.8765 |
| 6 | rs4385332 | 141484518 | ADD | 1629 | 0.9491 | -0.6613 | 0.5084 |
| 6 | rs10484601 | 148937666 | ADD | 1607 | 0.9587 | -0.4401 | 0.6599 |
| 6 | rs659156 | 150454526 | ADD | 1628 | 0.9799 | -0.2659 | 0.7903 |
| 6 | rs9397629 | 150464350 | ADD | 1617 | 1.061 | 0.5158 | 0.606 |
| 6 | rs17080320 | 151154676 | ADD | 1627 | 1.629 | 2.027 | 0.0427 |
| 6 | rs9383889 | 151772495 | ADD | 1630 | 1.001 | 0.015 | 0.988 |
| 6 | rs766127 | 153355525 | ADD | 1638 | 1.208 | 2.374 | 0.01758 |
| 6 | rs672170 | 153386878 | ADD | 1635 | 1.175 | 2.155 | 0.03114 |
| 6 | rs9322484 | 155286276 | ADD | 1634 | 0.9199 | -1.199 | 0.2307 |
| 6 | rs2025641 | 158318990 | ADD | 1637 | 0.8981 | -1.484 | 0.1379 |
| 6 | rs6455652 | 159847589 | ADD | 1637 | 1.063 | 0.8382 | 0.4019 |
| 6 | rs1488 | 161458240 | ADD | 1630 | 0.9688 | -0.4265 | 0.6697 |
| 6 | rs2293289 | 161471429 | ADD | 1636 | 0.9888 | -0.09888 | 0.9212 |
| 6 | rs1125640 | 161512090 | ADD | 1599 | 0.6953 | -2.371 | 0.01772 |
| 6 | rs868708 | 163179250 | ADD | 1636 | 1.098 | 1.323 | 0.1857 |
| 6 | rs2874453 | 163229786 | ADD | 1625 | 0.9165 | -1.055 | 0.2915 |
| 6 | rs10945909 | 163947534 | ADD | 1638 | 1.012 | 0.1649 | 0.869 |
| 6 | rs1039002 | 166075447 | ADD | 1636 | 1.264 | 1.569 | 0.1166 |
| 6 | rs697471 | 166299886 | ADD | 1638 | 0.9868 | -0.1882 | 0.8507 |
| 6 | rs697482 | 166341627 | ADD | 1594 | 1.058 | 0.631 | 0.528 |
| 6 | rs2187909 | 166871129 | ADD | 1625 | 0.9902 | -0.1313 | 0.8955 |
| 6 | rs2981956 | 167619694 | ADD | 1638 | 1.021 | 0.2784 | 0.7807 |
| 6 | rs4708582 | 169213632 | ADD | 1631 | 1.049 | 0.6593 | 0.5097 |
| 7 | rs11773532 | 3286698 | ADD | 1636 | 0.9137 | -1.169 | 0.2424 |
| 7 | rs10807911 | 4779909 | ADD | 1636 | 1.019 | 0.214 | 0.8306 |
| 7 | rs10234709 | 5342068 | ADD | 1590 | 0.9878 | -0.1657 | 0.8684 |
| 7 | rs852517 | 5664105 | ADD | 1632 | 1.039 | 0.4773 | 0.6331 |
| 7 | rs308092 | 5850048 | ADD | 1628 | 1.011 | 0.1652 | 0.8688 |
| 7 | rs6945447 | 7641130 | ADD | 1637 | 1.059 | 0.7835 | 0.4333 |
| 7 | rs4720815 | 8986351 | ADD | 1635 | 0.8472 | -1.562 | 0.1184 |
| 7 | rs10224784 | 11930901 | ADD | 1636 | 1.023 | 0.3133 | 0.754 |
| 7 | rs976852 | 12044688 | ADD | 1605 | 0.9302 | -0.9871 | 0.3236 |
| 7 | rs10256019 | 12901301 | ADD | 1618 | 0.9782 | -0.2239 | 0.8229 |
| 7 | rs1990077 | 14006654 | ADD | 1608 | 1.069 | 0.9316 | 0.3515 |
| 7 | rs6461292 | 16970912 | ADD | 1633 | 0.867 | -1.689 | 0.09115 |
| 7 | rs847440 | 16984957 | ADD | 1628 | 0.8576 | -2.067 | 0.03875 |
| 7 | rs17345625 | 18149018 | ADD | 1627 | 0.8629 | -0.9556 | 0.3393 |
| 7 | rs1178156 | 18749695 | ADD | 1636 | 0.9785 | -0.2625 | 0.7929 |
| 7 | rs756853 | 18856525 | ADD | 1637 | 0.9987 | -0.01812 | 0.9855 |
| 7 | rs7784579 | 20493949 | ADD | 1627 | 0.9494 | -0.7312 | 0.4647 |
| 7 | rs10234192 | 20550198 | ADD | 1570 | 0.9808 | -0.2114 | 0.8326 |
| 7 | rs999601 | 20562964 | ADD | 1630 | 0.9306 | -0.9914 | 0.3215 |
| 7 | rs1637078 | 22064001 | ADD | 1613 | 1.048 | 0.4208 | 0.6739 |
| 7 | rs16873129 | 22111609 | ADD | 1632 | 1.223 | 1.443 | 0.149 |
| 7 | rs1122913 | 22569457 | ADD | 1633 | 0.9392 | -0.8537 | 0.3933 |
| 7 | rs724080 | 22578824 | ADD | 1634 | 1.077 | 0.6864 | 0.4924 |
| 7 | rs11765793 | 25336560 | ADD | 1636 | 1.072 | 0.6762 | 0.4989 |
| 7 | rs10951191 | 28089502 | ADD | 1635 | 1.112 | 1.343 | 0.1793 |
| 7 | rs148735 | 28525090 | ADD | 1632 | 1.095 | 1.153 | 0.2489 |
| 7 | rs150613 | 28583155 | ADD | 1636 | 0.9258 | -0.9011 | 0.3676 |
| 7 | rs17156863 | 28585563 | ADD | 1632 | 0.9587 | -0.5015 | 0.616 |
| 7 | rs317700 | 29003618 | ADD | 1638 | 0.9326 | -0.5016 | 0.6159 |
| 7 | rs2252521 | 29007715 | ADD | 1624 | 0.929 | -0.9614 | 0.3364 |
| 7 | rs1420133 | 29495598 | ADD | 1631 | 0.9954 | -0.06067 | 0.9516 |
| 7 | rs7780166 | 30161583 | ADD | 1638 | 0.9448 | -0.7951 | 0.4266 |
| 7 | rs11771217 | 30749288 | ADD | 1635 | 1.067 | 0.9187 | 0.3582 |
| 7 | rs6961368 | 31976452 | ADD | 1628 | 0.9982 | -0.02498 | 0.9801 |
| 7 | rs2392147 | 32833248 | ADD | 1631 | 1.116 | 1.585 | 0.113 |
| 7 | rs1421341 | 33731535 | ADD | 1599 | 1.112 | 0.6055 | 0.5448 |
| 7 | rs7793728 | 36028310 | ADD | 1637 | 0.8269 | -2.241 | 0.02505 |
| 7 | rs2299945 | 38408168 | ADD | 1634 | 1.008 | 0.108 | 0.914 |
| 7 | rs10263645 | 38603416 | ADD | 1636 | 1.062 | 0.7585 | 0.4482 |
| 7 | rs2237400 | 39416508 | ADD | 1633 | 0.9662 | -0.4655 | 0.6416 |
| 7 | rs1113117 | 39619158 | ADD | 1636 | 0.9626 | -0.5174 | 0.6049 |
| 7 | rs1990136 | 41038100 | ADD | 1622 | 0.8778 | -1.193 | 0.2328 |
| 7 | rs2190947 | 43124493 | ADD | 1637 | 1.112 | 1.451 | 0.1467 |
| 7 | rs2024125 | 43125002 | ADD | 1589 | 1.072 | 0.9646 | 0.3348 |
| 7 | rs2979422 | 44128880 | ADD | 1605 | 1.15 | 1.466 | 0.1427 |
| 7 | rs17488993 | 45520206 | ADD | 1626 | 1.068 | 0.567 | 0.5707 |
| 7 | rs1462278 | 46399128 | ADD | 1638 | 1.022 | 0.3036 | 0.7614 |
| 7 | rs7787946 | 47756443 | ADD | 1638 | 1.082 | 1.091 | 0.2752 |
| 7 | rs9639018 | 48084209 | ADD | 1638 | 0.9271 | -1.024 | 0.3058 |
| 7 | rs1035042 | 52420218 | ADD | 1633 | 0.9835 | -0.2353 | 0.814 |
| 7 | rs6945518 | 52562247 | ADD | 1634 | 1.05 | 0.5646 | 0.5723 |
| 7 | rs2960632 | 52626737 | ADD | 1638 | 1.015 | 0.2031 | 0.8391 |
| 7 | rs6956675 | 62215205 | ADD | 1612 | 1.05 | 0.5873 | 0.557 |
| 7 | rs10233260 | 67243468 | ADD | 1612 | 1.03 | 0.3748 | 0.7078 |
| 7 | rs12538253 | 75225967 | ADD | 1578 | 1.036 | 0.4251 | 0.6708 |
| 7 | rs2215379 | 77888531 | ADD | 1637 | 0.9416 | -0.7443 | 0.4567 |
| 7 | rs13243127 | 77910933 | ADD | 1636 | 1.109 | 1.495 | 0.1348 |
| 7 | rs10485891 | 77918756 | ADD | 1623 | 1.032 | 0.374 | 0.7084 |
| 7 | rs7782195 | 77957867 | ADD | 1637 | 0.8912 | -1.57 | 0.1165 |
| 7 | rs17154432 | 80245639 | ADD | 1631 | 0.6294 | -1.919 | 0.05504 |
| 7 | rs12704597 | 90671120 | ADD | 1636 | 1.011 | 0.1204 | 0.9041 |
| 7 | rs2299267 | 94899857 | ADD | 1638 | 1.156 | 1.494 | 0.1353 |
| 7 | rs3757707 | 94904911 | ADD | 1638 | 0.9469 | -0.7208 | 0.4711 |
| 7 | rs17252863 | 97022359 | ADD | 1638 | 0.9782 | -0.1858 | 0.8526 |
| 7 | rs759658 | 97156083 | ADD | 1638 | 0.921 | -0.7679 | 0.4425 |
| 7 | rs1557689 | 103795122 | ADD | 1633 | 0.9918 | -0.0983 | 0.9217 |
| 7 | rs6465984 | 103797503 | ADD | 1594 | 0.8895 | -1.032 | 0.3021 |
| 7 | rs12705284 | 104284914 | ADD | 1638 | 0.9057 | -1.393 | 0.1636 |
| 7 | rs41813 | 106028727 | ADD | 1637 | 0.9309 | -0.8701 | 0.3842 |
| 7 | rs10953518 | 106187118 | ADD | 1638 | 1.115 | 0.9889 | 0.3227 |
| 7 | rs7787898 | 106197133 | ADD | 1570 | 0.9121 | -1.32 | 0.1867 |
| 7 | rs10248657 | 112773255 | ADD | 1631 | 1.083 | 0.9844 | 0.3249 |
| 7 | rs2191500 | 115925902 | ADD | 1605 | 1.014 | 0.177 | 0.8595 |
| 7 | rs10487483 | 126861374 | ADD | 1595 | 0.9848 | -0.1964 | 0.8443 |
| 7 | rs3800717 | 134161045 | ADD | 1632 | 0.8825 | -1.171 | 0.2417 |
| 7 | rs273957 | 137251230 | ADD | 1633 | 0.9077 | -1.321 | 0.1864 |
| 7 | rs11971702 | 139767015 | ADD | 1636 | 0.8805 | -1.751 | 0.08 |
| 7 | rs2966701 | 142913704 | ADD | 1638 | 1.076 | 0.8547 | 0.3927 |
| 7 | rs2693405 | 145870841 | ADD | 1638 | 0.9462 | -0.617 | 0.5373 |
| 7 | rs874607 | 150695805 | ADD | 1571 | 0.9524 | -0.514 | 0.6073 |
| 7 | rs13234689 | 150697474 | ADD | 1628 | 0.8784 | -1.819 | 0.06889 |
| 7 | rs3823527 | 154279263 | ADD | 1636 | 1.032 | 0.3751 | 0.7076 |
| 7 | rs6962792 | 155503804 | ADD | 1602 | 1.157 | 1.887 | 0.05916 |
| 7 | rs1880342 | 155900937 | ADD | 1638 | 0.9548 | -0.6402 | 0.522 |
| 7 | rs6966038 | 156573159 | ADD | 1630 | 0.7083 | -4.146 | 3.38e-05 |
| 7 | rs6970138 | 157119751 | ADD | 1636 | 1.003 | 0.03782 | 0.9698 |
| 7 | rs2366645 | 157150776 | ADD | 1638 | 1.094 | 1.26 | 0.2078 |
| 7 | rs17837793 | 157157183 | ADD | 1632 | 0.8658 | -0.958 | 0.338 |
| 8 | rs4876266 | 1903551 | ADD | 1638 | 1.22 | 2.164 | 0.03049 |
| 8 | rs2840445 | 5752350 | ADD | 1637 | 1.05 | 0.617 | 0.5372 |
| 8 | rs6993405 | 6069641 | ADD | 1638 | 0.9202 | -0.965 | 0.3345 |
| 8 | rs7842755 | 6737180 | ADD | 1628 | 0.9744 | -0.3203 | 0.7487 |
| 8 | rs10107215 | 8877993 | ADD | 1624 | 1.013 | 0.1677 | 0.8668 |
| 8 | rs3808518 | 11180682 | ADD | 1637 | 1.036 | 0.4924 | 0.6225 |
| 8 | rs17127548 | 13142719 | ADD | 1634 | 0.9602 | -0.2785 | 0.7806 |
| 8 | rs1481590 | 13498789 | ADD | 1638 | 1.018 | 0.2564 | 0.7977 |
| 8 | rs7003979 | 15216360 | ADD | 1636 | 1.209 | 2.656 | 0.00791 |
| 8 | rs4601360 | 17224369 | ADD | 1638 | 0.9062 | -1.371 | 0.1705 |
| 8 | rs7387668 | 17657886 | ADD | 1599 | 1.024 | 0.2526 | 0.8006 |
| 8 | rs447516 | 17677017 | ADD | 1606 | 1.016 | 0.1862 | 0.8523 |
| 8 | rs7388381 | 18026157 | ADD | 1622 | 1.054 | 0.6661 | 0.5054 |
| 8 | rs11783436 | 18401677 | ADD | 1635 | 0.9635 | -0.5292 | 0.5967 |
| 8 | rs36110451 | 18971080 | ADD | 1638 | 0.9698 | -0.4182 | 0.6758 |
| 8 | rs691605 | 19072929 | ADD | 1628 | 1.089 | 1.098 | 0.2721 |
| 8 | rs4922155 | 20198056 | ADD | 1621 | 1.068 | 0.8316 | 0.4056 |
| 8 | rs1376010 | 20637044 | ADD | 1638 | 1.004 | 0.03246 | 0.9741 |
| 8 | rs11991347 | 22699268 | ADD | 1613 | 1.056 | 0.7648 | 0.4444 |
| 8 | rs4872211 | 23983658 | ADD | 1609 | 0.8672 | -1.464 | 0.1432 |
| 8 | rs1918211 | 25049587 | ADD | 1612 | 0.8655 | -0.9341 | 0.3502 |
| 8 | rs2433086 | 25586078 | ADD | 1619 | 0.9761 | -0.3277 | 0.7432 |
| 8 | rs17059088 | 28356379 | ADD | 1638 | 0.8007 | -2.529 | 0.01142 |
| 8 | rs1060411 | 28358684 | ADD | 1572 | 1.011 | 0.1453 | 0.8845 |
| 8 | rs11786308 | 28677088 | ADD | 1562 | 1.047 | 0.5578 | 0.577 |
| 8 | rs7016719 | 29598440 | ADD | 1638 | 0.9484 | -0.5979 | 0.5499 |
| 8 | rs16876103 | 29857274 | ADD | 1603 | 1.119 | 0.5584 | 0.5766 |
| 8 | rs12676965 | 37800584 | ADD | 1631 | 1.084 | 0.8412 | 0.4002 |
| 8 | rs16919588 | 54914244 | ADD | 1620 | 1.034 | 0.1674 | 0.8671 |
| 8 | rs17789704 | 57961918 | ADD | 1559 | 1.177 | 1.688 | 0.09133 |
| 8 | rs2726599 | 59881250 | ADD | 1637 | 1.037 | 0.4867 | 0.6265 |
| 8 | rs367116 | 60092116 | ADD | 1635 | 0.9673 | -0.376 | 0.7069 |
| 8 | rs4554462 | 62017395 | ADD | 1632 | 1.008 | 0.1139 | 0.9093 |
| 8 | rs2956305 | 66170843 | ADD | 1622 | 0.934 | -0.9576 | 0.3382 |
| 8 | rs4737771 | 67354933 | ADD | 1638 | 1.255 | 2.005 | 0.04492 |
| 8 | rs6999098 | 69072314 | ADD | 1581 | 1.121 | 1.481 | 0.1386 |
| 8 | rs7836791 | 71022178 | ADD | 1607 | 0.9436 | -0.7779 | 0.4366 |
| 8 | rs1812736 | 76461693 | ADD | 1636 | 0.8679 | -1.482 | 0.1384 |
| 8 | rs16939127 | 76736389 | ADD | 1637 | 1.059 | 0.7195 | 0.4719 |
| 8 | rs1821018 | 77316621 | ADD | 1635 | 0.9743 | -0.2694 | 0.7876 |
| 8 | rs4739755 | 81665747 | ADD | 1627 | 1.051 | 0.5137 | 0.6074 |
| 8 | rs10808857 | 85194559 | ADD | 1636 | 0.9949 | -0.07155 | 0.943 |
| 8 | rs10097366 | 89125685 | ADD | 1635 | 0.9384 | -0.7674 | 0.4428 |
| 8 | rs7837480 | 94537554 | ADD | 1636 | 0.9459 | -0.7749 | 0.4384 |
| 8 | rs4336580 | 94606241 | ADD | 1638 | 0.9427 | -0.7919 | 0.4284 |
| 8 | rs4735277 | 95308504 | ADD | 1624 | 1.156 | 1.035 | 0.3007 |
| 8 | rs7017245 | 97169636 | ADD | 1633 | 0.9861 | -0.1964 | 0.8443 |
| 8 | rs10110404 | 98296183 | ADD | 1630 | 1.044 | 0.569 | 0.5694 |
| 8 | rs2444895 | 99090125 | ADD | 1637 | 1.121 | 1.513 | 0.1302 |
| 8 | rs8218 | 99184146 | ADD | 1634 | 1.024 | 0.2704 | 0.7869 |
| 8 | rs1460930 | 101183970 | ADD | 1637 | 0.8295 | -2.549 | 0.01079 |
| 8 | rs2380244 | 101894560 | ADD | 1635 | 1.065 | 0.8822 | 0.3776 |
| 8 | rs895602 | 102202011 | ADD | 1588 | 1.129 | 1.713 | 0.08673 |
| 8 | rs2515173 | 104190450 | ADD | 1638 | 1.029 | 0.4133 | 0.6794 |
| 8 | rs2454000 | 104205020 | ADD | 1638 | 1.006 | 0.06777 | 0.946 |
| 8 | rs2176751 | 121106373 | ADD | 1637 | 1.018 | 0.2307 | 0.8175 |
| 8 | rs13438834 | 121887476 | ADD | 1638 | 1.071 | 0.8458 | 0.3976 |
| 8 | rs4870806 | 123716146 | ADD | 1636 | 1.116 | 1.538 | 0.124 |
| 8 | rs7821956 | 126653633 | ADD | 1629 | 0.9745 | -0.2751 | 0.7832 |
| 8 | rs7011739 | 127727329 | ADD | 1636 | 0.9818 | -0.2402 | 0.8102 |
| 8 | rs13275576 | 129358294 | ADD | 1637 | 0.9004 | -0.9992 | 0.3177 |
| 8 | rs1835851 | 130422409 | ADD | 1634 | 0.9862 | -0.1753 | 0.8608 |
| 8 | rs16904196 | 131123472 | ADD | 1621 | 1.11 | 0.5117 | 0.6089 |
| 8 | rs4410884 | 134256900 | ADD | 1633 | 0.999 | -0.01354 | 0.9892 |
| 8 | rs11787445 | 135388710 | ADD | 1632 | 0.98 | -0.2352 | 0.8141 |
| 8 | rs4909801 | 135948341 | ADD | 1637 | 0.9832 | -0.2189 | 0.8267 |
| 8 | rs4909779 | 139443796 | ADD | 1618 | 1.042 | 0.4562 | 0.6482 |
| 8 | rs17725186 | 140574685 | ADD | 1630 | 1.09 | 1.162 | 0.2453 |
| 8 | rs2471083 | 140588523 | ADD | 1630 | 0.9534 | -0.6049 | 0.5452 |
| 8 | rs888345 | 140716172 | ADD | 1629 | 1.005 | 0.04715 | 0.9624 |
| 8 | rs759656 | 140739149 | ADD | 1602 | 1.244 | 2.794 | 0.0052 |
| 8 | rs7819785 | 140962053 | ADD | 1629 | 1.023 | 0.2382 | 0.8117 |
| 8 | rs6578061 | 141114914 | ADD | 1631 | 0.9628 | -0.5424 | 0.5875 |
| 8 | rs4961309 | 141583366 | ADD | 1637 | 0.9331 | -0.772 | 0.4401 |
| 8 | rs7001673 | 142267778 | ADD | 1618 | 1.081 | 1.086 | 0.2774 |
| 9 | rs4142436 | 1444200 | ADD | 1638 | 1.084 | 0.6768 | 0.4985 |
| 9 | rs12336107 | 4300558 | ADD | 1637 | 1.008 | 0.1117 | 0.9111 |
| 9 | rs7850837 | 4856840 | ADD | 1637 | 1.005 | 0.06424 | 0.9488 |
| 9 | rs9299064 | 8198999 | ADD | 1624 | 0.9604 | -0.5562 | 0.5781 |
| 9 | rs440238 | 9006071 | ADD | 1588 | 1.111 | 1.394 | 0.1632 |
| 9 | rs1331660 | 9029222 | ADD | 1608 | 1.065 | 0.5138 | 0.6074 |
| 9 | rs7027899 | 14238934 | ADD | 1631 | 1.025 | 0.3044 | 0.7608 |
| 9 | rs9298760 | 16366437 | ADD | 1591 | 1.059 | 0.7957 | 0.4262 |
| 9 | rs9407764 | 16367360 | ADD | 1632 | 0.9685 | -0.4387 | 0.6608 |
| 9 | rs10115913 | 16649259 | ADD | 1627 | 1.013 | 0.1789 | 0.858 |
| 9 | rs461567 | 19196930 | ADD | 1617 | 1.109 | 1.237 | 0.2162 |
| 9 | rs10964862 | 21141553 | ADD | 1617 | 1.054 | 0.7057 | 0.4804 |
| 9 | rs10511716 | 23063969 | ADD | 1631 | 0.969 | -0.3785 | 0.705 |
| 9 | rs7028478 | 24182964 | ADD | 1638 | 0.9152 | -1.092 | 0.2748 |
| 9 | rs7867489 | 24827608 | ADD | 1576 | 1.149 | 1.88 | 0.06013 |
| 9 | rs4316218 | 24896802 | ADD | 1632 | 1.026 | 0.3136 | 0.7538 |
| 9 | rs2225078 | 26518575 | ADD | 1569 | 1.059 | 0.5988 | 0.5493 |
| 9 | rs10115168 | 26696278 | ADD | 1637 | 1.123 | 1.315 | 0.1886 |
| 9 | rs534129 | 27204719 | ADD | 1638 | 1.021 | 0.2866 | 0.7744 |
| 9 | rs866630 | 28769376 | ADD | 1633 | 1.043 | 0.6062 | 0.5444 |
| 9 | rs7871600 | 28930111 | ADD | 1609 | 1.082 | 0.8527 | 0.3938 |
| 9 | rs10968884 | 29010053 | ADD | 1636 | 1.134 | 1.192 | 0.2333 |
| 9 | rs10969027 | 29234767 | ADD | 1638 | 0.858 | -2.029 | 0.04245 |
| 9 | rs7027564 | 32309302 | ADD | 1638 | 0.9327 | -0.6522 | 0.5143 |
| 9 | rs16918958 | 33026928 | ADD | 1630 | 0.8709 | -0.8244 | 0.4097 |
| 9 | rs894520 | 38179527 | ADD | 1638 | 0.9489 | -0.7109 | 0.4772 |
| 9 | rs10973734 | 38286609 | ADD | 1631 | 1.038 | 0.3128 | 0.7545 |
| 9 | rs786318 | 78423133 | ADD | 1628 | 1.019 | 0.257 | 0.7972 |
| 9 | rs7861307 | 78585692 | ADD | 1638 | 0.9537 | -0.526 | 0.5989 |
| 9 | rs1410502 | 81844152 | ADD | 1624 | 0.9684 | -0.3283 | 0.7427 |
| 9 | rs2164183 | 82317788 | ADD | 1634 | 1.13 | 1.719 | 0.08566 |
| 9 | rs10867741 | 83165767 | ADD | 1634 | 0.9461 | -0.8086 | 0.4188 |
| 9 | rs12342232 | 84611294 | ADD | 1628 | 1.12 | 1.108 | 0.2681 |
| 9 | rs4322086 | 84977538 | ADD | 1626 | 0.9823 | -0.2449 | 0.8065 |
| 9 | rs7021722 | 86398239 | ADD | 1629 | 0.9237 | -1.143 | 0.2531 |
| 9 | rs7855888 | 86641878 | ADD | 1637 | 1.063 | 0.8182 | 0.4133 |
| 9 | rs1576728 | 88232239 | ADD | 1635 | 0.8924 | -1.068 | 0.2855 |
| 9 | rs4405007 | 91290624 | ADD | 1637 | 1.004 | 0.05233 | 0.9583 |
| 9 | rs6479457 | 94950329 | ADD | 1638 | 1.101 | 1.276 | 0.2019 |
| 9 | rs10821077 | 94957073 | ADD | 1626 | 1.049 | 0.4587 | 0.6465 |
| 9 | rs7860361 | 97187625 | ADD | 1581 | 0.7971 | -2.689 | 0.007157 |
| 9 | rs16910396 | 97773603 | ADD | 1616 | 1.097 | 0.9008 | 0.3677 |
| 9 | rs2296812 | 98658902 | ADD | 1628 | 1.002 | 0.01978 | 0.9842 |
| 9 | rs10982444 | 99310019 | ADD | 1620 | 1.051 | 0.7055 | 0.4805 |
| 9 | rs2778913 | 100419601 | ADD | 1636 | 1.148 | 1.377 | 0.1685 |
| 9 | rs7044631 | 105023359 | ADD | 1592 | 0.9365 | -0.7436 | 0.4571 |
| 9 | rs2808374 | 109026900 | ADD | 1631 | 1.121 | 1.39 | 0.1647 |
| 9 | rs817852 | 109146043 | ADD | 1638 | 0.8637 | -1.28 | 0.2007 |
| 9 | rs4979219 | 109463841 | ADD | 1611 | 1.171 | 1.52 | 0.1286 |
| 9 | rs10816658 | 110113929 | ADD | 1626 | 1.011 | 0.1382 | 0.8901 |
| 9 | rs7852886 | 111406265 | ADD | 1636 | 1.016 | 0.2108 | 0.833 |
| 9 | rs2068439 | 111572497 | ADD | 1602 | 1.35 | 1.459 | 0.1444 |
| 9 | rs16914997 | 112211083 | ADD | 1626 | 0.8856 | -1.333 | 0.1826 |
| 9 | rs4460449 | 112346950 | ADD | 1602 | 1.04 | 0.4354 | 0.6633 |
| 9 | rs9299191 | 112904366 | ADD | 1638 | 1.035 | 0.4649 | 0.642 |
| 9 | rs7853673 | 115751062 | ADD | 1628 | 0.9129 | -1.273 | 0.2031 |
| 9 | rs10984339 | 120758935 | ADD | 1637 | 0.9031 | -0.8559 | 0.3921 |
| 9 | rs942152 | 122991506 | ADD | 1636 | 1.14 | 1.813 | 0.06978 |
| 9 | rs10739602 | 123750049 | ADD | 1634 | 0.9034 | -1.421 | 0.1553 |
| 9 | rs13283472 | 124145611 | ADD | 1636 | 1.089 | 0.7281 | 0.4666 |
| 9 | rs1536929 | 124431190 | ADD | 1568 | 1.088 | 1.076 | 0.2817 |
| 9 | rs3780591 | 126661465 | ADD | 1638 | 1.087 | 0.8361 | 0.4031 |
| 9 | rs1536960 | 127977496 | ADD | 1636 | 1.052 | 0.6525 | 0.5141 |
| 9 | rs7026497 | 134072800 | ADD | 1636 | 0.9681 | -0.4412 | 0.6591 |
| 9 | rs1999121 | 134592548 | ADD | 1638 | 1.057 | 0.6544 | 0.5129 |
| 9 | rs13290953 | 137026781 | ADD | 1638 | 0.9411 | -0.8442 | 0.3986 |
| 9 | rs11793385 | 138727787 | ADD | 1599 | 0.892 | -1.317 | 0.1877 |
| 10 | rs3123247 | 300225 | ADD | 1630 | 1.131 | 0.7988 | 0.4244 |
| 10 | rs3793735 | 1272828 | ADD | 1638 | 0.8623 | -1.897 | 0.0578 |
| 10 | rs4880516 | 1585155 | ADD | 1633 | 1.012 | 0.1706 | 0.8646 |
| 10 | rs7087615 | 2363295 | ADD | 1636 | 0.9261 | -0.865 | 0.3871 |
| 10 | rs7080649 | 4721081 | ADD | 1634 | 0.9486 | -0.7316 | 0.4644 |
| 10 | rs11599872 | 6258285 | ADD | 1619 | 1.118 | 0.7627 | 0.4456 |
| 10 | rs353206 | 6824006 | ADD | 1638 | 1 | 9.906e-05 | 0.9999 |
| 10 | rs413721 | 6836085 | ADD | 1624 | 1.075 | 0.9925 | 0.3209 |
| 10 | rs4146431 | 7056070 | ADD | 1563 | 1.055 | 0.7257 | 0.468 |
| 10 | rs7917456 | 7772329 | ADD | 1638 | 0.9807 | -0.2738 | 0.7843 |
| 10 | rs4750216 | 12531353 | ADD | 1638 | 1.061 | 0.6706 | 0.5025 |
| 10 | rs10508474 | 14571536 | ADD | 1638 | 1.01 | 0.09886 | 0.9212 |
| 10 | rs2257172 | 14817591 | ADD | 1634 | 0.9553 | -0.6181 | 0.5365 |
| 10 | rs17138545 | 16482944 | ADD | 1636 | 0.7561 | -1.498 | 0.1341 |
| 10 | rs10508512 | 16572580 | ADD | 1624 | 0.9438 | -0.4984 | 0.6182 |
| 10 | rs2497816 | 18405430 | ADD | 1633 | 1.145 | 1.493 | 0.1354 |
| 10 | rs7090118 | 18499288 | ADD | 1610 | 1.174 | 2.053 | 0.04009 |
| 10 | rs1779229 | 18569425 | ADD | 1638 | 1.095 | 1.17 | 0.2419 |
| 10 | rs12254400 | 18595516 | ADD | 1638 | 0.9837 | -0.1618 | 0.8715 |
| 10 | rs10764712 | 19047928 | ADD | 1638 | 0.9377 | -0.9035 | 0.3663 |
| 10 | rs11007642 | 19244248 | ADD | 1635 | 1.086 | 0.9706 | 0.3318 |
| 10 | rs9417900 | 19484188 | ADD | 1637 | 0.9398 | -0.7981 | 0.4248 |
| 10 | rs10827926 | 20345790 | ADD | 1638 | 1.036 | 0.4638 | 0.6428 |
| 10 | rs12573031 | 20958351 | ADD | 1619 | 0.9789 | -0.2822 | 0.7778 |
| 10 | rs7090625 | 23464283 | ADD | 1638 | 1.027 | 0.3029 | 0.7619 |
| 10 | rs4749165 | 26915072 | ADD | 1634 | 0.995 | -0.06254 | 0.9501 |
| 10 | rs6482566 | 26916278 | ADD | 1636 | 0.9492 | -0.7077 | 0.4791 |
| 10 | rs4747610 | 28338694 | ADD | 1636 | 0.965 | -0.4928 | 0.6221 |
| 10 | rs11007352 | 29330964 | ADD | 1621 | 1.315 | 1.77 | 0.07677 |
| 10 | rs2788453 | 29501170 | ADD | 1573 | 0.8711 | -0.8622 | 0.3886 |
| 10 | rs2532750 | 29508235 | ADD | 1638 | 1.032 | 0.2294 | 0.8186 |
| 10 | rs16929898 | 29530715 | ADD | 1637 | 1.586 | 2.423 | 0.0154 |
| 10 | rs1571957 | 30509940 | ADD | 1637 | 0.9796 | -0.2689 | 0.788 |
| 10 | rs8176984 | 30771666 | ADD | 1630 | 1.254 | 2.226 | 0.02602 |
| 10 | rs2492448 | 35235412 | ADD | 1625 | 1.016 | 0.1984 | 0.8427 |
| 10 | rs11597065 | 35252155 | ADD | 1630 | 1.068 | 0.804 | 0.4214 |
| 10 | rs702371 | 42558251 | ADD | 1629 | 1.002 | 0.02775 | 0.9779 |
| 10 | rs17154903 | 43839414 | ADD | 1638 | 0.6466 | -1.82 | 0.06874 |
| 10 | rs10900135 | 44766797 | ADD | 1635 | 1.016 | 0.1635 | 0.8701 |
| 10 | rs12412647 | 49654308 | ADD | 1625 | 0.855 | -1.089 | 0.2759 |
| 10 | rs1917805 | 50475989 | ADD | 1638 | 1.024 | 0.3458 | 0.7295 |
| 10 | rs11004610 | 56298295 | ADD | 1628 | 1.066 | 0.8848 | 0.3763 |
| 10 | rs1873800 | 58035421 | ADD | 1637 | 1.028 | 0.376 | 0.7069 |
| 10 | rs16916871 | 63357998 | ADD | 1628 | 1.74 | 1.644 | 0.1002 |
| 10 | rs10509195 | 65193372 | ADD | 1638 | 1.046 | 0.6153 | 0.5384 |
| 10 | rs12779355 | 67998550 | ADD | 1637 | 1.007 | 0.1022 | 0.9186 |
| 10 | rs10997242 | 68006301 | ADD | 1633 | 1.122 | 1.18 | 0.2378 |
| 10 | rs10762337 | 71526637 | ADD | 1638 | 1.061 | 0.7606 | 0.4469 |
| 10 | rs10823706 | 72678863 | ADD | 1638 | 0.9923 | -0.1062 | 0.9154 |
| 10 | rs10823772 | 72934436 | ADD | 1619 | 0.8971 | -1.304 | 0.1921 |
| 10 | rs11599279 | 72965347 | ADD | 1561 | 1.006 | 0.0695 | 0.9446 |
| 10 | rs11001296 | 76543433 | ADD | 1636 | 1.022 | 0.2699 | 0.7872 |
| 10 | rs11001539 | 77324931 | ADD | 1633 | 0.8864 | -0.605 | 0.5452 |
| 10 | rs1907321 | 77944394 | ADD | 1636 | 0.8151 | -1.104 | 0.2697 |
| 10 | rs16933641 | 77967980 | ADD | 1636 | 1.081 | 0.5398 | 0.5893 |
| 10 | rs17099339 | 83822284 | ADD | 1628 | 1.081 | 0.2973 | 0.7662 |
| 10 | rs10884217 | 83862680 | ADD | 1573 | 0.9269 | -0.7557 | 0.4498 |
| 10 | rs10885239 | 84460638 | ADD | 1617 | 0.8252 | -2.287 | 0.02221 |
| 10 | rs2483307 | 84554107 | ADD | 1610 | 1.031 | 0.423 | 0.6723 |
| 10 | rs7096107 | 88837965 | ADD | 1633 | 0.9546 | -0.6269 | 0.5307 |
| 10 | rs1441734 | 90706817 | ADD | 1637 | 1.045 | 0.6105 | 0.5415 |
| 10 | rs10887936 | 91007976 | ADD | 1638 | 0.9487 | -0.7163 | 0.4738 |
| 10 | rs160052 | 91185060 | ADD | 1626 | 1.049 | 0.5723 | 0.5671 |
| 10 | rs2800156 | 92431317 | ADD | 1638 | 1.094 | 1.195 | 0.2321 |
| 10 | rs11187820 | 95973043 | ADD | 1623 | 0.9623 | -0.5292 | 0.5966 |
| 10 | rs7081796 | 99194516 | ADD | 1637 | 0.9768 | -0.3293 | 0.7419 |
| 10 | rs2297643 | 99349977 | ADD | 1600 | 1.037 | 0.3577 | 0.7206 |
| 10 | rs7091572 | 101317841 | ADD | 1636 | 1.09 | 1.097 | 0.2727 |
| 10 | rs2111326 | 101352526 | ADD | 1617 | 0.9559 | -0.6241 | 0.5325 |
| 10 | rs7068079 | 101357305 | ADD | 1615 | 1.09 | 1.151 | 0.2499 |
| 10 | rs2269196 | 101475081 | ADD | 1638 | 0.968 | -0.3835 | 0.7013 |
| 10 | rs10883553 | 102625465 | ADD | 1627 | 1.039 | 0.5263 | 0.5986 |
| 10 | rs1660985 | 109259862 | ADD | 1610 | 1.2 | 1.13 | 0.2586 |
| 10 | rs2419840 | 115322088 | ADD | 1636 | 0.9776 | -0.307 | 0.7588 |
| 10 | rs3981351 | 115505110 | ADD | 1638 | 0.9444 | -0.7113 | 0.4769 |
| 10 | rs758577 | 118037288 | ADD | 1638 | 0.985 | -0.1698 | 0.8652 |
| 10 | rs1006286 | 119509000 | ADD | 1637 | 1.005 | 0.03925 | 0.9687 |
| 10 | rs7904164 | 119613646 | ADD | 1637 | 0.9999 | -0.001424 | 0.9989 |
| 10 | rs11199302 | 122127705 | ADD | 1614 | 1.057 | 0.3661 | 0.7143 |
| 10 | rs978854 | 122292028 | ADD | 1635 | 0.9447 | -0.7023 | 0.4825 |
| 10 | rs3816785 | 122336333 | ADD | 1629 | 0.9178 | -0.952 | 0.3411 |
| 10 | rs7906032 | 122710299 | ADD | 1636 | 0.9326 | -0.6094 | 0.5423 |
| 10 | rs10887110 | 123997563 | ADD | 1631 | 0.9002 | -1.438 | 0.1504 |
| 10 | rs11597044 | 125210246 | ADD | 1606 | 1.063 | 0.5553 | 0.5787 |
| 10 | rs7093782 | 128239728 | ADD | 1561 | 0.972 | -0.2263 | 0.8209 |
| 10 | rs556327 | 131637695 | ADD | 1638 | 0.8655 | -1.506 | 0.132 |
| 10 | rs611788 | 131729512 | ADD | 1635 | 0.9705 | -0.4196 | 0.6748 |
| 10 | rs1536090 | 131731159 | ADD | 1638 | 0.9839 | -0.2253 | 0.8217 |
| 10 | rs647216 | 131740881 | ADD | 1638 | 0.9419 | -0.8072 | 0.4195 |
| 10 | rs7917753 | 132358402 | ADD | 1638 | 1.021 | 0.2174 | 0.8279 |
| 10 | rs7070294 | 132780593 | ADD | 1588 | 1.033 | 0.3705 | 0.711 |
| 10 | rs4880335 | 133776155 | ADD | 1557 | 0.9351 | -0.7676 | 0.4427 |
| 11 | rs9666537 | 2642440 | ADD | 1615 | 1.039 | 0.4694 | 0.6388 |
| 11 | rs231841 | 2680180 | ADD | 1638 | 0.997 | -0.0423 | 0.9663 |
| 11 | rs3987740 | 2852376 | ADD | 1638 | 1.012 | 0.1479 | 0.8824 |
| 11 | rs11024437 | 2865232 | ADD | 1633 | 0.9101 | -0.7418 | 0.4582 |
| 11 | rs12286769 | 4423445 | ADD | 1638 | 1.026 | 0.3416 | 0.7326 |
| 11 | rs12574901 | 4634085 | ADD | 1629 | 0.9214 | -0.9618 | 0.3362 |
| 11 | rs1378738 | 4948858 | ADD | 1632 | 1.032 | 0.427 | 0.6694 |
| 11 | rs7946653 | 7394175 | ADD | 1636 | 0.9399 | -0.8462 | 0.3974 |
| 11 | rs1528125 | 8156792 | ADD | 1615 | 1.142 | 1.809 | 0.07044 |
| 11 | rs4441010 | 11225662 | ADD | 1604 | 1.059 | 0.7015 | 0.483 |
| 11 | rs11021911 | 11514149 | ADD | 1561 | 0.5146 | -1.909 | 0.05621 |
| 11 | rs10765863 | 11532033 | ADD | 1632 | 0.8462 | -1.679 | 0.09306 |
| 11 | rs12804449 | 12035502 | ADD | 1631 | 0.9567 | -0.4669 | 0.6406 |
| 11 | rs10832032 | 13367920 | ADD | 1637 | 0.9289 | -1.026 | 0.3048 |
| 11 | rs1065024 | 15945926 | ADD | 1636 | 1.052 | 0.5784 | 0.563 |
| 11 | rs2468802 | 18182318 | ADD | 1638 | 1.088 | 1.118 | 0.2636 |
| 11 | rs4757741 | 18939210 | ADD | 1637 | 0.9537 | -0.666 | 0.5054 |
| 11 | rs7102888 | 19162556 | ADD | 1619 | 0.8987 | -1.331 | 0.1833 |
| 11 | rs924693 | 19200953 | ADD | 1633 | 0.9999 | -0.001681 | 0.9987 |
| 11 | rs10834123 | 23497068 | ADD | 1636 | 0.9953 | -0.06228 | 0.9503 |
| 11 | rs7925363 | 23540943 | ADD | 1626 | 0.8752 | -1.867 | 0.06187 |
| 11 | rs10834971 | 26337381 | ADD | 1625 | 0.9526 | -0.4101 | 0.6817 |
| 11 | rs4755454 | 32859839 | ADD | 1631 | 0.9048 | -1.392 | 0.164 |
| 11 | rs4756077 | 33864531 | ADD | 1622 | 0.9105 | -1.106 | 0.2687 |
| 11 | rs3751076 | 34623426 | ADD | 1619 | 0.9609 | -0.4407 | 0.6594 |
| 11 | rs695121 | 35498041 | ADD | 1636 | 1.063 | 0.8264 | 0.4086 |
| 11 | rs10128679 | 35528490 | ADD | 1638 | 0.9801 | -0.2115 | 0.8325 |
| 11 | rs1374494 | 35550147 | ADD | 1638 | 1.232 | 0.9111 | 0.3622 |
| 11 | rs11033288 | 35801506 | ADD | 1632 | 1.065 | 0.8203 | 0.412 |
| 11 | rs10836489 | 36073916 | ADD | 1636 | 1.062 | 0.5857 | 0.5581 |
| 11 | rs10732499 | 44646657 | ADD | 1636 | 1.065 | 0.8531 | 0.3936 |
| 11 | rs7929114 | 45662738 | ADD | 1638 | 0.9779 | -0.311 | 0.7558 |
| 11 | rs1125082 | 46102457 | ADD | 1635 | 0.9555 | -0.5515 | 0.5813 |
| 11 | rs10897061 | 59955622 | ADD | 1636 | 1.035 | 0.4236 | 0.6719 |
| 11 | rs2120182 | 59968873 | ADD | 1632 | 0.9662 | -0.4362 | 0.6627 |
| 11 | rs524386 | 64341535 | ADD | 1624 | 1.018 | 0.1636 | 0.87 |
| 11 | rs10896050 | 65334092 | ADD | 1634 | 1.023 | 0.2539 | 0.7996 |
| 11 | rs11227673 | 66589104 | ADD | 1638 | 0.9002 | -1.504 | 0.1327 |
| 11 | rs3794186 | 67577612 | ADD | 1599 | 0.9431 | -0.4212 | 0.6736 |
| 11 | rs10896396 | 68561382 | ADD | 1638 | 0.887 | -1.449 | 0.1474 |
| 11 | rs879380 | 70697060 | ADD | 1623 | 1.027 | 0.3502 | 0.7262 |
| 11 | rs1660832 | 70725472 | ADD | 1627 | 1.134 | 1.429 | 0.153 |
| 11 | rs10897992 | 70728576 | ADD | 1638 | 0.9676 | -0.296 | 0.7672 |
| 11 | rs10899345 | 76425167 | ADD | 1638 | 1.168 | 2.144 | 0.03202 |
| 11 | rs1369458 | 78438121 | ADD | 1636 | 1.131 | 1.132 | 0.2577 |
| 11 | rs518556 | 78708426 | ADD | 1588 | 1.026 | 0.3509 | 0.7257 |
| 11 | rs489257 | 78768875 | ADD | 1636 | 0.8153 | -2.72 | 0.006523 |
| 11 | rs2512483 | 79772626 | ADD | 1638 | 1.028 | 0.3369 | 0.7362 |
| 11 | rs1608169 | 80428536 | ADD | 1634 | 1.068 | 0.867 | 0.3859 |
| 11 | rs10898563 | 86336861 | ADD | 1634 | 0.9639 | -0.484 | 0.6284 |
| 11 | rs4488224 | 86899478 | ADD | 1630 | 1.084 | 1.041 | 0.2978 |
| 11 | rs4310643 | 87354717 | ADD | 1638 | 0.9934 | -0.07204 | 0.9426 |
| 11 | rs2045459 | 92845829 | ADD | 1629 | 1.097 | 1.246 | 0.2128 |
| 11 | rs2608280 | 92849120 | ADD | 1620 | 0.9237 | -1.092 | 0.2749 |
| 11 | rs2252326 | 92886849 | ADD | 1630 | 0.833 | -1.443 | 0.1489 |
| 11 | rs12789145 | 94047384 | ADD | 1638 | 0.9764 | -0.2002 | 0.8413 |
| 11 | rs11021055 | 94529322 | ADD | 1636 | 1.163 | 1.147 | 0.2514 |
| 11 | rs7115578 | 95639748 | ADD | 1618 | 1.105 | 1.348 | 0.1778 |
| 11 | rs12224719 | 97681490 | ADD | 1609 | 0.9616 | -0.5552 | 0.5788 |
| 11 | rs11212509 | 107461981 | ADD | 1633 | 0.9702 | -0.3351 | 0.7375 |
| 11 | rs4938535 | 110780460 | ADD | 1616 | 0.9444 | -0.6892 | 0.4907 |
| 11 | rs4245155 | 112962534 | ADD | 1634 | 1.019 | 0.2647 | 0.7913 |
| 11 | rs1013416 | 114725464 | ADD | 1636 | 1.105 | 1.202 | 0.2293 |
| 11 | rs542671 | 115828211 | ADD | 1563 | 0.8561 | -1.625 | 0.1042 |
| 11 | rs236919 | 116600571 | ADD | 1637 | 1.202 | 2.617 | 0.00888 |
| 11 | rs1940037 | 116941305 | ADD | 1637 | 1.118 | 1.393 | 0.1637 |
| 11 | rs12790243 | 117183753 | ADD | 1637 | 0.9994 | -0.008669 | 0.9931 |
| 11 | rs17092876 | 119903853 | ADD | 1637 | 1.055 | 0.716 | 0.474 |
| 11 | rs10502260 | 121073107 | ADD | 1636 | 1.075 | 0.8635 | 0.3879 |
| 11 | rs531743 | 121625352 | ADD | 1638 | 1.172 | 1.216 | 0.2239 |
| 11 | rs7103798 | 123651252 | ADD | 1637 | 1.024 | 0.3159 | 0.752 |
| 11 | rs3924535 | 124305106 | ADD | 1637 | 0.9171 | -0.6374 | 0.5239 |
| 11 | rs863955 | 124757151 | ADD | 1636 | 1.086 | 1.093 | 0.2743 |
| 11 | rs509759 | 126028925 | ADD | 1636 | 0.933 | -0.9398 | 0.3473 |
| 11 | rs693253 | 126032975 | ADD | 1627 | 1.007 | 0.09838 | 0.9216 |
| 11 | rs613587 | 128138519 | ADD | 1637 | 0.9811 | -0.2403 | 0.8101 |
| 11 | rs4936105 | 129857755 | ADD | 1618 | 0.9951 | -0.06517 | 0.948 |
| 11 | rs1945921 | 130467592 | ADD | 1626 | 0.9731 | -0.3833 | 0.7015 |
| 11 | rs10894396 | 130831245 | ADD | 1629 | 1.081 | 1.025 | 0.3056 |
| 11 | rs12276840 | 131853608 | ADD | 1628 | 1.04 | 0.5374 | 0.591 |
| 11 | rs528190 | 132549590 | ADD | 1607 | 0.979 | -0.2436 | 0.8075 |
| 11 | rs4937775 | 132822925 | ADD | 1636 | 1.109 | 1.481 | 0.1386 |
| 11 | rs10791373 | 133853184 | ADD | 1588 | 1.108 | 1.166 | 0.2434 |
| 11 | rs4937919 | 133970430 | ADD | 1601 | 1.053 | 0.5222 | 0.6016 |
| 11 | rs11223936 | 134054023 | ADD | 1588 | 0.8835 | -1.689 | 0.09125 |
| 12 | rs2429142 | 2004228 | ADD | 1638 | 0.8857 | -1.461 | 0.144 |
| 12 | rs2370411 | 2152186 | ADD | 1637 | 0.9008 | -1.222 | 0.2215 |
| 12 | rs2370413 | 2225131 | ADD | 1614 | 1.109 | 1.484 | 0.1378 |
| 12 | rs11062658 | 3439663 | ADD | 1638 | 0.872 | -1.514 | 0.1301 |
| 12 | rs2532560 | 4032132 | ADD | 1638 | 1.202 | 2.002 | 0.04527 |
| 12 | rs6489530 | 4229392 | ADD | 1604 | 1.136 | 0.6291 | 0.5293 |
| 12 | rs1860958 | 5760433 | ADD | 1635 | 0.9873 | -0.1243 | 0.9011 |
| 12 | rs10849439 | 6285592 | ADD | 1584 | 0.9613 | -0.4526 | 0.6509 |
| 12 | rs7306824 | 7419738 | ADD | 1638 | 1.027 | 0.3597 | 0.7191 |
| 12 | rs7973596 | 7709039 | ADD | 1638 | 1.045 | 0.3854 | 0.6999 |
| 12 | rs2724610 | 11737633 | ADD | 1625 | 0.9649 | -0.5093 | 0.6106 |
| 12 | rs1894307 | 11896987 | ADD | 1634 | 0.9917 | -0.08237 | 0.9344 |
| 12 | rs3825271 | 13107338 | ADD | 1638 | 1.149 | 0.7297 | 0.4656 |
| 12 | rs4578488 | 13316196 | ADD | 1627 | 0.9211 | -1.124 | 0.261 |
| 12 | rs1514839 | 19096918 | ADD | 1636 | 1.151 | 1.709 | 0.0875 |
| 12 | rs12815398 | 19115452 | ADD | 1636 | 1.007 | 0.06231 | 0.9503 |
| 12 | rs10734694 | 19980066 | ADD | 1631 | 1.014 | 0.187 | 0.8517 |
| 12 | rs7301416 | 22847404 | ADD | 1618 | 0.93 | -0.5895 | 0.5555 |
| 12 | rs11047026 | 23697144 | ADD | 1638 | 0.9065 | -1.268 | 0.2048 |
| 12 | rs4963751 | 24351479 | ADD | 1571 | 0.849 | -1.533 | 0.1252 |
| 12 | rs1011185 | 25514998 | ADD | 1634 | 0.9159 | -1.179 | 0.2385 |
| 12 | rs10842682 | 26197489 | ADD | 1635 | 0.9644 | -0.5271 | 0.5981 |
| 12 | rs12823875 | 29701392 | ADD | 1628 | 1.001 | 0.01792 | 0.9857 |
| 12 | rs6487842 | 29709471 | ADD | 1633 | 1.01 | 0.1319 | 0.8951 |
| 12 | rs7972685 | 29872973 | ADD | 1637 | 0.8873 | -1.149 | 0.2505 |
| 12 | rs2046383 | 29995409 | ADD | 1634 | 1.163 | 1.906 | 0.05661 |
| 12 | rs2075364 | 30997917 | ADD | 1638 | 0.9038 | -0.5762 | 0.5645 |
| 12 | rs11177589 | 39327131 | ADD | 1637 | 1.144 | 1.624 | 0.1044 |
| 12 | rs10491998 | 40585149 | ADD | 1569 | 1.054 | 0.4655 | 0.6416 |
| 12 | rs12297983 | 41451988 | ADD | 1622 | 1.108 | 0.9824 | 0.3259 |
| 12 | rs7297927 | 46057101 | ADD | 1619 | 1.042 | 0.5488 | 0.5832 |
| 12 | rs2272485 | 50772158 | ADD | 1560 | 1.065 | 0.7614 | 0.4464 |
| 12 | rs1791643 | 51028003 | ADD | 1573 | 1.079 | 1.064 | 0.2872 |
| 12 | rs928994 | 51087563 | ADD | 1637 | 1.08 | 0.9648 | 0.3346 |
| 12 | rs7133633 | 52426199 | ADD | 1638 | 0.9618 | -0.5106 | 0.6097 |
| 12 | rs11171007 | 53339810 | ADD | 1638 | 0.9886 | -0.1423 | 0.8868 |
| 12 | rs4533076 | 54355498 | ADD | 1638 | 1.046 | 0.5648 | 0.5722 |
| 12 | rs3816804 | 54967012 | ADD | 1616 | 1.126 | 0.8375 | 0.4023 |
| 12 | rs7302068 | 60253353 | ADD | 1630 | 0.9467 | -0.5397 | 0.5894 |
| 12 | rs4547160 | 61789917 | ADD | 1621 | 0.9188 | -1.116 | 0.2645 |
| 12 | rs3741738 | 63672686 | ADD | 1637 | 0.9779 | -0.3134 | 0.754 |
| 12 | rs11176078 | 64861524 | ADD | 1636 | 0.8563 | -1.929 | 0.05377 |
| 12 | rs2127536 | 67030718 | ADD | 1637 | 0.8846 | -1.575 | 0.1151 |
| 12 | rs2035966 | 68496359 | ADD | 1632 | 0.8916 | -1.534 | 0.125 |
| 12 | rs2584021 | 69316000 | ADD | 1635 | 1.169 | 1.37 | 0.1707 |
| 12 | rs7298378 | 69360651 | ADD | 1625 | 1.021 | 0.2016 | 0.8402 |
| 12 | rs11178820 | 70156158 | ADD | 1638 | 0.9072 | -1.255 | 0.2094 |
| 12 | rs10784924 | 70213514 | ADD | 1638 | 0.9244 | -0.5626 | 0.5737 |
| 12 | rs1843809 | 70634965 | ADD | 1578 | 0.9996 | -0.004694 | 0.9963 |
| 12 | rs1386483 | 70698761 | ADD | 1623 | 1.051 | 0.6916 | 0.4892 |
| 12 | rs7307780 | 74506885 | ADD | 1628 | 0.9214 | -0.8071 | 0.4196 |
| 12 | rs7314267 | 74578155 | ADD | 1637 | 0.9134 | -0.875 | 0.3816 |
| 12 | rs1520798 | 76136716 | ADD | 1634 | 1.141 | 1.715 | 0.08629 |
| 12 | rs6539534 | 79775075 | ADD | 1638 | 0.9814 | -0.2597 | 0.7951 |
| 12 | rs7310349 | 79786689 | ADD | 1634 | 1.05 | 0.6429 | 0.5203 |
| 12 | rs12828169 | 79874850 | ADD | 1638 | 0.9451 | -0.4009 | 0.6885 |
| 12 | rs12580477 | 80760658 | ADD | 1631 | 1.181 | 1.954 | 0.05071 |
| 12 | rs10862940 | 83759289 | ADD | 1571 | 0.9515 | -0.4478 | 0.6543 |
| 12 | rs7297281 | 91388499 | ADD | 1638 | 0.9643 | -0.5154 | 0.6063 |
| 12 | rs7969076 | 92620173 | ADD | 1636 | 0.8841 | -1.705 | 0.08819 |
| 12 | rs10859569 | 92655442 | ADD | 1637 | 1.056 | 0.7446 | 0.4565 |
| 12 | rs7302717 | 93643947 | ADD | 1637 | 1.074 | 0.8682 | 0.3853 |
| 12 | rs1316566 | 93647288 | ADD | 1638 | 1.079 | 1.036 | 0.3004 |
| 12 | rs7972350 | 93684736 | ADD | 1627 | 0.947 | -0.7 | 0.484 |
| 12 | rs7133053 | 94713065 | ADD | 1617 | 0.9521 | -0.6804 | 0.4963 |
| 12 | rs17027262 | 96558094 | ADD | 1633 | 1.128 | 0.7539 | 0.4509 |
| 12 | rs7975512 | 97323500 | ADD | 1638 | 0.979 | -0.2938 | 0.7689 |
| 12 | rs249842 | 97395488 | ADD | 1638 | 1.168 | 1.787 | 0.07394 |
| 12 | rs733327 | 100417446 | ADD | 1612 | 0.9886 | -0.1606 | 0.8724 |
| 12 | rs4255604 | 103097845 | ADD | 1635 | 0.873 | -1.07 | 0.2848 |
| 12 | rs835481 | 103573044 | ADD | 1631 | 1.07 | 0.8586 | 0.3906 |
| 12 | rs7956015 | 103577995 | ADD | 1554 | 1.033 | 0.4277 | 0.6688 |
| 12 | rs2434081 | 105052584 | ADD | 1638 | 1.117 | 1.484 | 0.1378 |
| 12 | rs2162316 | 106281133 | ADD | 1636 | 0.9226 | -0.9292 | 0.3528 |
| 12 | rs2075263 | 108188708 | ADD | 1637 | 1.017 | 0.1769 | 0.8596 |
| 12 | rs249047 | 112562237 | ADD | 1637 | 0.9455 | -0.7819 | 0.4343 |
| 12 | rs11067772 | 114712808 | ADD | 1637 | 0.9736 | -0.3508 | 0.7257 |
| 12 | rs1715586 | 114712969 | ADD | 1636 | 1.027 | 0.3012 | 0.7633 |
| 12 | rs2575286 | 114782996 | ADD | 1637 | 1.04 | 0.3481 | 0.7278 |
| 12 | rs605265 | 116620279 | ADD | 1631 | 1.25 | 3.038 | 0.00238 |
| 12 | rs604356 | 116620471 | ADD | 1613 | 1.22 | 2.077 | 0.03783 |
| 12 | rs4767654 | 116951936 | ADD | 1634 | 1.021 | 0.286 | 0.7749 |
| 12 | rs7309145 | 117821487 | ADD | 1593 | 0.8867 | -1.235 | 0.2167 |
| 12 | rs2204498 | 117846555 | ADD | 1638 | 0.9766 | -0.332 | 0.7399 |
| 12 | rs10847980 | 121953875 | ADD | 1626 | 1.181 | 1.433 | 0.1518 |
| 12 | rs7953077 | 124156215 | ADD | 1637 | 0.9804 | -0.2643 | 0.7915 |
| 12 | rs225476 | 125031349 | ADD | 1638 | 1.03 | 0.3694 | 0.7118 |
| 12 | rs10847353 | 126521139 | ADD | 1637 | 0.9231 | -1.119 | 0.2631 |
| 12 | rs1021831 | 126707469 | ADD | 1635 | 0.9819 | -0.2148 | 0.8299 |
| 12 | rs7316076 | 127112932 | ADD | 1638 | 1.128 | 1.336 | 0.1814 |
| 12 | rs9705571 | 127145511 | ADD | 1637 | 1.021 | 0.2838 | 0.7766 |
| 12 | rs1486634 | 128241338 | ADD | 1635 | 0.8931 | -1.087 | 0.2768 |
| 12 | rs10773613 | 128266650 | ADD | 1635 | 1.112 | 1.431 | 0.1524 |
| 12 | rs11060784 | 129272960 | ADD | 1638 | 1.006 | 0.03238 | 0.9742 |
| 13 | rs7995321 | 19822535 | ADD | 1637 | 1.056 | 0.6952 | 0.4869 |
| 13 | rs2315540 | 20814905 | ADD | 1635 | 1.078 | 1.017 | 0.3091 |
| 13 | rs4769191 | 21547069 | ADD | 1618 | 1.091 | 0.5562 | 0.5781 |
| 13 | rs11838655 | 21593311 | ADD | 1635 | 1.087 | 1.111 | 0.2665 |
| 13 | rs7331655 | 21684026 | ADD | 1634 | 0.8868 | -1.662 | 0.0966 |
| 13 | rs2503340 | 21708964 | ADD | 1602 | 0.8879 | -1.321 | 0.1864 |
| 13 | rs9317292 | 22392319 | ADD | 1558 | 1.148 | 1.5 | 0.1336 |
| 13 | rs17078394 | 22651669 | ADD | 1638 | 1.355 | 3.002 | 0.002681 |
| 13 | rs9508556 | 29278407 | ADD | 1632 | 1.159 | 1.371 | 0.1705 |
| 13 | rs4141774 | 29389900 | ADD | 1638 | 0.8946 | -0.815 | 0.4151 |
| 13 | rs4254165 | 30215878 | ADD | 1637 | 1.051 | 0.6462 | 0.5182 |
| 13 | rs4769065 | 30270019 | ADD | 1638 | 0.9683 | -0.3719 | 0.71 |
| 13 | rs8001778 | 30331110 | ADD | 1598 | 1.037 | 0.349 | 0.7271 |
| 13 | rs648202 | 32533463 | ADD | 1635 | 1.06 | 0.6675 | 0.5044 |
| 13 | rs9540823 | 33895343 | ADD | 1637 | 1.046 | 0.6428 | 0.5203 |
| 13 | rs11618012 | 34237417 | ADD | 1638 | 1.279 | 2.6 | 0.009312 |
| 13 | rs9315310 | 34249431 | ADD | 1570 | 1.21 | 2.112 | 0.0347 |
| 13 | rs2324614 | 39902048 | ADD | 1623 | 1.034 | 0.3481 | 0.7277 |
| 13 | rs1037272 | 41688458 | ADD | 1636 | 0.8692 | -1.488 | 0.1367 |
| 13 | rs9594724 | 41767119 | ADD | 1613 | 0.8365 | -2.084 | 0.03717 |
| 13 | rs3918568 | 42352448 | ADD | 1638 | 1.072 | 0.8675 | 0.3857 |
| 13 | rs930577 | 42704889 | ADD | 1637 | 1.026 | 0.2953 | 0.7678 |
| 13 | rs1410942 | 43086455 | ADD | 1629 | 0.8936 | -0.8173 | 0.4138 |
| 13 | rs1888140 | 43715707 | ADD | 1628 | 1.004 | 0.05215 | 0.9584 |
| 13 | rs1886191 | 44525809 | ADD | 1619 | 1.072 | 0.7818 | 0.4343 |
| 13 | rs1536207 | 45013732 | ADD | 1635 | 0.9477 | -0.7361 | 0.4616 |
| 13 | rs582854 | 46343878 | ADD | 1602 | 0.8743 | -1.829 | 0.06738 |
| 13 | rs2794467 | 50145211 | ADD | 1631 | 1.104 | 0.9857 | 0.3243 |
| 13 | rs1891948 | 52537146 | ADD | 1609 | 0.9186 | -1.155 | 0.248 |
| 13 | rs1317699 | 56818937 | ADD | 1581 | 0.9067 | -1.084 | 0.2785 |
| 13 | rs9527675 | 57113143 | ADD | 1632 | 0.9703 | -0.406 | 0.6847 |
| 13 | rs9538404 | 58876919 | ADD | 1623 | 0.9995 | -0.00396 | 0.9968 |
| 13 | rs4884871 | 69490705 | ADD | 1630 | 0.8633 | -1.762 | 0.07802 |
| 13 | rs1999078 | 69642883 | ADD | 1636 | 0.9294 | -0.7927 | 0.428 |
| 13 | rs9529804 | 70215962 | ADD | 1637 | 1.019 | 0.2397 | 0.8106 |
| 13 | rs7995527 | 70740432 | ADD | 1636 | 1.073 | 0.9889 | 0.3227 |
| 13 | rs17090071 | 72827429 | ADD | 1613 | 1.009 | 0.1059 | 0.9157 |
| 13 | rs9530237 | 73243361 | ADD | 1618 | 1.034 | 0.4223 | 0.6728 |
| 13 | rs7338627 | 74181492 | ADD | 1636 | 0.9857 | -0.1886 | 0.8504 |
| 13 | rs9565180 | 75129471 | ADD | 1629 | 1.098 | 1.072 | 0.2836 |
| 13 | rs1324790 | 77639935 | ADD | 1638 | 0.9679 | -0.4198 | 0.6746 |
| 13 | rs7324958 | 80560020 | ADD | 1588 | 0.9375 | -0.9007 | 0.3678 |
| 13 | rs7322093 | 83030766 | ADD | 1632 | 1.097 | 1.232 | 0.2179 |
| 13 | rs7336307 | 85808355 | ADD | 1597 | 0.956 | -0.6262 | 0.5312 |
| 13 | rs1504432 | 88980864 | ADD | 1615 | 0.985 | -0.2048 | 0.8377 |
| 13 | rs16946369 | 91098191 | ADD | 1636 | 0.9762 | -0.1909 | 0.8486 |
| 13 | rs9561336 | 92832141 | ADD | 1635 | 0.9837 | -0.1592 | 0.8735 |
| 13 | rs9524925 | 94787191 | ADD | 1635 | 0.9441 | -0.7863 | 0.4317 |
| 13 | rs7333503 | 94884481 | ADD | 1638 | 0.9288 | -1.058 | 0.2898 |
| 13 | rs16951472 | 95871978 | ADD | 1589 | 0.8749 | -1.405 | 0.1601 |
| 13 | rs7318798 | 96263426 | ADD | 1633 | 0.9861 | -0.1964 | 0.8443 |
| 13 | rs7329817 | 97371139 | ADD | 1637 | 1.007 | 0.05239 | 0.9582 |
| 13 | rs7330774 | 98048526 | ADD | 1637 | 0.9662 | -0.4202 | 0.6744 |
| 13 | rs12184508 | 99150799 | ADD | 1635 | 0.9259 | -1.101 | 0.2707 |
| 13 | rs4772352 | 100391674 | ADD | 1635 | 0.9404 | -0.7674 | 0.4429 |
| 13 | rs9554742 | 100394543 | ADD | 1638 | 0.8924 | -1.024 | 0.3057 |
| 13 | rs1898199 | 101102895 | ADD | 1628 | 1.109 | 1.337 | 0.1814 |
| 13 | rs1537197 | 103196429 | ADD | 1638 | 0.9366 | -0.6096 | 0.5421 |
| 13 | rs980728 | 104051148 | ADD | 1637 | 0.9171 | -0.9595 | 0.3373 |
| 13 | rs16968712 | 105925785 | ADD | 1638 | 0.9392 | -0.7224 | 0.47 |
| 13 | rs7335820 | 107123363 | ADD | 1631 | 1.01 | 0.1231 | 0.902 |
| 13 | rs1553705 | 107290898 | ADD | 1633 | 1.062 | 0.6893 | 0.4906 |
| 13 | rs1543002 | 107301357 | ADD | 1582 | 1.016 | 0.2164 | 0.8287 |
| 13 | rs2277424 | 110320421 | ADD | 1638 | 1.067 | 0.6382 | 0.5234 |
| 13 | rs7332528 | 110336048 | ADD | 1634 | 1.109 | 1.402 | 0.1609 |
| 13 | rs2893380 | 110373427 | ADD | 1638 | 1.086 | 0.8799 | 0.3789 |
| 13 | rs7317887 | 110455200 | ADD | 1638 | 0.9765 | -0.3255 | 0.7448 |
| 13 | rs831167 | 110593545 | ADD | 1637 | 1.149 | 1.239 | 0.2153 |
| 13 | rs1295890 | 112030830 | ADD | 1612 | 1.115 | 1.345 | 0.1787 |
| 13 | rs7986656 | 113157383 | ADD | 1624 | 1.054 | 0.5835 | 0.5596 |
| 13 | rs389862 | 113948331 | ADD | 1638 | 1.13 | 1.047 | 0.2949 |
| 14 | rs12588812 | 20374803 | ADD | 1638 | 0.917 | -0.5974 | 0.5502 |
| 14 | rs745696 | 20640113 | ADD | 1638 | 1.074 | 0.9009 | 0.3677 |
| 14 | rs7161544 | 20693488 | ADD | 1597 | 1.005 | 0.0655 | 0.9478 |
| 14 | rs994827 | 21419526 | ADD | 1623 | 1.274 | 2.19 | 0.02851 |
| 14 | rs17120578 | 22161536 | ADD | 1623 | 1.013 | 0.1683 | 0.8663 |
| 14 | rs1015089 | 22677086 | ADD | 1615 | 1.006 | 0.07996 | 0.9363 |
| 14 | rs1998055 | 22693667 | ADD | 1638 | 0.961 | -0.4164 | 0.6771 |
| 14 | rs7145877 | 23086206 | ADD | 1635 | 0.8635 | -1.382 | 0.1668 |
| 14 | rs2180818 | 25199667 | ADD | 1638 | 1.049 | 0.6919 | 0.489 |
| 14 | rs7148442 | 28353760 | ADD | 1633 | 0.966 | -0.4839 | 0.6285 |
| 14 | rs4981770 | 30265856 | ADD | 1637 | 0.9202 | -1.183 | 0.2368 |
| 14 | rs7144454 | 32721646 | ADD | 1638 | 0.9557 | -0.5891 | 0.5558 |
| 14 | rs10483489 | 36689921 | ADD | 1637 | 1.027 | 0.2435 | 0.8076 |
| 14 | rs12590869 | 42210086 | ADD | 1627 | 0.9968 | -0.0317 | 0.9747 |
| 14 | rs12888829 | 46465551 | ADD | 1635 | 0.903 | -1.23 | 0.2188 |
| 14 | rs1557191 | 51762335 | ADD | 1635 | 1.062 | 0.7818 | 0.4343 |
| 14 | rs1679804 | 55563222 | ADD | 1637 | 1.125 | 1.579 | 0.1143 |
| 14 | rs1188690 | 55868252 | ADD | 1631 | 1.062 | 0.7709 | 0.4407 |
| 14 | rs1152492 | 55869750 | ADD | 1638 | 1.048 | 0.6424 | 0.5206 |
| 14 | rs10431700 | 57211552 | ADD | 1636 | 0.9808 | -0.2636 | 0.7921 |
| 14 | rs10483695 | 57585309 | ADD | 1623 | 1.044 | 0.5922 | 0.5537 |
| 14 | rs4312236 | 61054788 | ADD | 1633 | 1.017 | 0.1532 | 0.8783 |
| 14 | rs2144112 | 62787585 | ADD | 1636 | 1.015 | 0.1289 | 0.8975 |
| 14 | rs2296316 | 64589999 | ADD | 1638 | 1.108 | 1.476 | 0.1399 |
| 14 | rs11158685 | 67112327 | ADD | 1634 | 0.9481 | -0.735 | 0.4624 |
| 14 | rs1957572 | 67806224 | ADD | 1632 | 0.9579 | -0.3576 | 0.7206 |
| 14 | rs11622335 | 75871777 | ADD | 1638 | 0.9926 | -0.1011 | 0.9194 |
| 14 | rs4344663 | 75921152 | ADD | 1594 | 0.9162 | -1.197 | 0.2312 |
| 14 | rs11159695 | 84763606 | ADD | 1633 | 0.8453 | -2.053 | 0.04008 |
| 14 | rs3850389 | 87857231 | ADD | 1598 | 1.104 | 0.694 | 0.4877 |
| 14 | rs11845781 | 88346184 | ADD | 1636 | 0.998 | -0.02835 | 0.9774 |
| 14 | rs917908 | 91433539 | ADD | 1632 | 1.03 | 0.3107 | 0.756 |
| 14 | rs1242105 | 92264079 | ADD | 1638 | 0.9962 | -0.0472 | 0.9624 |
| 14 | rs1341001 | 94432449 | ADD | 1637 | 0.9643 | -0.5102 | 0.6099 |
| 14 | rs17093029 | 95158512 | ADD | 1634 | 0.8995 | -1.081 | 0.2797 |
| 14 | rs11625059 | 96327079 | ADD | 1637 | 1.29 | 1.076 | 0.2818 |
| 14 | rs10134270 | 96841187 | ADD | 1628 | 1.127 | 1.027 | 0.3043 |
| 14 | rs1973150 | 97331822 | ADD | 1638 | 1.098 | 1.146 | 0.252 |
| 14 | rs17096688 | 97724652 | ADD | 1606 | 1.47 | 1.988 | 0.04678 |
| 14 | rs868666 | 98670272 | ADD | 1633 | 0.8728 | -1.895 | 0.05814 |
| 14 | rs1269096 | 98672872 | ADD | 1637 | 1.067 | 0.9248 | 0.355 |
| 14 | rs17098899 | 99148653 | ADD | 1636 | 1.087 | 0.6901 | 0.4901 |
| 14 | rs17098912 | 99174082 | ADD | 1634 | 1.004 | 0.03985 | 0.9682 |
| 14 | rs710100 | 102672031 | ADD | 1622 | 1.051 | 0.6725 | 0.5013 |
| 14 | rs12892765 | 102846524 | ADD | 1637 | 1.143 | 1.275 | 0.2022 |
| 15 | rs2345408 | 21278748 | ADD | 1607 | 0.9797 | -0.2775 | 0.7814 |
| 15 | rs8031166 | 21633826 | ADD | 1637 | 1.13 | 1.627 | 0.1038 |
| 15 | rs11161309 | 24230205 | ADD | 1629 | 0.9276 | -0.9836 | 0.3253 |
| 15 | rs9920483 | 24230680 | ADD | 1635 | 0.9879 | -0.1672 | 0.8672 |
| 15 | rs16950272 | 25552496 | ADD | 1637 | 1.033 | 0.4213 | 0.6735 |
| 15 | rs4780121 | 31547408 | ADD | 1606 | 0.9717 | -0.3451 | 0.73 |
| 15 | rs2070664 | 32872493 | ADD | 1633 | 1.186 | 2.297 | 0.02164 |
| 15 | rs16954301 | 33653376 | ADD | 1612 | 1.088 | 0.5802 | 0.5618 |
| 15 | rs4502172 | 34114820 | ADD | 1636 | 1.07 | 0.9391 | 0.3477 |
| 15 | rs17536109 | 34130070 | ADD | 1634 | 0.96 | -0.5603 | 0.5753 |
| 15 | rs17574546 | 36689768 | ADD | 1630 | 1.105 | 1.055 | 0.2915 |
| 15 | rs16954437 | 36914379 | ADD | 1634 | 1.106 | 0.9014 | 0.3674 |
| 15 | rs7174767 | 38057092 | ADD | 1636 | 0.9984 | -0.01149 | 0.9908 |
| 15 | rs2412716 | 40550665 | ADD | 1633 | 0.641 | -1.658 | 0.09728 |
| 15 | rs6493278 | 45517307 | ADD | 1638 | 1.008 | 0.09638 | 0.9232 |
| 15 | rs6493397 | 48139853 | ADD | 1631 | 1.056 | 0.553 | 0.5803 |
| 15 | rs16963311 | 48192467 | ADD | 1636 | 0.9041 | -0.8964 | 0.37 |
| 15 | rs12102156 | 50376391 | ADD | 1630 | 1.224 | 2.154 | 0.03125 |
| 15 | rs2431016 | 55395984 | ADD | 1617 | 1.016 | 0.1573 | 0.875 |
| 15 | rs4774943 | 55550756 | ADD | 1638 | 0.9905 | -0.1304 | 0.8962 |
| 15 | rs11636064 | 57660153 | ADD | 1637 | 0.961 | -0.5663 | 0.5712 |
| 15 | rs4775287 | 58774522 | ADD | 1629 | 0.9489 | -0.6719 | 0.5017 |
| 15 | rs7179299 | 60835719 | ADD | 1638 | 0.9845 | -0.2109 | 0.833 |
| 15 | rs6494361 | 60869556 | ADD | 1638 | 1.132 | 1.277 | 0.2017 |
| 15 | rs8036528 | 68218165 | ADD | 1637 | 0.9888 | -0.1207 | 0.9039 |
| 15 | rs16954285 | 68286152 | ADD | 1633 | 0.9524 | -0.4112 | 0.6809 |
| 15 | rs8032896 | 78358161 | ADD | 1635 | 0.9046 | -1.344 | 0.179 |
| 15 | rs925111 | 78872538 | ADD | 1638 | 1.073 | 0.9685 | 0.3328 |
| 15 | rs9806128 | 80051917 | ADD | 1636 | 1.228 | 2.39 | 0.01683 |
| 15 | rs12324755 | 80093252 | ADD | 1638 | 1.223 | 1.469 | 0.1419 |
| 15 | rs17352886 | 80140026 | ADD | 1638 | 0.9455 | -0.7713 | 0.4405 |
| 15 | rs1553883 | 81269403 | ADD | 1638 | 0.9257 | -0.854 | 0.3931 |
| 15 | rs17158132 | 81470710 | ADD | 1635 | 0.912 | -0.5961 | 0.5511 |
| 15 | rs1568657 | 81517183 | ADD | 1635 | 0.9402 | -0.6984 | 0.4849 |
| 15 | rs1431240 | 84753896 | ADD | 1628 | 1.014 | 0.09286 | 0.926 |
| 15 | rs11633969 | 85221240 | ADD | 1604 | 0.9372 | -0.8601 | 0.3897 |
| 15 | rs2881640 | 85261718 | ADD | 1638 | 0.8372 | -2.04 | 0.04133 |
| 15 | rs977133 | 85807138 | ADD | 1618 | 0.927 | -0.9943 | 0.3201 |
| 15 | rs894290 | 86625685 | ADD | 1637 | 1.023 | 0.2894 | 0.7723 |
| 15 | rs7182912 | 86839816 | ADD | 1617 | 1.18 | 1.92 | 0.05483 |
| 15 | rs953065 | 87203929 | ADD | 1638 | 1.134 | 1.591 | 0.1117 |
| 15 | rs150353 | 87729193 | ADD | 1632 | 0.8645 | -2.012 | 0.04424 |
| 15 | rs7181019 | 90252895 | ADD | 1621 | 1.128 | 1.43 | 0.1527 |
| 15 | rs4932599 | 90344382 | ADD | 1623 | 0.9418 | -0.7141 | 0.4752 |
| 15 | rs4778031 | 90532460 | ADD | 1624 | 0.9074 | -1.241 | 0.2148 |
| 15 | rs8025225 | 90741904 | ADD | 1638 | 0.9127 | -1.274 | 0.2028 |
| 15 | rs7171722 | 90887691 | ADD | 1636 | 0.9991 | -0.01031 | 0.9918 |
| 15 | rs901965 | 91767715 | ADD | 1635 | 0.9991 | -0.007038 | 0.9944 |
| 15 | rs12906594 | 93012566 | ADD | 1637 | 1.163 | 1.47 | 0.1415 |
| 15 | rs16975866 | 94295636 | ADD | 1638 | 1.068 | 0.577 | 0.5639 |
| 15 | rs16975868 | 94299720 | ADD | 1637 | 0.7692 | -1.273 | 0.2032 |
| 15 | rs2577002 | 94597264 | ADD | 1637 | 1.07 | 0.9415 | 0.3465 |
| 15 | rs4297661 | 95791348 | ADD | 1638 | 1.043 | 0.5497 | 0.5825 |
| 15 | rs11632922 | 97908966 | ADD | 1635 | 0.9131 | -1.279 | 0.2007 |
| 15 | rs4419043 | 98563203 | ADD | 1633 | 1.001 | 0.01002 | 0.992 |
| 15 | rs930847 | 99376085 | ADD | 1638 | 1.056 | 0.7057 | 0.4804 |
| 15 | rs1039037 | 99753680 | ADD | 1617 | 0.9208 | -1.115 | 0.265 |
| 16 | rs2238418 | 3815169 | ADD | 1638 | 0.6241 | -1.417 | 0.1565 |
| 16 | rs490662 | 5394487 | ADD | 1635 | 0.9867 | -0.193 | 0.847 |
| 16 | rs10500329 | 5834783 | ADD | 1638 | 0.9145 | -1.22 | 0.2225 |
| 16 | rs7200256 | 5843318 | ADD | 1636 | 0.9968 | -0.0424 | 0.9662 |
| 16 | rs12149886 | 6017104 | ADD | 1633 | 1.096 | 0.9646 | 0.3348 |
| 16 | rs8050137 | 7391457 | ADD | 1636 | 1.026 | 0.2211 | 0.825 |
| 16 | rs4786215 | 8154306 | ADD | 1632 | 0.9222 | -0.7919 | 0.4284 |
| 16 | rs720763 | 9473575 | ADD | 1615 | 1.011 | 0.1525 | 0.8788 |
| 16 | rs767019 | 11164437 | ADD | 1638 | 1.032 | 0.4373 | 0.6619 |
| 16 | rs16959859 | 12543114 | ADD | 1634 | 1.139 | 1.203 | 0.229 |
| 16 | rs8054265 | 13887849 | ADD | 1602 | 0.9894 | -0.1467 | 0.8834 |
| 16 | rs3751877 | 15062399 | ADD | 1638 | 0.9559 | -0.4023 | 0.6875 |
| 16 | rs9302387 | 20386225 | ADD | 1612 | 1.049 | 0.7008 | 0.4834 |
| 16 | rs208568 | 22863477 | ADD | 1636 | 1.008 | 0.09099 | 0.9275 |
| 16 | rs4788423 | 23926364 | ADD | 1638 | 0.9833 | -0.234 | 0.815 |
| 16 | rs716447 | 26648730 | ADD | 1636 | 0.9678 | -0.3869 | 0.6988 |
| 16 | rs11644952 | 49440976 | ADD | 1617 | 0.9265 | -0.8483 | 0.3962 |
| 16 | rs16951056 | 50909613 | ADD | 1638 | 0.9357 | -0.4795 | 0.6316 |
| 16 | rs11639960 | 54090771 | ADD | 1620 | 0.9813 | -0.2425 | 0.8084 |
| 16 | rs6500024 | 57790679 | ADD | 1637 | 0.9696 | -0.4447 | 0.6565 |
| 16 | rs12598492 | 58087166 | ADD | 1620 | 1.001 | 0.01051 | 0.9916 |
| 16 | rs10500464 | 60893955 | ADD | 1637 | 1.102 | 0.8054 | 0.4206 |
| 16 | rs16965622 | 61685347 | ADD | 1637 | 0.9554 | -0.5918 | 0.554 |
| 16 | rs11075517 | 62338554 | ADD | 1632 | 1.071 | 0.9473 | 0.3435 |
| 16 | rs12931204 | 63696897 | ADD | 1637 | 1.085 | 1.003 | 0.3161 |
| 16 | rs1423780 | 63770907 | ADD | 1638 | 0.9485 | -0.624 | 0.5327 |
| 16 | rs9933029 | 66860911 | ADD | 1614 | 0.9662 | -0.4609 | 0.6449 |
| 16 | rs889792 | 67081809 | ADD | 1620 | 1.023 | 0.251 | 0.8018 |
| 16 | rs963303 | 71929999 | ADD | 1638 | 0.8974 | -0.8806 | 0.3785 |
| 16 | rs7203412 | 72002536 | ADD | 1633 | 1.079 | 0.6698 | 0.503 |
| 16 | rs17765320 | 74572232 | ADD | 1638 | 0.9542 | -0.3075 | 0.7584 |
| 16 | rs9923061 | 74881862 | ADD | 1629 | 0.8656 | -1.214 | 0.2248 |
| 16 | rs2220233 | 75607795 | ADD | 1638 | 0.992 | -0.09632 | 0.9233 |
| 16 | rs17769245 | 75821218 | ADD | 1616 | 0.9967 | -0.03799 | 0.9697 |
| 16 | rs8051539 | 76268225 | ADD | 1628 | 1.195 | 1.929 | 0.0537 |
| 16 | rs2738545 | 77186821 | ADD | 1638 | 0.9501 | -0.7258 | 0.468 |
| 16 | rs2550626 | 77236228 | ADD | 1637 | 1.069 | 0.9721 | 0.331 |
| 16 | rs1111230 | 77728638 | ADD | 1636 | 0.8909 | -1.655 | 0.09789 |
| 16 | rs1466181 | 79175082 | ADD | 1624 | 0.9188 | -1.153 | 0.2489 |
| 16 | rs9926366 | 79645030 | ADD | 1637 | 0.8479 | -1.157 | 0.2475 |
| 16 | rs4889448 | 80543096 | ADD | 1637 | 0.9978 | -0.02955 | 0.9764 |
| 16 | rs4782872 | 80986567 | ADD | 1637 | 0.9913 | -0.1257 | 0.9 |
| 16 | rs4783102 | 83535549 | ADD | 1638 | 1.005 | 0.07128 | 0.9432 |
| 16 | rs1318275 | 83609155 | ADD | 1637 | 1.094 | 1.066 | 0.2863 |
| 16 | rs276949 | 84802018 | ADD | 1633 | 1.093 | 0.977 | 0.3286 |
| 16 | rs12446146 | 85039522 | ADD | 1635 | 1.011 | 0.1113 | 0.9114 |
| 16 | rs9923313 | 85078818 | ADD | 1637 | 1.02 | 0.2065 | 0.8364 |
| 16 | rs1867485 | 85238532 | ADD | 1621 | 1.088 | 1.202 | 0.2293 |
| 16 | rs4843669 | 86267434 | ADD | 1637 | 1.102 | 1.296 | 0.1948 |
| 16 | rs3096309 | 87959104 | ADD | 1638 | 1.149 | 1.305 | 0.1917 |
| 17 | rs11871557 | 5599344 | ADD | 1636 | 0.9624 | -0.5318 | 0.5948 |
| 17 | rs4602096 | 7414181 | ADD | 1638 | 1.039 | 0.4888 | 0.625 |
| 17 | rs4534894 | 9137020 | ADD | 1636 | 1.051 | 0.6484 | 0.5167 |
| 17 | rs2270115 | 9745449 | ADD | 1626 | 1.014 | 0.184 | 0.854 |
| 17 | rs11078839 | 10134845 | ADD | 1608 | 0.8946 | -1.303 | 0.1926 |
| 17 | rs1974700 | 11336115 | ADD | 1636 | 1.034 | 0.4236 | 0.6718 |
| 17 | rs11078034 | 11662069 | ADD | 1638 | 0.9037 | -1.412 | 0.158 |
| 17 | rs2215117 | 13247369 | ADD | 1637 | 0.9825 | -0.2222 | 0.8242 |
| 17 | rs17707404 | 14568034 | ADD | 1637 | 0.9293 | -1.044 | 0.2967 |
| 17 | rs12951251 | 14645763 | ADD | 1592 | 0.9334 | -0.8276 | 0.4079 |
| 17 | rs12951674 | 15040582 | ADD | 1623 | 0.8969 | -1.522 | 0.1279 |
| 17 | rs597985 | 16311498 | ADD | 1620 | 1.047 | 0.595 | 0.5519 |
| 17 | rs3935891 | 28843327 | ADD | 1637 | 1.04 | 0.4712 | 0.6375 |
| 17 | rs2003533 | 29525887 | ADD | 1638 | 1.161 | 1.322 | 0.1863 |
| 17 | rs854684 | 31336328 | ADD | 1596 | 0.7484 | -2.754 | 0.005895 |
| 17 | rs2023906 | 35967212 | ADD | 1635 | 0.9726 | -0.3201 | 0.7489 |
| 17 | rs11869926 | 43038124 | ADD | 1623 | 0.7658 | -0.8971 | 0.3697 |
| 17 | rs7226040 | 46975449 | ADD | 1636 | 0.9485 | -0.7438 | 0.457 |
| 17 | rs16950363 | 47109472 | ADD | 1637 | 1.088 | 0.6921 | 0.4889 |
| 17 | rs16950626 | 47269665 | ADD | 1636 | 1.181 | 1.046 | 0.2955 |
| 17 | rs17758761 | 51409524 | ADD | 1638 | 0.9815 | -0.1008 | 0.9197 |
| 17 | rs17759236 | 51534381 | ADD | 1634 | 0.9126 | -1.251 | 0.2109 |
| 17 | rs8073749 | 52630528 | ADD | 1638 | 0.868 | -1.517 | 0.1292 |
| 17 | rs2111016 | 52935949 | ADD | 1638 | 1.043 | 0.3725 | 0.7096 |
| 17 | rs2188729 | 57023916 | ADD | 1638 | 1.025 | 0.3418 | 0.7325 |
| 17 | rs1985961 | 59470994 | ADD | 1634 | 0.9195 | -1.223 | 0.2215 |
| 17 | rs11871336 | 60802782 | ADD | 1603 | 0.9905 | -0.122 | 0.9029 |
| 17 | rs8073426 | 60993743 | ADD | 1635 | 1.031 | 0.4034 | 0.6867 |
| 17 | rs4791021 | 62288734 | ADD | 1638 | 1.008 | 0.1101 | 0.9123 |
| 17 | rs740805 | 62301532 | ADD | 1636 | 0.8852 | -1.205 | 0.2284 |
| 17 | rs4791017 | 62313154 | ADD | 1635 | 1.073 | 0.8097 | 0.4181 |
| 17 | rs9972951 | 63902871 | ADD | 1635 | 0.8257 | -1.035 | 0.3008 |
| 17 | rs16972958 | 63942484 | ADD | 1635 | 1.073 | 0.5675 | 0.5704 |
| 17 | rs10445211 | 63942518 | ADD | 1611 | 1.047 | 0.6175 | 0.5369 |
| 17 | rs10852738 | 63998422 | ADD | 1633 | 1.048 | 0.4379 | 0.6614 |
| 17 | rs9910153 | 67932884 | ADD | 1631 | 1.015 | 0.2141 | 0.8305 |
| 17 | rs857501 | 68065897 | ADD | 1636 | 0.9017 | -1.392 | 0.1641 |
| 17 | rs11077627 | 68189849 | ADD | 1634 | 1.005 | 0.06201 | 0.9506 |
| 17 | rs11077629 | 68238950 | ADD | 1611 | 1.069 | 0.8699 | 0.3844 |
| 17 | rs9915558 | 68540933 | ADD | 1635 | 0.898 | -1.486 | 0.1374 |
| 17 | rs2620044 | 69347869 | ADD | 1638 | 0.9734 | -0.2993 | 0.7647 |
| 18 | rs561802 | 605394 | ADD | 1635 | 1.134 | 1.452 | 0.1464 |
| 18 | rs2612090 | 674766 | ADD | 1638 | 0.9402 | -0.8308 | 0.4061 |
| 18 | rs17521158 | 2156794 | ADD | 1637 | 0.9484 | -0.6962 | 0.4863 |
| 18 | rs2096823 | 2161031 | ADD | 1638 | 1.018 | 0.2537 | 0.7997 |
| 18 | rs1662805 | 3319448 | ADD | 1638 | 0.8 | -2.278 | 0.02271 |
| 18 | rs906800 | 7450379 | ADD | 1638 | 1.1 | 0.6135 | 0.5395 |
| 18 | rs6506598 | 8553622 | ADD | 1630 | 1.05 | 0.6671 | 0.5047 |
| 18 | rs7241781 | 9478704 | ADD | 1638 | 0.9964 | -0.04419 | 0.9648 |
| 18 | rs329017 | 9482196 | ADD | 1635 | 0.8759 | -1.742 | 0.08154 |
| 18 | rs1942150 | 9528533 | ADD | 1633 | 1.039 | 0.4194 | 0.6749 |
| 18 | rs206499 | 10274173 | ADD | 1638 | 1.005 | 0.06778 | 0.946 |
| 18 | rs547668 | 10466821 | ADD | 1638 | 1.047 | 0.526 | 0.5989 |
| 18 | rs11665145 | 10802708 | ADD | 1638 | 1.005 | 0.07247 | 0.9422 |
| 18 | rs9303722 | 11234106 | ADD | 1629 | 1.024 | 0.3195 | 0.7494 |
| 18 | rs6505813 | 13412042 | ADD | 1637 | 1.089 | 0.8554 | 0.3923 |
| 18 | rs4800181 | 20042745 | ADD | 1635 | 1.113 | 0.534 | 0.5934 |
| 18 | rs4340399 | 22329374 | ADD | 1636 | 1.045 | 0.5766 | 0.5642 |
| 18 | rs1030198 | 22733958 | ADD | 1638 | 0.9948 | -0.0734 | 0.9415 |
| 18 | rs12327234 | 22786511 | ADD | 1622 | 1.038 | 0.3175 | 0.7508 |
| 18 | rs8084703 | 23750943 | ADD | 1634 | 0.9085 | -0.862 | 0.3887 |
| 18 | rs355306 | 31359715 | ADD | 1638 | 1.013 | 0.1055 | 0.916 |
| 18 | rs16970456 | 34337125 | ADD | 1626 | 1.024 | 0.2845 | 0.776 |
| 18 | rs12326401 | 34353899 | ADD | 1624 | 0.9845 | -0.1733 | 0.8624 |
| 18 | rs2032185 | 35956480 | ADD | 1631 | 1.101 | 0.8067 | 0.4198 |
| 18 | rs11082490 | 41666626 | ADD | 1638 | 1.075 | 0.7506 | 0.4529 |
| 18 | rs1489169 | 44129658 | ADD | 1622 | 0.9143 | -1.201 | 0.2297 |
| 18 | rs1038670 | 44152193 | ADD | 1609 | 0.9528 | -0.4499 | 0.6528 |
| 18 | rs7237395 | 44564353 | ADD | 1624 | 0.9478 | -0.6658 | 0.5056 |
| 18 | rs7233656 | 47242446 | ADD | 1635 | 1.013 | 0.1764 | 0.86 |
| 18 | rs8097646 | 53159904 | ADD | 1638 | 1.063 | 0.4033 | 0.6867 |
| 18 | rs7229750 | 53312878 | ADD | 1637 | 1.088 | 1.088 | 0.2767 |
| 18 | rs11872825 | 54362167 | ADD | 1638 | 1.01 | 0.1323 | 0.8947 |
| 18 | rs9966194 | 54870416 | ADD | 1618 | 0.8803 | -1.747 | 0.08056 |
| 18 | rs10503023 | 54983755 | ADD | 1638 | 0.8613 | -1.764 | 0.07778 |
| 18 | rs9956301 | 58929998 | ADD | 1634 | 1.054 | 0.4476 | 0.6544 |
| 18 | rs7237857 | 60012386 | ADD | 1637 | 1.165 | 2.045 | 0.04089 |
| 18 | rs3764472 | 62327249 | ADD | 1636 | 1.135 | 0.5721 | 0.5673 |
| 18 | rs6566391 | 64660831 | ADD | 1634 | 0.9098 | -1.314 | 0.1887 |
| 18 | rs8098624 | 68669199 | ADD | 1636 | 1.125 | 1.615 | 0.1064 |
| 18 | rs17088303 | 69596587 | ADD | 1608 | 0.9922 | -0.09692 | 0.9228 |
| 18 | rs1790887 | 70041720 | ADD | 1634 | 1.143 | 1.466 | 0.1426 |
| 18 | rs7228143 | 70042132 | ADD | 1628 | 0.908 | -0.6471 | 0.5176 |
| 18 | rs1582404 | 70122069 | ADD | 1632 | 1.239 | 2.252 | 0.02433 |
| 18 | rs1256726 | 70216946 | ADD | 1638 | 1.098 | 1.264 | 0.2063 |
| 18 | rs1559806 | 70259767 | ADD | 1638 | 0.9711 | -0.4057 | 0.685 |
| 18 | rs12964454 | 70389710 | ADD | 1634 | 0.9806 | -0.2692 | 0.7878 |
| 19 | rs4806891 | 2898152 | ADD | 1638 | 0.9932 | -0.07373 | 0.9412 |
| 19 | rs10424563 | 3369556 | ADD | 1618 | 1.072 | 0.56 | 0.5755 |
| 19 | rs6603120 | 7343349 | ADD | 1583 | 1.028 | 0.3174 | 0.7509 |
| 19 | rs282749 | 11826918 | ADD | 1601 | 0.9235 | -1.082 | 0.2791 |
| 19 | rs2112460 | 13451412 | ADD | 1637 | 1.172 | 2.203 | 0.02762 |
| 19 | rs10409463 | 13493383 | ADD | 1633 | 0.9721 | -0.3876 | 0.6983 |
| 19 | rs3786853 | 15389983 | ADD | 1617 | 0.9231 | -0.9738 | 0.3301 |
| 19 | rs2283612 | 15621213 | ADD | 1635 | 1.187 | 1.627 | 0.1036 |
| 19 | rs7250793 | 19896864 | ADD | 1605 | 0.9888 | -0.1114 | 0.9113 |
| 19 | rs4932829 | 23156405 | ADD | 1636 | 0.845 | -2.042 | 0.04114 |
| 19 | rs667874 | 23437034 | ADD | 1595 | 1.015 | 0.1935 | 0.8466 |
| 19 | rs17772344 | 34038879 | ADD | 1636 | 1.068 | 0.5982 | 0.5497 |
| 19 | rs12461309 | 34059832 | ADD | 1636 | 0.9541 | -0.661 | 0.5086 |
| 19 | rs12974188 | 34066325 | ADD | 1613 | 0.9602 | -0.5414 | 0.5882 |
| 19 | rs4804918 | 35305432 | ADD | 1633 | 0.9356 | -0.8946 | 0.371 |
| 19 | rs7255838 | 35899432 | ADD | 1631 | 0.8041 | -2.258 | 0.02393 |
| 19 | rs983495 | 35989883 | ADD | 1638 | 1.089 | 1.175 | 0.2399 |
| 19 | rs16968313 | 38823404 | ADD | 1637 | 1.119 | 0.8396 | 0.4011 |
| 19 | rs2191139 | 46693050 | ADD | 1638 | 1.133 | 1.712 | 0.08685 |
| 19 | rs204541 | 49646953 | ADD | 1634 | 1.205 | 2.614 | 0.008951 |
| 19 | rs7255053 | 53200453 | ADD | 1627 | 1.057 | 0.7359 | 0.4618 |
| 19 | rs7256201 | 56396851 | ADD | 1587 | 1.063 | 0.7871 | 0.4312 |
| 19 | rs324119 | 57591368 | ADD | 1629 | 1.183 | 1.268 | 0.2049 |
| 19 | rs10401904 | 57812371 | ADD | 1635 | 1.014 | 0.1876 | 0.8512 |
| 19 | rs7258857 | 58798706 | ADD | 1638 | 1.27 | 1.047 | 0.2952 |
| 19 | rs873732 | 61437332 | ADD | 1599 | 0.986 | -0.1481 | 0.8823 |
| 20 | rs4813841 | 779093 | ADD | 1626 | 1.019 | 0.2566 | 0.7975 |
| 20 | rs1610304 | 2097912 | ADD | 1637 | 0.9336 | -0.4783 | 0.6324 |
| 20 | rs4815349 | 2495597 | ADD | 1638 | 1.037 | 0.5149 | 0.6066 |
| 20 | rs6084154 | 2694354 | ADD | 1634 | 0.9852 | -0.1647 | 0.8692 |
| 20 | rs8184236 | 2987115 | ADD | 1633 | 1.055 | 0.7508 | 0.4528 |
| 20 | rs6116265 | 4139058 | ADD | 1637 | 0.9634 | -0.411 | 0.6811 |
| 20 | rs2065706 | 4631295 | ADD | 1581 | 1.051 | 0.5325 | 0.5944 |
| 20 | rs6118724 | 9694345 | ADD | 1627 | 1.02 | 0.269 | 0.7879 |
| 20 | rs1883949 | 11205383 | ADD | 1613 | 0.8962 | -1.458 | 0.1448 |
| 20 | rs6108958 | 11341882 | ADD | 1595 | 1.009 | 0.1285 | 0.8978 |
| 20 | rs6110305 | 14403497 | ADD | 1634 | 0.9971 | -0.02896 | 0.9769 |
| 20 | rs1233757 | 15502640 | ADD | 1638 | 1.055 | 0.7209 | 0.471 |
| 20 | rs6044565 | 16930918 | ADD | 1622 | 1.141 | 1.719 | 0.08565 |
| 20 | rs6046805 | 20272341 | ADD | 1575 | 0.8387 | -2.377 | 0.01744 |
| 20 | rs11697967 | 30975363 | ADD | 1638 | 1.109 | 1.358 | 0.1745 |
| 20 | rs1062943 | 41603741 | ADD | 1631 | 0.9419 | -0.7943 | 0.427 |
| 20 | rs11086907 | 41986851 | ADD | 1638 | 1.201 | 1.616 | 0.1061 |
| 20 | rs6031256 | 42017031 | ADD | 1638 | 1.037 | 0.4749 | 0.6349 |
| 20 | rs1998033 | 42992323 | ADD | 1638 | 1.004 | 0.04856 | 0.9613 |
| 20 | rs2425795 | 44301702 | ADD | 1637 | 1.023 | 0.3107 | 0.756 |
| 20 | rs1591168 | 45760849 | ADD | 1630 | 1.012 | 0.146 | 0.884 |
| 20 | rs2073073 | 46702944 | ADD | 1638 | 0.8912 | -0.7475 | 0.4547 |
| 20 | rs6063312 | 46776466 | ADD | 1638 | 0.9475 | -0.5453 | 0.5856 |
| 20 | rs1810812 | 48452841 | ADD | 1638 | 0.9354 | -0.8813 | 0.3782 |
| 20 | rs2869991 | 48497644 | ADD | 1628 | 0.8604 | -1.878 | 0.06045 |
| 20 | rs156618 | 51426981 | ADD | 1638 | 0.9932 | -0.08005 | 0.9362 |
| 20 | rs16984758 | 53528718 | ADD | 1615 | 0.9457 | -0.3895 | 0.6969 |
| 20 | rs666073 | 53592561 | ADD | 1638 | 0.9708 | -0.2702 | 0.787 |
| 20 | rs584635 | 53671210 | ADD | 1637 | 0.9265 | -0.9856 | 0.3243 |
| 20 | rs6014763 | 54582692 | ADD | 1623 | 1.091 | 1.198 | 0.2309 |
| 20 | rs8117354 | 54917455 | ADD | 1637 | 0.8542 | -1.541 | 0.1233 |
| 20 | rs6064671 | 56558178 | ADD | 1617 | 0.9085 | -1.214 | 0.2248 |
| 20 | rs4810164 | 57377765 | ADD | 1637 | 1.023 | 0.2784 | 0.7807 |
| 20 | rs6026872 | 57420894 | ADD | 1636 | 0.9556 | -0.3712 | 0.7105 |
| 20 | rs17793950 | 58855929 | ADD | 1638 | 1.171 | 1.025 | 0.3054 |
| 20 | rs868544 | 59716357 | ADD | 1622 | 0.9734 | -0.3748 | 0.7078 |
| 21 | rs7364073 | 17555710 | ADD | 1630 | 0.9932 | -0.09157 | 0.927 |
| 21 | rs2408239 | 22254497 | ADD | 1636 | 1.003 | 0.02442 | 0.9805 |
| 21 | rs2828654 | 24254905 | ADD | 1637 | 0.9075 | -0.9177 | 0.3588 |
| 21 | rs2831270 | 28246805 | ADD | 1637 | 0.9949 | -0.05506 | 0.9561 |
| 21 | rs2831440 | 28349969 | ADD | 1622 | 0.7883 | -3.126 | 0.001774 |
| 21 | rs2250910 | 28725372 | ADD | 1638 | 1.02 | 0.1851 | 0.8531 |
| 21 | rs459617 | 30062624 | ADD | 1637 | 1.122 | 1.448 | 0.1475 |
| 21 | rs7283316 | 31138124 | ADD | 1620 | 1.06 | 0.8306 | 0.4062 |
| 21 | rs11702844 | 33681746 | ADD | 1638 | 0.9465 | -0.501 | 0.6164 |
| 21 | rs8129326 | 34734497 | ADD | 1638 | 0.957 | -0.6319 | 0.5274 |
| 21 | rs2243982 | 34823160 | ADD | 1637 | 1.059 | 0.813 | 0.4162 |
| 21 | rs2836365 | 38690144 | ADD | 1638 | 1.082 | 1.026 | 0.3047 |
| 21 | rs16998883 | 40204778 | ADD | 1632 | 0.8465 | -1.725 | 0.08448 |
| 21 | rs2837302 | 40225765 | ADD | 1607 | 0.9277 | -1.048 | 0.2948 |
| 21 | rs4816665 | 40235027 | ADD | 1638 | 1.311 | 2.893 | 0.003817 |
| 21 | rs2837468 | 40454200 | ADD | 1637 | 0.9545 | -0.569 | 0.5693 |
| 21 | rs11701426 | 41120633 | ADD | 1638 | 0.9502 | -0.6083 | 0.543 |
| 21 | rs965494 | 41431693 | ADD | 1637 | 1.061 | 0.7717 | 0.4403 |
| 21 | rs3746893 | 42034946 | ADD | 1593 | 0.9959 | -0.05625 | 0.9551 |
| 21 | rs7283380 | 42064663 | ADD | 1619 | 1.083 | 1.085 | 0.278 |
| 21 | rs11203189 | 42158495 | ADD | 1621 | 0.8179 | -2.243 | 0.02489 |
| 21 | rs220153 | 42417692 | ADD | 1637 | 0.994 | -0.07447 | 0.9406 |
| 21 | rs3827225 | 42523848 | ADD | 1637 | 0.9524 | -0.5689 | 0.5694 |
| 21 | rs2269127 | 42950306 | ADD | 1626 | 1.007 | 0.07294 | 0.9419 |
| 21 | rs967445 | 43069463 | ADD | 1628 | 1.023 | 0.3068 | 0.759 |
| 21 | rs6586252 | 43149456 | ADD | 1635 | 0.997 | -0.0419 | 0.9666 |
| 21 | rs4818955 | 43653515 | ADD | 1626 | 1.026 | 0.3488 | 0.7273 |
| 22 | rs11704699 | 16150181 | ADD | 1638 | 1.056 | 0.4733 | 0.636 |
| 22 | rs4239844 | 16265697 | ADD | 1585 | 1.164 | 2.056 | 0.03976 |
| 22 | rs2252257 | 17020300 | ADD | 1632 | 1.039 | 0.4524 | 0.651 |
| 22 | rs5748410 | 18098144 | ADD | 1636 | 0.9298 | -0.9821 | 0.3261 |
| 22 | rs5761913 | 19095021 | ADD | 1631 | 1.04 | 0.4659 | 0.6413 |
| 22 | rs5751713 | 22363466 | ADD | 1638 | 1.021 | 0.1822 | 0.8554 |
| 22 | rs7288210 | 25272127 | ADD | 1622 | 1.056 | 0.7331 | 0.4635 |
| 22 | rs12628385 | 34312535 | ADD | 1636 | 0.8061 | -0.9846 | 0.3248 |
| 22 | rs132985 | 36893417 | ADD | 1619 | 0.9791 | -0.2898 | 0.772 |
| 22 | rs5750871 | 38399395 | ADD | 1638 | 0.9978 | -0.01957 | 0.9844 |
| 22 | rs6006632 | 42778379 | ADD | 1585 | 1.025 | 0.3184 | 0.7502 |
| 22 | rs5764076 | 42790778 | ADD | 1631 | 0.8161 | -1.652 | 0.09859 |
| 22 | rs16992075 | 43128674 | ADD | 1634 | 0.8842 | -1.227 | 0.2199 |
| 22 | rs1557553 | 43139648 | ADD | 1606 | 1.092 | 0.8396 | 0.4011 |
| 22 | rs9614868 | 43144976 | ADD | 1635 | 0.9629 | -0.5216 | 0.6019 |
| 22 | rs7285004 | 43153364 | ADD | 1628 | 1.035 | 0.458 | 0.647 |
| 22 | rs9616423 | 47872695 | ADD | 1560 | 1.054 | 0.6086 | 0.5428 |
| 22 | rs17825961 | 48055889 | ADD | 1638 | 0.8857 | -0.7662 | 0.4436 |
| 22 | rs848710 | 48138336 | ADD | 1637 | 1.041 | 0.5273 | 0.598 |
| 22 | rs9617104 | 48782778 | ADD | 1634 | 1.103 | 0.7339 | 0.463 |
| 22 | rs1555048 | 49030475 | ADD | 1625 | 0.9583 | -0.5082 | 0.6113 |
