# Supplementary material for: Genome-wide association study of antidepressant response: involvement of the inorganic cation transmembrane transporter activity pathway
Source: BMC Psychiatry. 2016 Apr 18;16:106. doi: 10.1186/s12888-016-0813-x (PMC4836090; doi:10.1186/s12888-016-0813-x)
Supplement: Additional file 7: Table S5. — Description and statistics referred to the SNPs that showed p<0.05 (remission phenotype) in both the analyzed samples. (DOC 155 kb) [file 12888_2016_813_MOESM7_ESM.doc]

**Table S5**: description and statistics referred to the SNPs that showed p<0.05 (remission phenotype) in both the analyzed samples.

|  | | **STAR*D sample** | | | | **Korean sample** | | | | **Annotation** |
| --- | --- | --- | --- | --- | --- | --- | --- | --- | --- | --- |
| **SNP** | **Chr** | **position** | **odds ratio** | **statistic** | **p** | **position** | **odds ratio** | **statistic** | **p** |  |
| rs10885239 | 10 | 84460638 | 0.8252 | -2.287 | 0.02221 | 84470658 | 3.439 | 2.444 | 0.01454 | NRG3(0) |
| rs10899345 | 11 | 76425167 | 1.168 | 2.144 | 0.03202 | 76747519 | 0.4047 | -2.435 | 0.0149 | B3GNT6(0)|ACER3(+12.67kb) |
| rs10969027 | 9 | 29234767 | 0.858 | -2.029 | 0.04245 | 29244767 | 2.686 | 2.671 | 0.007565 | . |
| rs11159695 | 14 | 84763606 | 0.8453 | -2.053 | 0.04008 | 85693853 | 2.652 | 2.419 | 0.01557 | . |
| rs11203189 | 21 | 42158495 | 0.8179 | -2.243 | 0.02489 | 43285426 | 0.4494 | -1.993 | 0.04622 | PRDM15(0)|C2CD2(-19.79kb) |
| rs1125640 | 6 | 161512090 | 0.6953 | -2.371 | 0.01772 | 161592100 | 0.2303 | -2.175 | 0.02962 | AGPAT4-IT1(+9.086kb)|AGPAT4(0) |
| rs11589992 | 1 | 32072717 | 0.8595 | -2.126 | 0.03347 | 32300130 | 1.924 | 2.004 | 0.04503 | SPOCD1(+18.48kb)|=missense |
| rs11618012 | 13 | 34237417 | 1.279 | 2.6 | 0.009312 | 35339417 | 2.433 | 2.359 | 0.01832 | . |
| rs1209063 | 5 | 157275238 | 1.175 | 2.251 | 0.02442 | 157342660 | 2.123 | 2.019 | 0.04347 | . |
| rs12102156 | 15 | 50376391 | 1.224 | 2.154 | 0.03125 | 52589099 | 2.571 | 2.447 | 0.0144 | MYO5C(+1.104kb)|MYO5A(-10.38kb)|MIR1266(+19.7kb) |
| rs13096649 | 3 | 185694678 | 0.8392 | -2.449 | 0.01431 | 184211984 | 2.423 | 2.128 | 0.03333 | . |
| rs1460930 | 8 | 101183970 | 0.8295 | -2.549 | 0.01079 | 101114794 | 2.273 | 2.211 | 0.02706 | RGS22(0) |
| rs1482900 | 5 | 105172733 | 0.8074 | -2.064 | 0.03902 | 105144834 | 2.37 | 2.156 | 0.03112 | . |
| rs150353 | 15 | 87729193 | 0.8645 | -2.012 | 0.04424 | 89928189 | 0.5223 | -2.015 | 0.04395 | MIR9-3(+16.85kb)|LINC00925(0) |
| rs1582404 | 18 | 70122069 | 1.239 | 2.252 | 0.02433 | 71971089 | 3.953 | 2.804 | 0.005055 | CYB5A(+11.84kb)|C18orf63(-12.02kb) |
| rs1662805 | 18 | 3319448 | 0.8 | -2.278 | 0.02271 | 3329448 | 0.1231 | -2.327 | 0.01998 | . |
| rs168549 | 1 | 55771023 | 1.175 | 1.994 | 0.04614 | 55998435 | 0.4399 | -2.293 | 0.02185 | . |
| rs16929898 | 10 | 29530715 | 1.586 | 2.423 | 0.0154 | 29490709 | 8.319 | 2.821 | 0.004786 | . |
| rs17059088 | 8 | 28356379 | 0.8007 | -2.529 | 0.01142 | 28300460 | 2.452 | 2.386 | 0.01705 | FBXO16(0) |
| rs17078394 | 13 | 22651669 | 1.355 | 3.002 | 0.002681 | 23753669 | 2.236 | 2 | 0.04549 | SGCG(-1.39kb) |
| rs17080320 | 6 | 151154676 | 1.629 | 2.027 | 0.0427 | 151112983 | 3.877 | 2.032 | 0.04211 | PLEKHG1(0) |
| rs17096688 | 14 | 97724652 | 1.47 | 1.988 | 0.04678 | 98654899 | 3.111 | 2.103 | 0.03551 | . |
| rs17493545 | 4 | 37061405 | 0.735 | -3.144 | 0.001666 | 37385010 | 0.399 | -1.99 | 0.0466 | NWD2(0) |
| rs1776447 | 6 | 37710438 | 1.225 | 2.064 | 0.03903 | 37602460 | 0.03434 | -2.03 | 0.04241 | MDGA1(0) |
| rs1994816 | 4 | 181205425 | 1.223 | 2.368 | 0.01789 | 180968431 | 2.544 | 2.161 | 0.03071 | . |
| rs204541 | 19 | 49646953 | 1.205 | 2.614 | 0.008951 | 44955113 | 2.361 | 2.417 | 0.01564 | ZNF229(+2.347kb) |
| rs2070664 | 15 | 32872493 | 1.186 | 2.297 | 0.02164 | 35085201 | 2.096 | 1.97 | 0.04889 | LOC101928174(0)|ACTC1(0) |
| rs2112460 | 19 | 13451412 | 1.172 | 2.203 | 0.02762 | 13590412 | 2.514 | 2.045 | 0.04082 | CACNA1A(0) |
| rs2237077 | 5 | 139700584 | 1.254 | 2.821 | 0.004786 | 139720400 | 0.2954 | -2.121 | 0.03388 | SLC4A9(-19.39kb)|HBEGF(0) |
| rs236919 | 11 | 116600571 | 1.202 | 2.617 | 0.00888 | 117095361 | 2.793 | 2.384 | 0.01712 | TAGLN(+19.85kb)|RNF214(-7.979kb)|PCSK7(0) |
| rs2532560 | 12 | 4032132 | 1.202 | 2.002 | 0.04527 | 4161871 | 0.1673 | -3.079 | 0.002075 | . |
| rs271421 | 5 | 6407132 | 1.299 | 2.634 | 0.008432 | 6354132 | 5.021 | 2.871 | 0.00409 | MED10(-17.91kb)|FLJ33360(+16.73kb) |
| rs2831440 | 21 | 28349969 | 0.7883 | -3.126 | 0.001774 | 29428098 | 3.531 | 3.045 | 0.002327 | . |
| rs2881640 | 15 | 85261718 | 0.8372 | -2.04 | 0.04133 | 87460714 | 2.051 | 2.19 | 0.02851 | AGBL1(0) |
| rs4129100 | 1 | 3978308 | 1.16 | 2.155 | 0.03114 | 4078448 | 0.4443 | -2.357 | 0.01843 | . |
| rs4147839 | 1 | 94281814 | 0.8453 | -2.215 | 0.02676 | 94509226 | 2.347 | 2.091 | 0.03649 | ABCA4(0) |
| rs4239844 | 22 | 16265697 | 1.164 | 2.056 | 0.03976 | 17885697 | 0.4418 | -2.309 | 0.02093 | CECR2(0) |
| rs4278360 | 1 | 241119680 | 0.819 | -2.122 | 0.03381 | 243053057 | 2.18 | 2.226 | 0.02604 | . |
| rs4533716 | 4 | 163462694 | 0.8413 | -2.4 | 0.01639 | 163243244 | 0.4893 | -2.06 | 0.03937 | . |
| rs4665665 | 2 | 24234219 | 1.272 | 2.228 | 0.02585 | 24380715 | 0.3308 | -2.002 | 0.04526 | FAM228A(-17.26kb)|FAM228B(0)|=missense |
| rs4737771 | 8 | 67354933 | 1.255 | 2.005 | 0.04492 | 67192379 | 0.2423 | -2.943 | 0.003246 | . |
| rs4816665 | 21 | 40235027 | 1.311 | 2.893 | 0.003817 | 41313157 | 4.093 | 2.049 | 0.04042 | PCP4(+11.84kb) |
| rs4876266 | 8 | 1903551 | 1.22 | 2.164 | 0.03049 | 1916144 | 6.294 | 1.963 | 0.0497 | KBTBD11(-5.899kb)|ARHGEF10(+9.337kb) |
| rs489257 | 11 | 78768875 | 0.8153 | -2.72 | 0.006523 | 79091227 | 2.453 | 2.429 | 0.01513 | TENM4(0) |
| rs4932829 | 19 | 23156405 | 0.845 | -2.042 | 0.04114 | 23364565 | 0.3612 | -2.228 | 0.0259 | =missense |
| rs4952404 | 2 | 40414134 | 0.8245 | -2.221 | 0.02635 | 40560630 | 0.4381 | -2.083 | 0.03726 | SLC8A1(0) |
| rs604356 | 12 | 116620471 | 1.22 | 2.077 | 0.03783 | 118136088 | 2.859 | 2.498 | 0.0125 | KSR2(0) |
| rs6046805 | 20 | 20272341 | 0.8387 | -2.377 | 0.01744 | 20324341 | 0.4842 | -1.964 | 0.04958 | C20orf26(0) |
| rs605265 | 12 | 116620279 | 1.25 | 3.038 | 0.00238 | 118135896 | 2.225 | 2.423 | 0.01539 | KSR2(0) |
| rs672170 | 6 | 153386878 | 1.175 | 2.155 | 0.03114 | 153345185 | 3.484 | 3.13 | 0.001748 | RGS17(0)|=missense |
| rs6746088 | 2 | 107353467 | 1.175 | 2.294 | 0.02179 | 107987035 | 2.554 | 2.238 | 0.02521 | . |
| rs6757604 | 2 | 119961173 | 1.154 | 1.985 | 0.04714 | 120244703 | 3.43 | 2.823 | 0.004759 | SCTR(0) |
| rs6782465 | 3 | 144584003 | 0.7657 | -2.359 | 0.01835 | 143101313 | 0.2987 | -2.392 | 0.01677 | SLC9A9(0) |
| rs6856464 | 4 | 26432096 | 1.406 | 2.277 | 0.02276 | 26822998 | 9.263 | 1.961 | 0.04983 | . |
| rs6902696 | 6 | 16915071 | 1.296 | 2.914 | 0.003574 | 16807092 | 0.4191 | -2.088 | 0.03679 | . |
| rs6966038 | 7 | 156573159 | 0.7083 | -4.146 | 3.38e-05 | 156880398 | 0.1193 | -2.233 | 0.02555 | . |
| rs6966038 | 7 | 156573159 | 0.7083 | -4.146 | 3.38e-05 | 156880398 | 0.1193 | -2.233 | 0.02555 | . |
| rs7003979 | 8 | 15216360 | 1.209 | 2.656 | 0.00791 | 15171989 | 0.5535 | -2.005 | 0.045 | . |
| rs706349 | 4 | 140819757 | 1.166 | 2.055 | 0.03983 | 140600307 | 0.4013 | -2.191 | 0.02843 | MGST2(0) |
| rs7090118 | 10 | 18499288 | 1.174 | 2.053 | 0.04009 | 18459282 | 2.718 | 2.544 | 0.01097 | CACNB2(0) |
| rs7237857 | 18 | 60012386 | 1.165 | 2.045 | 0.04089 | 61861406 | 0.4184 | -2.414 | 0.0158 | LOC400654(-18.91kb)|LOC284294(0) |
| rs7255838 | 19 | 35899432 | 0.8041 | -2.258 | 0.02393 | 31207592 | 0.4174 | -2.125 | 0.03363 | . |
| rs759656 | 8 | 140739149 | 1.244 | 2.794 | 0.0052 | 140669967 | 2.783 | 2.05 | 0.04033 | KCNK9(0) |
| rs766127 | 6 | 153355525 | 1.208 | 2.374 | 0.01758 | 153313832 | 2.896 | 2.954 | 0.003134 | RGS17(-18.2kb)|MTRF1L(0)|FBXO5(+9.092kb)|=missense |
| rs7670660 | 4 | 129777843 | 1.248 | 2.495 | 0.01261 | 129558393 | 2.485 | 2.06 | 0.03942 | . |
| rs7737692 | 5 | 1514167 | 1.324 | 3.686 | 0.0002276 | 1461167 | 1.992 | 2.017 | 0.04371 | SLC6A3(+15.62kb)|LPCAT1(-0.374kb) |
| rs7793728 | 7 | 36028310 | 0.8269 | -2.241 | 0.02505 | 36061785 | 0.3914 | -2.237 | 0.02527 | . |
| rs7860361 | 9 | 97187625 | 0.7971 | -2.689 | 0.007157 | 98147804 | 0.1519 | -2.073 | 0.03819 | . |
| rs8176984 | 10 | 30771666 | 1.254 | 2.226 | 0.02602 | 30731660 | 2.352 | 2.047 | 0.04062 | MAP3K8(0) |
| rs847440 | 7 | 16984957 | 0.8576 | -2.067 | 0.03875 | 17018432 | 0.3158 | -2.534 | 0.01128 | . |
| rs854684 | 17 | 31336328 | 0.7484 | -2.754 | 0.005895 | 34312215 | 2.495 | 2.081 | 0.03743 | CCL15(-12.4kb)|CCL16(+3.692kb)|CCL15-CCL14(0)|CCL14(0) |
| rs9315310 | 13 | 34249431 | 1.21 | 2.112 | 0.0347 | 35351431 | 3.672 | 3.132 | 0.001735 | . |
| rs9444725 | 6 | 90770775 | 0.8182 | -2.435 | 0.01488 | 90714054 | 0.4412 | -2.051 | 0.04023 | BACH2(0) |
| rs9594724 | 13 | 41767119 | 0.8365 | -2.084 | 0.03717 | 42869119 | 0.1222 | -1.969 | 0.049 | AKAP11(0) |
| rs9806128 | 15 | 80051917 | 1.228 | 2.39 | 0.01683 | 82264862 | 3.255 | 2.571 | 0.01014 | . |
| rs994827 | 14 | 21419526 | 1.274 | 2.19 | 0.02851 | 22349686 | 0.3536 | -2.589 | 0.009618 | . |
