# Supplementary material for: Genome-wide association study of antidepressant response: involvement of the inorganic cation transmembrane transporter activity pathway
Source: BMC Psychiatry. 2016 Apr 18;16:106. doi: 10.1186/s12888-016-0813-x (PMC4836090; doi:10.1186/s12888-016-0813-x)
Supplement: Additional file 8: Table S6. — SNPs that showed p<0.05 (response phenotype) in the Korean sample and are available in the STAR*D. (DOC 1542 kb) [file 12888_2016_813_MOESM8_ESM.doc]

**Table S6**: SNPs that showed p<0.05 (response phenotype) in the Korean sample and are available in the STAR*D.

| **chromosome** | **SNP** | **position** | **test** | **non missing** | **odds ratio** | **stat** | **p** |
| --- | --- | --- | --- | --- | --- | --- | --- |
| 1 | rs7527871 | 2271586 | ADD | 1655 | 1.085 | 0.8879 | 0.3746 |
| 1 | rs1798246 | 3070715 | ADD | 1667 | 1.081 | 1.105 | 0.269 |
| 1 | rs9439550 | 5257031 | ADD | 1662 | 0.9357 | -0.9468 | 0.3437 |
| 1 | rs4908605 | 7168021 | ADD | 1664 | 0.9086 | -1.331 | 0.1831 |
| 1 | rs1476047 | 7234759 | ADD | 1667 | 1.034 | 0.465 | 0.6419 |
| 1 | rs707458 | 7754799 | ADD | 1666 | 1.095 | 1.294 | 0.1956 |
| 1 | rs17032281 | 8144743 | ADD | 1667 | 0.9205 | -1.153 | 0.2491 |
| 1 | rs7546225 | 8794552 | ADD | 1612 | 0.9224 | -1.033 | 0.3014 |
| 1 | rs4240896 | 9636087 | ADD | 1665 | 1.004 | 0.05923 | 0.9528 |
| 1 | rs6541017 | 9694151 | ADD | 1667 | 1.101 | 1.001 | 0.3167 |
| 1 | rs10803459 | 14336754 | ADD | 1667 | 0.9205 | -1.193 | 0.233 |
| 1 | rs6694029 | 15145768 | ADD | 1614 | 1.021 | 0.2232 | 0.8234 |
| 1 | rs10927791 | 15669395 | ADD | 1661 | 1.115 | 1.591 | 0.1116 |
| 1 | rs2142629 | 20480787 | ADD | 1657 | 1.104 | 1.421 | 0.1553 |
| 1 | rs1767523 | 27928929 | ADD | 1649 | 1.023 | 0.3383 | 0.7351 |
| 1 | rs3737606 | 29503543 | ADD | 1667 | 0.9475 | -0.6993 | 0.4843 |
| 1 | rs4654342 | 29638231 | ADD | 1667 | 0.8413 | -1.772 | 0.07638 |
| 1 | rs646198 | 30658112 | ADD | 1625 | 1.09 | 0.867 | 0.3859 |
| 1 | rs7530095 | 31090117 | ADD | 1662 | 0.9997 | -0.003716 | 0.997 |
| 1 | rs552124 | 32038613 | ADD | 1666 | 0.9951 | -0.06361 | 0.9493 |
| 1 | rs11589992 | 32072717 | ADD | 1667 | 0.8747 | -1.937 | 0.05276 |
| 1 | rs4653036 | 34688708 | ADD | 1665 | 1.059 | 0.8289 | 0.4072 |
| 1 | rs6425939 | 35401376 | ADD | 1627 | 1.023 | 0.2432 | 0.8078 |
| 1 | rs6679637 | 38579217 | ADD | 1661 | 1.104 | 0.8744 | 0.3819 |
| 1 | rs539853 | 39967490 | ADD | 1665 | 0.8576 | -1.638 | 0.1014 |
| 1 | rs841860 | 43139478 | ADD | 1665 | 1.064 | 0.6965 | 0.4861 |
| 1 | rs6429541 | 44738310 | ADD | 1667 | 0.9321 | -0.9407 | 0.3469 |
| 1 | rs868351 | 45782405 | ADD | 1651 | 0.9855 | -0.1723 | 0.8632 |
| 1 | rs720413 | 46992410 | ADD | 1667 | 1.009 | 0.1291 | 0.8972 |
| 1 | rs545670 | 47715009 | ADD | 1663 | 0.8165 | -1.825 | 0.06804 |
| 1 | rs1342876 | 58351200 | ADD | 1614 | 1.051 | 0.5326 | 0.5943 |
| 1 | rs1440575 | 59796529 | ADD | 1665 | 1.057 | 0.8075 | 0.4194 |
| 1 | rs437021 | 61510858 | ADD | 1665 | 0.9618 | -0.565 | 0.572 |
| 1 | rs3790861 | 63860032 | ADD | 1657 | 0.9778 | -0.1789 | 0.858 |
| 1 | rs1340429 | 74036816 | ADD | 1667 | 0.8988 | -1.504 | 0.1325 |
| 1 | rs7521977 | 79006081 | ADD | 1660 | 1.015 | 0.1679 | 0.8666 |
| 1 | rs1896176 | 81249647 | ADD | 1666 | 0.8549 | -1.16 | 0.2462 |
| 1 | rs551156 | 84613239 | ADD | 1622 | 0.9571 | -0.6384 | 0.5232 |
| 1 | rs1108394 | 86424070 | ADD | 1631 | 0.9973 | -0.02607 | 0.9792 |
| 1 | rs12750427 | 86768404 | ADD | 1663 | 0.9553 | -0.458 | 0.647 |
| 1 | rs17130969 | 90328738 | ADD | 1630 | 0.9038 | -0.9486 | 0.3428 |
| 1 | rs871664 | 94382066 | ADD | 1667 | 0.8842 | -1.761 | 0.07821 |
| 1 | rs10874867 | 94856424 | ADD | 1663 | 1.142 | 1.738 | 0.08217 |
| 1 | rs6694095 | 102057631 | ADD | 1662 | 0.9884 | -0.1314 | 0.8954 |
| 1 | rs769163 | 102076740 | ADD | 1662 | 1.058 | 0.8175 | 0.4136 |
| 1 | rs9729206 | 102389039 | ADD | 1665 | 1.018 | 0.2641 | 0.7917 |
| 1 | rs1528688 | 106188422 | ADD | 1667 | 0.9472 | -0.6977 | 0.4854 |
| 1 | rs10494092 | 108935362 | ADD | 1603 | 0.9905 | -0.1176 | 0.9064 |
| 1 | rs521093 | 110169753 | ADD | 1665 | 1.209 | 2.096 | 0.03604 |
| 1 | rs1936942 | 111251931 | ADD | 1667 | 1.076 | 1.056 | 0.2909 |
| 1 | rs3393 | 111843672 | ADD | 1665 | 0.9699 | -0.445 | 0.6563 |
| 1 | rs17027867 | 111853828 | ADD | 1666 | 0.8931 | -1.415 | 0.1569 |
| 1 | rs3818729 | 112793385 | ADD | 1623 | 0.9649 | -0.4299 | 0.6672 |
| 1 | rs4428890 | 116007371 | ADD | 1667 | 0.8533 | -1.36 | 0.1739 |
| 1 | rs688325 | 145543907 | ADD | 1647 | 0.9633 | -0.4124 | 0.68 |
| 1 | rs7416823 | 155653018 | ADD | 1659 | 0.95 | -0.6818 | 0.4954 |
| 1 | rs862991 | 157430988 | ADD | 1663 | 0.9934 | -0.08719 | 0.9305 |
| 1 | rs10919096 | 160529894 | ADD | 1665 | 0.9155 | -1.308 | 0.1909 |
| 1 | rs4657219 | 160906398 | ADD | 1663 | 1.043 | 0.5048 | 0.6137 |
| 1 | rs4657226 | 160919898 | ADD | 1664 | 1.014 | 0.1941 | 0.8461 |
| 1 | rs16843630 | 160936667 | ADD | 1664 | 0.951 | -0.4925 | 0.6223 |
| 1 | rs2684879 | 161069239 | ADD | 1666 | 1.104 | 1.266 | 0.2055 |
| 1 | rs1128977 | 163655753 | ADD | 1660 | 0.9352 | -0.8956 | 0.3704 |
| 1 | rs904224 | 165392933 | ADD | 1667 | 1.152 | 0.4998 | 0.6172 |
| 1 | rs864537 | 165678008 | ADD | 1594 | 0.981 | -0.2586 | 0.7959 |
| 1 | rs2419114 | 166804269 | ADD | 1663 | 1.002 | 0.02931 | 0.9766 |
| 1 | rs12042109 | 168352077 | ADD | 1667 | 0.9254 | -1.077 | 0.2815 |
| 1 | rs7547439 | 173697478 | ADD | 1665 | 1.015 | 0.1764 | 0.86 |
| 1 | rs12035381 | 175915356 | ADD | 1667 | 0.9658 | -0.3918 | 0.6952 |
| 1 | rs2104212 | 175917833 | ADD | 1655 | 1.044 | 0.4973 | 0.6189 |
| 1 | rs12409286 | 176237770 | ADD | 1640 | 1.068 | 0.9107 | 0.3624 |
| 1 | rs10753199 | 177826255 | ADD | 1664 | 0.8934 | -1.675 | 0.09389 |
| 1 | rs6670140 | 179568242 | ADD | 1597 | 0.9767 | -0.3279 | 0.743 |
| 1 | rs546191 | 180019653 | ADD | 1665 | 1.035 | 0.4493 | 0.6532 |
| 1 | rs9286844 | 180094186 | ADD | 1666 | 1.098 | 1.305 | 0.1917 |
| 1 | rs630341 | 180094612 | ADD | 1632 | 1.032 | 0.4488 | 0.6535 |
| 1 | rs1321996 | 183782084 | ADD | 1665 | 0.9797 | -0.3019 | 0.7628 |
| 1 | rs4650754 | 186338589 | ADD | 1622 | 1.05 | 0.6904 | 0.4899 |
| 1 | rs10919814 | 198371595 | ADD | 1667 | 1.025 | 0.319 | 0.7498 |
| 1 | rs6427830 | 198731426 | ADD | 1666 | 0.9032 | -1.34 | 0.1804 |
| 1 | rs16849710 | 200373420 | ADD | 1647 | 1.032 | 0.449 | 0.6534 |
| 1 | rs6691378 | 201423745 | ADD | 1667 | 1.072 | 0.7712 | 0.4406 |
| 1 | rs16851314 | 201566903 | ADD | 1666 | 0.9891 | -0.1272 | 0.8988 |
| 1 | rs7540760 | 202001608 | ADD | 1645 | 1.122 | 1.288 | 0.1976 |
| 1 | rs3795579 | 202336461 | ADD | 1667 | 0.9666 | -0.4717 | 0.6371 |
| 1 | rs1997034 | 202384983 | ADD | 1663 | 0.9979 | -0.02598 | 0.9793 |
| 1 | rs6693954 | 202399261 | ADD | 1627 | 1.032 | 0.4213 | 0.6735 |
| 1 | rs11240735 | 202625956 | ADD | 1662 | 0.9625 | -0.5361 | 0.5919 |
| 1 | rs3789043 | 202855773 | ADD | 1656 | 1.431 | 1.139 | 0.2549 |
| 1 | rs2275696 | 203193759 | ADD | 1667 | 1.143 | 1.617 | 0.1059 |
| 1 | rs4845112 | 204824480 | ADD | 1666 | 1.073 | 0.5392 | 0.5898 |
| 1 | rs11119107 | 204834561 | ADD | 1667 | 1.074 | 0.9438 | 0.3453 |
| 1 | rs1499585 | 206692354 | ADD | 1666 | 1.089 | 0.7155 | 0.4743 |
| 1 | rs17259881 | 206960459 | ADD | 1667 | 0.9198 | -0.6967 | 0.486 |
| 1 | rs1890844 | 208973862 | ADD | 1644 | 1.047 | 0.6608 | 0.5088 |
| 1 | rs924569 | 209146241 | ADD | 1664 | 1.029 | 0.3771 | 0.7061 |
| 1 | rs4846514 | 214769308 | ADD | 1667 | 1.011 | 0.08626 | 0.9313 |
| 1 | rs1416526 | 215303518 | ADD | 1666 | 0.9846 | -0.1595 | 0.8733 |
| 1 | rs7555255 | 215417006 | ADD | 1664 | 1.165 | 1.64 | 0.1011 |
| 1 | rs12044275 | 219593075 | ADD | 1645 | 0.8736 | -1.819 | 0.06893 |
| 1 | rs2241097 | 221367336 | ADD | 1667 | 1.016 | 0.1958 | 0.8448 |
| 1 | rs10495250 | 225040004 | ADD | 1663 | 0.9572 | -0.5174 | 0.6049 |
| 1 | rs6661550 | 225159485 | ADD | 1666 | 0.9933 | -0.08636 | 0.9312 |
| 1 | rs1582114 | 226082993 | ADD | 1658 | 1.072 | 0.986 | 0.3241 |
| 1 | rs644850 | 227208556 | ADD | 1667 | 1.054 | 0.5454 | 0.5855 |
| 1 | rs7550262 | 228315362 | ADD | 1667 | 1.127 | 1.385 | 0.1661 |
| 1 | rs11122458 | 228375478 | ADD | 1615 | 0.9423 | -0.6917 | 0.4891 |
| 1 | rs6541320 | 228750026 | ADD | 1645 | 1.045 | 0.5229 | 0.601 |
| 1 | rs35225245 | 231576204 | ADD | 1662 | 0.825 | -1.633 | 0.1024 |
| 1 | rs10797461 | 231687211 | ADD | 1664 | 1.067 | 0.7816 | 0.4344 |
| 1 | rs6689479 | 234362945 | ADD | 1613 | 1.114 | 1.064 | 0.2873 |
| 1 | rs10802704 | 236766283 | ADD | 1663 | 1.114 | 0.8125 | 0.4165 |
| 1 | rs560229 | 236792385 | ADD | 1649 | 1.098 | 0.8965 | 0.37 |
| 1 | rs7542176 | 237008960 | ADD | 1601 | 1.038 | 0.4588 | 0.6464 |
| 1 | rs1509701 | 237281106 | ADD | 1666 | 1.054 | 0.5807 | 0.5614 |
| 1 | rs6686630 | 238837411 | ADD | 1663 | 1.056 | 0.7848 | 0.4325 |
| 1 | rs10465632 | 239386990 | ADD | 1658 | 1.002 | 0.02301 | 0.9816 |
| 1 | rs316901 | 240477737 | ADD | 1666 | 0.9306 | -0.8829 | 0.3773 |
| 1 | rs2796135 | 240622572 | ADD | 1666 | 1.029 | 0.3441 | 0.7308 |
| 1 | rs4658649 | 242921444 | ADD | 1667 | 1.042 | 0.5338 | 0.5935 |
| 2 | rs17247373 | 2114316 | ADD | 1591 | 0.7288 | -1.805 | 0.07104 |
| 2 | rs7572345 | 8697275 | ADD | 1663 | 1.058 | 0.7952 | 0.4265 |
| 2 | rs4668685 | 10457837 | ADD | 1666 | 0.9399 | -0.8606 | 0.3895 |
| 2 | rs4669613 | 10791963 | ADD | 1664 | 1.011 | 0.1315 | 0.8954 |
| 2 | rs735206 | 11069048 | ADD | 1664 | 1.062 | 0.8527 | 0.3938 |
| 2 | rs2716628 | 11933214 | ADD | 1638 | 1.032 | 0.4186 | 0.6755 |
| 2 | rs949981 | 12506659 | ADD | 1660 | 1.034 | 0.4624 | 0.6438 |
| 2 | rs875077 | 23438070 | ADD | 1658 | 1.051 | 0.6778 | 0.4979 |
| 2 | rs17445645 | 23627453 | ADD | 1667 | 1.033 | 0.4069 | 0.6841 |
| 2 | rs935172 | 26657751 | ADD | 1660 | 1.043 | 0.6037 | 0.546 |
| 2 | rs10195538 | 37770867 | ADD | 1655 | 0.9045 | -1.458 | 0.1449 |
| 2 | rs11901530 | 39964883 | ADD | 1653 | 0.987 | -0.1909 | 0.8486 |
| 2 | rs17024614 | 40006422 | ADD | 1667 | 1.044 | 0.3583 | 0.7201 |
| 2 | rs7569588 | 45273586 | ADD | 1667 | 1.109 | 1.458 | 0.1449 |
| 2 | rs11125038 | 45949960 | ADD | 1664 | 0.9241 | -1.081 | 0.2798 |
| 2 | rs1868271 | 46170852 | ADD | 1663 | 1.076 | 1.032 | 0.3021 |
| 2 | rs698840 | 49489185 | ADD | 1667 | 1.1 | 1.086 | 0.2777 |
| 2 | rs9309200 | 50870615 | ADD | 1665 | 1.145 | 1.681 | 0.09272 |
| 2 | rs1451465 | 52859646 | ADD | 1635 | 0.948 | -0.7045 | 0.4811 |
| 2 | rs2034254 | 53212457 | ADD | 1667 | 0.9658 | -0.4274 | 0.6691 |
| 2 | rs7564091 | 54871341 | ADD | 1666 | 0.8789 | -1.132 | 0.2578 |
| 2 | rs2567969 | 54895981 | ADD | 1621 | 1.05 | 0.4677 | 0.64 |
| 2 | rs10490098 | 58983445 | ADD | 1623 | 1.21 | 1.745 | 0.08094 |
| 2 | rs7573672 | 59008609 | ADD | 1622 | 1.188 | 2.345 | 0.01901 |
| 2 | rs4672274 | 59090006 | ADD | 1654 | 0.9986 | -0.01997 | 0.9841 |
| 2 | rs10205398 | 62533657 | ADD | 1594 | 0.859 | -1.58 | 0.1141 |
| 2 | rs17029079 | 64589576 | ADD | 1601 | 0.9613 | -0.1545 | 0.8772 |
| 2 | rs6733507 | 64789976 | ADD | 1667 | 1.135 | 1.581 | 0.1138 |
| 2 | rs6746740 | 68383186 | ADD | 1653 | 0.9822 | -0.2559 | 0.798 |
| 2 | rs11895277 | 71931468 | ADD | 1664 | 1.027 | 0.292 | 0.7703 |
| 2 | rs10199560 | 74296559 | ADD | 1664 | 1.34 | 2.05 | 0.04041 |
| 2 | rs11903030 | 78997454 | ADD | 1610 | 0.9445 | -0.6268 | 0.5308 |
| 2 | rs17015868 | 78997707 | ADD | 1640 | 0.9795 | -0.1208 | 0.9038 |
| 2 | rs6711618 | 81339645 | ADD | 1664 | 0.8985 | -1.442 | 0.1492 |
| 2 | rs7589293 | 81437068 | ADD | 1622 | 1.005 | 0.07489 | 0.9403 |
| 2 | rs736711 | 84919756 | ADD | 1663 | 0.9444 | -0.5315 | 0.595 |
| 2 | rs17618119 | 86196827 | ADD | 1666 | 1.064 | 0.6022 | 0.5471 |
| 2 | rs12617962 | 86366613 | ADD | 1662 | 0.8063 | -1.091 | 0.2754 |
| 2 | rs1659248 | 88416088 | ADD | 1667 | 1 | 0.0001085 | 0.9999 |
| 2 | rs12617019 | 96269295 | ADD | 1635 | 1.098 | 0.9567 | 0.3387 |
| 2 | rs4850905 | 99518797 | ADD | 1667 | 1.131 | 1.446 | 0.1482 |
| 2 | rs1567804 | 100710070 | ADD | 1667 | 1.102 | 1.4 | 0.1615 |
| 2 | rs17020852 | 104717323 | ADD | 1667 | 0.9507 | -0.6703 | 0.5027 |
| 2 | rs17687727 | 105232399 | ADD | 1666 | 0.9988 | -0.01367 | 0.9891 |
| 2 | rs4374396 | 105390883 | ADD | 1666 | 1.098 | 1.288 | 0.1978 |
| 2 | rs6543317 | 105526930 | ADD | 1650 | 0.9623 | -0.4978 | 0.6186 |
| 2 | rs1037381 | 105669675 | ADD | 1634 | 1.012 | 0.1661 | 0.8681 |
| 2 | rs6746088 | 107353467 | ADD | 1664 | 1.201 | 2.674 | 0.007489 |
| 2 | rs10496417 | 107998161 | ADD | 1667 | 1.106 | 1.425 | 0.1541 |
| 2 | rs967895 | 111517873 | ADD | 1666 | 1.003 | 0.04842 | 0.9614 |
| 2 | rs11680181 | 113151729 | ADD | 1665 | 0.9831 | -0.2522 | 0.8009 |
| 2 | rs4849101 | 113161308 | ADD | 1659 | 0.9461 | -0.7864 | 0.4316 |
| 2 | rs4251961 | 113590938 | ADD | 1663 | 0.9773 | -0.3087 | 0.7576 |
| 2 | rs6757604 | 119961173 | ADD | 1620 | 1.149 | 1.993 | 0.0463 |
| 2 | rs2920692 | 119982320 | ADD | 1658 | 0.8311 | -1.039 | 0.2987 |
| 2 | rs2140779 | 121019189 | ADD | 1660 | 0.9618 | -0.3838 | 0.7011 |
| 2 | rs17364261 | 122850905 | ADD | 1664 | 0.876 | -0.4769 | 0.6335 |
| 2 | rs1453678 | 122857073 | ADD | 1667 | 0.9085 | -1.385 | 0.1662 |
| 2 | rs2419554 | 123654025 | ADD | 1653 | 1.138 | 0.9915 | 0.3214 |
| 2 | rs12990473 | 124170989 | ADD | 1666 | 1.205 | 1.976 | 0.04817 |
| 2 | rs1820931 | 124875246 | ADD | 1667 | 1.14 | 1.887 | 0.05922 |
| 2 | rs17321135 | 125176631 | ADD | 1663 | 0.9841 | -0.2259 | 0.8213 |
| 2 | rs13033217 | 129707339 | ADD | 1665 | 0.9766 | -0.341 | 0.7331 |
| 2 | rs3108980 | 130146561 | ADD | 1663 | 1.061 | 0.8494 | 0.3956 |
| 2 | rs12691834 | 133668510 | ADD | 1620 | 1.058 | 0.7217 | 0.4705 |
| 2 | rs7561228 | 143982974 | ADD | 1617 | 1.194 | 2.194 | 0.02821 |
| 2 | rs4664645 | 153665310 | ADD | 1667 | 0.9227 | -0.9531 | 0.3405 |
| 2 | rs2881691 | 153739928 | ADD | 1665 | 0.9161 | -0.8347 | 0.4039 |
| 2 | rs2034710 | 166175597 | ADD | 1667 | 0.9824 | -0.2544 | 0.7992 |
| 2 | rs836692 | 168353652 | ADD | 1666 | 1.02 | 0.2862 | 0.7747 |
| 2 | rs2288331 | 170300955 | ADD | 1662 | 1.027 | 0.2925 | 0.7699 |
| 2 | rs970797 | 176820065 | ADD | 1656 | 0.9952 | -0.0689 | 0.9451 |
| 2 | rs6433931 | 182348106 | ADD | 1629 | 1.012 | 0.1651 | 0.8688 |
| 2 | rs10497616 | 183986209 | ADD | 1650 | 0.8562 | -1.724 | 0.08464 |
| 2 | rs1517843 | 192113695 | ADD | 1645 | 0.9023 | -1.44 | 0.15 |
| 2 | rs7558259 | 192587245 | ADD | 1667 | 1.04 | 0.5781 | 0.5632 |
| 2 | rs12619354 | 193711027 | ADD | 1665 | 0.929 | -1.01 | 0.3126 |
| 2 | rs12614240 | 199461919 | ADD | 1662 | 0.9575 | -0.6249 | 0.532 |
| 2 | rs166849 | 200487111 | ADD | 1650 | 1.052 | 0.7199 | 0.4716 |
| 2 | rs1986415 | 201199805 | ADD | 1664 | 0.9194 | -0.7563 | 0.4495 |
| 2 | rs4321346 | 202560461 | ADD | 1666 | 1.106 | 1.291 | 0.1967 |
| 2 | rs35321733 | 204775754 | ADD | 1667 | 0.84 | -1.863 | 0.06248 |
| 2 | rs6735824 | 205094487 | ADD | 1667 | 0.9671 | -0.4331 | 0.6649 |
| 2 | rs7606468 | 205853078 | ADD | 1666 | 1.013 | 0.1759 | 0.8604 |
| 2 | rs2704638 | 205866562 | ADD | 1663 | 0.965 | -0.3755 | 0.7073 |
| 2 | rs12694115 | 209180827 | ADD | 1666 | 0.9093 | -1.36 | 0.1738 |
| 2 | rs7421820 | 212296872 | ADD | 1667 | 0.9288 | -0.7829 | 0.4337 |
| 2 | rs3770536 | 216547065 | ADD | 1659 | 1.128 | 1.715 | 0.08643 |
| 2 | rs3770564 | 216579725 | ADD | 1666 | 0.8395 | -1.771 | 0.0765 |
| 2 | rs768921 | 218372224 | ADD | 1623 | 1.126 | 0.8304 | 0.4063 |
| 2 | rs2680835 | 222174010 | ADD | 1662 | 1.002 | 0.03585 | 0.9714 |
| 2 | rs6726046 | 233951960 | ADD | 1656 | 1.088 | 1.207 | 0.2276 |
| 2 | rs6746703 | 235727802 | ADD | 1665 | 1.026 | 0.3275 | 0.7433 |
| 2 | rs7598559 | 238100137 | ADD | 1667 | 0.9675 | -0.3559 | 0.7219 |
| 2 | rs11124235 | 240417716 | ADD | 1658 | 1.109 | 1.42 | 0.1556 |
| 2 | rs4676431 | 241105074 | ADD | 1657 | 0.8891 | -1.191 | 0.2336 |
| 2 | rs11676358 | 241210447 | ADD | 1667 | 0.8927 | -1.172 | 0.2414 |
| 3 | rs1317443 | 1685731 | ADD | 1666 | 1.03 | 0.35 | 0.7263 |
| 3 | rs11717189 | 3220960 | ADD | 1667 | 0.9325 | -0.9473 | 0.3435 |
| 3 | rs17215946 | 6181901 | ADD | 1587 | 0.7677 | -1.216 | 0.2239 |
| 3 | rs6790803 | 6211038 | ADD | 1666 | 1.093 | 1.304 | 0.1923 |
| 3 | rs3967024 | 6297086 | ADD | 1651 | 0.9989 | -0.01559 | 0.9876 |
| 3 | rs156168 | 6398320 | ADD | 1658 | 0.8617 | -1.746 | 0.08075 |
| 3 | rs6769829 | 6492587 | ADD | 1667 | 1.521 | 2.237 | 0.02529 |
| 3 | rs456835 | 6886400 | ADD | 1636 | 1.053 | 0.72 | 0.4715 |
| 3 | rs670764 | 7001341 | ADD | 1661 | 0.9824 | -0.258 | 0.7964 |
| 3 | rs1874981 | 7147721 | ADD | 1664 | 0.9463 | -0.4822 | 0.6297 |
| 3 | rs3749448 | 7163116 | ADD | 1649 | 1.082 | 0.9702 | 0.3319 |
| 3 | rs1106486 | 7537364 | ADD | 1661 | 1.083 | 1.075 | 0.2825 |
| 3 | rs301556 | 11766680 | ADD | 1665 | 1.062 | 0.8405 | 0.4006 |
| 3 | rs357116 | 13296852 | ADD | 1652 | 1.028 | 0.4007 | 0.6887 |
| 3 | rs9881693 | 14286277 | ADD | 1638 | 1.217 | 2.212 | 0.02697 |
| 3 | rs17318350 | 14610052 | ADD | 1667 | 0.9182 | -1.057 | 0.2904 |
| 3 | rs17040258 | 14771148 | ADD | 1663 | 1.087 | 0.6156 | 0.5382 |
| 3 | rs9813959 | 16685683 | ADD | 1665 | 1.098 | 1.183 | 0.2368 |
| 3 | rs9881375 | 17110188 | ADD | 1662 | 1.038 | 0.5153 | 0.6064 |
| 3 | rs9825481 | 21072117 | ADD | 1665 | 0.9814 | -0.2723 | 0.7854 |
| 3 | rs4102411 | 21600105 | ADD | 1667 | 0.9186 | -1.093 | 0.2745 |
| 3 | rs296160 | 22808291 | ADD | 1666 | 1.019 | 0.1994 | 0.842 |
| 3 | rs184174 | 22885441 | ADD | 1666 | 0.9219 | -0.8405 | 0.4007 |
| 3 | rs9881863 | 24804092 | ADD | 1664 | 1.109 | 0.9963 | 0.3191 |
| 3 | rs7616467 | 25483432 | ADD | 1663 | 1.175 | 2.24 | 0.02508 |
| 3 | rs17665280 | 29312488 | ADD | 1667 | 0.9286 | -1.001 | 0.3169 |
| 3 | rs11706573 | 30052486 | ADD | 1660 | 0.9809 | -0.2302 | 0.818 |
| 3 | rs11129399 | 30370955 | ADD | 1667 | 0.8855 | -0.9545 | 0.3398 |
| 3 | rs11921928 | 31709993 | ADD | 1667 | 0.9989 | -0.01431 | 0.9886 |
| 3 | rs13096226 | 32299121 | ADD | 1651 | 0.9287 | -0.5528 | 0.5804 |
| 3 | rs4678523 | 33012725 | ADD | 1659 | 1.039 | 0.5088 | 0.6109 |
| 3 | rs10865916 | 42229487 | ADD | 1665 | 1.068 | 0.9315 | 0.3516 |
| 3 | rs4432683 | 54255177 | ADD | 1667 | 1.189 | 1.449 | 0.1473 |
| 3 | rs6445693 | 54550810 | ADD | 1660 | 0.9331 | -0.8886 | 0.3742 |
| 3 | rs6767040 | 55545992 | ADD | 1667 | 1.102 | 0.6517 | 0.5146 |
| 3 | rs11130620 | 58203122 | ADD | 1667 | 1.001 | 0.01 | 0.992 |
| 3 | rs1982758 | 58524687 | ADD | 1655 | 0.9143 | -1.272 | 0.2032 |
| 3 | rs9813383 | 59406382 | ADD | 1665 | 1.282 | 2.36 | 0.01825 |
| 3 | rs49411 | 59732615 | ADD | 1640 | 0.8522 | -2.277 | 0.02276 |
| 3 | rs107342 | 60511592 | ADD | 1599 | 1.079 | 0.5952 | 0.5517 |
| 3 | rs2594141 | 60783325 | ADD | 1664 | 1.004 | 0.04583 | 0.9634 |
| 3 | rs4129352 | 60968955 | ADD | 1667 | 1.348 | 1.43 | 0.1528 |
| 3 | rs33080 | 61926723 | ADD | 1656 | 0.9834 | -0.1989 | 0.8423 |
| 3 | rs6764198 | 62092225 | ADD | 1665 | 0.9387 | -0.9 | 0.3681 |
| 3 | rs9968060 | 62446322 | ADD | 1666 | 1.032 | 0.4683 | 0.6396 |
| 3 | rs9871763 | 62452739 | ADD | 1660 | 1.142 | 1.646 | 0.09968 |
| 3 | rs1376917 | 62473418 | ADD | 1667 | 1.091 | 0.8436 | 0.3989 |
| 3 | rs2367600 | 63046040 | ADD | 1665 | 1.026 | 0.3742 | 0.7082 |
| 3 | rs2122797 | 63424246 | ADD | 1665 | 1.031 | 0.3422 | 0.7322 |
| 3 | rs924067 | 63619734 | ADD | 1660 | 0.9766 | -0.2782 | 0.7808 |
| 3 | rs1221474 | 63719773 | ADD | 1662 | 1.099 | 0.9929 | 0.3208 |
| 3 | rs2194093 | 64719092 | ADD | 1619 | 0.9697 | -0.4031 | 0.6869 |
| 3 | rs6549127 | 68567687 | ADD | 1667 | 1.02 | 0.2276 | 0.82 |
| 3 | rs12489786 | 69870758 | ADD | 1615 | 0.9604 | -0.203 | 0.8391 |
| 3 | rs7616034 | 72034471 | ADD | 1667 | 1.021 | 0.2676 | 0.789 |
| 3 | rs2874710 | 72039132 | ADD | 1634 | 1.081 | 1.139 | 0.2547 |
| 3 | rs9832640 | 72045237 | ADD | 1659 | 1.17 | 1.521 | 0.1282 |
| 3 | rs1579905 | 74711280 | ADD | 1658 | 1.009 | 0.1275 | 0.8985 |
| 3 | rs11712680 | 75091709 | ADD | 1667 | 0.9437 | -0.6079 | 0.5432 |
| 3 | rs17018482 | 81212634 | ADD | 1663 | 1.153 | 1.601 | 0.1094 |
| 3 | rs12638798 | 85634510 | ADD | 1632 | 1.042 | 0.5622 | 0.574 |
| 3 | rs9820995 | 86540225 | ADD | 1657 | 1.01 | 0.1295 | 0.897 |
| 3 | rs7103 | 99781290 | ADD | 1664 | 1.041 | 0.553 | 0.5802 |
| 3 | rs13083225 | 112486553 | ADD | 1667 | 1.018 | 0.189 | 0.8501 |
| 3 | rs4510355 | 115615506 | ADD | 1657 | 0.9436 | -0.7919 | 0.4284 |
| 3 | rs1401072 | 117764397 | ADD | 1664 | 1.088 | 1.091 | 0.2754 |
| 3 | rs10934409 | 118834895 | ADD | 1636 | 0.8232 | -1.629 | 0.1034 |
| 3 | rs2241994 | 120592696 | ADD | 1663 | 0.9718 | -0.2563 | 0.7977 |
| 3 | rs6773050 | 120606504 | ADD | 1660 | 1.102 | 1.408 | 0.159 |
| 3 | rs17295401 | 124611103 | ADD | 1667 | 0.8618 | -1.757 | 0.07884 |
| 3 | rs33926252 | 125752222 | ADD | 1666 | 0.8136 | -1.69 | 0.09098 |
| 3 | rs684030 | 126345558 | ADD | 1667 | 1.013 | 0.1858 | 0.8526 |
| 3 | rs4679251 | 127704774 | ADD | 1663 | 0.9549 | -0.656 | 0.5118 |
| 3 | rs9855392 | 131415936 | ADD | 1666 | 0.8147 | -2.339 | 0.01933 |
| 3 | rs17748304 | 131853438 | ADD | 1664 | 0.9334 | -0.4845 | 0.6281 |
| 3 | rs7610027 | 132933577 | ADD | 1666 | 0.9899 | -0.1005 | 0.9199 |
| 3 | rs6802764 | 133215110 | ADD | 1648 | 0.9753 | -0.3264 | 0.7441 |
| 3 | rs9840108 | 135039035 | ADD | 1667 | 0.944 | -0.8241 | 0.4099 |
| 3 | rs1610111 | 138784424 | ADD | 1665 | 1.091 | 1.036 | 0.3 |
| 3 | rs1353887 | 144182963 | ADD | 1623 | 1.012 | 0.1273 | 0.8987 |
| 3 | rs6440165 | 144580138 | ADD | 1667 | 1.026 | 0.2992 | 0.7648 |
| 3 | rs6782465 | 144584003 | ADD | 1666 | 0.9014 | -0.9343 | 0.3502 |
| 3 | rs905142 | 144637716 | ADD | 1662 | 1.045 | 0.5926 | 0.5534 |
| 3 | rs4355248 | 149440411 | ADD | 1655 | 1.078 | 0.9244 | 0.3553 |
| 3 | rs2061201 | 152115464 | ADD | 1665 | 1.011 | 0.1586 | 0.874 |
| 3 | rs2870518 | 152427933 | ADD | 1659 | 0.8913 | -1.457 | 0.1452 |
| 3 | rs3773621 | 152479124 | ADD | 1650 | 0.9673 | -0.4485 | 0.6538 |
| 3 | rs7647733 | 153215331 | ADD | 1643 | 0.9674 | -0.3805 | 0.7036 |
| 3 | rs10936111 | 159069541 | ADD | 1664 | 1.033 | 0.4622 | 0.6439 |
| 3 | rs11928737 | 161414110 | ADD | 1662 | 0.9779 | -0.1892 | 0.8499 |
| 3 | rs1605389 | 169456755 | ADD | 1662 | 0.9977 | -0.03045 | 0.9757 |
| 3 | rs4894643 | 172463751 | ADD | 1637 | 1.113 | 1.271 | 0.2039 |
| 3 | rs9826915 | 173105193 | ADD | 1659 | 1.024 | 0.3253 | 0.7449 |
| 3 | rs4894559 | 173716063 | ADD | 1666 | 0.9774 | -0.2848 | 0.7758 |
| 3 | rs374777 | 173814679 | ADD | 1656 | 1.016 | 0.2056 | 0.8371 |
| 3 | rs2268933 | 180938305 | ADD | 1663 | 1.052 | 0.6784 | 0.4975 |
| 3 | rs6786156 | 185951807 | ADD | 1667 | 0.879 | -1.807 | 0.07074 |
| 3 | rs4686880 | 188648468 | ADD | 1667 | 1.053 | 0.4931 | 0.622 |
| 3 | rs2600998 | 188766542 | ADD | 1662 | 1.023 | 0.3171 | 0.7512 |
| 3 | rs4686917 | 189208807 | ADD | 1663 | 1.014 | 0.1954 | 0.8451 |
| 3 | rs1711001 | 189361698 | ADD | 1605 | 1.064 | 0.8514 | 0.3945 |
| 3 | rs16863396 | 189715374 | ADD | 1664 | 0.9853 | -0.2089 | 0.8345 |
| 3 | rs7636839 | 190839635 | ADD | 1667 | 0.9476 | -0.7746 | 0.4386 |
| 3 | rs7641791 | 191147311 | ADD | 1667 | 1.046 | 0.6473 | 0.5175 |
| 3 | rs7630486 | 191284685 | ADD | 1666 | 1.026 | 0.1858 | 0.8526 |
| 3 | rs6805912 | 194936427 | ADD | 1648 | 0.9235 | -1.092 | 0.2747 |
| 3 | rs7652843 | 196036174 | ADD | 1667 | 1.073 | 1.008 | 0.3137 |
| 3 | rs2898596 | 196038502 | ADD | 1654 | 1.184 | 1.165 | 0.244 |
| 3 | rs2030534 | 197916564 | ADD | 1666 | 1.059 | 0.7728 | 0.4397 |
| 4 | rs3775261 | 4914646 | ADD | 1620 | 1.124 | 1.486 | 0.1372 |
| 4 | rs4464513 | 4918223 | ADD | 1667 | 0.9375 | -0.9205 | 0.3573 |
| 4 | rs9291090 | 5441538 | ADD | 1663 | 1.043 | 0.256 | 0.798 |
| 4 | rs11939527 | 5812267 | ADD | 1667 | 0.9704 | -0.3758 | 0.7071 |
| 4 | rs4443226 | 5975530 | ADD | 1607 | 0.9918 | -0.1177 | 0.9063 |
| 4 | rs7698546 | 6670279 | ADD | 1665 | 0.9498 | -0.4117 | 0.6806 |
| 4 | rs12506309 | 7545963 | ADD | 1666 | 1.057 | 0.815 | 0.4151 |
| 4 | rs6821770 | 7783705 | ADD | 1654 | 1.123 | 1.44 | 0.1499 |
| 4 | rs503506 | 12758220 | ADD | 1667 | 1.003 | 0.04202 | 0.9665 |
| 4 | rs12498177 | 15523899 | ADD | 1660 | 0.9013 | -1.235 | 0.217 |
| 4 | rs4698434 | 15577672 | ADD | 1665 | 0.9648 | -0.5204 | 0.6028 |
| 4 | rs715213 | 19877013 | ADD | 1611 | 0.7414 | -1.556 | 0.1197 |
| 4 | rs1488290 | 26806884 | ADD | 1657 | 1.01 | 0.1381 | 0.8902 |
| 4 | rs10517141 | 27961515 | ADD | 1664 | 0.9769 | -0.2824 | 0.7777 |
| 4 | rs10050320 | 31585804 | ADD | 1616 | 0.9517 | -0.6857 | 0.4929 |
| 4 | rs12641222 | 37831102 | ADD | 1663 | 1.129 | 1.472 | 0.141 |
| 4 | rs4565081 | 38429380 | ADD | 1665 | 1.105 | 1.241 | 0.2146 |
| 4 | rs1648082 | 43380218 | ADD | 1665 | 0.9971 | -0.03189 | 0.9746 |
| 4 | rs3934674 | 46707895 | ADD | 1666 | 1.064 | 0.8321 | 0.4054 |
| 4 | rs11133427 | 56733218 | ADD | 1620 | 1.113 | 1.488 | 0.1366 |
| 4 | rs6842825 | 56819933 | ADD | 1658 | 1.2 | 1.248 | 0.2121 |
| 4 | rs1107674 | 57533447 | ADD | 1665 | 1.068 | 0.9115 | 0.362 |
| 4 | rs12648062 | 61349372 | ADD | 1663 | 1.102 | 0.8264 | 0.4086 |
| 4 | rs725761 | 61594457 | ADD | 1607 | 1.179 | 2.158 | 0.03093 |
| 4 | rs6852744 | 67842380 | ADD | 1596 | 0.9309 | -1.018 | 0.3087 |
| 4 | rs900791 | 68153050 | ADD | 1648 | 0.8961 | -1.207 | 0.2274 |
| 4 | rs959636 | 68446389 | ADD | 1661 | 0.9391 | -0.8235 | 0.4102 |
| 4 | rs168482 | 68491562 | ADD | 1628 | 0.9769 | -0.2871 | 0.7741 |
| 4 | rs11735430 | 71060137 | ADD | 1662 | 1.16 | 2.159 | 0.03085 |
| 4 | rs11249497 | 71272010 | ADD | 1667 | 1.028 | 0.262 | 0.7933 |
| 4 | rs6446945 | 72010366 | ADD | 1667 | 1.225 | 1.269 | 0.2045 |
| 4 | rs11097403 | 77678624 | ADD | 1667 | 0.9922 | -0.1138 | 0.9094 |
| 4 | rs6849534 | 78292679 | ADD | 1657 | 0.9904 | -0.1372 | 0.8909 |
| 4 | rs6857600 | 89285099 | ADD | 1667 | 0.9827 | -0.2156 | 0.8293 |
| 4 | rs9996250 | 105911383 | ADD | 1658 | 1.047 | 0.53 | 0.5961 |
| 4 | rs902444 | 106142496 | ADD | 1667 | 1.065 | 0.9021 | 0.367 |
| 4 | rs12507470 | 109431427 | ADD | 1663 | 0.9324 | -0.8393 | 0.4013 |
| 4 | rs219456 | 109540178 | ADD | 1667 | 1.156 | 2.043 | 0.04103 |
| 4 | rs924758 | 112639008 | ADD | 1666 | 1.128 | 1.712 | 0.08688 |
| 4 | rs1081010 | 114951978 | ADD | 1667 | 1.009 | 0.1051 | 0.9163 |
| 4 | rs4645284 | 118614104 | ADD | 1664 | 0.9295 | -1.062 | 0.2881 |
| 4 | rs17343008 | 121772276 | ADD | 1666 | 0.9579 | -0.5697 | 0.5689 |
| 4 | rs9996039 | 125764176 | ADD | 1665 | 0.9236 | -1.004 | 0.3156 |
| 4 | rs1543936 | 125965670 | ADD | 1630 | 1.033 | 0.4614 | 0.6445 |
| 4 | rs1548111 | 127423698 | ADD | 1656 | 1.098 | 1.246 | 0.2127 |
| 4 | rs2088797 | 132776329 | ADD | 1665 | 1.033 | 0.4515 | 0.6516 |
| 4 | rs10519357 | 136539097 | ADD | 1663 | 1.022 | 0.2514 | 0.8015 |
| 4 | rs13109827 | 137863382 | ADD | 1653 | 1.064 | 0.6573 | 0.511 |
| 4 | rs2406747 | 138586803 | ADD | 1665 | 1.077 | 1.025 | 0.3052 |
| 4 | rs12498882 | 138600718 | ADD | 1665 | 1.218 | 2.843 | 0.004476 |
| 4 | rs706349 | 140819757 | ADD | 1661 | 1.152 | 1.955 | 0.0506 |
| 4 | rs1521496 | 141288597 | ADD | 1663 | 0.9509 | -0.5312 | 0.5953 |
| 4 | rs6841736 | 146876245 | ADD | 1659 | 1.004 | 0.04051 | 0.9677 |
| 4 | rs988163 | 146984333 | ADD | 1667 | 0.9667 | -0.4862 | 0.6268 |
| 4 | rs17770092 | 147701051 | ADD | 1630 | 1.103 | 0.8951 | 0.3707 |
| 4 | rs11737660 | 149243863 | ADD | 1667 | 0.842 | -1.991 | 0.04653 |
| 4 | rs569802 | 153045050 | ADD | 1642 | 1.04 | 0.5605 | 0.5751 |
| 4 | rs3864176 | 154686389 | ADD | 1662 | 1.016 | 0.2257 | 0.8215 |
| 4 | rs10857274 | 155526876 | ADD | 1655 | 1.025 | 0.3701 | 0.7113 |
| 4 | rs4547780 | 155652394 | ADD | 1656 | 1.018 | 0.2736 | 0.7844 |
| 4 | rs17035016 | 157783358 | ADD | 1655 | 1.062 | 0.8691 | 0.3848 |
| 4 | rs2699027 | 160568882 | ADD | 1663 | 1.022 | 0.3022 | 0.7625 |
| 4 | rs17473073 | 164680095 | ADD | 1663 | 1.064 | 0.4014 | 0.6881 |
| 4 | rs2714807 | 166810771 | ADD | 1667 | 1.082 | 1.166 | 0.2436 |
| 4 | rs17504069 | 166812299 | ADD | 1650 | 0.8909 | -1.603 | 0.109 |
| 4 | rs13111857 | 166884068 | ADD | 1667 | 1.118 | 1.184 | 0.2364 |
| 4 | rs12498755 | 168291597 | ADD | 1655 | 0.9979 | -0.02636 | 0.979 |
| 4 | rs1609380 | 171577472 | ADD | 1646 | 0.8547 | -1.846 | 0.06488 |
| 4 | rs17056275 | 172011828 | ADD | 1663 | 1.001 | 0.005885 | 0.9953 |
| 4 | rs1365625 | 175707244 | ADD | 1662 | 1.096 | 1.249 | 0.2115 |
| 4 | rs2715399 | 179574993 | ADD | 1661 | 0.9713 | -0.3966 | 0.6917 |
| 4 | rs4861798 | 180861045 | ADD | 1666 | 1.023 | 0.3236 | 0.7463 |
| 4 | rs7659727 | 182034590 | ADD | 1667 | 1.055 | 0.7838 | 0.4332 |
| 4 | rs6813301 | 183096346 | ADD | 1667 | 1.023 | 0.2753 | 0.7831 |
| 4 | rs17072756 | 183232158 | ADD | 1660 | 1.085 | 1.01 | 0.3126 |
| 4 | rs335067 | 183285690 | ADD | 1666 | 1.016 | 0.2329 | 0.8158 |
| 4 | rs9985535 | 183755240 | ADD | 1630 | 1.135 | 1.828 | 0.06752 |
| 4 | rs1511674 | 184543507 | ADD | 1665 | 1.067 | 0.6501 | 0.5156 |
| 4 | rs7670109 | 184691188 | ADD | 1662 | 1.124 | 1.687 | 0.09163 |
| 4 | rs12506141 | 186296887 | ADD | 1598 | 0.8873 | -1.687 | 0.09162 |
| 4 | rs2046535 | 186307426 | ADD | 1667 | 0.8612 | -2.17 | 0.02997 |
| 4 | rs4862644 | 187294806 | ADD | 1643 | 1.057 | 0.8273 | 0.408 |
| 5 | rs4246740 | 1239086 | ADD | 1642 | 0.9294 | -1.029 | 0.3036 |
| 5 | rs7737692 | 1514167 | ADD | 1664 | 1.29 | 3.487 | 0.0004887 |
| 5 | rs7448302 | 2202503 | ADD | 1662 | 0.9288 | -0.8206 | 0.4119 |
| 5 | rs7735656 | 2203273 | ADD | 1667 | 0.9703 | -0.3969 | 0.6915 |
| 5 | rs6864829 | 2836869 | ADD | 1665 | 1.063 | 0.7801 | 0.4354 |
| 5 | rs4476727 | 3340958 | ADD | 1666 | 0.943 | -0.7442 | 0.4567 |
| 5 | rs156251 | 5116242 | ADD | 1665 | 1.063 | 0.6694 | 0.5032 |
| 5 | rs3846567 | 6076338 | ADD | 1667 | 1.141 | 1.462 | 0.1437 |
| 5 | rs553169 | 10032084 | ADD | 1663 | 0.9372 | -0.8534 | 0.3934 |
| 5 | rs1911942 | 10211297 | ADD | 1667 | 0.982 | -0.2651 | 0.7909 |
| 5 | rs16901339 | 11222601 | ADD | 1666 | 0.9123 | -1.07 | 0.2845 |
| 5 | rs17526653 | 13958149 | ADD | 1667 | 1.495 | 1.428 | 0.1532 |
| 5 | rs10050676 | 14011443 | ADD | 1660 | 1.025 | 0.3137 | 0.7538 |
| 5 | rs26217 | 14587994 | ADD | 1666 | 0.7365 | -2.739 | 0.006159 |
| 5 | rs32199 | 16090234 | ADD | 1645 | 1.066 | 0.9123 | 0.3616 |
| 5 | rs891163 | 16915998 | ADD | 1664 | 1.031 | 0.3406 | 0.7334 |
| 5 | rs2929726 | 17264649 | ADD | 1664 | 0.9807 | -0.2802 | 0.7794 |
| 5 | rs959738 | 18724733 | ADD | 1666 | 0.9925 | -0.04726 | 0.9623 |
| 5 | rs17409624 | 31564490 | ADD | 1667 | 1.064 | 0.9107 | 0.3624 |
| 5 | rs1428256 | 38309217 | ADD | 1662 | 0.907 | -1.069 | 0.2852 |
| 5 | rs17423367 | 38343577 | ADD | 1666 | 0.971 | -0.3229 | 0.7467 |
| 5 | rs391781 | 41300000 | ADD | 1634 | 0.9384 | -0.9281 | 0.3533 |
| 5 | rs27964 | 50188224 | ADD | 1667 | 1.07 | 0.9998 | 0.3174 |
| 5 | rs11741572 | 51404742 | ADD | 1667 | 1.026 | 0.3789 | 0.7047 |
| 5 | rs35920 | 53601968 | ADD | 1666 | 1.086 | 0.7143 | 0.4751 |
| 5 | rs10045084 | 55369682 | ADD | 1654 | 1.125 | 1.596 | 0.1105 |
| 5 | rs182572 | 55488262 | ADD | 1646 | 1.167 | 1.915 | 0.05544 |
| 5 | rs2279980 | 57949136 | ADD | 1658 | 1.228 | 2.609 | 0.009071 |
| 5 | rs1498609 | 58380275 | ADD | 1666 | 1.023 | 0.2878 | 0.7735 |
| 5 | rs4583865 | 71084370 | ADD | 1665 | 0.9665 | -0.3764 | 0.7066 |
| 5 | rs10462509 | 74285663 | ADD | 1634 | 0.8563 | -0.8414 | 0.4001 |
| 5 | rs3935470 | 74387936 | ADD | 1664 | 1.041 | 0.5943 | 0.5523 |
| 5 | rs7707801 | 75866793 | ADD | 1666 | 0.9983 | -0.02508 | 0.98 |
| 5 | rs1982658 | 76535298 | ADD | 1655 | 1.068 | 0.9108 | 0.3624 |
| 5 | rs10075871 | 79533171 | ADD | 1667 | 1.025 | 0.3473 | 0.7283 |
| 5 | rs3762986 | 95796618 | ADD | 1667 | 0.9 | -1.489 | 0.1365 |
| 5 | rs1837253 | 110429771 | ADD | 1666 | 1.11 | 1.318 | 0.1876 |
| 5 | rs17458552 | 110809436 | ADD | 1665 | 1.083 | 1.093 | 0.2743 |
| 5 | rs680878 | 115980173 | ADD | 1637 | 1.042 | 0.5733 | 0.5664 |
| 5 | rs1439609 | 124597237 | ADD | 1667 | 1.012 | 0.1451 | 0.8846 |
| 5 | rs2147866 | 127263913 | ADD | 1667 | 0.981 | -0.2776 | 0.7813 |
| 5 | rs4279383 | 133335005 | ADD | 1663 | 0.9717 | -0.4067 | 0.6842 |
| 5 | rs248504 | 141189651 | ADD | 1666 | 1.104 | 1.162 | 0.2452 |
| 5 | rs1423029 | 145910737 | ADD | 1645 | 1.021 | 0.3011 | 0.7634 |
| 5 | rs4705377 | 149143607 | ADD | 1667 | 0.9395 | -0.8631 | 0.3881 |
| 5 | rs30884 | 149218109 | ADD | 1664 | 0.9183 | -1.175 | 0.2402 |
| 5 | rs216146 | 149426114 | ADD | 1665 | 0.9822 | -0.2535 | 0.7999 |
| 5 | rs1093167 | 153460729 | ADD | 1660 | 1.015 | 0.1564 | 0.8757 |
| 5 | rs2926053 | 162316642 | ADD | 1637 | 0.9883 | -0.09993 | 0.9204 |
| 5 | rs3926173 | 163663585 | ADD | 1666 | 1.077 | 0.8479 | 0.3965 |
| 5 | rs1862370 | 164844511 | ADD | 1666 | 1.089 | 0.5021 | 0.6156 |
| 5 | rs1450629 | 165820253 | ADD | 1666 | 0.9695 | -0.3816 | 0.7028 |
| 5 | rs347980 | 166009784 | ADD | 1664 | 1.055 | 0.6577 | 0.5107 |
| 5 | rs7714651 | 167162881 | ADD | 1665 | 1.025 | 0.2665 | 0.7899 |
| 5 | rs10516050 | 168131879 | ADD | 1666 | 0.975 | -0.2758 | 0.7827 |
| 5 | rs4867879 | 169064953 | ADD | 1654 | 1.017 | 0.2468 | 0.805 |
| 5 | rs17736846 | 169066403 | ADD | 1652 | 1.331 | 1.377 | 0.1684 |
| 5 | rs17669654 | 169136824 | ADD | 1654 | 1.189 | 1.864 | 0.06233 |
| 5 | rs4076077 | 170796114 | ADD | 1614 | 0.9916 | -0.1164 | 0.9074 |
| 5 | rs17681152 | 170881599 | ADD | 1665 | 1.058 | 0.6159 | 0.5379 |
| 5 | rs895312 | 172697004 | ADD | 1659 | 0.8488 | -1.33 | 0.1835 |
| 5 | rs10476071 | 172718311 | ADD | 1666 | 1.033 | 0.4435 | 0.6574 |
| 5 | rs6870544 | 172777221 | ADD | 1667 | 0.9676 | -0.36 | 0.7189 |
| 5 | rs17738166 | 172930584 | ADD | 1666 | 0.9548 | -0.6544 | 0.5128 |
| 5 | rs17076923 | 173543990 | ADD | 1616 | 1.341 | 2.276 | 0.02283 |
| 5 | rs1432621 | 173615579 | ADD | 1667 | 1.168 | 1.728 | 0.08399 |
| 5 | rs2436319 | 173620108 | ADD | 1667 | 1.019 | 0.2613 | 0.7939 |
| 5 | rs4242185 | 174701337 | ADD | 1664 | 0.9827 | -0.2437 | 0.8074 |
| 5 | rs6601221 | 177507027 | ADD | 1667 | 0.8963 | -1.521 | 0.1282 |
| 5 | rs340419 | 178691515 | ADD | 1659 | 1.067 | 0.9205 | 0.3573 |
| 6 | rs9405444 | 1114047 | ADD | 1667 | 1.01 | 0.1252 | 0.9003 |
| 6 | rs9328052 | 1388759 | ADD | 1660 | 1.145 | 1.308 | 0.191 |
| 6 | rs9405484 | 1443456 | ADD | 1641 | 1.116 | 1.252 | 0.2105 |
| 6 | rs3800046 | 1711972 | ADD | 1660 | 1.013 | 0.1914 | 0.8482 |
| 6 | rs2326106 | 2934581 | ADD | 1659 | 1.075 | 0.8633 | 0.388 |
| 6 | rs9504439 | 5539198 | ADD | 1667 | 1.051 | 0.7369 | 0.4612 |
| 6 | rs11756027 | 6077751 | ADD | 1667 | 1.036 | 0.4081 | 0.6832 |
| 6 | rs11969912 | 6120974 | ADD | 1638 | 1.118 | 1.507 | 0.1318 |
| 6 | rs3851513 | 6219160 | ADD | 1654 | 1.111 | 1.187 | 0.2354 |
| 6 | rs2765353 | 6904467 | ADD | 1639 | 1.18 | 1.258 | 0.2085 |
| 6 | rs6922037 | 10177720 | ADD | 1649 | 0.9897 | -0.1384 | 0.8899 |
| 6 | rs1990665 | 13863564 | ADD | 1644 | 0.9999 | -0.0006123 | 0.9995 |
| 6 | rs2619514 | 15843987 | ADD | 1667 | 0.8166 | -1.768 | 0.07704 |
| 6 | rs1144696 | 16850904 | ADD | 1663 | 1.033 | 0.3754 | 0.7074 |
| 6 | rs7744164 | 18278503 | ADD | 1659 | 0.9958 | -0.0604 | 0.9518 |
| 6 | rs760793 | 19916249 | ADD | 1653 | 0.9895 | -0.1058 | 0.9158 |
| 6 | rs973244 | 19934609 | ADD | 1636 | 0.969 | -0.4275 | 0.669 |
| 6 | rs2457336 | 20215406 | ADD | 1667 | 1.003 | 0.04227 | 0.9663 |
| 6 | rs10946425 | 21126634 | ADD | 1652 | 1.003 | 0.04759 | 0.962 |
| 6 | rs10946440 | 21342403 | ADD | 1664 | 1.102 | 1.173 | 0.2408 |
| 6 | rs4236026 | 23351541 | ADD | 1666 | 0.8959 | -1.324 | 0.1855 |
| 6 | rs9393425 | 23764859 | ADD | 1667 | 0.9024 | -0.904 | 0.366 |
| 6 | rs2328208 | 24393550 | ADD | 1667 | 1.069 | 0.8985 | 0.3689 |
| 6 | rs1165200 | 25983191 | ADD | 1664 | 0.9168 | -1.015 | 0.3101 |
| 6 | rs1156457 | 27408559 | ADD | 1665 | 0.9293 | -0.743 | 0.4575 |
| 6 | rs2235358 | 28324006 | ADD | 1666 | 1.152 | 0.7933 | 0.4276 |
| 6 | rs6935041 | 33961697 | ADD | 1658 | 0.9289 | -1.071 | 0.284 |
| 6 | rs1880090 | 35098946 | ADD | 1667 | 1.066 | 0.9104 | 0.3626 |
| 6 | rs6918733 | 37698163 | ADD | 1657 | 1.012 | 0.1738 | 0.862 |
| 6 | rs6901022 | 39414041 | ADD | 1667 | 0.9333 | -0.9288 | 0.353 |
| 6 | rs742552 | 52234664 | ADD | 1655 | 1.115 | 1.481 | 0.1387 |
| 6 | rs2167460 | 56992410 | ADD | 1663 | 1.02 | 0.2322 | 0.8164 |
| 6 | rs2350516 | 65816297 | ADD | 1663 | 1.252 | 1.983 | 0.04737 |
| 6 | rs3798974 | 69732559 | ADD | 1649 | 1.038 | 0.4959 | 0.62 |
| 6 | rs11753467 | 74103723 | ADD | 1667 | 1.028 | 0.2248 | 0.8222 |
| 6 | rs12194422 | 74731052 | ADD | 1662 | 1.074 | 0.8845 | 0.3764 |
| 6 | rs9352668 | 79629397 | ADD | 1640 | 0.9297 | -1.019 | 0.308 |
| 6 | rs10484941 | 80219091 | ADD | 1664 | 0.832 | -1.826 | 0.0679 |
| 6 | rs9361886 | 82835221 | ADD | 1665 | 1.062 | 0.8784 | 0.3797 |
| 6 | rs12212188 | 83767006 | ADD | 1658 | 0.8682 | -1.538 | 0.1241 |
| 6 | rs6903593 | 85121967 | ADD | 1662 | 0.9398 | -0.9054 | 0.3653 |
| 6 | rs1538408 | 87395345 | ADD | 1666 | 1.08 | 0.7793 | 0.4358 |
| 6 | rs9444725 | 90770775 | ADD | 1640 | 0.88 | -1.581 | 0.114 |
| 6 | rs3734660 | 90973066 | ADD | 1611 | 0.913 | -1.314 | 0.1888 |
| 6 | rs2730846 | 93207521 | ADD | 1638 | 0.8931 | -1.128 | 0.2592 |
| 6 | rs16871170 | 94097830 | ADD | 1667 | 0.8767 | -0.9292 | 0.3528 |
| 6 | rs4707796 | 94133139 | ADD | 1640 | 0.9695 | -0.2549 | 0.7988 |
| 6 | rs6905617 | 95699969 | ADD | 1653 | 1.012 | 0.1186 | 0.9056 |
| 6 | rs1334328 | 97201165 | ADD | 1627 | 0.8914 | -1.665 | 0.096 |
| 6 | rs6940468 | 105192818 | ADD | 1657 | 1.053 | 0.5016 | 0.616 |
| 6 | rs620611 | 113815512 | ADD | 1667 | 1.101 | 1.036 | 0.3002 |
| 6 | rs9400661 | 114147623 | ADD | 1667 | 1.093 | 1.228 | 0.2194 |
| 6 | rs1031800 | 114256717 | ADD | 1633 | 1.028 | 0.339 | 0.7346 |
| 6 | rs4946259 | 117922786 | ADD | 1650 | 1.077 | 1.089 | 0.2763 |
| 6 | rs17082894 | 121262988 | ADD | 1659 | 1.092 | 0.8799 | 0.3789 |
| 6 | rs492800 | 125444410 | ADD | 1667 | 1.065 | 0.9084 | 0.3637 |
| 6 | rs4895532 | 138907017 | ADD | 1665 | 1.13 | 1.654 | 0.09821 |
| 6 | rs6902780 | 142440419 | ADD | 1614 | 1.048 | 0.5702 | 0.5685 |
| 6 | rs12528289 | 144299137 | ADD | 1665 | 0.9607 | -0.5488 | 0.5832 |
| 6 | rs7753282 | 145406043 | ADD | 1665 | 0.9785 | -0.1564 | 0.8757 |
| 6 | rs644866 | 150405702 | ADD | 1624 | 1.014 | 0.1833 | 0.8546 |
| 6 | rs659156 | 150454526 | ADD | 1657 | 0.9368 | -0.8774 | 0.3803 |
| 6 | rs9397629 | 150464350 | ADD | 1646 | 1.022 | 0.1998 | 0.8416 |
| 6 | rs2057557 | 150536566 | ADD | 1640 | 1.076 | 0.8531 | 0.3936 |
| 6 | rs11155719 | 150679170 | ADD | 1655 | 1.068 | 0.9593 | 0.3374 |
| 6 | rs2248586 | 152137025 | ADD | 1667 | 1.013 | 0.1618 | 0.8715 |
| 6 | rs2756117 | 153108601 | ADD | 1665 | 0.8764 | -1.126 | 0.2602 |
| 6 | rs6455652 | 159847589 | ADD | 1666 | 0.9849 | -0.2162 | 0.8288 |
| 6 | rs7757336 | 160609548 | ADD | 1645 | 0.9346 | -0.7606 | 0.4469 |
| 6 | rs1488 | 161458240 | ADD | 1659 | 1.125 | 1.631 | 0.103 |
| 6 | rs2293289 | 161471429 | ADD | 1665 | 1.079 | 0.6894 | 0.4906 |
| 6 | rs2294458 | 163586326 | ADD | 1660 | 0.8361 | -1.571 | 0.1161 |
| 6 | rs697471 | 166299886 | ADD | 1667 | 1.064 | 0.9073 | 0.3643 |
| 6 | rs697482 | 166341627 | ADD | 1622 | 1.219 | 2.292 | 0.02189 |
| 6 | rs9348093 | 166513881 | ADD | 1662 | 0.9746 | -0.2008 | 0.8409 |
| 6 | rs4518470 | 167005045 | ADD | 1602 | 1.039 | 0.3718 | 0.71 |
| 6 | rs1387921 | 168212567 | ADD | 1650 | 1.012 | 0.1004 | 0.92 |
| 6 | rs1232314 | 168632154 | ADD | 1666 | 0.8559 | -1.677 | 0.09345 |
| 6 | rs4708582 | 169213632 | ADD | 1660 | 1.009 | 0.1323 | 0.8947 |
| 6 | rs6605524 | 169398045 | ADD | 1667 | 1.042 | 0.5991 | 0.5491 |
| 7 | rs4723604 | 4298964 | ADD | 1666 | 1.011 | 0.1492 | 0.8814 |
| 7 | rs308092 | 5850048 | ADD | 1656 | 1.018 | 0.2681 | 0.7886 |
| 7 | rs1526520 | 11746243 | ADD | 1664 | 0.9917 | -0.1238 | 0.9014 |
| 7 | rs4463318 | 11824029 | ADD | 1641 | 1.04 | 0.5142 | 0.6071 |
| 7 | rs11760715 | 13184576 | ADD | 1663 | 0.887 | -1.761 | 0.07831 |
| 7 | rs6967846 | 14202326 | ADD | 1662 | 1.074 | 0.8479 | 0.3965 |
| 7 | rs6461292 | 16970912 | ADD | 1662 | 0.8854 | -1.468 | 0.1421 |
| 7 | rs847440 | 16984957 | ADD | 1657 | 0.8029 | -3.038 | 0.002382 |
| 7 | rs756853 | 18856525 | ADD | 1666 | 1.033 | 0.4656 | 0.6415 |
| 7 | rs17140345 | 18944036 | ADD | 1664 | 0.9905 | -0.1077 | 0.9142 |
| 7 | rs7784579 | 20493949 | ADD | 1656 | 0.945 | -0.8184 | 0.4131 |
| 7 | rs6461569 | 21502301 | ADD | 1620 | 0.9964 | -0.04996 | 0.9602 |
| 7 | rs2222521 | 21646838 | ADD | 1667 | 1.034 | 0.2669 | 0.7895 |
| 7 | rs16873129 | 22111609 | ADD | 1661 | 1.323 | 2.103 | 0.03547 |
| 7 | rs6959894 | 22164250 | ADD | 1667 | 1.021 | 0.2001 | 0.8414 |
| 7 | rs1122913 | 22569457 | ADD | 1661 | 0.9625 | -0.5326 | 0.5943 |
| 7 | rs3814095 | 26113744 | ADD | 1666 | 1.007 | 0.07944 | 0.9367 |
| 7 | rs2252521 | 29007715 | ADD | 1653 | 0.9618 | -0.5237 | 0.6005 |
| 7 | rs2024421 | 29490878 | ADD | 1665 | 0.9223 | -1.137 | 0.2556 |
| 7 | rs17158705 | 30210812 | ADD | 1646 | 1.035 | 0.4287 | 0.6682 |
| 7 | rs11771217 | 30749288 | ADD | 1664 | 1.127 | 1.746 | 0.0808 |
| 7 | rs7784067 | 31050624 | ADD | 1661 | 0.9773 | -0.3314 | 0.7404 |
| 7 | rs6961368 | 31976452 | ADD | 1657 | 1.095 | 1.32 | 0.1869 |
| 7 | rs2392147 | 32833248 | ADD | 1659 | 1.156 | 2.16 | 0.03074 |
| 7 | rs11768361 | 34196895 | ADD | 1664 | 0.9279 | -1.089 | 0.2763 |
| 7 | rs318572 | 34243290 | ADD | 1665 | 0.9977 | -0.03362 | 0.9732 |
| 7 | rs340383 | 35371115 | ADD | 1657 | 1.013 | 0.1879 | 0.851 |
| 7 | rs343041 | 35473258 | ADD | 1631 | 0.9975 | -0.03363 | 0.9732 |
| 7 | rs756507 | 36844270 | ADD | 1651 | 0.999 | -0.009988 | 0.992 |
| 7 | rs2299945 | 38408168 | ADD | 1662 | 1.053 | 0.6932 | 0.4882 |
| 7 | rs273123 | 41452144 | ADD | 1667 | 1.095 | 1.227 | 0.2199 |
| 7 | rs740094 | 44636223 | ADD | 1667 | 1.008 | 0.1178 | 0.9062 |
| 7 | rs7787946 | 47756443 | ADD | 1667 | 1.047 | 0.6524 | 0.5142 |
| 7 | rs6945518 | 52562247 | ADD | 1663 | 1.128 | 1.431 | 0.1525 |
| 7 | rs2960632 | 52626737 | ADD | 1667 | 1.033 | 0.4552 | 0.649 |
| 7 | rs1442256 | 52646539 | ADD | 1620 | 0.9812 | -0.2108 | 0.833 |
| 7 | rs12536631 | 53883344 | ADD | 1653 | 0.9785 | -0.2669 | 0.7896 |
| 7 | rs4425715 | 54233081 | ADD | 1642 | 1.06 | 0.7936 | 0.4274 |
| 7 | rs6956675 | 62215205 | ADD | 1641 | 1.149 | 1.716 | 0.08612 |
| 7 | rs7459368 | 69726632 | ADD | 1663 | 0.7904 | -3.377 | 0.000733 |
| 7 | rs12538253 | 75225967 | ADD | 1606 | 0.9255 | -0.9621 | 0.336 |
| 7 | rs13243127 | 77910933 | ADD | 1665 | 1.064 | 0.9301 | 0.3523 |
| 7 | rs10485891 | 77918756 | ADD | 1652 | 1.071 | 0.8507 | 0.395 |
| 7 | rs7782195 | 77957867 | ADD | 1666 | 0.8218 | -2.723 | 0.006462 |
| 7 | rs17156605 | 82193015 | ADD | 1663 | 0.8171 | -0.8474 | 0.3968 |
| 7 | rs12704637 | 91712622 | ADD | 1666 | 0.9819 | -0.2454 | 0.8061 |
| 7 | rs7806426 | 92483969 | ADD | 1661 | 0.9534 | -0.5589 | 0.5762 |
| 7 | rs2299267 | 94899857 | ADD | 1667 | 1.262 | 2.486 | 0.01293 |
| 7 | rs3757707 | 94904911 | ADD | 1667 | 0.876 | -1.785 | 0.07431 |
| 7 | rs12704811 | 95086305 | ADD | 1664 | 0.9874 | -0.1503 | 0.8805 |
| 7 | rs2245617 | 103016271 | ADD | 1666 | 1.02 | 0.225 | 0.822 |
| 7 | rs28253 | 103942086 | ADD | 1636 | 0.9428 | -0.826 | 0.4088 |
| 7 | rs42172 | 106274210 | ADD | 1649 | 0.9462 | -0.8029 | 0.422 |
| 7 | rs17147421 | 124171184 | ADD | 1656 | 1.029 | 0.372 | 0.7099 |
| 7 | rs10278805 | 126709864 | ADD | 1665 | 1.059 | 0.7412 | 0.4586 |
| 7 | rs290796 | 130066212 | ADD | 1649 | 0.8725 | -1.292 | 0.1965 |
| 7 | rs2971769 | 131058480 | ADD | 1667 | 1.077 | 1.064 | 0.2875 |
| 7 | rs2350786 | 136327110 | ADD | 1665 | 1.045 | 0.5823 | 0.5604 |
| 7 | rs322295 | 136571602 | ADD | 1662 | 1.157 | 2.094 | 0.03622 |
| 7 | rs2174362 | 138450882 | ADD | 1664 | 0.861 | -1.006 | 0.3144 |
| 7 | rs8192835 | 139303078 | ADD | 1654 | 1.05 | 0.5944 | 0.5522 |
| 7 | rs6962291 | 139317586 | ADD | 1665 | 0.9153 | -1.189 | 0.2343 |
| 7 | rs7785094 | 139828871 | ADD | 1667 | 1.114 | 1.279 | 0.2008 |
| 7 | rs7777187 | 154921923 | ADD | 1666 | 1.072 | 0.6784 | 0.4975 |
| 7 | rs1873884 | 155721317 | ADD | 1655 | 0.8805 | -1.583 | 0.1134 |
| 7 | rs1880342 | 155900937 | ADD | 1667 | 0.9584 | -0.6072 | 0.5437 |
| 7 | rs4716610 | 155936323 | ADD | 1662 | 0.916 | -1.198 | 0.231 |
| 7 | rs2177558 | 156001449 | ADD | 1667 | 0.9292 | -0.9295 | 0.3526 |
| 7 | rs6966038 | 156573159 | ADD | 1658 | 0.7525 | -3.451 | 0.0005578 |
| 7 | rs2366645 | 157150776 | ADD | 1667 | 1.083 | 1.15 | 0.2501 |
| 7 | rs16868174 | 158712865 | ADD | 1667 | 0.9466 | -0.4466 | 0.6551 |
| 8 | rs10088894 | 400640 | ADD | 1659 | 1.041 | 0.4745 | 0.6351 |
| 8 | rs17667487 | 854408 | ADD | 1667 | 1.115 | 1.085 | 0.2778 |
| 8 | rs10093402 | 1280468 | ADD | 1665 | 1.013 | 0.1784 | 0.8584 |
| 8 | rs4292733 | 1827380 | ADD | 1667 | 0.9624 | -0.5575 | 0.5772 |
| 8 | rs2406305 | 3005790 | ADD | 1667 | 1.091 | 0.956 | 0.3391 |
| 8 | rs2469358 | 3438519 | ADD | 1667 | 1.002 | 0.02379 | 0.981 |
| 8 | rs4875303 | 4028983 | ADD | 1666 | 0.934 | -0.8615 | 0.389 |
| 8 | rs9693235 | 4109411 | ADD | 1665 | 1.249 | 1.695 | 0.09011 |
| 8 | rs7817362 | 4912668 | ADD | 1663 | 1.066 | 0.9495 | 0.3424 |
| 8 | rs2921023 | 8387780 | ADD | 1656 | 1.102 | 1.249 | 0.2117 |
| 8 | rs2980754 | 8398664 | ADD | 1660 | 0.9526 | -0.6686 | 0.5037 |
| 8 | rs10099072 | 8474533 | ADD | 1659 | 0.8701 | -1.798 | 0.0722 |
| 8 | rs10107215 | 8877993 | ADD | 1653 | 1.018 | 0.2359 | 0.8135 |
| 8 | rs10100760 | 9407969 | ADD | 1667 | 0.8542 | -2.216 | 0.02669 |
| 8 | rs11774592 | 9858434 | ADD | 1656 | 0.9347 | -0.5108 | 0.6095 |
| 8 | rs10503418 | 11156393 | ADD | 1655 | 1.01 | 0.143 | 0.8863 |
| 8 | rs3808518 | 11180682 | ADD | 1666 | 1.016 | 0.2287 | 0.8191 |
| 8 | rs4469501 | 11862365 | ADD | 1666 | 0.9274 | -0.9766 | 0.3288 |
| 8 | rs1872342 | 15988678 | ADD | 1660 | 0.9276 | -0.9881 | 0.3231 |
| 8 | rs2959634 | 17071223 | ADD | 1660 | 1.01 | 0.1521 | 0.8791 |
| 8 | rs7388381 | 18026157 | ADD | 1650 | 0.979 | -0.2761 | 0.7825 |
| 8 | rs6586734 | 18238667 | ADD | 1630 | 1.287 | 3.179 | 0.001476 |
| 8 | rs17127180 | 18676556 | ADD | 1663 | 1.101 | 0.3924 | 0.6947 |
| 8 | rs691605 | 19072929 | ADD | 1657 | 1.066 | 0.8452 | 0.398 |
| 8 | rs10503656 | 19195160 | ADD | 1597 | 0.9964 | -0.04599 | 0.9633 |
| 8 | rs17089124 | 23249254 | ADD | 1667 | 1.028 | 0.154 | 0.8776 |
| 8 | rs1918211 | 25049587 | ADD | 1641 | 0.9064 | -0.6428 | 0.5203 |
| 8 | rs10102339 | 27023405 | ADD | 1661 | 1.015 | 0.207 | 0.836 |
| 8 | rs4732849 | 28249765 | ADD | 1664 | 0.9621 | -0.5151 | 0.6065 |
| 8 | rs2954794 | 29163307 | ADD | 1667 | 0.8543 | -0.9996 | 0.3175 |
| 8 | rs4073220 | 29558828 | ADD | 1667 | 0.9355 | -0.7709 | 0.4408 |
| 8 | rs12543523 | 36780844 | ADD | 1657 | 1.128 | 1.362 | 0.1733 |
| 8 | rs7812568 | 39870563 | ADD | 1648 | 1.004 | 0.0461 | 0.9632 |
| 8 | rs7822883 | 49720677 | ADD | 1661 | 1.09 | 0.7728 | 0.4396 |
| 8 | rs906656 | 51805005 | ADD | 1665 | 0.9043 | -1.297 | 0.1945 |
| 8 | rs16919588 | 54914244 | ADD | 1649 | 1.378 | 1.682 | 0.09258 |
| 8 | rs2975997 | 56513192 | ADD | 1667 | 0.9874 | -0.1695 | 0.8654 |
| 8 | rs2726599 | 59881250 | ADD | 1666 | 1.105 | 1.396 | 0.1627 |
| 8 | rs2013112 | 61559383 | ADD | 1667 | 1.036 | 0.4985 | 0.6181 |
| 8 | rs11986500 | 62113757 | ADD | 1615 | 1.004 | 0.03689 | 0.9706 |
| 8 | rs10097272 | 63434767 | ADD | 1664 | 1.143 | 1.937 | 0.05277 |
| 8 | rs4737771 | 67354933 | ADD | 1667 | 1.165 | 1.437 | 0.1506 |
| 8 | rs16939127 | 76736389 | ADD | 1666 | 1.119 | 1.465 | 0.1428 |
| 8 | rs4739755 | 81665747 | ADD | 1656 | 1.008 | 0.08293 | 0.9339 |
| 8 | rs7830040 | 83079508 | ADD | 1666 | 0.9107 | -1.342 | 0.1795 |
| 8 | rs10097366 | 89125685 | ADD | 1664 | 0.9757 | -0.3063 | 0.7594 |
| 8 | rs4336580 | 94606241 | ADD | 1667 | 0.9376 | -0.8867 | 0.3752 |
| 8 | rs10808664 | 95060910 | ADD | 1637 | 0.9406 | -0.8695 | 0.3846 |
| 8 | rs7817230 | 95480162 | ADD | 1664 | 1.2 | 1.363 | 0.173 |
| 8 | rs2016731 | 96813579 | ADD | 1628 | 1.001 | 0.006678 | 0.9947 |
| 8 | rs7017245 | 97169636 | ADD | 1662 | 0.9969 | -0.04456 | 0.9645 |
| 8 | rs2444895 | 99090125 | ADD | 1666 | 0.9715 | -0.3956 | 0.6924 |
| 8 | rs1460930 | 101183970 | ADD | 1666 | 0.8663 | -2.003 | 0.04518 |
| 8 | rs4734487 | 101931489 | ADD | 1664 | 1.059 | 0.8399 | 0.401 |
| 8 | rs895602 | 102202011 | ADD | 1613 | 1.068 | 0.9574 | 0.3384 |
| 8 | rs16867827 | 102644282 | ADD | 1666 | 1.103 | 0.6605 | 0.5089 |
| 8 | rs2511733 | 103629532 | ADD | 1665 | 0.9554 | -0.6507 | 0.5152 |
| 8 | rs2515173 | 104190450 | ADD | 1667 | 1.034 | 0.4899 | 0.6242 |
| 8 | rs2454000 | 104205020 | ADD | 1667 | 1.056 | 0.6649 | 0.5061 |
| 8 | rs3107548 | 106174444 | ADD | 1667 | 1.136 | 1.264 | 0.2063 |
| 8 | rs10481055 | 110720499 | ADD | 1666 | 1.018 | 0.2014 | 0.8404 |
| 8 | rs4559271 | 116226459 | ADD | 1665 | 0.9941 | -0.08407 | 0.933 |
| 8 | rs12682047 | 117986808 | ADD | 1642 | 0.9551 | -0.3988 | 0.69 |
| 8 | rs13438834 | 121887476 | ADD | 1667 | 1.035 | 0.4341 | 0.6642 |
| 8 | rs4870806 | 123716146 | ADD | 1664 | 1.076 | 1.059 | 0.2894 |
| 8 | rs13275576 | 129358294 | ADD | 1666 | 0.924 | -0.7717 | 0.4403 |
| 8 | rs7844704 | 129914817 | ADD | 1667 | 0.9287 | -0.9591 | 0.3375 |
| 8 | rs16904196 | 131123472 | ADD | 1650 | 1.322 | 1.437 | 0.1506 |
| 8 | rs11775398 | 131508949 | ADD | 1665 | 1.039 | 0.3706 | 0.7109 |
| 8 | rs273403 | 132580190 | ADD | 1592 | 1.049 | 0.2932 | 0.7694 |
| 8 | rs16904657 | 133400220 | ADD | 1659 | 0.9347 | -0.4654 | 0.6417 |
| 8 | rs4410884 | 134256900 | ADD | 1662 | 0.9985 | -0.02228 | 0.9822 |
| 8 | rs16904971 | 134727142 | ADD | 1664 | 1.078 | 0.7562 | 0.4495 |
| 8 | rs16905528 | 137310055 | ADD | 1666 | 0.8269 | -1.49 | 0.1363 |
| 8 | rs10112884 | 138847029 | ADD | 1659 | 1.059 | 0.8372 | 0.4025 |
| 8 | rs3898456 | 139244531 | ADD | 1619 | 1.038 | 0.4736 | 0.6358 |
| 8 | rs2471083 | 140588523 | ADD | 1659 | 0.9688 | -0.4147 | 0.6783 |
| 8 | rs7819785 | 140962053 | ADD | 1658 | 1.013 | 0.1439 | 0.8856 |
| 8 | rs6578061 | 141114914 | ADD | 1660 | 1.041 | 0.5941 | 0.5524 |
| 8 | rs4961309 | 141583366 | ADD | 1666 | 0.9938 | -0.07142 | 0.9431 |
| 8 | rs3922678 | 142266863 | ADD | 1633 | 1.008 | 0.1177 | 0.9063 |
| 9 | rs512774 | 1699308 | ADD | 1664 | 1.277 | 2.138 | 0.0325 |
| 9 | rs2150720 | 2459742 | ADD | 1667 | 1.035 | 0.4759 | 0.6341 |
| 9 | rs6476798 | 4038636 | ADD | 1664 | 0.8643 | -1.756 | 0.07911 |
| 9 | rs7864244 | 4899719 | ADD | 1666 | 0.8948 | -1.525 | 0.1273 |
| 9 | rs6477134 | 6964101 | ADD | 1665 | 1.109 | 1.509 | 0.1312 |
| 9 | rs2031195 | 14414929 | ADD | 1667 | 1.04 | 0.3793 | 0.7045 |
| 9 | rs10115913 | 16649259 | ADD | 1656 | 1.021 | 0.3076 | 0.7584 |
| 9 | rs10962549 | 16709445 | ADD | 1635 | 0.9583 | -0.4575 | 0.6473 |
| 9 | rs10811063 | 18911386 | ADD | 1666 | 0.8853 | -1.515 | 0.1299 |
| 9 | rs10964234 | 19590362 | ADD | 1645 | 1.01 | 0.09934 | 0.9209 |
| 9 | rs10966380 | 24660982 | ADD | 1662 | 1.067 | 0.8744 | 0.3819 |
| 9 | rs7861757 | 24840287 | ADD | 1665 | 1.005 | 0.06991 | 0.9443 |
| 9 | rs4316218 | 24896802 | ADD | 1661 | 0.9643 | -0.4629 | 0.6434 |
| 9 | rs7038176 | 24966322 | ADD | 1667 | 0.9774 | -0.31 | 0.7565 |
| 9 | rs10738742 | 26010806 | ADD | 1645 | 1.028 | 0.3582 | 0.7202 |
| 9 | rs1932713 | 26238035 | ADD | 1606 | 1.145 | 1.561 | 0.1185 |
| 9 | rs1857079 | 27723472 | ADD | 1667 | 1.119 | 1.036 | 0.3002 |
| 9 | rs7857732 | 28226666 | ADD | 1666 | 0.9323 | -0.7594 | 0.4476 |
| 9 | rs7871600 | 28930111 | ADD | 1638 | 1.218 | 2.236 | 0.02534 |
| 9 | rs10968884 | 29010053 | ADD | 1665 | 1.156 | 1.427 | 0.1535 |
| 9 | rs1412341 | 31609571 | ADD | 1667 | 1.045 | 0.5902 | 0.5551 |
| 9 | rs16917898 | 32106520 | ADD | 1653 | 0.9454 | -0.4898 | 0.6243 |
| 9 | rs7027564 | 32309302 | ADD | 1667 | 0.9796 | -0.1975 | 0.8435 |
| 9 | rs10814295 | 35895761 | ADD | 1664 | 0.9942 | -0.07287 | 0.9419 |
| 9 | rs10972784 | 36176264 | ADD | 1667 | 0.974 | -0.3153 | 0.7526 |
| 9 | rs4745035 | 72564825 | ADD | 1659 | 1.02 | 0.2605 | 0.7944 |
| 9 | rs10511999 | 73486317 | ADD | 1666 | 0.9518 | -0.7002 | 0.4838 |
| 9 | rs10118224 | 76735058 | ADD | 1666 | 1.109 | 1.169 | 0.2424 |
| 9 | rs11144179 | 76797965 | ADD | 1664 | 0.9554 | -0.2578 | 0.7966 |
| 9 | rs10869801 | 78525441 | ADD | 1661 | 1.018 | 0.2009 | 0.8408 |
| 9 | rs7861307 | 78585692 | ADD | 1667 | 1.001 | 0.009541 | 0.9924 |
| 9 | rs10780510 | 83149231 | ADD | 1609 | 0.9624 | -0.5018 | 0.6158 |
| 9 | rs10116287 | 86667094 | ADD | 1656 | 1.093 | 0.8214 | 0.4114 |
| 9 | rs10491846 | 88352001 | ADD | 1667 | 1.164 | 2.205 | 0.02742 |
| 9 | rs10746839 | 90073450 | ADD | 1662 | 1.002 | 0.0276 | 0.978 |
| 9 | rs10991978 | 93353660 | ADD | 1602 | 0.9574 | -0.2418 | 0.8089 |
| 9 | rs10512215 | 93414535 | ADD | 1667 | 0.9844 | -0.1934 | 0.8467 |
| 9 | rs6479457 | 94950329 | ADD | 1667 | 1.187 | 2.364 | 0.01809 |
| 9 | rs7860361 | 97187625 | ADD | 1610 | 0.862 | -1.8 | 0.07182 |
| 9 | rs2296812 | 98658902 | ADD | 1657 | 0.9969 | -0.0269 | 0.9785 |
| 9 | rs2778913 | 100419601 | ADD | 1665 | 1.039 | 0.4028 | 0.6871 |
| 9 | rs1407877 | 103395276 | ADD | 1664 | 0.7956 | -1.935 | 0.05304 |
| 9 | rs7860970 | 105481693 | ADD | 1666 | 0.942 | -0.7291 | 0.4659 |
| 9 | rs10978761 | 109026648 | ADD | 1664 | 1.168 | 1.519 | 0.1289 |
| 9 | rs2808374 | 109026900 | ADD | 1659 | 1.117 | 1.391 | 0.1642 |
| 9 | rs10816481 | 109082305 | ADD | 1643 | 1.071 | 0.909 | 0.3634 |
| 9 | rs10979023 | 109517708 | ADD | 1623 | 1.069 | 0.6657 | 0.5056 |
| 9 | rs10816625 | 109876894 | ADD | 1667 | 0.9605 | -0.3053 | 0.7602 |
| 9 | rs4135168 | 112056706 | ADD | 1666 | 1.072 | 0.8711 | 0.3837 |
| 9 | rs7853673 | 115751062 | ADD | 1657 | 0.9111 | -1.335 | 0.1819 |
| 9 | rs2636870 | 116141867 | ADD | 1667 | 0.9611 | -0.5803 | 0.5617 |
| 9 | rs1372330 | 118559409 | ADD | 1667 | 1.051 | 0.4835 | 0.6287 |
| 9 | rs545966 | 119317275 | ADD | 1665 | 1.011 | 0.1562 | 0.8758 |
| 9 | rs16911180 | 123778532 | ADD | 1666 | 1.162 | 1.858 | 0.06318 |
| 9 | rs13283472 | 124145611 | ADD | 1665 | 0.9647 | -0.3164 | 0.7517 |
| 9 | rs10760430 | 128205909 | ADD | 1667 | 1.041 | 0.4786 | 0.6322 |
| 9 | rs10987595 | 129129144 | ADD | 1651 | 1.1 | 0.4199 | 0.6746 |
| 9 | rs10819490 | 131096139 | ADD | 1660 | 0.9662 | -0.5033 | 0.6147 |
| 9 | rs522464 | 133204204 | ADD | 1662 | 1.071 | 0.8949 | 0.3708 |
| 9 | rs1999121 | 134592548 | ADD | 1667 | 1.175 | 1.969 | 0.04893 |
| 9 | rs3780804 | 135587949 | ADD | 1617 | 0.9985 | -0.01652 | 0.9868 |
| 9 | rs1075654 | 135918097 | ADD | 1660 | 1.142 | 1.15 | 0.2501 |
| 9 | rs3922982 | 136726816 | ADD | 1665 | 0.7969 | -2.328 | 0.01993 |
| 9 | rs2031825 | 137166820 | ADD | 1667 | 0.9305 | -0.9116 | 0.362 |
| 9 | rs1318383 | 137807894 | ADD | 1666 | 0.8787 | -1.645 | 0.09988 |
| 9 | rs4842091 | 138086988 | ADD | 1662 | 0.9505 | -0.649 | 0.5164 |
| 9 | rs11103350 | 138175430 | ADD | 1662 | 1.106 | 1.431 | 0.1523 |
| 9 | rs11793385 | 138727787 | ADD | 1628 | 1.028 | 0.3283 | 0.7427 |
| 10 | rs3793735 | 1272828 | ADD | 1667 | 0.8649 | -1.906 | 0.05667 |
| 10 | rs945254 | 2085216 | ADD | 1647 | 1.033 | 0.4725 | 0.6366 |
| 10 | rs10903713 | 2282180 | ADD | 1662 | 0.9325 | -0.8887 | 0.3742 |
| 10 | rs7080649 | 4721081 | ADD | 1662 | 0.9183 | -1.212 | 0.2254 |
| 10 | rs11257726 | 6351755 | ADD | 1667 | 0.897 | -1.147 | 0.2515 |
| 10 | rs4146431 | 7056070 | ADD | 1589 | 0.9609 | -0.5586 | 0.5764 |
| 10 | rs1514232 | 8182713 | ADD | 1666 | 1.099 | 1.259 | 0.2081 |
| 10 | rs6602348 | 9051752 | ADD | 1651 | 1.043 | 0.4922 | 0.6226 |
| 10 | rs1934358 | 10004714 | ADD | 1667 | 1.005 | 0.06141 | 0.951 |
| 10 | rs17151584 | 12443034 | ADD | 1667 | 0.9825 | -0.1405 | 0.8882 |
| 10 | rs663177 | 13131552 | ADD | 1667 | 0.8514 | -2.179 | 0.02932 |
| 10 | rs880853 | 14498588 | ADD | 1667 | 1.075 | 0.9815 | 0.3263 |
| 10 | rs4489642 | 14730749 | ADD | 1663 | 1.025 | 0.2791 | 0.7802 |
| 10 | rs7903263 | 14738958 | ADD | 1666 | 0.9836 | -0.2273 | 0.8202 |
| 10 | rs10737093 | 15225509 | ADD | 1615 | 1.026 | 0.2502 | 0.8024 |
| 10 | rs7915486 | 16559769 | ADD | 1665 | 1.091 | 1.291 | 0.1968 |
| 10 | rs10508512 | 16572580 | ADD | 1652 | 1.052 | 0.4502 | 0.6526 |
| 10 | rs11254171 | 16840124 | ADD | 1603 | 0.8266 | -1.15 | 0.2501 |
| 10 | rs7090118 | 18499288 | ADD | 1638 | 1.107 | 1.353 | 0.176 |
| 10 | rs1779229 | 18569425 | ADD | 1667 | 1.093 | 1.189 | 0.2343 |
| 10 | rs10827926 | 20345790 | ADD | 1667 | 0.9992 | -0.01135 | 0.9909 |
| 10 | rs7098524 | 20663570 | ADD | 1667 | 0.985 | -0.2161 | 0.8289 |
| 10 | rs33932343 | 21284093 | ADD | 1660 | 1.003 | 0.04432 | 0.9646 |
| 10 | rs11013556 | 23861364 | ADD | 1667 | 1.232 | 0.6742 | 0.5002 |
| 10 | rs4747610 | 28338694 | ADD | 1664 | 1.028 | 0.3913 | 0.6956 |
| 10 | rs12767457 | 29260297 | ADD | 1613 | 1.052 | 0.4902 | 0.624 |
| 10 | rs2788453 | 29501170 | ADD | 1600 | 0.9772 | -0.146 | 0.8839 |
| 10 | rs16929898 | 29530715 | ADD | 1666 | 1.347 | 1.719 | 0.08569 |
| 10 | rs2488264 | 29609361 | ADD | 1666 | 1.032 | 0.3641 | 0.7157 |
| 10 | rs2776632 | 30252761 | ADD | 1667 | 1.171 | 2.255 | 0.02412 |
| 10 | rs2785264 | 33729749 | ADD | 1630 | 0.996 | -0.05696 | 0.9546 |
| 10 | rs2492448 | 35235412 | ADD | 1654 | 1.004 | 0.0507 | 0.9596 |
| 10 | rs2384598 | 36706518 | ADD | 1661 | 1.063 | 0.6188 | 0.536 |
| 10 | rs702371 | 42558251 | ADD | 1658 | 0.9964 | -0.04945 | 0.9606 |
| 10 | rs726786 | 49668575 | ADD | 1666 | 1.022 | 0.3202 | 0.7488 |
| 10 | rs10994675 | 51233999 | ADD | 1650 | 1.155 | 2.069 | 0.03856 |
| 10 | rs10822867 | 52815711 | ADD | 1660 | 0.9695 | -0.2875 | 0.7738 |
| 10 | rs10997890 | 52946536 | ADD | 1666 | 1.035 | 0.4419 | 0.6586 |
| 10 | rs11004610 | 56298295 | ADD | 1657 | 1.097 | 1.326 | 0.1848 |
| 10 | rs7085788 | 62288404 | ADD | 1667 | 1.029 | 0.4018 | 0.6878 |
| 10 | rs7087125 | 63443045 | ADD | 1666 | 0.9929 | -0.1061 | 0.9155 |
| 10 | rs1553789 | 64549235 | ADD | 1660 | 1.07 | 0.9557 | 0.3392 |
| 10 | rs10509195 | 65193372 | ADD | 1667 | 0.9818 | -0.2608 | 0.7942 |
| 10 | rs12779355 | 67998550 | ADD | 1666 | 1.071 | 0.9855 | 0.3244 |
| 10 | rs10997242 | 68006301 | ADD | 1662 | 1.301 | 2.788 | 0.005303 |
| 10 | rs716547 | 68424659 | ADD | 1666 | 0.9807 | -0.2543 | 0.7992 |
| 10 | rs2497099 | 70247228 | ADD | 1666 | 1.122 | 1.529 | 0.1264 |
| 10 | rs3843584 | 71140731 | ADD | 1667 | 0.9812 | -0.2654 | 0.7907 |
| 10 | rs2763336 | 71237767 | ADD | 1661 | 0.869 | -1.932 | 0.05333 |
| 10 | rs2271700 | 71519942 | ADD | 1667 | 1.065 | 0.7044 | 0.4812 |
| 10 | rs10823772 | 72934436 | ADD | 1648 | 0.8348 | -2.203 | 0.02762 |
| 10 | rs7921151 | 72978865 | ADD | 1652 | 1 | 0.002806 | 0.9978 |
| 10 | rs4746136 | 74971000 | ADD | 1664 | 1.036 | 0.3878 | 0.6981 |
| 10 | rs7069982 | 78337197 | ADD | 1666 | 1.004 | 0.04488 | 0.9642 |
| 10 | rs1250576 | 80634802 | ADD | 1666 | 1.002 | 0.02781 | 0.9778 |
| 10 | rs11192252 | 83788239 | ADD | 1664 | 0.9999 | -0.0006145 | 0.9995 |
| 10 | rs17099339 | 83822284 | ADD | 1657 | 1.03 | 0.1188 | 0.9054 |
| 10 | rs10885239 | 84460638 | ADD | 1646 | 0.9013 | -1.27 | 0.2042 |
| 10 | rs10749482 | 85962023 | ADD | 1652 | 0.9777 | -0.3159 | 0.752 |
| 10 | rs10887363 | 86515945 | ADD | 1666 | 1.156 | 1.418 | 0.1563 |
| 10 | rs17105725 | 87359244 | ADD | 1667 | 1.006 | 0.05261 | 0.958 |
| 10 | rs7096107 | 88837965 | ADD | 1662 | 0.9773 | -0.3204 | 0.7487 |
| 10 | rs575510 | 89951637 | ADD | 1667 | 1.031 | 0.335 | 0.7377 |
| 10 | rs10509557 | 90653819 | ADD | 1666 | 1.104 | 1.232 | 0.2178 |
| 10 | rs1441734 | 90706817 | ADD | 1666 | 1.04 | 0.5677 | 0.5702 |
| 10 | rs2800156 | 92431317 | ADD | 1667 | 1.003 | 0.04319 | 0.9656 |
| 10 | rs17112076 | 98459683 | ADD | 1650 | 0.9028 | -1.046 | 0.2955 |
| 10 | rs7081796 | 99194516 | ADD | 1666 | 1.001 | 0.01034 | 0.9918 |
| 10 | rs2297643 | 99349977 | ADD | 1628 | 1.005 | 0.05122 | 0.9592 |
| 10 | rs11189937 | 100770705 | ADD | 1608 | 1.441 | 2.891 | 0.003841 |
| 10 | rs7068079 | 101357305 | ADD | 1643 | 1.021 | 0.2932 | 0.7694 |
| 10 | rs902997 | 105374252 | ADD | 1667 | 0.978 | -0.2986 | 0.7652 |
| 10 | rs821683 | 108132851 | ADD | 1658 | 1.111 | 1.48 | 0.139 |
| 10 | rs1660985 | 109259862 | ADD | 1639 | 0.9724 | -0.1799 | 0.8572 |
| 10 | rs2419840 | 115322088 | ADD | 1665 | 0.9342 | -0.9493 | 0.3425 |
| 10 | rs1006286 | 119509000 | ADD | 1666 | 0.8168 | -1.549 | 0.1213 |
| 10 | rs17098212 | 120687545 | ADD | 1667 | 1.065 | 0.6662 | 0.5053 |
| 10 | rs11199297 | 122114585 | ADD | 1663 | 0.8519 | -1.159 | 0.2465 |
| 10 | rs11199302 | 122127705 | ADD | 1642 | 1.058 | 0.38 | 0.704 |
| 10 | rs1041168 | 122208645 | ADD | 1666 | 1.033 | 0.4167 | 0.6769 |
| 10 | rs4751793 | 122355150 | ADD | 1635 | 1.027 | 0.3278 | 0.7431 |
| 10 | rs11199859 | 123009541 | ADD | 1667 | 1.041 | 0.3693 | 0.7119 |
| 10 | rs7911727 | 123214869 | ADD | 1667 | 1.156 | 2.067 | 0.03877 |
| 10 | rs11597044 | 125210246 | ADD | 1634 | 0.9646 | -0.3373 | 0.7359 |
| 10 | rs4329614 | 125663537 | ADD | 1661 | 0.9017 | -1.466 | 0.1427 |
| 10 | rs1254703 | 126790332 | ADD | 1662 | 1.106 | 0.7161 | 0.4739 |
| 10 | rs10794060 | 127814909 | ADD | 1666 | 1.005 | 0.0651 | 0.9481 |
| 10 | rs7896870 | 127817674 | ADD | 1596 | 1.136 | 1.781 | 0.07494 |
| 10 | rs17154652 | 127957650 | ADD | 1667 | 0.9941 | -0.07208 | 0.9425 |
| 10 | rs11015984 | 129662632 | ADD | 1663 | 1.026 | 0.2847 | 0.7759 |
| 10 | rs7918656 | 130302400 | ADD | 1647 | 0.9072 | -1.379 | 0.1679 |
| 10 | rs4751321 | 132787419 | ADD | 1666 | 1.068 | 0.8156 | 0.4147 |
| 10 | rs4880335 | 133776155 | ADD | 1586 | 0.9271 | -0.8877 | 0.3747 |
| 11 | rs9666537 | 2642440 | ADD | 1643 | 0.9423 | -0.7537 | 0.4511 |
| 11 | rs3987740 | 2852376 | ADD | 1667 | 1.007 | 0.09667 | 0.923 |
| 11 | rs11024437 | 2865232 | ADD | 1662 | 0.9464 | -0.4409 | 0.6593 |
| 11 | rs1378738 | 4948858 | ADD | 1661 | 1.041 | 0.5692 | 0.5692 |
| 11 | rs7104040 | 6267802 | ADD | 1667 | 0.8925 | -1.541 | 0.1233 |
| 11 | rs1487863 | 7234854 | ADD | 1656 | 0.9352 | -0.9153 | 0.36 |
| 11 | rs1487864 | 7234977 | ADD | 1659 | 0.9755 | -0.2939 | 0.7688 |
| 11 | rs7946653 | 7394175 | ADD | 1664 | 0.9326 | -0.9817 | 0.3262 |
| 11 | rs1528125 | 8156792 | ADD | 1644 | 1.103 | 1.381 | 0.1673 |
| 11 | rs557096 | 10918954 | ADD | 1583 | 0.9425 | -0.6437 | 0.5198 |
| 11 | rs4441010 | 11225662 | ADD | 1631 | 0.972 | -0.3592 | 0.7194 |
| 11 | rs11021911 | 11514149 | ADD | 1588 | 0.518 | -1.727 | 0.08418 |
| 11 | rs6485587 | 12201919 | ADD | 1607 | 1.01 | 0.1261 | 0.8997 |
| 11 | rs7644 | 12508420 | ADD | 1608 | 0.9698 | -0.4074 | 0.6837 |
| 11 | rs12283632 | 14014854 | ADD | 1666 | 0.8843 | -1.587 | 0.1125 |
| 11 | rs1945607 | 15235997 | ADD | 1655 | 0.9491 | -0.7427 | 0.4576 |
| 11 | rs11024792 | 18759079 | ADD | 1666 | 0.9576 | -0.5736 | 0.5663 |
| 11 | rs4757741 | 18939210 | ADD | 1666 | 0.9265 | -1.104 | 0.2696 |
| 11 | rs1354269 | 19330820 | ADD | 1664 | 0.9802 | -0.2746 | 0.7836 |
| 11 | rs10834086 | 23416687 | ADD | 1667 | 1.089 | 1.059 | 0.2895 |
| 11 | rs4923190 | 24467816 | ADD | 1663 | 0.9322 | -0.8631 | 0.3881 |
| 11 | rs4923205 | 24708320 | ADD | 1667 | 0.8855 | -1.641 | 0.1007 |
| 11 | rs10500989 | 24708471 | ADD | 1664 | 0.9174 | -1.088 | 0.2764 |
| 11 | rs1007719 | 24750900 | ADD | 1662 | 1.047 | 0.6259 | 0.5314 |
| 11 | rs4755454 | 32859839 | ADD | 1660 | 0.925 | -1.12 | 0.2625 |
| 11 | rs2273549 | 33036156 | ADD | 1664 | 0.9586 | -0.441 | 0.6592 |
| 11 | rs10128679 | 35528490 | ADD | 1667 | 1.024 | 0.2555 | 0.7984 |
| 11 | rs1374494 | 35550147 | ADD | 1667 | 1.092 | 0.4 | 0.6892 |
| 11 | rs1512369 | 37072232 | ADD | 1647 | 0.9324 | -0.9322 | 0.3512 |
| 11 | rs11034587 | 38085211 | ADD | 1589 | 0.9875 | -0.06836 | 0.9455 |
| 11 | rs11034972 | 38816598 | ADD | 1665 | 0.953 | -0.6405 | 0.5219 |
| 11 | rs10768703 | 41522604 | ADD | 1666 | 1.245 | 1.992 | 0.04641 |
| 11 | rs16937773 | 43949775 | ADD | 1667 | 1.073 | 0.816 | 0.4145 |
| 11 | rs7929114 | 45662738 | ADD | 1667 | 0.953 | -0.6908 | 0.4897 |
| 11 | rs2165832 | 59863681 | ADD | 1666 | 1.145 | 1.869 | 0.06161 |
| 11 | rs2120182 | 59968873 | ADD | 1661 | 0.9583 | -0.5559 | 0.5783 |
| 11 | rs11227332 | 65380315 | ADD | 1667 | 1.07 | 0.7701 | 0.4413 |
| 11 | rs2510389 | 68164580 | ADD | 1654 | 1.005 | 0.04853 | 0.9613 |
| 11 | rs10896396 | 68561382 | ADD | 1667 | 0.9542 | -0.5809 | 0.5613 |
| 11 | rs10431158 | 70644645 | ADD | 1660 | 1.066 | 0.8554 | 0.3924 |
| 11 | rs879380 | 70697060 | ADD | 1652 | 0.9918 | -0.1119 | 0.9109 |
| 11 | rs11236673 | 75655493 | ADD | 1664 | 0.9694 | -0.3473 | 0.7283 |
| 11 | rs2371933 | 75657634 | ADD | 1644 | 1.128 | 1.637 | 0.1017 |
| 11 | rs7943716 | 76515147 | ADD | 1659 | 1.028 | 0.4032 | 0.6868 |
| 11 | rs489257 | 78768875 | ADD | 1665 | 0.8516 | -2.197 | 0.028 |
| 11 | rs17143592 | 81872423 | ADD | 1666 | 0.9385 | -0.7875 | 0.431 |
| 11 | rs10898549 | 86281138 | ADD | 1667 | 0.9395 | -0.7233 | 0.4695 |
| 11 | rs12224600 | 86625265 | ADD | 1660 | 1.083 | 0.5976 | 0.5501 |
| 11 | rs4488224 | 86899478 | ADD | 1659 | 1.154 | 1.907 | 0.05656 |
| 11 | rs16913876 | 87742387 | ADD | 1662 | 0.7788 | -1.398 | 0.162 |
| 11 | rs1439528 | 96115783 | ADD | 1647 | 0.9954 | -0.06628 | 0.9472 |
| 11 | rs11212509 | 107461981 | ADD | 1662 | 1.009 | 0.09907 | 0.9211 |
| 11 | rs17117563 | 114203979 | ADD | 1667 | 1.116 | 1.03 | 0.303 |
| 11 | rs920447 | 115614112 | ADD | 1664 | 0.8837 | -1.357 | 0.1748 |
| 11 | rs17120197 | 116350314 | ADD | 1651 | 1.121 | 1.298 | 0.1943 |
| 11 | rs11217364 | 119006231 | ADD | 1644 | 1.034 | 0.4728 | 0.6364 |
| 11 | rs17092876 | 119903853 | ADD | 1666 | 1.032 | 0.4345 | 0.6639 |
| 11 | rs2887763 | 121073389 | ADD | 1602 | 0.9487 | -0.7227 | 0.4699 |
| 11 | rs531743 | 121625352 | ADD | 1667 | 0.9781 | -0.182 | 0.8556 |
| 11 | rs863955 | 124757151 | ADD | 1665 | 1.033 | 0.4477 | 0.6544 |
| 11 | rs635819 | 125296227 | ADD | 1666 | 1.042 | 0.4714 | 0.6374 |
| 11 | rs11221163 | 127506541 | ADD | 1665 | 0.9532 | -0.6966 | 0.486 |
| 11 | rs613587 | 128138519 | ADD | 1666 | 1.03 | 0.3872 | 0.6986 |
| 11 | rs6590359 | 128311024 | ADD | 1667 | 0.9805 | -0.1955 | 0.845 |
| 11 | rs1031063 | 128587624 | ADD | 1660 | 0.9664 | -0.4628 | 0.6435 |
| 11 | rs1493547 | 128755737 | ADD | 1665 | 0.9253 | -0.8547 | 0.3927 |
| 11 | rs6590400 | 129005055 | ADD | 1651 | 1.03 | 0.4263 | 0.6699 |
| 11 | rs1630972 | 130591101 | ADD | 1649 | 1.052 | 0.6901 | 0.4902 |
| 11 | rs10894396 | 130831245 | ADD | 1658 | 1.032 | 0.4347 | 0.6638 |
| 11 | rs12276840 | 131853608 | ADD | 1657 | 0.9977 | -0.03228 | 0.9742 |
| 11 | rs10791275 | 132444980 | ADD | 1663 | 0.912 | -1.036 | 0.3 |
| 11 | rs4937752 | 132537965 | ADD | 1667 | 1.065 | 0.7478 | 0.4546 |
| 11 | rs4937775 | 132822925 | ADD | 1665 | 1.15 | 2.069 | 0.03854 |
| 12 | rs7298766 | 531917 | ADD | 1667 | 1.098 | 1.239 | 0.2152 |
| 12 | rs11064294 | 675712 | ADD | 1664 | 1.031 | 0.3416 | 0.7327 |
| 12 | rs2532560 | 4032132 | ADD | 1667 | 1.237 | 2.423 | 0.01537 |
| 12 | rs11063027 | 4169574 | ADD | 1664 | 1.01 | 0.09252 | 0.9263 |
| 12 | rs6489530 | 4229392 | ADD | 1633 | 1.259 | 1.184 | 0.2363 |
| 12 | rs997868 | 4569493 | ADD | 1667 | 1.081 | 1.008 | 0.3133 |
| 12 | rs10849439 | 6285592 | ADD | 1613 | 0.9449 | -0.6651 | 0.506 |
| 12 | rs4418897 | 9859080 | ADD | 1624 | 0.997 | -0.03732 | 0.9702 |
| 12 | rs2724610 | 11737633 | ADD | 1654 | 0.9509 | -0.7397 | 0.4595 |
| 12 | rs3825271 | 13107338 | ADD | 1667 | 1.02 | 0.1092 | 0.9131 |
| 12 | rs717061 | 13549472 | ADD | 1661 | 0.8771 | -1.071 | 0.2843 |
| 12 | rs12815398 | 19115452 | ADD | 1665 | 1.036 | 0.331 | 0.7407 |
| 12 | rs16925543 | 22987781 | ADD | 1666 | 1.066 | 0.5637 | 0.5729 |
| 12 | rs1011185 | 25514998 | ADD | 1662 | 0.9424 | -0.8163 | 0.4143 |
| 12 | rs1531680 | 28840469 | ADD | 1660 | 1.063 | 0.8746 | 0.3818 |
| 12 | rs12823875 | 29701392 | ADD | 1657 | 1.018 | 0.2475 | 0.8045 |
| 12 | rs6487842 | 29709471 | ADD | 1662 | 1.032 | 0.4445 | 0.6567 |
| 12 | rs7972685 | 29872973 | ADD | 1666 | 1.019 | 0.1822 | 0.8554 |
| 12 | rs12313180 | 30200135 | ADD | 1662 | 1.115 | 1.515 | 0.1298 |
| 12 | rs9971825 | 31390938 | ADD | 1660 | 1.061 | 0.848 | 0.3964 |
| 12 | rs7963790 | 38515289 | ADD | 1665 | 0.9563 | -0.5857 | 0.5581 |
| 12 | rs11177589 | 39327131 | ADD | 1666 | 1.076 | 0.9239 | 0.3555 |
| 12 | rs11181126 | 40404909 | ADD | 1664 | 1.129 | 1.731 | 0.08339 |
| 12 | rs10491998 | 40585149 | ADD | 1597 | 1.044 | 0.3894 | 0.697 |
| 12 | rs7138803 | 48533735 | ADD | 1643 | 1.159 | 1.971 | 0.04869 |
| 12 | rs7133633 | 52426199 | ADD | 1667 | 0.9935 | -0.08767 | 0.9301 |
| 12 | rs11173096 | 58279092 | ADD | 1665 | 0.9607 | -0.55 | 0.5823 |
| 12 | rs7305206 | 61002597 | ADD | 1665 | 0.9689 | -0.2729 | 0.785 |
| 12 | rs4547160 | 61789917 | ADD | 1650 | 1.003 | 0.03961 | 0.9684 |
| 12 | rs6581482 | 61969165 | ADD | 1665 | 0.9751 | -0.3321 | 0.7398 |
| 12 | rs11176078 | 64861524 | ADD | 1665 | 0.7681 | -3.293 | 0.0009895 |
| 12 | rs4283063 | 65367426 | ADD | 1666 | 0.9761 | -0.3421 | 0.7323 |
| 12 | rs10506555 | 66195617 | ADD | 1663 | 1.082 | 1.029 | 0.3035 |
| 12 | rs1616138 | 66322984 | ADD | 1665 | 1.032 | 0.4488 | 0.6535 |
| 12 | rs2906853 | 66672279 | ADD | 1665 | 0.9881 | -0.1722 | 0.8632 |
| 12 | rs2127536 | 67030718 | ADD | 1666 | 0.8893 | -1.553 | 0.1205 |
| 12 | rs11177718 | 68241952 | ADD | 1641 | 0.714 | -1.962 | 0.0498 |
| 12 | rs11178820 | 70156158 | ADD | 1667 | 0.9035 | -1.344 | 0.1788 |
| 12 | rs7314267 | 74578155 | ADD | 1666 | 0.9916 | -0.08341 | 0.9335 |
| 12 | rs6539534 | 79775075 | ADD | 1667 | 0.9364 | -0.9368 | 0.3489 |
| 12 | rs7310349 | 79786689 | ADD | 1663 | 1.134 | 1.699 | 0.08937 |
| 12 | rs12580477 | 80760658 | ADD | 1660 | 1.114 | 1.318 | 0.1876 |
| 12 | rs10862940 | 83759289 | ADD | 1598 | 0.9802 | -0.1846 | 0.8536 |
| 12 | rs10859024 | 89600629 | ADD | 1658 | 0.9922 | -0.09981 | 0.9205 |
| 12 | rs7969076 | 92620173 | ADD | 1665 | 0.8751 | -1.902 | 0.05715 |
| 12 | rs10859569 | 92655442 | ADD | 1666 | 1.022 | 0.3016 | 0.7629 |
| 12 | rs733752 | 92807103 | ADD | 1665 | 1.066 | 0.4369 | 0.6622 |
| 12 | rs7134784 | 93106608 | ADD | 1647 | 1.109 | 1.092 | 0.2749 |
| 12 | rs10859684 | 93118690 | ADD | 1666 | 1.033 | 0.4541 | 0.6498 |
| 12 | rs7972350 | 93684736 | ADD | 1656 | 1.001 | 0.01613 | 0.9871 |
| 12 | rs10777715 | 94553462 | ADD | 1665 | 1.009 | 0.1277 | 0.8984 |
| 12 | rs759394 | 94983473 | ADD | 1654 | 1.024 | 0.3332 | 0.739 |
| 12 | rs169302 | 97764966 | ADD | 1641 | 1.088 | 1.02 | 0.3078 |
| 12 | rs11110822 | 100409830 | ADD | 1663 | 1.049 | 0.3798 | 0.7041 |
| 12 | rs919214 | 100694908 | ADD | 1664 | 1.023 | 0.2559 | 0.798 |
| 12 | rs4255604 | 103097845 | ADD | 1664 | 0.8999 | -0.8503 | 0.3952 |
| 12 | rs835481 | 103573044 | ADD | 1660 | 1.062 | 0.7909 | 0.429 |
| 12 | rs12426430 | 104529168 | ADD | 1650 | 1.049 | 0.6005 | 0.5482 |
| 12 | rs7960241 | 107263514 | ADD | 1661 | 1.079 | 1.022 | 0.3068 |
| 12 | rs1865147 | 107916997 | ADD | 1665 | 1.096 | 1.269 | 0.2046 |
| 12 | rs2075263 | 108188708 | ADD | 1666 | 0.9661 | -0.3756 | 0.7072 |
| 12 | rs4766722 | 113074817 | ADD | 1664 | 1.064 | 0.8218 | 0.4112 |
| 12 | rs10774792 | 114036981 | ADD | 1667 | 1.14 | 1.839 | 0.06595 |
| 12 | rs11067690 | 114559145 | ADD | 1660 | 0.998 | -0.02796 | 0.9777 |
| 12 | rs11067772 | 114712808 | ADD | 1666 | 1.039 | 0.5132 | 0.6078 |
| 12 | rs1151899 | 115299808 | ADD | 1665 | 0.8577 | -2.141 | 0.03228 |
| 12 | rs605265 | 116620279 | ADD | 1660 | 1.22 | 2.821 | 0.004786 |
| 12 | rs7302554 | 118014807 | ADD | 1640 | 0.9491 | -0.6849 | 0.4934 |
| 12 | rs4758677 | 121241034 | ADD | 1589 | 0.9149 | -1.055 | 0.2912 |
| 12 | rs989640 | 125121680 | ADD | 1589 | 0.9202 | -1.067 | 0.2861 |
| 12 | rs2398428 | 127801519 | ADD | 1663 | 0.9673 | -0.4079 | 0.6834 |
| 12 | rs10773613 | 128266650 | ADD | 1664 | 1.064 | 0.8648 | 0.3871 |
| 12 | rs10847896 | 128583266 | ADD | 1638 | 1.036 | 0.4248 | 0.671 |
| 12 | rs11060784 | 129272960 | ADD | 1667 | 1.124 | 0.6957 | 0.4866 |
| 13 | rs17064065 | 18532794 | ADD | 1662 | 1.148 | 0.5367 | 0.5915 |
| 13 | rs17378638 | 21644539 | ADD | 1663 | 1.115 | 1.223 | 0.2215 |
| 13 | rs7331655 | 21684026 | ADD | 1663 | 0.9578 | -0.6145 | 0.5389 |
| 13 | rs4769240 | 22595921 | ADD | 1664 | 0.8326 | -1.168 | 0.2429 |
| 13 | rs942876 | 23512951 | ADD | 1636 | 1.047 | 0.6238 | 0.5327 |
| 13 | rs1536205 | 23533901 | ADD | 1643 | 0.946 | -0.7183 | 0.4726 |
| 13 | rs9508066 | 28050505 | ADD | 1663 | 0.866 | -1.864 | 0.06239 |
| 13 | rs2453703 | 28339809 | ADD | 1637 | 1.05 | 0.6559 | 0.5119 |
| 13 | rs1331697 | 30031133 | ADD | 1666 | 1.246 | 3.057 | 0.002239 |
| 13 | rs10162089 | 30214738 | ADD | 1661 | 1.011 | 0.1516 | 0.8795 |
| 13 | rs2858822 | 32580194 | ADD | 1667 | 0.9287 | -0.8393 | 0.4013 |
| 13 | rs9540823 | 33895343 | ADD | 1666 | 1.07 | 1.006 | 0.3145 |
| 13 | rs11618012 | 34237417 | ADD | 1667 | 1.378 | 3.528 | 0.0004188 |
| 13 | rs9315310 | 34249431 | ADD | 1599 | 1.229 | 2.364 | 0.01807 |
| 13 | rs1171066 | 35359920 | ADD | 1631 | 0.7054 | -1.956 | 0.05041 |
| 13 | rs7994174 | 35573018 | ADD | 1666 | 1.161 | 1.416 | 0.1566 |
| 13 | rs7985641 | 38454142 | ADD | 1667 | 0.9499 | -0.6319 | 0.5274 |
| 13 | rs1186468 | 38847162 | ADD | 1665 | 1 | 0.002115 | 0.9983 |
| 13 | rs2324614 | 39902048 | ADD | 1652 | 1.039 | 0.4086 | 0.6828 |
| 13 | rs1410942 | 43086455 | ADD | 1658 | 1.079 | 0.567 | 0.5707 |
| 13 | rs1536207 | 45013732 | ADD | 1664 | 0.9251 | -1.098 | 0.2724 |
| 13 | rs582854 | 46343878 | ADD | 1631 | 0.8641 | -2.041 | 0.04123 |
| 13 | rs2478327 | 47021448 | ADD | 1667 | 1.109 | 1.505 | 0.1324 |
| 13 | rs8181889 | 52511991 | ADD | 1666 | 1.004 | 0.05845 | 0.9534 |
| 13 | rs1891948 | 52537146 | ADD | 1637 | 0.9315 | -0.9916 | 0.3214 |
| 13 | rs7985340 | 58536705 | ADD | 1664 | 1.142 | 1.301 | 0.1931 |
| 13 | rs4884662 | 65721667 | ADD | 1662 | 0.9779 | -0.3221 | 0.7474 |
| 13 | rs3003202 | 69469515 | ADD | 1661 | 1.008 | 0.1002 | 0.9202 |
| 13 | rs9599553 | 69605722 | ADD | 1655 | 0.9365 | -0.767 | 0.4431 |
| 13 | rs9542259 | 69763894 | ADD | 1629 | 1.014 | 0.163 | 0.8705 |
| 13 | rs9542293 | 69856709 | ADD | 1654 | 0.9851 | -0.1628 | 0.8707 |
| 13 | rs9529804 | 70215962 | ADD | 1666 | 0.9516 | -0.6628 | 0.5074 |
| 13 | rs1965830 | 70239508 | ADD | 1667 | 1.043 | 0.5935 | 0.5528 |
| 13 | rs4506781 | 70626126 | ADD | 1665 | 1.005 | 0.0716 | 0.9429 |
| 13 | rs7995527 | 70740432 | ADD | 1665 | 0.9799 | -0.2937 | 0.7689 |
| 13 | rs1218307 | 78315833 | ADD | 1667 | 0.9795 | -0.2927 | 0.7698 |
| 13 | rs4310745 | 80383365 | ADD | 1661 | 1.079 | 1.064 | 0.2875 |
| 13 | rs17078997 | 84470421 | ADD | 1662 | 1.085 | 0.7615 | 0.4464 |
| 13 | rs9301668 | 89726966 | ADD | 1661 | 0.9132 | -1.281 | 0.2002 |
| 13 | rs9301862 | 92656760 | ADD | 1650 | 1.224 | 2.896 | 0.003783 |
| 13 | rs9300342 | 95749951 | ADD | 1666 | 1.18 | 2.358 | 0.01835 |
| 13 | rs7329296 | 96584269 | ADD | 1653 | 1.091 | 1.168 | 0.243 |
| 13 | rs4771988 | 96709564 | ADD | 1614 | 1.022 | 0.2805 | 0.7791 |
| 13 | rs1160294 | 98731595 | ADD | 1651 | 1.005 | 0.07691 | 0.9387 |
| 13 | rs1028711 | 99280688 | ADD | 1667 | 0.9474 | -0.7548 | 0.4504 |
| 13 | rs9518426 | 100960081 | ADD | 1663 | 0.931 | -0.6668 | 0.5049 |
| 13 | rs875118 | 100973809 | ADD | 1666 | 1.03 | 0.3999 | 0.6892 |
| 13 | rs1898199 | 101102895 | ADD | 1657 | 1.058 | 0.7571 | 0.449 |
| 13 | rs7327500 | 101650093 | ADD | 1667 | 0.926 | -0.8263 | 0.4087 |
| 13 | rs1537197 | 103196429 | ADD | 1667 | 0.9259 | -0.7352 | 0.4622 |
| 13 | rs778330 | 104952561 | ADD | 1667 | 1.067 | 0.7229 | 0.4698 |
| 13 | rs7992416 | 106070115 | ADD | 1667 | 1.047 | 0.6327 | 0.5269 |
| 13 | rs7999734 | 108778942 | ADD | 1659 | 1.082 | 0.8142 | 0.4155 |
| 13 | rs1887638 | 108824695 | ADD | 1641 | 1.089 | 1.157 | 0.2472 |
| 13 | rs831167 | 110593545 | ADD | 1666 | 0.9267 | -0.7031 | 0.482 |
| 13 | rs7986656 | 113157383 | ADD | 1653 | 1.097 | 1.067 | 0.286 |
| 13 | rs389862 | 113948331 | ADD | 1667 | 1.164 | 1.371 | 0.1703 |
| 14 | rs6576284 | 20116652 | ADD | 1665 | 1.002 | 0.02652 | 0.9788 |
| 14 | rs10483263 | 21448675 | ADD | 1665 | 1.325 | 2.647 | 0.008112 |
| 14 | rs1885592 | 23207855 | ADD | 1666 | 1.003 | 0.04936 | 0.9606 |
| 14 | rs7154849 | 24196182 | ADD | 1652 | 0.8892 | -1.635 | 0.102 |
| 14 | rs11159287 | 24960206 | ADD | 1650 | 0.9288 | -0.5884 | 0.5562 |
| 14 | rs1950419 | 26223900 | ADD | 1660 | 1.077 | 0.6601 | 0.5092 |
| 14 | rs2378931 | 31042040 | ADD | 1667 | 0.9849 | -0.2251 | 0.8219 |
| 14 | rs1950702 | 32110983 | ADD | 1665 | 0.9688 | -0.3636 | 0.7161 |
| 14 | rs7144454 | 32721646 | ADD | 1667 | 1.021 | 0.2805 | 0.7791 |
| 14 | rs967440 | 32977498 | ADD | 1667 | 1.025 | 0.2755 | 0.7829 |
| 14 | rs4981220 | 33669097 | ADD | 1652 | 0.9678 | -0.4264 | 0.6698 |
| 14 | rs848086 | 36349467 | ADD | 1667 | 0.8654 | -1.484 | 0.1378 |
| 14 | rs10483489 | 36689921 | ADD | 1666 | 0.9141 | -0.8272 | 0.4081 |
| 14 | rs12590869 | 42210086 | ADD | 1656 | 1.045 | 0.4483 | 0.6539 |
| 14 | rs7159152 | 43532155 | ADD | 1602 | 0.9265 | -1.081 | 0.2799 |
| 14 | rs2022759 | 43894624 | ADD | 1666 | 1.057 | 0.5432 | 0.587 |
| 14 | rs34774961 | 47505219 | ADD | 1666 | 0.9843 | -0.2022 | 0.8398 |
| 14 | rs7150200 | 47780011 | ADD | 1631 | 0.9978 | -0.02678 | 0.9786 |
| 14 | rs17123103 | 50438245 | ADD | 1646 | 1.107 | 1.196 | 0.2318 |
| 14 | rs1557191 | 51762335 | ADD | 1664 | 1.028 | 0.3616 | 0.7176 |
| 14 | rs2761887 | 53494802 | ADD | 1659 | 0.9642 | -0.5113 | 0.6092 |
| 14 | rs8014071 | 53501250 | ADD | 1659 | 0.9573 | -0.6209 | 0.5346 |
| 14 | rs10498479 | 55415998 | ADD | 1667 | 0.7046 | -2.456 | 0.01406 |
| 14 | rs1679804 | 55563222 | ADD | 1666 | 1.098 | 1.294 | 0.1956 |
| 14 | rs1188690 | 55868252 | ADD | 1659 | 1.048 | 0.6268 | 0.5308 |
| 14 | rs1152492 | 55869750 | ADD | 1667 | 1.08 | 1.084 | 0.2785 |
| 14 | rs4312236 | 61054788 | ADD | 1662 | 1.005 | 0.04781 | 0.9619 |
| 14 | rs4902490 | 67112008 | ADD | 1666 | 0.9605 | -0.5735 | 0.5663 |
| 14 | rs1561193 | 68388004 | ADD | 1666 | 1.031 | 0.2042 | 0.8382 |
| 14 | rs2293796 | 72790915 | ADD | 1665 | 0.9967 | -0.04228 | 0.9663 |
| 14 | rs17835229 | 78097257 | ADD | 1648 | 0.9452 | -0.6592 | 0.5098 |
| 14 | rs10483938 | 79451730 | ADD | 1661 | 0.9612 | -0.3769 | 0.7063 |
| 14 | rs1242444 | 82391377 | ADD | 1667 | 1.173 | 0.8915 | 0.3727 |
| 14 | rs8012291 | 83422711 | ADD | 1654 | 0.9977 | -0.02255 | 0.982 |
| 14 | rs11845781 | 88346184 | ADD | 1665 | 0.9328 | -1.005 | 0.315 |
| 14 | rs11622455 | 90739682 | ADD | 1664 | 1.072 | 0.7357 | 0.4619 |
| 14 | rs917908 | 91433539 | ADD | 1661 | 1.034 | 0.3564 | 0.7216 |
| 14 | rs7152962 | 91964791 | ADD | 1667 | 1.005 | 0.05425 | 0.9567 |
| 14 | rs10140983 | 93427956 | ADD | 1666 | 1.006 | 0.04806 | 0.9617 |
| 14 | rs10747304 | 95686613 | ADD | 1660 | 1.126 | 1.731 | 0.08337 |
| 14 | rs234600 | 96150354 | ADD | 1659 | 0.9878 | -0.1789 | 0.858 |
| 14 | rs1269096 | 98672872 | ADD | 1665 | 1.054 | 0.7721 | 0.44 |
| 14 | rs17098899 | 99148653 | ADD | 1665 | 1.186 | 1.478 | 0.1393 |
| 14 | rs17098912 | 99174082 | ADD | 1663 | 1.059 | 0.598 | 0.5498 |
| 14 | rs1190967 | 99605854 | ADD | 1664 | 0.9519 | -0.6839 | 0.494 |
| 14 | rs7158663 | 100389177 | ADD | 1661 | 0.8988 | -1.557 | 0.1194 |
| 14 | rs710100 | 102672031 | ADD | 1651 | 1.035 | 0.4754 | 0.6345 |
| 14 | rs12892765 | 102846524 | ADD | 1666 | 1.175 | 1.597 | 0.1102 |
| 15 | rs8031166 | 21633826 | ADD | 1666 | 1.228 | 2.828 | 0.004678 |
| 15 | rs16950272 | 25552496 | ADD | 1666 | 0.9751 | -0.3384 | 0.7351 |
| 15 | rs2311843 | 25856949 | ADD | 1663 | 0.977 | -0.2529 | 0.8004 |
| 15 | rs4780121 | 31547408 | ADD | 1633 | 0.9162 | -1.074 | 0.2828 |
| 15 | rs10775211 | 31720282 | ADD | 1665 | 1.038 | 0.5129 | 0.608 |
| 15 | rs4923735 | 32757965 | ADD | 1618 | 1.028 | 0.2315 | 0.8169 |
| 15 | rs2070664 | 32872493 | ADD | 1662 | 1.046 | 0.6273 | 0.5305 |
| 15 | rs4502172 | 34114820 | ADD | 1665 | 0.9509 | -0.718 | 0.4727 |
| 15 | rs17536109 | 34130070 | ADD | 1663 | 1.096 | 1.297 | 0.1945 |
| 15 | rs2444728 | 34536722 | ADD | 1663 | 1.181 | 1.085 | 0.2777 |
| 15 | rs16965556 | 35846980 | ADD | 1666 | 0.9966 | -0.0356 | 0.9716 |
| 15 | rs16954437 | 36914379 | ADD | 1663 | 1.128 | 1.133 | 0.2573 |
| 15 | rs542036 | 41328431 | ADD | 1659 | 1.081 | 0.9901 | 0.3221 |
| 15 | rs2218309 | 43725953 | ADD | 1664 | 1.118 | 1.056 | 0.2908 |
| 15 | rs606670 | 47799141 | ADD | 1667 | 0.9752 | -0.156 | 0.876 |
| 15 | rs16963311 | 48192467 | ADD | 1665 | 0.9662 | -0.3119 | 0.7551 |
| 15 | rs1996309 | 51491250 | ADD | 1665 | 1.07 | 0.9231 | 0.3559 |
| 15 | rs2459367 | 51824412 | ADD | 1667 | 1.057 | 0.7428 | 0.4576 |
| 15 | rs4775229 | 58081006 | ADD | 1667 | 1.02 | 0.2206 | 0.8254 |
| 15 | rs8042883 | 64757325 | ADD | 1649 | 1.045 | 0.6276 | 0.5303 |
| 15 | rs2168010 | 66857590 | ADD | 1663 | 0.9654 | -0.3407 | 0.7333 |
| 15 | rs8036528 | 68218165 | ADD | 1666 | 0.9627 | -0.4191 | 0.6751 |
| 15 | rs16954285 | 68286152 | ADD | 1662 | 0.8973 | -0.933 | 0.3508 |
| 15 | rs10518929 | 69082246 | ADD | 1633 | 0.9923 | -0.1015 | 0.9192 |
| 15 | rs17773784 | 71867727 | ADD | 1661 | 1.078 | 0.7965 | 0.4257 |
| 15 | rs999742 | 71902570 | ADD | 1667 | 0.9164 | -0.9901 | 0.3221 |
| 15 | rs10851883 | 73778506 | ADD | 1660 | 1.071 | 0.9978 | 0.3184 |
| 15 | rs4886456 | 73802922 | ADD | 1664 | 1.042 | 0.5893 | 0.5556 |
| 15 | rs4779101 | 77587096 | ADD | 1658 | 0.941 | -0.8592 | 0.3902 |
| 15 | rs17183085 | 77628356 | ADD | 1667 | 0.7787 | -1.867 | 0.06186 |
| 15 | rs2654191 | 80149180 | ADD | 1662 | 1.086 | 0.9246 | 0.3552 |
| 15 | rs4778973 | 80181060 | ADD | 1666 | 1.073 | 0.9796 | 0.3273 |
| 15 | rs1553883 | 81269403 | ADD | 1667 | 0.9246 | -0.8864 | 0.3754 |
| 15 | rs10520609 | 84481778 | ADD | 1641 | 0.9439 | -0.5069 | 0.6122 |
| 15 | rs8031104 | 84627087 | ADD | 1662 | 0.9411 | -0.8893 | 0.3738 |
| 15 | rs11633107 | 84680494 | ADD | 1667 | 0.9932 | -0.09733 | 0.9225 |
| 15 | rs11633969 | 85221240 | ADD | 1633 | 0.99 | -0.1364 | 0.8915 |
| 15 | rs2881640 | 85261718 | ADD | 1667 | 0.8866 | -1.407 | 0.1594 |
| 15 | rs977133 | 85807138 | ADD | 1646 | 0.9376 | -0.8644 | 0.3873 |
| 15 | rs16942719 | 87471831 | ADD | 1667 | 0.9858 | -0.1372 | 0.8909 |
| 15 | rs2589967 | 88708880 | ADD | 1649 | 1.184 | 2.068 | 0.03866 |
| 15 | rs8025225 | 90741904 | ADD | 1667 | 0.9215 | -1.173 | 0.241 |
| 15 | rs17540498 | 91601429 | ADD | 1666 | 1.035 | 0.4667 | 0.6407 |
| 15 | rs8041151 | 92427459 | ADD | 1667 | 0.9876 | -0.1667 | 0.8676 |
| 15 | rs1471150 | 93347701 | ADD | 1666 | 1.038 | 0.5344 | 0.5931 |
| 15 | rs6496245 | 96054222 | ADD | 1667 | 1.128 | 1.758 | 0.07872 |
| 15 | rs11632922 | 97908966 | ADD | 1664 | 0.985 | -0.2191 | 0.8266 |
| 15 | rs2727184 | 98393341 | ADD | 1624 | 0.9662 | -0.2833 | 0.7769 |
| 15 | rs7183000 | 98508979 | ADD | 1667 | 1.034 | 0.4734 | 0.6359 |
| 15 | rs1689936 | 99040267 | ADD | 1663 | 1.092 | 1.205 | 0.2282 |
| 15 | rs1598492 | 99395057 | ADD | 1667 | 0.8326 | -2.356 | 0.01847 |
| 15 | rs7175368 | 99704278 | ADD | 1660 | 0.8995 | -1.359 | 0.1742 |
| 16 | rs1076573 | 3170889 | ADD | 1666 | 0.9461 | -0.7037 | 0.4816 |
| 16 | rs2238418 | 3815169 | ADD | 1667 | 0.5276 | -1.713 | 0.08678 |
| 16 | rs11640110 | 5546417 | ADD | 1656 | 0.927 | -1.075 | 0.2822 |
| 16 | rs4786784 | 5840278 | ADD | 1666 | 1.032 | 0.4239 | 0.6717 |
| 16 | rs7200256 | 5843318 | ADD | 1665 | 1.026 | 0.345 | 0.7301 |
| 16 | rs8050038 | 5957977 | ADD | 1660 | 0.9431 | -0.7894 | 0.4299 |
| 16 | rs8050137 | 7391457 | ADD | 1665 | 1.039 | 0.3433 | 0.7314 |
| 16 | rs7186099 | 7501334 | ADD | 1660 | 1.149 | 1.47 | 0.1415 |
| 16 | rs720763 | 9473575 | ADD | 1644 | 1.035 | 0.4856 | 0.6272 |
| 16 | rs16959859 | 12543114 | ADD | 1663 | 1.165 | 1.481 | 0.1387 |
| 16 | rs7194966 | 12635395 | ADD | 1582 | 0.8432 | -2.369 | 0.01782 |
| 16 | rs150071 | 13154856 | ADD | 1662 | 0.9324 | -1.012 | 0.3114 |
| 16 | rs3751877 | 15062399 | ADD | 1667 | 0.9581 | -0.3948 | 0.693 |
| 16 | rs12935466 | 24407049 | ADD | 1665 | 1.044 | 0.5948 | 0.5519 |
| 16 | rs4788003 | 27806901 | ADD | 1666 | 0.9461 | -0.7506 | 0.4529 |
| 16 | rs4784045 | 57094125 | ADD | 1667 | 0.9338 | -0.9915 | 0.3215 |
| 16 | rs9927197 | 57568279 | ADD | 1666 | 1.021 | 0.2283 | 0.8194 |
| 16 | rs11075517 | 62338554 | ADD | 1661 | 0.9799 | -0.2876 | 0.7737 |
| 16 | rs13329873 | 63454604 | ADD | 1646 | 0.8077 | -1.019 | 0.3083 |
| 16 | rs9933029 | 66860911 | ADD | 1642 | 0.9527 | -0.6671 | 0.5047 |
| 16 | rs9923061 | 74881862 | ADD | 1658 | 0.9968 | -0.02792 | 0.9777 |
| 16 | rs2738545 | 77186821 | ADD | 1667 | 1.054 | 0.7758 | 0.4379 |
| 16 | rs1477417 | 77318865 | ADD | 1652 | 0.9496 | -0.5892 | 0.5558 |
| 16 | rs12444393 | 77998819 | ADD | 1666 | 1.029 | 0.3283 | 0.7427 |
| 16 | rs7204357 | 78810372 | ADD | 1663 | 0.8608 | -1.525 | 0.1273 |
| 16 | rs9926366 | 79645030 | ADD | 1666 | 0.8638 | -1.041 | 0.298 |
| 16 | rs12924670 | 79990571 | ADD | 1665 | 1 | 0.003262 | 0.9974 |
| 16 | rs4782872 | 80986567 | ADD | 1666 | 1.016 | 0.2356 | 0.8137 |
| 16 | rs244783 | 82917556 | ADD | 1665 | 1.087 | 1.092 | 0.2748 |
| 16 | rs4843460 | 85382973 | ADD | 1661 | 1.116 | 1.543 | 0.1227 |
| 17 | rs2360735 | 450509 | ADD | 1658 | 1.029 | 0.3337 | 0.7386 |
| 17 | rs11871557 | 5599344 | ADD | 1665 | 0.9978 | -0.03118 | 0.9751 |
| 17 | rs4602096 | 7414181 | ADD | 1667 | 1.022 | 0.2818 | 0.7781 |
| 17 | rs9897780 | 8500491 | ADD | 1652 | 1.024 | 0.2643 | 0.7916 |
| 17 | rs12944446 | 8675465 | ADD | 1662 | 1.086 | 0.9337 | 0.3505 |
| 17 | rs11654567 | 9185149 | ADD | 1659 | 1.046 | 0.6393 | 0.5226 |
| 17 | rs16944856 | 11335292 | ADD | 1641 | 0.9237 | -0.5861 | 0.5578 |
| 17 | rs1974700 | 11336115 | ADD | 1665 | 0.9463 | -0.7158 | 0.4741 |
| 17 | rs9903786 | 13370390 | ADD | 1601 | 1.025 | 0.3468 | 0.7288 |
| 17 | rs12951251 | 14645763 | ADD | 1620 | 1.016 | 0.1999 | 0.8416 |
| 17 | rs16950913 | 14893723 | ADD | 1665 | 1.014 | 0.1952 | 0.8452 |
| 17 | rs11078296 | 15249408 | ADD | 1667 | 0.9583 | -0.6005 | 0.5482 |
| 17 | rs597985 | 16311498 | ADD | 1649 | 1.057 | 0.734 | 0.463 |
| 17 | rs11080055 | 23673851 | ADD | 1666 | 1.008 | 0.1151 | 0.9083 |
| 17 | rs140701 | 25562658 | ADD | 1618 | 1.061 | 0.8623 | 0.3885 |
| 17 | rs319763 | 28589146 | ADD | 1666 | 0.9387 | -0.9035 | 0.3663 |
| 17 | rs1109593 | 32346901 | ADD | 1661 | 1.102 | 1.271 | 0.2037 |
| 17 | rs9330251 | 32663451 | ADD | 1649 | 1.092 | 0.6447 | 0.5192 |
| 17 | rs2023906 | 35967212 | ADD | 1664 | 0.9154 | -1.044 | 0.2964 |
| 17 | rs1859251 | 36350144 | ADD | 1651 | 1.03 | 0.3142 | 0.7534 |
| 17 | rs4987082 | 44836373 | ADD | 1662 | 1.085 | 1.187 | 0.2353 |
| 17 | rs16950363 | 47109472 | ADD | 1666 | 1.071 | 0.5835 | 0.5596 |
| 17 | rs10515044 | 49047083 | ADD | 1651 | 0.9868 | -0.1944 | 0.8458 |
| 17 | rs17759236 | 51534381 | ADD | 1663 | 0.8539 | -2.2 | 0.02779 |
| 17 | rs8082249 | 52937931 | ADD | 1667 | 0.894 | -1.566 | 0.1174 |
| 17 | rs1024637 | 55393675 | ADD | 1665 | 1.056 | 0.8153 | 0.4149 |
| 17 | rs16945628 | 57222011 | ADD | 1667 | 0.9699 | -0.4273 | 0.6691 |
| 17 | rs9892756 | 61153269 | ADD | 1665 | 0.9684 | -0.4616 | 0.6443 |
| 17 | rs4791021 | 62288734 | ADD | 1667 | 1.112 | 1.506 | 0.1321 |
| 17 | rs4791017 | 62313154 | ADD | 1664 | 1.017 | 0.2 | 0.8415 |
| 17 | rs16972958 | 63942484 | ADD | 1664 | 1.006 | 0.05229 | 0.9583 |
| 17 | rs2952283 | 63988022 | ADD | 1667 | 1.01 | 0.1283 | 0.8979 |
| 17 | rs10852738 | 63998422 | ADD | 1662 | 1.103 | 0.9611 | 0.3365 |
| 17 | rs4968909 | 64154289 | ADD | 1645 | 0.9636 | -0.526 | 0.5989 |
| 17 | rs4793324 | 66519356 | ADD | 1666 | 1.024 | 0.2803 | 0.7792 |
| 17 | rs11654929 | 72820946 | ADD | 1641 | 1.234 | 2.515 | 0.01191 |
| 17 | rs1108366 | 74069538 | ADD | 1664 | 1.049 | 0.4958 | 0.6201 |
| 17 | rs7211994 | 75767109 | ADD | 1667 | 0.9198 | -1.2 | 0.23 |
| 17 | rs8065364 | 75777554 | ADD | 1665 | 0.9822 | -0.2393 | 0.8108 |
| 18 | rs16953183 | 934533 | ADD | 1667 | 1.004 | 0.05209 | 0.9585 |
| 18 | rs17521158 | 2156794 | ADD | 1666 | 0.9278 | -1.008 | 0.3137 |
| 18 | rs2096823 | 2161031 | ADD | 1667 | 0.9441 | -0.8379 | 0.4021 |
| 18 | rs6506201 | 4395472 | ADD | 1667 | 1.002 | 0.02533 | 0.9798 |
| 18 | rs1785062 | 4579960 | ADD | 1666 | 1.162 | 1.564 | 0.1179 |
| 18 | rs906800 | 7450379 | ADD | 1667 | 1.175 | 1.089 | 0.2763 |
| 18 | rs6506598 | 8553622 | ADD | 1658 | 1.023 | 0.3129 | 0.7544 |
| 18 | rs7241781 | 9478704 | ADD | 1667 | 0.9128 | -1.136 | 0.2559 |
| 18 | rs329017 | 9482196 | ADD | 1664 | 0.9943 | -0.07729 | 0.9384 |
| 18 | rs1942150 | 9528533 | ADD | 1662 | 0.9809 | -0.2163 | 0.8288 |
| 18 | rs7243701 | 9642343 | ADD | 1666 | 0.8954 | -0.9926 | 0.3209 |
| 18 | rs8084083 | 11072818 | ADD | 1661 | 0.9179 | -1.246 | 0.2127 |
| 18 | rs7504649 | 11134142 | ADD | 1664 | 0.9418 | -0.8591 | 0.3903 |
| 18 | rs9303722 | 11234106 | ADD | 1658 | 0.9358 | -0.9188 | 0.3582 |
| 18 | rs7236593 | 11262254 | ADD | 1654 | 1.055 | 0.5949 | 0.5519 |
| 18 | rs11665065 | 13522208 | ADD | 1666 | 0.8147 | -2.634 | 0.008449 |
| 18 | rs4800181 | 20042745 | ADD | 1664 | 1.088 | 0.4474 | 0.6546 |
| 18 | rs10502481 | 22831048 | ADD | 1653 | 1.045 | 0.5778 | 0.5634 |
| 18 | rs7239805 | 27322066 | ADD | 1667 | 1.099 | 1.372 | 0.1699 |
| 18 | rs1439495 | 34529576 | ADD | 1666 | 0.9937 | -0.08426 | 0.9329 |
| 18 | rs2456 | 39103191 | ADD | 1667 | 1.143 | 1.811 | 0.0701 |
| 18 | rs1815937 | 45809633 | ADD | 1667 | 1.005 | 0.0709 | 0.9435 |
| 18 | rs2852095 | 45834049 | ADD | 1606 | 1.004 | 0.05848 | 0.9534 |
| 18 | rs9952908 | 45895399 | ADD | 1663 | 0.9655 | -0.4923 | 0.6225 |
| 18 | rs7233656 | 47242446 | ADD | 1664 | 0.9761 | -0.3452 | 0.73 |
| 18 | rs8089159 | 47256074 | ADD | 1667 | 0.738 | -2.619 | 0.008812 |
| 18 | rs8097646 | 53159904 | ADD | 1667 | 1.199 | 1.254 | 0.2099 |
| 18 | rs533952 | 53387340 | ADD | 1667 | 0.9903 | -0.1242 | 0.9012 |
| 18 | rs7407770 | 55749153 | ADD | 1663 | 1.064 | 0.8534 | 0.3934 |
| 18 | rs9956301 | 58929998 | ADD | 1663 | 0.9887 | -0.1002 | 0.9202 |
| 18 | rs7237857 | 60012386 | ADD | 1666 | 1.149 | 1.934 | 0.05306 |
| 18 | rs1592717 | 60133809 | ADD | 1641 | 0.8875 | -1.161 | 0.2455 |
| 18 | rs4410187 | 62923071 | ADD | 1667 | 1.056 | 0.6183 | 0.5364 |
| 18 | rs1516807 | 64297702 | ADD | 1667 | 0.9209 | -1.104 | 0.2694 |
| 18 | rs4243315 | 65484826 | ADD | 1654 | 1.167 | 1.794 | 0.07283 |
| 18 | rs7240537 | 69040825 | ADD | 1659 | 0.9083 | -1.338 | 0.1808 |
| 18 | rs7228143 | 70042132 | ADD | 1657 | 0.8219 | -1.327 | 0.1845 |
| 18 | rs1559806 | 70259767 | ADD | 1667 | 0.9353 | -0.9521 | 0.3411 |
| 18 | rs10514199 | 71869625 | ADD | 1664 | 1.028 | 0.3845 | 0.7006 |
| 18 | rs4129743 | 73808203 | ADD | 1626 | 1.04 | 0.5551 | 0.5789 |
| 18 | rs11081650 | 74566171 | ADD | 1639 | 0.8316 | -2.404 | 0.01621 |
| 18 | rs4799062 | 75400268 | ADD | 1667 | 0.7977 | -1.217 | 0.2238 |
| 19 | rs2779180 | 4792151 | ADD | 1667 | 0.9877 | -0.177 | 0.8595 |
| 19 | rs10423998 | 5496034 | ADD | 1657 | 0.9376 | -0.5739 | 0.5661 |
| 19 | rs10854117 | 8874355 | ADD | 1667 | 0.9392 | -0.8107 | 0.4176 |
| 19 | rs8113425 | 9861306 | ADD | 1667 | 1.05 | 0.6849 | 0.4934 |
| 19 | rs4926143 | 13186733 | ADD | 1667 | 0.963 | -0.4174 | 0.6764 |
| 19 | rs2112460 | 13451412 | ADD | 1666 | 1.191 | 2.497 | 0.01253 |
| 19 | rs12462428 | 16555610 | ADD | 1663 | 0.9913 | -0.1114 | 0.9113 |
| 19 | rs10422843 | 33891629 | ADD | 1667 | 1.003 | 0.04392 | 0.965 |
| 19 | rs17772344 | 34038879 | ADD | 1665 | 1.195 | 1.673 | 0.09438 |
| 19 | rs12461309 | 34059832 | ADD | 1665 | 0.9068 | -1.409 | 0.1587 |
| 19 | rs12974188 | 34066325 | ADD | 1640 | 0.9147 | -1.218 | 0.2231 |
| 19 | rs16968313 | 38823404 | ADD | 1666 | 1.061 | 0.4598 | 0.6456 |
| 19 | rs4806163 | 40695946 | ADD | 1667 | 0.9184 | -1.002 | 0.3162 |
| 19 | rs7255053 | 53200453 | ADD | 1656 | 1.029 | 0.3945 | 0.6932 |
| 19 | rs4802703 | 55576697 | ADD | 1666 | 1.041 | 0.5215 | 0.602 |
| 19 | rs12459891 | 55971694 | ADD | 1651 | 1.01 | 0.09437 | 0.9248 |
| 19 | rs17728296 | 56263880 | ADD | 1653 | 0.8843 | -1.186 | 0.2355 |
| 19 | rs10407043 | 58293461 | ADD | 1662 | 0.952 | -0.6324 | 0.5271 |
| 19 | rs1020947 | 60922956 | ADD | 1662 | 0.9598 | -0.5898 | 0.5553 |
| 19 | rs2574764 | 61226336 | ADD | 1665 | 1.099 | 1.161 | 0.2458 |
| 20 | rs4813841 | 779093 | ADD | 1655 | 1.024 | 0.3405 | 0.7335 |
| 20 | rs6083607 | 2475510 | ADD | 1666 | 1.012 | 0.1691 | 0.8657 |
| 20 | rs4815349 | 2495597 | ADD | 1667 | 1.027 | 0.3864 | 0.6992 |
| 20 | rs215529 | 2715620 | ADD | 1665 | 0.9568 | -0.6328 | 0.5268 |
| 20 | rs6115865 | 3307303 | ADD | 1650 | 1.079 | 1.027 | 0.3044 |
| 20 | rs6139130 | 3383475 | ADD | 1631 | 0.9903 | -0.1381 | 0.8902 |
| 20 | rs609203 | 3618945 | ADD | 1667 | 1.088 | 0.9914 | 0.3215 |
| 20 | rs3848818 | 4257856 | ADD | 1667 | 1.028 | 0.3383 | 0.7351 |
| 20 | rs6139746 | 5422296 | ADD | 1649 | 1.048 | 0.6392 | 0.5227 |
| 20 | rs6053621 | 5643218 | ADD | 1667 | 1.034 | 0.4026 | 0.6873 |
| 20 | rs4441549 | 5990852 | ADD | 1666 | 0.9742 | -0.3694 | 0.7118 |
| 20 | rs6053870 | 5998478 | ADD | 1610 | 0.9015 | -1.246 | 0.2126 |
| 20 | rs2149642 | 6962445 | ADD | 1667 | 1.229 | 2.607 | 0.009121 |
| 20 | rs8126205 | 13001282 | ADD | 1629 | 1.046 | 0.6136 | 0.5395 |
| 20 | rs387336 | 15194984 | ADD | 1667 | 1.017 | 0.2366 | 0.813 |
| 20 | rs6135591 | 15822488 | ADD | 1657 | 1.138 | 1.222 | 0.2216 |
| 20 | rs11700097 | 15919797 | ADD | 1666 | 0.8924 | -1.607 | 0.108 |
| 20 | rs6135697 | 16116311 | ADD | 1666 | 1.063 | 0.4775 | 0.633 |
| 20 | rs6044565 | 16930918 | ADD | 1651 | 1.068 | 0.889 | 0.374 |
| 20 | rs11697967 | 30975363 | ADD | 1667 | 1.052 | 0.6851 | 0.4933 |
| 20 | rs6088466 | 32377195 | ADD | 1661 | 1.007 | 0.09255 | 0.9263 |
| 20 | rs221308 | 34709812 | ADD | 1651 | 1.086 | 1.098 | 0.2723 |
| 20 | rs10392 | 36984349 | ADD | 1665 | 1.042 | 0.4646 | 0.6422 |
| 20 | rs1548244 | 39013047 | ADD | 1609 | 1.01 | 0.09337 | 0.9256 |
| 20 | rs6124439 | 40353349 | ADD | 1663 | 0.9324 | -0.8895 | 0.3738 |
| 20 | rs4812591 | 40427440 | ADD | 1665 | 0.952 | -0.7018 | 0.4828 |
| 20 | rs1062943 | 41603741 | ADD | 1660 | 0.9008 | -1.418 | 0.1562 |
| 20 | rs1998033 | 42992323 | ADD | 1667 | 0.9751 | -0.3554 | 0.7223 |
| 20 | rs910187 | 45274459 | ADD | 1667 | 0.9574 | -0.585 | 0.5585 |
| 20 | rs1591168 | 45760849 | ADD | 1658 | 1.004 | 0.05038 | 0.9598 |
| 20 | rs6018788 | 45971701 | ADD | 1662 | 1.029 | 0.4167 | 0.6769 |
| 20 | rs4141783 | 47212009 | ADD | 1652 | 0.8372 | -1.589 | 0.112 |
| 20 | rs944932 | 48307784 | ADD | 1663 | 1.012 | 0.1229 | 0.9022 |
| 20 | rs1810812 | 48452841 | ADD | 1667 | 0.8873 | -1.619 | 0.1055 |
| 20 | rs8119787 | 49580478 | ADD | 1653 | 0.9953 | -0.06628 | 0.9472 |
| 20 | rs6021631 | 50068834 | ADD | 1666 | 0.918 | -1.224 | 0.2211 |
| 20 | rs6013355 | 50071067 | ADD | 1667 | 1.104 | 1.15 | 0.2503 |
| 20 | rs6126919 | 51733766 | ADD | 1665 | 1.008 | 0.1118 | 0.911 |
| 20 | rs2426689 | 54803001 | ADD | 1665 | 1.124 | 1.637 | 0.1016 |
| 20 | rs230191 | 55206090 | ADD | 1666 | 1.005 | 0.06959 | 0.9445 |
| 20 | rs6128241 | 56010987 | ADD | 1659 | 0.9732 | -0.3123 | 0.7548 |
| 20 | rs6026872 | 57420894 | ADD | 1665 | 0.9935 | -0.05422 | 0.9568 |
| 20 | rs868544 | 59716357 | ADD | 1651 | 0.9653 | -0.5058 | 0.613 |
| 20 | rs6142886 | 59944585 | ADD | 1665 | 1.024 | 0.2917 | 0.7705 |
| 20 | rs6142711 | 60095481 | ADD | 1667 | 1.035 | 0.4858 | 0.6271 |
| 21 | rs4816223 | 14369083 | ADD | 1666 | 1.069 | 0.7552 | 0.4501 |
| 21 | rs2826492 | 21023059 | ADD | 1666 | 0.9069 | -0.9448 | 0.3448 |
| 21 | rs2226698 | 21494175 | ADD | 1657 | 1.166 | 1.239 | 0.2154 |
| 21 | rs1984333 | 23606950 | ADD | 1652 | 1.122 | 1.433 | 0.1518 |
| 21 | rs454017 | 26186455 | ADD | 1667 | 0.978 | -0.2634 | 0.7922 |
| 21 | rs2250910 | 28725372 | ADD | 1667 | 1.059 | 0.566 | 0.5714 |
| 21 | rs2832394 | 29840646 | ADD | 1589 | 1.112 | 1.439 | 0.1501 |
| 21 | rs2832438 | 30059808 | ADD | 1664 | 1.071 | 0.7788 | 0.4361 |
| 21 | rs459617 | 30062624 | ADD | 1666 | 1.105 | 1.305 | 0.1919 |
| 21 | rs2833556 | 32207171 | ADD | 1667 | 1.133 | 1.632 | 0.1026 |
| 21 | rs2833592 | 32281852 | ADD | 1667 | 0.9146 | -1.011 | 0.312 |
| 21 | rs2834371 | 34348725 | ADD | 1661 | 1.082 | 1.127 | 0.2596 |
| 21 | rs8127044 | 39216204 | ADD | 1664 | 1.094 | 1.029 | 0.3035 |
| 21 | rs2837774 | 40962525 | ADD | 1662 | 1.05 | 0.6887 | 0.491 |
| 21 | rs2026273 | 41000709 | ADD | 1663 | 1.017 | 0.1471 | 0.883 |
| 21 | rs7276043 | 41007790 | ADD | 1667 | 0.9262 | -0.6952 | 0.4869 |
| 21 | rs460976 | 41757364 | ADD | 1641 | 1.357 | 1.908 | 0.0564 |
| 21 | rs2269139 | 42977018 | ADD | 1650 | 0.9966 | -0.02995 | 0.9761 |
| 21 | rs9653818 | 43073927 | ADD | 1667 | 1.058 | 0.5765 | 0.5643 |
| 21 | rs6586252 | 43149456 | ADD | 1664 | 1.02 | 0.2813 | 0.7785 |
| 22 | rs5747087 | 16246198 | ADD | 1666 | 0.9505 | -0.7004 | 0.4837 |
| 22 | rs2252257 | 17020300 | ADD | 1661 | 1.031 | 0.3725 | 0.7095 |
| 22 | rs5761913 | 19095021 | ADD | 1660 | 0.9745 | -0.3191 | 0.7497 |
| 22 | rs7288210 | 25272127 | ADD | 1651 | 1.128 | 1.666 | 0.09579 |
| 22 | rs4822836 | 25768108 | ADD | 1667 | 1.021 | 0.1598 | 0.873 |
| 22 | rs713812 | 25773584 | ADD | 1663 | 1.069 | 0.8015 | 0.4229 |
| 22 | rs6006427 | 28999993 | ADD | 1665 | 1.053 | 0.6603 | 0.5091 |
| 22 | rs2066926 | 29198102 | ADD | 1599 | 1.048 | 0.5111 | 0.6093 |
| 22 | rs929036 | 30669213 | ADD | 1666 | 0.9882 | -0.1686 | 0.8661 |
| 22 | rs4821544 | 35588449 | ADD | 1654 | 1.039 | 0.5273 | 0.598 |
| 22 | rs6006632 | 42778379 | ADD | 1614 | 1.003 | 0.04581 | 0.9635 |
| 22 | rs5764076 | 42790778 | ADD | 1660 | 0.7922 | -1.892 | 0.05848 |
| 22 | rs16992075 | 43128674 | ADD | 1663 | 0.9288 | -0.7587 | 0.448 |
| 22 | rs1557553 | 43139648 | ADD | 1635 | 1.104 | 0.9931 | 0.3206 |
| 22 | rs9614868 | 43144976 | ADD | 1664 | 0.9896 | -0.1486 | 0.8818 |
| 22 | rs7285004 | 43153364 | ADD | 1657 | 1.025 | 0.3393 | 0.7344 |
| 22 | rs929048 | 46230421 | ADD | 1665 | 0.9825 | -0.1242 | 0.9012 |
| 22 | rs2337497 | 47565552 | ADD | 1664 | 0.9097 | -1.392 | 0.1638 |
| 22 | rs738413 | 48478152 | ADD | 1664 | 1.005 | 0.05835 | 0.9535 |
| 22 | rs1555048 | 49030475 | ADD | 1654 | 1.012 | 0.1462 | 0.8837 |
