# Supplementary material for: Genome-wide association study of antidepressant response: involvement of the inorganic cation transmembrane transporter activity pathway
Source: BMC Psychiatry. 2016 Apr 18;16:106. doi: 10.1186/s12888-016-0813-x (PMC4836090; doi:10.1186/s12888-016-0813-x)
Supplement: Additional file 9: Table S7. — Description and statistics referred to the SNPs that showed p<0.05 (response phenotype) in both the analyzed samples. (DOC 157 kb) [file 12888_2016_813_MOESM9_ESM.doc]

**Table S7**: description and statistics referred to the SNPs that showed p<0.05 (response phenotype) in both the analyzed samples.

|  |  | **STAR*D sample** | | | | **Korean sample** | | | | **Annotation** |
| --- | --- | --- | --- | --- | --- | --- | --- | --- | --- | --- |
| **SNP** | **Chr** | **position** | **odds ratio** | **statistic** | **p** | **position** | **odds ratio** | **statistic** | **p** |  |
| rs10100760 | 8 | 9407969 | 0.8542 | -2.216 | 0.02669 | 9370559 | 2.524 | 1.985 | 0.04718 | . |
| rs10199560 | 2 | 74296559 | 1.34 | 2.05 | 0.04041 | 74443051 | 0.2925 | -2.449 | 0.01434 | SLC4A5(-0.317kb)|MTHFD2(+0.627kb) |
| rs10483263 | 14 | 21448675 | 1.325 | 2.647 | 0.008112 | 22378835 | 1.965 | 2.191 | 0.02848 | . |
| rs10491846 | 9 | 88352001 | 1.164 | 2.205 | 0.02742 | 89162181 | 3.461 | 2.063 | 0.03912 | . |
| rs10498479 | 14 | 55415998 | 0.7046 | -2.456 | 0.01406 | 56346245 | 2.414 | 2.073 | 0.03813 | . |
| rs10768703 | 11 | 41522604 | 1.245 | 1.992 | 0.04641 | 41566028 | 4.39 | 2.474 | 0.01337 | . |
| rs10823772 | 10 | 72934436 | 0.8348 | -2.203 | 0.02762 | 73264430 | 2.371 | 2.274 | 0.02296 | CDH23(0) |
| rs10994675 | 10 | 51233999 | 1.155 | 2.069 | 0.03856 | 51563993 | 0.5495 | -2.04 | 0.04136 | TIMM23B(0)|NCOA4(-1.114kb)|MSMB(+1.475kb) |
| rs10997242 | 10 | 68006301 | 1.301 | 2.788 | 0.005303 | 68336295 | 0.3246 | -3.025 | 0.002489 | CTNNA3(0) |
| rs11081650 | 18 | 74566171 | 0.8316 | -2.404 | 0.01621 | 76465183 | 2.268 | 2.113 | 0.03463 | . |
| rs11176078 | 12 | 64861524 | 0.7681 | -3.293 | 0.0009895 | 66575257 | 1.864 | 1.963 | 0.04959 | TMBIM4(+11.4kb)|IRAK3(-7.72kb)|=missense |
| rs11177718 | 12 | 68241952 | 0.714 | -1.962 | 0.0498 | 69955685 | 1.937 | 2.228 | 0.02585 | FRS2(0) |
| rs11189937 | 10 | 100770705 | 1.441 | 2.891 | 0.003841 | 100780715 | 3.546 | 2.177 | 0.02952 | HPSE2(0) |
| rs1151899 | 12 | 115299808 | 0.8577 | -2.141 | 0.03228 | 116815425 | 0.482 | -2.169 | 0.03006 | . |
| rs11618012 | 13 | 34237417 | 1.378 | 3.528 | 0.0004188 | 35339417 | 1.865 | 1.98 | 0.04774 | . |
| rs11654929 | 17 | 72820946 | 1.234 | 2.515 | 0.01191 | 75309351 | 2.17 | 2.141 | 0.03229 | SEPT9(0) |
| rs11665065 | 18 | 13522208 | 0.8147 | -2.634 | 0.008449 | 13532208 | 0.2098 | -2.095 | 0.03614 | LDLRAD4(0) |
| rs11735430 | 4 | 71060137 | 1.16 | 2.159 | 0.03085 | 71025548 | 1.926 | 2.004 | 0.04507 | PRR27(0)|CSN1S2BP(+13.13kb) |
| rs11737660 | 4 | 149243863 | 0.842 | -1.991 | 0.04653 | 149024413 | 1.853 | 2.136 | 0.03266 | NR3C2(0) |
| rs12498882 | 4 | 138600718 | 1.218 | 2.843 | 0.004476 | 138381268 | 2.02 | 2.36 | 0.01826 | . |
| rs12990473 | 2 | 124170989 | 1.205 | 1.976 | 0.04817 | 124454519 | 0.5455 | -2.065 | 0.03892 | . |
| rs1331697 | 13 | 30031133 | 1.246 | 3.057 | 0.002239 | 31133133 | 2.015 | 2.359 | 0.01834 | . |
| rs1460930 | 8 | 101183970 | 0.8663 | -2.003 | 0.04518 | 101114794 | 2.36 | 2.716 | 0.006598 | RGS22(0) |
| rs1598492 | 15 | 99395057 | 0.8326 | -2.356 | 0.01847 | 101577534 | 0.3826 | -2.569 | 0.01019 | LRRK1(0) |
| rs16873129 | 7 | 22111609 | 1.323 | 2.103 | 0.03547 | 22145084 | 0.319 | -2.912 | 0.003597 | RAPGEF5(-12.82kb) |
| rs17076923 | 5 | 173543990 | 1.341 | 2.276 | 0.02283 | 173611384 | 2.374 | 2.48 | 0.01314 | . |
| rs17759236 | 17 | 51534381 | 0.8539 | -2.2 | 0.02779 | 54179382 | 0.5131 | -2.17 | 0.02998 | . |
| rs1999121 | 9 | 134592548 | 1.175 | 1.969 | 0.04893 | 135602727 | 0.2693 | -2.602 | 0.009258 | AK8(0) |
| rs2046535 | 4 | 186307426 | 0.8612 | -2.17 | 0.02997 | 186070432 | 1.925 | 2.028 | 0.04254 | SLC25A4(0)|KIAA1430(-10.38kb)|=missense |
| rs2112460 | 19 | 13451412 | 1.191 | 2.497 | 0.01253 | 13590412 | 2.844 | 2.759 | 0.005792 | CACNA1A(0) |
| rs2149642 | 20 | 6962445 | 1.229 | 2.607 | 0.009121 | 7014445 | 1.828 | 2.017 | 0.0437 | . |
| rs219456 | 4 | 109540178 | 1.156 | 2.043 | 0.04103 | 109320729 | 0.4857 | -2.006 | 0.04482 | . |
| rs2279980 | 5 | 57949136 | 1.228 | 2.609 | 0.009071 | 57913379 | 0.5516 | -2.06 | 0.03944 | RAB3C(0) |
| rs2299267 | 7 | 94899857 | 1.262 | 2.486 | 0.01293 | 95061921 | 0.4645 | -2.858 | 0.004265 | PON2(0) |
| rs2350516 | 6 | 65816297 | 1.252 | 1.983 | 0.04737 | 65759576 | 0.53 | -2.202 | 0.02766 | EYS(0) |
| rs2392147 | 7 | 32833248 | 1.156 | 2.16 | 0.03074 | 32866723 | 0.4857 | -2.409 | 0.01599 | . |
| rs2532560 | 12 | 4032132 | 1.237 | 2.423 | 0.01537 | 4161871 | 0.3256 | -2.508 | 0.01215 | . |
| rs2589967 | 15 | 88708880 | 1.184 | 2.068 | 0.03866 | 90907876 | 0.5333 | -2.043 | 0.04102 | ZNF774(+3.161kb)|GABARAPL3(+15.2kb) |
| rs26217 | 5 | 14587994 | 0.7365 | -2.739 | 0.006159 | 14534994 | 0.4294 | -2.382 | 0.01721 | . |
| rs2776632 | 10 | 30252761 | 1.171 | 2.255 | 0.02412 | 30212755 | 0.5142 | -2.096 | 0.03609 | . |
| rs322295 | 7 | 136571602 | 1.157 | 2.094 | 0.03622 | 136921062 | 0.485 | -2.335 | 0.01953 | PTN(0) |
| rs3922982 | 9 | 136726816 | 0.7969 | -2.328 | 0.01993 | 137586995 | 0.176 | -2.144 | 0.03205 | COL5A1(0) |
| rs489257 | 11 | 78768875 | 0.8516 | -2.197 | 0.028 | 79091227 | 1.853 | 2.066 | 0.03882 | TENM4(0) |
| rs4937775 | 11 | 132822925 | 1.15 | 2.069 | 0.03854 | 133317715 | 0.5441 | -2.027 | 0.04262 | OPCML(0) |
| rs49411 | 3 | 59732615 | 0.8522 | -2.277 | 0.02276 | 59757575 | 0.3625 | -3.292 | 0.0009939 | FHIT(0) |
| rs512774 | 9 | 1699308 | 1.277 | 2.138 | 0.0325 | 1709308 | 0.1819 | -2.083 | 0.03729 | . |
| rs521093 | 1 | 110169753 | 1.209 | 2.096 | 0.03604 | 110368230 | 5.13 | 2.728 | 0.006376 | . |
| rs582854 | 13 | 46343878 | 0.8641 | -2.041 | 0.04123 | 47445877 | 2.144 | 2.344 | 0.01908 | HTR2A-AS1(+15.44kb)|HTR2A(0) |
| rs605265 | 12 | 116620279 | 1.22 | 2.821 | 0.004786 | 118135896 | 1.755 | 2.075 | 0.03803 | KSR2(0) |
| rs6479457 | 9 | 94950329 | 1.187 | 2.364 | 0.01809 | 95910508 | 4.377 | 2.675 | 0.007471 | NINJ1(+13.94kb) |
| rs6586734 | 8 | 18238667 | 1.287 | 3.179 | 0.001476 | 18194387 | 1.845 | 2.113 | 0.03464 | . |
| rs663177 | 10 | 13131552 | 0.8514 | -2.179 | 0.02932 | 13091546 | 0.5196 | -2.158 | 0.03089 | CCDC3(0) |
| rs6746088 | 2 | 107353467 | 1.201 | 2.674 | 0.007489 | 107987035 | 2.003 | 2.012 | 0.04426 | . |
| rs6757604 | 2 | 119961173 | 1.149 | 1.993 | 0.0463 | 120244703 | 1.975 | 2.194 | 0.02827 | SCTR(0) |
| rs6769829 | 3 | 6492587 | 1.521 | 2.237 | 0.02529 | 6517587 | 0.4344 | -2.075 | 0.03799 | . |
| rs6966038 | 7 | 156573159 | 0.7525 | -3.451 | 0.0005578 | 156880398 | 0.1935 | -2.002 | 0.04532 | . |
| rs697482 | 6 | 166341627 | 1.219 | 2.292 | 0.02189 | 166421637 | 0.3858 | -2.206 | 0.02737 | LINC00602(+18.53kb) |
| rs7138803 | 12 | 48533735 | 1.159 | 1.971 | 0.04869 | 50247468 | 2.008 | 1.977 | 0.0481 | FAIM2(-13.21kb)|BCDIN3D-AS1(+12.53kb)|BCDIN3D(+10.56kb) |
| rs7194966 | 16 | 12635395 | 0.8432 | -2.369 | 0.01782 | 12727894 | 2.56 | 2.037 | 0.04163 | . |
| rs725761 | 4 | 61594457 | 1.179 | 2.158 | 0.03093 | 61911862 | 2.153 | 2.172 | 0.02989 | . |
| rs7459368 | 7 | 69726632 | 0.7904 | -3.377 | 0.000733 | 70088696 | 0.4199 | -2.152 | 0.03137 | AUTS2(0) |
| rs7561228 | 2 | 143982974 | 1.194 | 2.194 | 0.02821 | 144266504 | 0.5304 | -2.062 | 0.03919 | ARHGAP15(0) |
| rs7573672 | 2 | 59008609 | 1.188 | 2.345 | 0.01901 | 59155105 | 2.105 | 2.388 | 0.01692 | LINC01122(0) |
| rs7616467 | 3 | 25483432 | 1.175 | 2.24 | 0.02508 | 25508428 | 0.4754 | -1.994 | 0.0462 | RARB(0) |
| rs7737692 | 5 | 1514167 | 1.29 | 3.487 | 0.0004887 | 1461167 | 2.01 | 2.404 | 0.01621 | SLC6A3(+15.62kb)|LPCAT1(-0.374kb) |
| rs7782195 | 7 | 77957867 | 0.8218 | -2.723 | 0.006462 | 78119931 | 0.3793 | -2.658 | 0.007871 | MAGI2(0) |
| rs7871600 | 9 | 28930111 | 1.218 | 2.236 | 0.02534 | 28940111 | 0.397 | -2.177 | 0.02952 | LINGO2(0) |
| rs7911727 | 10 | 123214869 | 1.156 | 2.067 | 0.03877 | 123224879 | 0.4637 | -2.173 | 0.02979 | FGFR2(-12.96kb) |
| rs8031166 | 15 | 21633826 | 1.228 | 2.828 | 0.004678 | 24082733 | 0.3259 | -2.693 | 0.007085 | . |
| rs8089159 | 18 | 47256074 | 0.738 | -2.619 | 0.008812 | 49002076 | 3.591 | 2.094 | 0.03629 | LOC100287225(0) |
| rs847440 | 7 | 16984957 | 0.8029 | -3.038 | 0.002382 | 17018432 | 0.4063 | -2.482 | 0.01306 | . |
| rs9300342 | 13 | 95749951 | 1.18 | 2.358 | 0.01835 | 96951950 | 0.5241 | -2.104 | 0.03534 | HS6ST3(0) |
| rs9301862 | 13 | 92656760 | 1.224 | 2.896 | 0.003783 | 93858759 | 0.5402 | -2.032 | 0.0422 | . |
| rs9315310 | 13 | 34249431 | 1.229 | 2.364 | 0.01807 | 35351431 | 2.273 | 2.512 | 0.012 | . |
| rs9813383 | 3 | 59406382 | 1.282 | 2.36 | 0.01825 | 59431342 | 0.5193 | -2.13 | 0.03313 | . |
| rs9855392 | 3 | 131415936 | 0.8147 | -2.339 | 0.01933 | 129933246 | 2.222 | 2.24 | 0.02506 | COL6A4P2(0) |
| rs9881693 | 3 | 14286277 | 1.217 | 2.212 | 0.02697 | 14311273 | 2.101 | 2.349 | 0.0188 | . |
