# Supplementary material for: Genome-wide association study of antidepressant response: involvement of the inorganic cation transmembrane transporter activity pathway
Source: BMC Psychiatry. 2016 Apr 18;16:106. doi: 10.1186/s12888-016-0813-x (PMC4836090; doi:10.1186/s12888-016-0813-x)
Supplement: Additional file 11: Table S8. — Top SNPs in the Korean sample (p<10e-05) for the response (A) and remission (B) phenotypes. When a SNP is localized in an intergenic region, the nearest gene is reported with distance in parenthesis. MAF=minor allele frequency. Chr=chromosome. (DOCX 17 kb) [file 12888_2016_813_MOESM11_ESM.docx]

**Table S8**: top SNPs in the Korean sample (p<10e-05) for the response (**A**) and remission (**B**) phenotypes. When a SNP is localized in an intergenic region, the nearest gene is reported with distance in parenthesis. MAF=minor allele frequency. Chr=chromosome.

**A**

| **SNP** | **Chr:base pair** | **Gene (distance)** | **MAF** | **OR** | **Stat** | **P** |
| --- | --- | --- | --- | --- | --- | --- |
| rs12474003 | 2:184360632 | NUP35 (334 Kbp) | 0.29 | 0.20 | -4.29 | 1.79e-05 |
| rs1236910 | 11:115598377 | CADM1 (223 Kbp) | 0.27 | 5.00 | 4.08 | 4.50e-05 |
| rs6820424 | 4:52735484 | DCUN1D4 (0) | 0.50 | 3.76 | 4.05 | 5.11e-05 |
| rs1827489 | 15:82390989 | LOC105370924 (0)  EFL1 (164 Kbp) | 0.44 | 0.26 | -4.03 | 5.71e-05 |
| rs11103693 | 9:138016657 | OLFM1 (3627 bp) | 0.49 | 0.27 | -3.95 | 7.86e-05 |
| rs4700947 | 5:179801752 | GFPT2 (21 Kbp) | 0.34 | 3.99 | 3.92 | 8.74e-05 |
| rs12623821 | 2: 184389762 | NUP35 (363 Kbp) | 0.28 | 0.23 | -3.91 | 9.24e-05 |

**B**

| **SNP** | **Chr:base pair** | **Gene (distance)** | **MAF** | **OR** | **Stat** | **P** |
| --- | --- | --- | --- | --- | --- | --- |
| rs17019491 | 1:212804965 | FAM71A (4845 bp) | 0.31 | 0.11 | -4.55 | 5.47e-06 |
| rs3744306 | 17:66426505 | WIPI1 (0)  PRKAR1A (0) | 0.22 | 0.09 | -4.10 | 4.18e-05 |
| rs739172 | 22:45161740 | PRR5-ARHGAP8 (0)  ARHGAP8 (0) | 0.17 | 14.35 | 4.04 | 5.32e-05 |
| rs6820424 | 4:52735484 | DCUN1D4 (0) | 0.50 | 5.24 | 4.03 | 5.43e-05 |
| rs76185620 | 17:66438342 | WIPI1 (0)  PRKAR1A (0) | 0.25 | 0.14 | -3.99 | 6.67e-05 |
| rs2501846 | 1:212841934 | BATF3 (18 Kbp) | 0.35 | 0.21 | -3.98 | 6.93e-05 |
| kgp4264378 | 4:25735428 | SEL1L3 (14 Kbp) | 0.17 | 0.08 | -3.97 | 7.34e-05 |
| rs7969076 | 12:94096042 | CRADD (0) | 0.35 | 8.77 | 3.95 | 7.91e-05 |
| rs9555742 | 13:111569220 | ANKRD10 (1766 bp) | 0.31 | 0.20 | -3.95 | 7.95e-05 |
| rs4789626 | 17:72167585 | RPL38 (32 Kbp) | 0.36 | 7.08 | 3.91 | 9.28e-05 |
| rs2385199 | 2: 219750746 | WNT10A (0) | 0.32 | 0.13 | -3.90 | 9.75e-05 |
| rs2630441 | 3:13559567 | HDAC11 (12 Kbp) | 0.45 | 7.55 | 3.89 | 9.91e-05 |
